# FYN and ABL Regulate the Interaction Networks of the DCBLD Receptor Family

**DOI:** 10.1074/mcp.RA120.002163

**Published:** 2020-11-25

**Authors:** Anna M. Schmoker, Jaye L. Weinert, Jacob M. Markwood, Kathryn S. Albretsen, Michelle L. Lunde, Marion E. Weir, Alicia M. Ebert, Karen L. Hinkle, Bryan A. Ballif

**Affiliations:** 1 Department of Biology, University of Vermont, Marsh Life Sciences, Burlington, Vermont, USA; 2 Department of Biology, Norwich University, Northfield, Vermont, USA

**Keywords:** Phosphorylation, mass spectrometry, protein-protein interactions, label-free quantification, SILAC, 14-3-3, ABL, DCBLD, ESDN, FYN

## Abstract

The Discoidin, CUB, and LCCL domain-containing protein (DCBLD) family consists of two type-I transmembrane scaffolding receptors, DCBLD1 and DCBLD2, which play important roles in development and cancer. The nonreceptor tyrosine kinases FYN and ABL are known to drive phosphorylation of tyrosine residues in YXXP motifs within the intracellular domains of DCBLD family members, which leads to the recruitment of the Src homology 2 (SH2) domain of the adaptors CT10 regulator of kinase (CRK) and CRK-like (CRKL). We previously characterized the FYN- and ABL-driven phosphorylation of DCBLD family YXXP motifs. However, we have identified additional FYN- and ABL-dependent phosphorylation sites on DCBLD1 and DCBLD2. This suggests that beyond CRK and CRKL, additional DCBLD interactors may be regulated by FYN and ABL activity. Here, we report a quantitative proteomics approach in which we map the FYN- and ABL-regulated interactomes of DCBLD family members. We found FYN and ABL regulated the binding of several signaling molecules to DCBLD1 and DCBLD2, including members of the 14-3-3 family of adaptors. Biochemical investigation of the DCBLD2/14-3-3 interaction revealed ABL-induced binding of 14-3-3 family members directly to DCBLD2.

## Teaser

The FYN- and ABL-regulated interactomes of DCBLD proteins have been determined using quantitative mass spectrometry-based proteomics. FYN and ABL drove the binding of several signaling molecules to DCBLD1 and DCBLD2, including members of the 14-3-3 family of adaptors. Biochemical investigation of the DCBLD2/14-3-3 interaction revealed ABL-induced binding of 14-3-3 family members directly to DCBLD2.The interactions identified in this screen present important steps in elucidating signaling events involving DCBLD proteins in developmental and oncogenic signaling.

## Graphical Abstract

**Figure:**
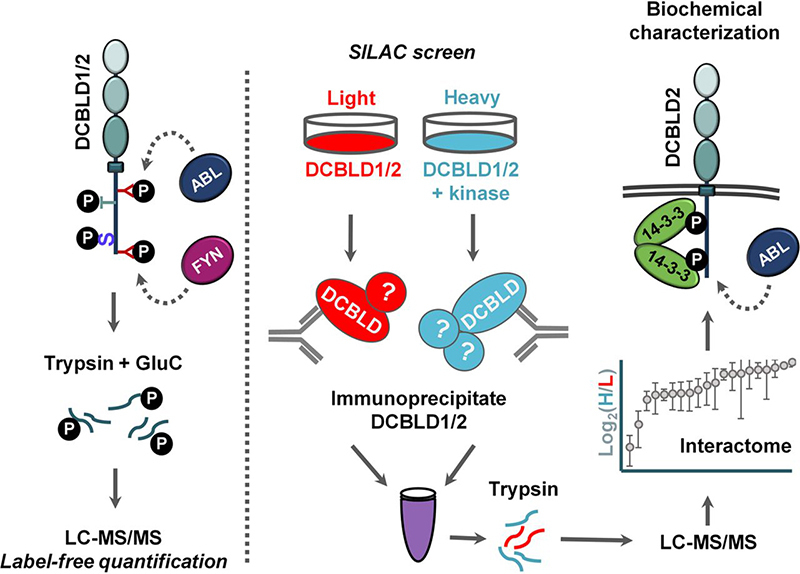

**Highlights**

• FYN and ABL differentially regulate DCBLD Ser/Thr/Tyr phosphorylation.
• DCBLD1 and DCBLD2 interactomes are modulated by FYN and ABL.
• ABL drives a direct DCBLD2/14-3-3 interaction.

The Discoidin, CUB, and LCCL domain-containing protein (DCBLD) receptor family is composed of two single-pass transmembrane proteins that play integral roles in vertebrate development and disease (1). Both family members possess CUB, LCCL, and coagulation factor FV/FVIII (Discoidin) extracellular domains, as well as a cytoplasmic scaffolding region (1). Although they remain orphan receptors, there is evidence that a class IV semaphorin, SEMA4B, may be a ligand for DCBLD2 (2). DCBLD2 is also known to modulate signaling of receptor tyrosine kinases (RTKs), including vascular endothelial growth factor receptor 2 (VEGFR2) and platelet derived growth factor receptor β (PDGFRβ) in blood vessel development and repair (3, 4, 5), insulin receptor (INSR) in glucose homeostasis (6), and epidermal growth factor receptor (EGFR) in oncogenesis and cancer progression (7, 8). However, the molecular mechanisms of DCBLD2 action on RTK signaling are not well understood.

The intracellular scaffolding domains of DCBLD family members harbor seven (DCBLD2) and eight (DCBLD1) intracellular binding motifs of the Src homology 2 (SH2) domains of the signaling adaptors CT10 regulator of kinase (CRK) and CRK-like (CRKL). Binding of the CRK/CRKL-SH2 domain requires tyrosine phosphorylation within these YXXP motifs, as mutation of all such motifs via tyrosine-to-phenylalanine substitutions prevents their phosphorylation and abolishes the SH2-mediated DCBLD-CRK/CRKL interaction (9, 10). The nonreceptor tyrosine kinases FYN and ABL can drive phosphorylation of tyrosines within these YXXP motifs, thereby inducing the interaction between DCBLD proteins and the SH2 domain of CRK/CRKL (1, 9). FYN (11, 12) and ABL (13) can be activated downstream of RTKs and in many other signaling pathways. Thus, FYN and ABL activity could regulate proteins in complex with DCBLD proteins and thereby engage DCBLD proteins to participate in RTK or other signal transduction mechanisms. To identify and measure the networks of proteins that dynamically interact with DCBLD family members dependent on FYN and ABL, we employed affinity chromatography and quantitative liquid chromatography-tandem MS (LC–MS/MS).

## EXPERIMENTAL PROCEDURES

##### Materials

Penicillin/Streptomycin 100 × solution and Dulbecco's Modified Eagle Medium (DMEM) were obtained from Mediatech (Manassas, VA). DMEM for stable isotope labeling of amino acids in cell culture (SILAC) and kanamycin sulfate were acquired from Thermo Scientific (Waltham, MA), and fetal bovine serum (FBS), dialyzed FBS for SILAC experiments, and cosmic calf serum (CCS) were purchased from Hyclone (Logan, Utah). Heavy labeled l-arginine (^13^C_6_, ^15^N_4_) and l-lysine (^13^C_6_, ^15^N_2_) were obtained from Cambridge Isotope Laboratories (Tewksbury, MA), and unlabeled amino acids were purchased from MP Biomedicals (Santa Ana, CA). The sequencing-grade trypsin used in enzymatic digests before LC–MS/MS analysis was from Promega (Madison, WI). The BSA standard for Bradford assays and the Bradford Reagent were obtained from Amresco Life Sciences, LLC. (Cleveland, OH). Protein G resin was obtained from G-Biosciences (St. Louis, MO). Protein A resin and enhanced chemiluminescence (ECL) reagents were purchased from Pierce (Rockford, IL), and x-ray film was from Denville scientific (Metuchen, NJ). Packing material used for HPLC was purchased from Michrom Bioresources Inc. (Auburn, CA). Nitrocellulose membranes were from GVS Life Sciences (Sanford, ME). All additional reagents were purchased from Sigma (St. Louis, MO) unless otherwise noted.

##### Plasmids

The mammalian expression construct for full-length human DCBLD2 (RC224483) in pCMV6-Entry, tagged with FLAG and MYC sequences at C termini, were obtained from OriGene (Rockville, MD). The C-terminal MYC- and FLAG-tagged human DCBLD1 construct in pCMV6 vectors was synthesized by Bio Basic Inc. (Markham, ON). The human c-ABL construct, with a C-terminal FLAG tag was kindly gifted by A. Howe (U. of Vermont), originally constructed in the Kufe laboratory(Harvard Medical School) (14). The bacterial expression plasmids encoding the fusion of GSH S-transferase with 14-3-3β (Addgene # 13276), 14-3-3ε (Addgene # 13279), 14-3-3ε K49E (Addgene # 11945), 14-3-3ζ (Addgene # 13278), 14-3-3σ (Addgene # 11944) were kind gifts from M. Yaffe (15, 16).

##### Antibodies

The mouse α-Flag (M2) antibody (Ab) and Affinity Gel were from Sigma and the free Ab was used for Western blotting at a concentration of 0.5 μg/ml. The rabbit α-GST (1:2000 for Western blotting) was also from Sigma. Cell Signaling Technologies Inc. (Danvers, MA) was the source of the following antibodies, used at 1:1000 dilutions: α-FLAG (M2, rabbit mAb), α-MYC (71D10, rabbit mAb), α-FYN (rabbit polyclonal), α-pTyr416-Src (D49G4, rabbit mAb), α-α-tubulin (DM1A, mouse mAb), α-pTyr412-ABL (247C7, rabbit mAb), α-c-ABL (rabbit polyclonal), α-RXXpS/pT (110B7E, rabbit mAb), and α-RXXpSXP (rabbit polyclonal). The α-pY (4G10; 1:1000) was from EMD Millipore (Billerica, MA). For immunoblotting all primary antibodies were diluted in 1.5% BSA in Tris-Buffered Saline (0.9% NaCl, 0.4% Tris-HCl, and 0.1% Tris-base) with 0.05% Tween 20 (TBS-T) and containing 0.005% sodium azide. Horseradish peroxidase (HRP)-conjugated secondary antibodies were obtained from EMD Millipore and used at the following concentrations: goat α-mouse IgG-HRP (1:5,000), light-chain-specific goat α-mouse and α-rabbit IgG-HRP (1:10,000), and goat α-rabbit IgG-HRP (1:15,000). All secondary antibodies were diluted in TBS-T.

##### Cell Culture, Transfection, and Lysis

Adenovirus early region 1A (E1A)-transformed HEK 293 cells were cultured in DMEM supplemented with 5% each of FBS and CCS, 50 U/ml penicillin, and 50 μg/ml streptomycin at 37˚C in 5% atmospheric CO_2_. For SILAC experiments HEK 293 cells were cultured in labeled (heavy) or unlabeled (light) growth medium for at least 1 week before transfection to ensure full incorporation of stable isotopes into proteins. SILAC media, lacking l-lysine and l-arginine were supplemented with 10% dialyzed FBS and antibiotics as stated above, and with 60 mg/L unlabeled l-proline, 100 mg/L of l-lysine either unlabeled or labeled (^13^C_6_, ^15^N_2_), and 100 mg/L of l-arginine either unlabeled or labeled (^13^C_6_, ^15^N_4_).

HEK 293 cells were grown to 60% of confluence before transfection via calcium phosphate precipitation. The following amount of plasmid was transfected per 10 cm dish: WT and mutant DCBLD1 (10 μg) and DCBLD2 (6 μg), WT and mutant FYN (1.5 μg), and WT c-ABL (1 μg), WT- or ΔSH3-ABL:ER (4 μg). Six hours post-transfection, cells were washed with PBS (PBS) and returned to full medium overnight before lysis. Cells were placed on ice and immediately washed with PBS (4˚C) before lysis in Brain Complex Lysis Buffer (BCLB: 25 mm Tris pH 7.2, 137 mm NaCl, 10% glycerol, 1% igepal, 25 mm NaF, 10 mm Na_2_H_2_P_2_O_7_, 1 mm Na_3_VO_4_, 1 mm phenylmethylsulfonyl fluoride (PMSF), 10 μg/ml each of leupeptin and pepstatin-A). Lysates were centrifuged and the supernatant was reserved for immunoprecipitation or immunoblotting.

##### Immunoprecipitation and SDS-PAGE for Immunoblotting

Protein concentration was determined as described above. For immunoprecipitations, normalized lysates (1 μg/μL) were incubated separately with α-FLAG Affinity Gel (10 μL of a 50% slurry) or the α-MYC antibody with a 50/50 mixture of protein A and G resin (20 μL of a 50% slurry) overnight, rocking at 4˚C. Beads were washed three times with BCLB, after which bound proteins were eluted and denatured in 25 μL sample buffer at 95˚C for 5 min. For whole cell extracts (WCEs), normalized lysates were denatured in sample buffer (final concentration of 0.75 μg/μL) at 95˚C for 5 min. Immunoprecipitations and WCEs (15 μg per well) were separated on 10% acrylamide gels with 4.2% acrylamide stacking gels, as described above. Following separation, proteins were transferred onto nitrocellulose membranes in a submersible transfer unit at 4˚C in 1.13% glycine, 0.25% Tris-base and 20% methanol. Membranes were stained with a reversible Ponceau stain to assess total protein levels. Membranes were blocked with 5% nonfat dry milk in TBS-T and incubated in primary antibody solutions overnight at 4˚C. Membranes were then washed and incubated with HRP-conjugated secondary antibody solution for three hours at 25˚C. Membranes were briefly incubated in ECL reagents and exposed to x-ray film.

##### GST-14-3-3 far-Western Blotting

To generate GST-14-3-3-conjugated GSH beads, 50 ml cultures of Luria Broth (LB; 0.5% Tryptone, 0.5% yeast extract, 171 mm NaCl) containing 50 μg/ml ampicillin were inoculated with *E. coli* harboring a pGEX plasmid encoding GST-14-3-3β, GST-14-3-3ε, GST-14-3-3ε_K49E_, GST-14-3-3σ, or GST-14-3-3ζ and incubated overnight at 37˚C, shaking at 215 rpm. This initial culture was then spiked into 500-ml LB with ampicillin, and incubation was continued for 2 h. Expression of GST-14-3-3 fusions was induced by the addition of isopropyl β-d-1-thiogalactopyranoside (IPTG) to 1 mm, followed by an additional 4–5 h of incubation. Bacteria were pelleted and stored at -20˚C. Pellets were re-suspended in 10 ml Bacterial Lysis Buffer (BLB; 100 mm ethylenediaminetetraacetic acid, 1 mm PMSF, 10 μg/ml each leupeptin and pepstatin-A in PBS). Cells were sonicated in six 30 s intervals, intermitted with equal rest periods on ice, after which 1 ml of 10% Triton X-100 was added. Lysates were mixed separately and insoluble material was pelleted. Glutathione resin (400 μL of a 50% slurry in BLB) was added to the supernatant and rocked overnight at 4˚C. Beads were washed 2× in BLB, 3× in BCLB, and 2× in PBS and then stored in PBS (∼50% slurry) at 4˚C. For pulldown assays, cell lysates were rocked with 20 μL of the 50% slurry of individual GST-14-3-3-conjugated GSH beads at 4˚C. Beads were washed 3× with BCLB and proteins were eluted and denatured in 25 μL of sample buffer at 95˚C for 5 min before analysis via SDS-PAGE and Western blotting, as described above.

For far-Western blotting, GST-fusion proteins were eluted from the GSH resin in 50 mm Tris (pH 7–8), 100 mm NaCl, and 20 mm GSH for 30 min at 4˚C while rocking. Following centrifugation, the supernatant was removed and reserved. The eluent was dialyzed in 25 mm Tris and 50% glycerol in PBS overnight and stored at −20˚C. Eluted fusion proteins were diluted to a final concentration of approximately 50–100 ng/ml in 1.5% BSA in TBST with 0.005% sodium azide. Western blotting of α-FLAG or α-MYC immunoprecipitations was carried out as described above, with the additional incubation of blocked nitrocellulose membranes with diluted GST-fusion proteins before primary antibody incubation (α-GST). Densitometric analyses of α-GST and α-FLAG or α-MYC signals were conducted in Photoshop. Mean intensities in α-GST signals, normalized to α-FLAG/α-MYC signals, were compared in JMP using an all pairs, Tukey HSD test.

##### Immunoprecipitation and SDS-PAGE for Mass Spectrometry

Protein concentrations were determined using Bradford assays and an Eppendorf BioPhotometer Plus (Eppendorf; Hamburg, Germany) with BSA standards. For SILAC immunoprecipitations, normalized lysates (3.5 × 10^3^ μg total protein in 1 ml) from heavy/light conditions were incubated separately with α-FLAG Affinity Gel (10 μL of a 50% slurry) overnight, rocking at 4˚C. The beads were washed three times with BCLB, after which bound proteins were eluted and denatured in 30 μL sample buffer (150 mm Tris pH 6.8, 2% SDS, 5% β-mercaptoethanol, 7.8% glycerol, 0.25 ng/ml bromphenol blue) at 95˚C for 5 min. Immunoprecipitations were combined (10 μL each per well) separated on 10 and 15% acrylamide gels (30% w/v and 37.5:1 acrylamide:bis-acrylamide) with 4.2% acrylamide stacking gels. Current was maintained at 20 mA and 30 mA per gel through the stacking and separating layers, respectively. Following separation, proteins were stained with Coomassie. Immunoprecipitations from nonquantitative experiments for LC–MS/MS were carried out as described above and separated on 10% acrylamide gels (20 μL each per well).

To identify DCBLD2 interactors from zebrafish extracts, adult zebrafish were anesthetized with tricaine and then chilled in ice water before freezing. Frozen zebrafish were placed in BCLB and homogenized on ice using a tissue homogenizer (IKA Works Inc.; Wilmington, NC). Insoluble material was pelleted and the supernatant was used for immunoprecipitations using resins charged with DCBLD2: DCBLD2-charged resins were prepared by immunoprecipitating FLAG-tagged DCBLD2 constructs from HEK cells expressing DCBLD2 alone or DCBLD2 with ABL. The immune complexes were washed three times with BCLB and incubated with zebrafish extracts overnight. The complexes were washed again with BCLB three times before bound proteins were denatured at 95˚C and analyzed by SDS-PAGE, as described above. Animal care and use was according to an approved University of Vermont IACUC protocol by Alicia Ebert.

##### Peptide Preparation and LC–MS/MS Methods

Regions were excised from Coomassie-stained acrylamide gels, diced to 1-mm cubes, and transferred to separate microcentrifuge tubes. Gel pieces were washed with HPLC-grade H_2_O and then de-stained in 50 mm ammonium bicarbonate (NH_4_HCO_3_) and 50% acetonitrile (MeCN) at 37˚C for 30 min. De-stain was removed and gel pieces were dehydrated in 100% MeCN. For protein identification experiments, proteins were subjected to proteolytic digest with sequencing grade modified trypsin (10 ng/μL in 50 mm NH_4_HCO_3_) overnight at 37˚C. After centrifugation, supernatants were transferred to new microcentrifuge tubes. Remaining peptides were extracted from gel pieces with the addition of 50% MeCN, 2.5% formic acid (FA). Supernatants were combined with the initial tryptic digest supernatants, and gel pieces were dehydrated in 100% MeCN. The final extraction was combined with the previous two extractions and peptides were dried in a speed-vac. For mapping phosphorylation sites on DCBLD family members, gel bands containing DCBLD proteins were subjected to a short tryptic digest (4 h at 37˚C) and peptides were extracted as described above. Dried peptides were re-suspended in GluC (10 ng/μL in 50 mm NH_4_HCO_3_) and incubated for 2 h at 37˚C. The reaction was quenched in FA (2.5% in 50% MeCN), gel pieces were dehydrated as described above, and doubly digested peptides were dried in a speed-vac.

Dried peptides were re-suspended in Solvent A (2.5% MeCN, 0.15% formic acid (FA)) and separated via HPLC (300 nL/min) using the Easy n-LC 1200 before MS/MS analysis on the Q Exactive Plus mass spectrometer fitted with a Nanospray Flex ion source and supplied with Thermo Xcalibur 4.0 software (instruments and software from Thermo Fisher Scientific). Chromatography columns (15 cm × 100 μm) were packed in-house with 2.7 μm C18 packing material (Halo, pore size = 90 Å from Bruker, Billlerica, MA). Peptides were eluted using a 0–50% gradient of Solvent B (80% MeCN, 0.15% FA) over 60 min and ionized by nanospray ionization (2.2 kV). This gradient was followed by 10 min at 100% Solvent B before a 15-min equilibration in 100% Solvent A. The precursor scan (scan range = 360–1700 *m*/*z*, resolution = 7.0 × 10^4^, AGC = 10^6^, maximum IT = 100 ms, lock mass = 371.1012 *m/z*) was followed by ten higher energy C-trap dissociation (HCD) tandem mass spectra of the top ten ions in the precursor scan (resolution = 3.5 × 10^4^, AGC = 5.0 × 10^4^, maximum IT = 50 ms, isolation window = ±1.6 *m*/*z*, normalized collision energy = 26%, dynamic exclusion = 30 s).

##### Experimental Design and Statistical Rationale

SILAC immunoprecipitations were carried out with 293 cell extracts from two 10-cm dishes (7 mg of total protein) per experimental condition, which allowed us to obtain sufficient DCBLD1 or DCBLD2 protein for phosphorylation site analysis and to detect the known interactors of the CRK family as positive controls. Three biological replicates were conducted to apply statistical analyses to identified DCBLD1 and DCBLD2 interacting partners. To subtract nonspecific interactors, immunoprecipitations were conducted from 293 cells in the following heavy/light pairs for each biological replicate: mock transfections in both heavy and light, FYN expression (heavy) with mock transfection (light), and ABL transfection (heavy) with mock transfection (light). The heavy-to-light ratio of each DCBLD interactor was assessed for significance at 95% confidence using a student's *t* test with a Benjamini-Hochberg (BH) correction. This was deemed an appropriate statistical analysis given the normal distribution of background in the mock (heavy)/mock (light) condition.

##### Mass Spectrometry Data Filtering and Statistical Analysis

For PTM-mapping of DCBLD1 and DCBLD2 experiments, raw spectra were searched for matches within forward and reverse human DCBLD1 and DCBLD2 sequences using SEQUEST (version 28) with no enzyme indicated, 2 missed cleavages permitted, a precursor mass tolerance of ± 5 PPM and a fragment ion tolerance of ± 0.006 Da. The following differential modifications were permitted: phosphorylation of serine, threonine and tyrosine (+79.9663 Da), ubiquitylation of lysine (+114.0429 Da), oxidation of methionine (+15.9949 Da), carboxyamidomethylation of cysteine (+57.0215 Da), and acrylamidation of cysteine (+71.0371 Da). Peptides were then filtered to remove any cut sites other than after K, R, E, or D, as well as by precursor mass accuracy (tolerance = ±4 ppm) and cross-correlation (XCorr) scores dependent on charge state (XCorr^z=+1^ = 1.8; XCorr^z=+2^ = 2.0; XCorr^z=+3^ = 2.2; XCorr^z=+4^ = 2.4; XCorr^z=+5^ = 2.6). All identified DCBLD1 and DCBLD2 peptides from the double digest are tabulated in supplemental Table S1. Label free quantification (previously described in (9)) was achieved by taking ratios of phosphopeptide intensities across conditions, normalized to that of reference peptides within DCBLD1 (LGGQISVLQR, AAIHAGIIADE, DVAGDISGNMVDGYR, LQDQGPSWASGDSSNNHKPR) or DCBLD2 (FGDFDIEDSDSC^HFNYLR, ITGIITTGSTM*VEHNYYVSAYR, KPGPPWAAFATDE, LKKPGPPWAAFATDE), where C^ = Cys carboxyamidomethylation and M* = Met oxidation. The modified DCBLD2 peptides were chosen for use as loading controls because of their high abundance and comparable intensity changes across conditions to that of the unmodified loading controls. We note that these modifications are artifacts from the sample preparation, rather than post-translational, and therefore the same percentage of these sites should be modified on these reference peptides across conditions. Average precursor ion intensities of reference peptides and signal-to-noise ratios in each experimental condition are tabulated in Table I. Phosphorylation site localizations were scored using the ModScore algorithm (supplemental Table S1) (17).

For SILAC experiments, raw spectra from three biological replicates were searched for matches within a forward and reverse human proteome (Uniprot, 2018, >70,000 entries) using SEQUEST (version 28), requiring tryptic peptides and permitting 2 missed cleavages and the following differential modifications: heavy lysine (+8.0142 Da) and arginine (+10.0083 Da), phosphorylation of serine, threonine and tyrosine (+79.9663 Da), oxidation of methionine (+15.9949 Da), carboxyamidomethylation of cysteine (+57.0215 Da), and acrylamidation of cysteine (+71.0371 Da). Peptides were filtered by precursor mass accuracy (tolerance = ±4 ppm), XCorr scores (XCorr^z=+1^ = 1.8; XCorr^z=+2^ = 2.0; XCorr^z=+3^ = 2.2; XCorr^z=+4^ = 2.4; XCorr^z=+5^ = 2.6) and unique ΔCorr (≥0.15). These parameters resulted in a false discovery rate (FDR) < 1% calculated as follows: [# reverse hits]/([# forward hits] − [# reverse hits]). Proteins were considered identified by three or more peptides. Peptide heavy-to-light ratios (H/L) were calculated using Vista (18) by precursor maxima. Peptides were considered quantifiable if the signal-to-noise ratio (S/N) of either the heavy or light peptide was >10 (18). Quantified proteins were required to have three or more quantifiable peptides. Peptide H/L were averaged across a given protein to obtain a protein H/L with an associated standard deviation (S.D.).

For each experimental condition in a given set (supplemental Fig. S1), H/L of proteins present in both experimental and mock conditions were compared (experimental-to-mock fold change (E/M)). Those falling 2 standard deviations (SDs) from the mean E/M were retained in the experimental data set. All other proteins identified in the mock condition were removed from each experimental data set. Proteins that were identified bound to DCBLD1 or DCBLD2 in all three biological replicates in a given experimental condition were considered specific interactors. H/L were normalized to that of DCBLD(X) in a given treatment group before individual protein statistical comparisons at 95% confidence to the DCBLD(X) H/L ± S.D. using a student's *t* test with a Benjamini-Hochberg (BH) correction. For proteins identified by multiple isoforms, H/L were averaged across isoforms. H/L and corrected *p*-values of quantified specific interactors are tabulated in supplemental Table S4.

For the analysis of DCBLD2 interactors from zebrafish extracts, spectra were searched against a forward and reverse database of combined zebrafish and human proteomes (Uniprot). Peptides were filtered with the same parameters for mass tolerance, XCorr, and unique ΔCorr as described above. Peptides that mapped to zebrafish proteins were analyzed by the NCBI Protein BLAST tool (blast.ncbi.nlm.nih.gov (19)) to determine whether peptides were unique to zebrafish or common to human. DCBLD2 interactors identified in the zebrafish immunoprecipitation are tabulated in supplemental Tables S7 and S8.

##### Gene Ontology Term Enrichment Analysis

Gene Ontology (GO) term enrichment analyses of GO Molecular Function, GO Biological Process, and GO Cellular Compartment were conducted via the Metascape (metascape.org) platform (20). Metascape's default statistical parameters were used for the analysis. GO terms were considered enriched in the input data set if they possessed a corrected *p*-value of < 0.05.

## RESULTS

##### PTM Mapping of DCBLD1 and DCBLD2 Reveals Novel FYN- and ABL-Regulated Phosphorylation Sites

Previously, using a targeted LC–MS/MS approach, we found that the nonreceptor tyrosine kinases FYN and ABL regulated several tyrosine phosphorylation sites on DCBLD family members (human DCBLD2 and mouse DCBLD1) (9). Given that we found the tyrosine residues in DCBLD intracellular YXXP motifs critical to the DCBLD/CRKL-SH2 interaction (10), our targeted approach in that study focused primarily on the phosphorylation of YXXP tyrosines. Although a few YXXP tyrosines remained refractory to quantification because of their presence in long tryptic peptides housing multiple potential phosphorylation sites, several were quantitatively monitored. We found that both FYN and ABL induced DCBLD2 tyrosine phosphorylation in YXXP motifs. Although both cytoplasmic kinases induced the phosphorylation of common sites, FYN and ABL exhibited distinct specificity for certain DCBLD2 YXXP tyrosines (9). However, ABL, but not FYN, induced YXXP tyrosine phosphorylation of DCBLD1 (9). Therefore, we conducted a quantitative proteomics analysis of DCBLD receptor binding proteins in the presence or absence of either FYN or ABL for DCBLD2 and in the presence or absence of only ABL for DCBLD1. This approach also afforded us the opportunity to examine in a more unbiased fashion the phosphorylation of serine, threonine and tyrosine residues outside of YXXP motifs, as well as to consider alternate approaches to analyze the previously unidentifiable or unquantifiable pYXXP-containing peptides in DCBLD proteins. As these analyses go hand-in-hand, and as differential phosphorylation analyses may reveal mechanisms behind changing interactomes, we first describe an in-depth phosphorylation analysis of DCBLD proteins, followed by an analysis of their interactomes dependent on ABL and also on FYN for DCBLD2.

To increase coverage and thereby identify phosphorylation sites not readily observed in tryptic peptides, we took a more comprehensive approach to map phosphorylation sites on DCBLD1 (human) and DCBLD2 (human) using a limiting, double-enzyme digest before LC–MS/MS analysis. DCBLD1 and DCBLD2 were transiently expressed in 293 cells with and without FYN (DCBLD2 only) or ABL (both family members). DCBLD proteins were immunoprecipitated (α-FLAG) and subjected to SDS-PAGE and Coomassie staining. Bands containing DCBLD1 or DCBLD2 were excised and subjected to a double-enzymatic digest with trypsin, followed by GluC. Peptides were analyzed by LC–MS/MS and spectra were searched via SEQUEST against forward and reverse human DCBLD1 or DCBLD2 sequences. No enzyme was specified to expand the database of potential hits and the resulting data were filtered to include only trypsin and/or GluC cut sites, resulting in a FDR of <1%.

**Fig. 1:**
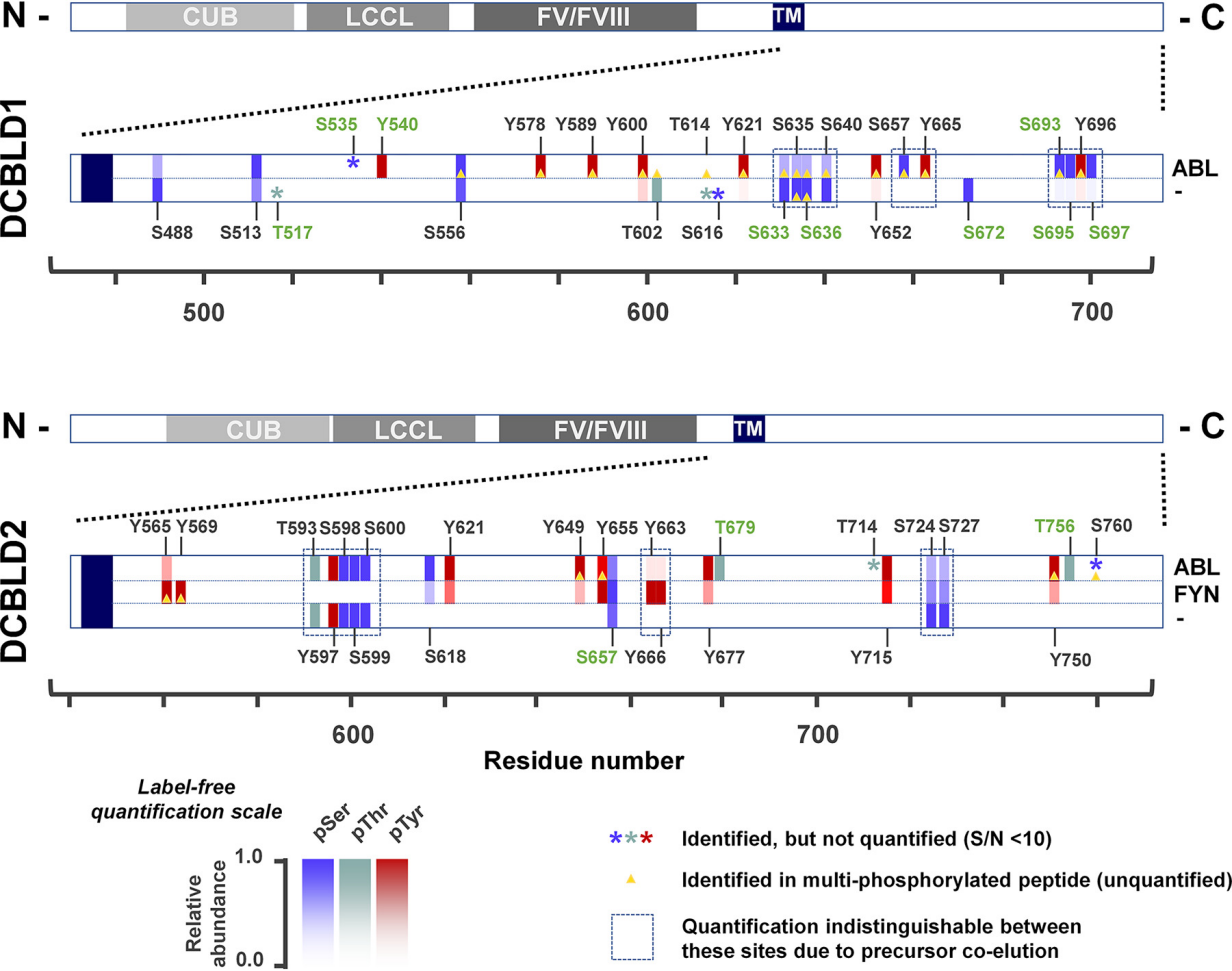
**Differential phosphorylation of DCBLD family members by FYN and ABL.** DCBLD1-FLAG and DCBLD2-FLAG were transiently expressed in 293 cells in the presence and absence of ABL or FYN. Immunoprecipitates (α-FLAG) from 293 lysates were analyzed by SDS-PAGE and Coomassie staining. Bands containing DCBLD1 and DCBLD2 were excised and subjected to proteolytic digest with Trypsin and GluC before analysis via LC–MS/MS. RAW spectra were searched against DCBLD1 or DCBLD2 sequences. Intensities of quantifiable singly phosphorylated peptides (S/N <10) were normalized to unmodified loading control peptides for comparison of relative phosphopeptide intensities across conditions. Relative phosphopeptides abundance is plotted as a spatially resolved heat map across DCBLD1 and DCBLD2 sequences, with blue, green, and red gradients displaying phosphoserine (pSer), phosphothreonine (pThr), and phosphotyrosine (pTyr) abundances, respectively. Unquantifiable phosphorylation sites are indicated by asterisks (*) of the same colors. Sites identified in multiply phosphorylated peptides in each condition are indicated with yellow triangles and tabulated in supplemental Table S1 (supplemental Spectra), but not quantified. In cases where peptides containing multiple potential phosphorylation sites co-eluted and distinct fragment ions demonstrating phosphorylation site localization were not identified, proximal sites were quantified together, indicated by dashed boxes. Notated residues of phosphorylation sites identified in this study that have not yet been curated in the PhosphoSitePlus (phosphosite.org) database are highlighted in green.

Several serine, threonine and tyrosine phosphopeptides were identified on both DCBLD1 and DCBLD2. Singly phosphorylated peptides were quantified by relative ion intensity across experimental conditions, normalized to loading control peptides within the DCBLD1 or DCBLD2 sequence. Quantification of individual serine (blue), threonine (green) and tyrosine (red) phosphorylation sites are represented in a spatially resolved heat map along DCBLD protein sequences in Fig. 1, and in tabulated form in Table II. Singly phosphorylated peptides possessing multiple potential S/T/Y phosphorylation sites that were indistinguishable by fragment ions were quantified together and surrounded by boxes in Fig. 1 (Table II). Although the specific phosphorylation sites remained ambiguous, it was still important to show regions where phosphorylation increased or decreased, as these may represent important regulatory regions on DCBLD proteins. However, we note that additional targeted studies would be required to resolve those sites. Unquantifiable sites (S/N < 10) and sites identified in multiply phosphorylated peptides in each experimental condition are indicated by asterisks and yellow triangles, respectively (Fig. 1). All DCBLD1 and DCBLD2 peptides identified, with ModScore values for phosphopeptides (17), are tabulated in supplemental Table S1. Annotated spectra of confirmed phosphorylation sites are included in the supplemental material.

**Table I:** Reference peptide intensity and signal-to-noise (S/N) for DCBLD1 and DCBLD2 label free quantification

z	Protein	Peptide	DCBLD(X) alone		DCBLD(X) + ABL		DCBLD(X) + FYN	
			Intensity	S/N	Intensity	S/N	Intensity	S/N
2	DCBLD1	E.LGGQISVLQR.K	5.01E + 08	16587.2	1.81E + 08	8321.4	–	–
2	DCBLD1	K.AAIHAGIIADE.L	1.35E + 08	944.8	6.97E + 07	1586.6	–	–
2	DCBLD1	R.DVAGDISGNMVDGYR.D	9.17E + 07	51.2	3.77E + 07	38.6	–	–
3	DCBLD1	R.LQDQGPSWASGDSSNNHKPR.E	4.25E + 08	640.8	1.25E + 08	320	–	–
3	DCBLD2	K.FGDFDIEDSDSC^HFNYLR.I	2.15E + 07	451.9	3.24E + 07	490.8	5.75E + 06	113.4
3	DCBLD2	K.ITGIITTGSTM*VEHNYYVSAYR.I	1.37E + 06	197.9	3.04E + 06	218.4	2.66E + 06	72.9
2	DCBLD2	K.KPGPPWAAFATDE.Y	6.16E + 07	1183.5	1.02E + 08	1991.5	2.70E + 07	607.8
3	DCBLD2	R.LKKPGPPWAAFATDE.Y	3.43E + 07	1011.2	7.02E + 07	766	1.45E + 07	75.4


Average reference peptide intensities were used for normalization purposes to compare phosphopeptide intensity across experimental conditions. ^ = Cys carbamidomethylation, * = Met oxidation.

**Fig. 2:**
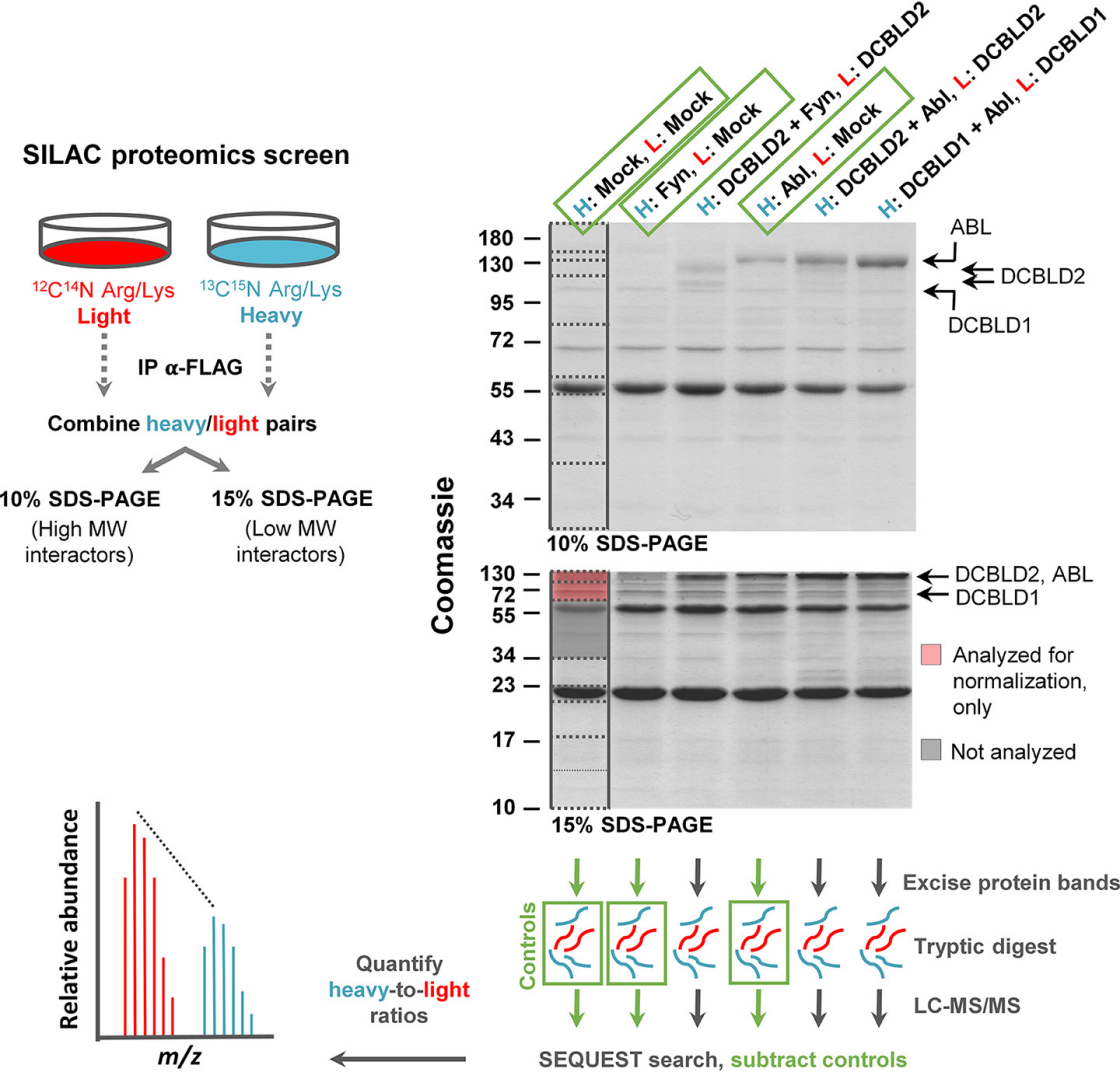
**Schematic illustrating SILAC experimental design for the identification of DCBLD1 and DCBLD2 interactors.** DCBLD1 and DCBLD2 were transiently expressed with and without FYN and/or ABL in 293 cells grown in DMEM supplemented with arginine and lysine containing either heavy (^13^C, ^15^N) or light (^12^C, ^14^N) stable isotopes of carbon and nitrogen. DCBLD(X)-FLAG constructs were immunoprecipitated from 293 lysates and heavy and light experimental pairs were combined before SDS-PAGE and Coomassie staining. Immunoprecipitates were split across 10 and 15% acrylamide gels to maximize separation of high molecular weight proteins (10% SDS-PAGE) while retaining smaller proteins (15% SDS-PAGE) in the analysis. Each lane was divided into the indicated regions of molecular weight for analysis. All regions of 10% gels were analyzed for binding partners, whereas the 15% gels were only analyzed below 34 kDa to minimize overlap in each data set. The region containing DCBLD family members in 15% gels were also analyzed for normalization purposes. Each region was subjected to tryptic digestion and analyzed via LC–MS/MS. Raw spectra were searched against a forward and reverse human protein database (Uniprot 2011) with the SEQUEST algorithm. Proteins identified by three or more peptides within ± 4 ppm of theoretical precursor masses and unique delta-correlation scores of 0.15 or higher were considered identified. Proteins identified in control heavy/light conditions (heavy = Mock, light = Mock; heavy = FYN, light = Mock; heavy = ABL, light = Mock), denoted by green boxes, were removed from experimental datasets. Quantifiable peptides possessed summed heavy/light signal-to-noise ratios (S/N) of >20. This workflow was carried out for three separate experimental replicates.

**Table II:** Average ion intensities DCBLD1 and DCBLD2 phosphopeptides. Average ion intensities of all quantifiable singly phosphorylated peptides identified in label-free quantification studies for A, DCBLD1 and B, DCBLD2 are tabulated. Intensities of quantifiable singly phosphorylated peptides (S/N >10) were normalized to unmodified loading control peptides (Table I) for comparison of relative phosphopeptide intensities across conditions. Phosphorylated species that were not resolve chromatographically were quantified together, indicated by multiple site localizations with one average intensity. These intensities were used to quantify relative changes in phosphopeptide intensities represented in the heat map in Fig. 1

A		
Site(s)	Average normalized intensity	
	DCBLD1	DCBLD1 + ABL
S488	4.14E + 07	9.85E + 06
S513	6.52E + 07	1.29E + 08
Y540	–	8.16E + 07
S556	2.03E + 08	2.33E + 08
Y578	–	3.22E + 08
Y589	–	9.91E + 07
Y600	3.65E + 07	7.12E + 08
T602	2.04E + 07	–
Y621	9.27E + 06	9.12E + 08
S633, S635, S636, S640	8.83E + 08	1.90E + 08
Y652	4.57E + 06	2.28E + 08
S657, Y665	–	5.04E + 07
S672	2.16E + 06	–
S963, S695, Y696, S697	3.89E + 06	9.39E + 08

B			
Site(s)	Average Normalized Intensity		
	DCBLD2	DCBLD2 + ABL	DCBLD2 + FYN
Y565	–	1.09E + 07	8.35E + 07
Y569	–	–	4.38E + 07
T593, Y597, S598, S599, S600	8.42E + 07	8.71E + 07	–
S618	–	5.36E + 07	1.16E + 07
Y621	–	1.45E + 08	4.71E + 07
Y649	–	1.21E + 08	1.39E + 07
Y655	–	3.89E + 06	5.21E + 06
S657	4.35E + 06	3.73E + 06	5.96E + 06
Y663, Y666	–	1.17E + 06	5.22E + 07
Y677	–	1.58E + 07	3.40E + 06
T679	–	2.00E + 06	–
Y715	–	8.91E + 07	5.29E + 07
S724, S727	9.58E + 07	1.16E + 07	4.70E + 07
Y750	–	6.14E + 07	2.02E + 07
T756, S760	–	7.63E + 05	–

Quantification of FYN- and ABL-driven phosphorylation of DCBLD1 Y589, Y600, and Y621 and DCBLD2 Y565, Y621, Y715 (nonYXXP), and Y750 corroborated previous reports (9, 10). Additional tyrosine resides on both DCBLD1 and DCBLD2 sequences were further characterized as FYN- and/or ABL- driven phosphorylation sites, including DCBLD1 YXXP tyrosines Y540, Y578, Y652, Y665 and Y696, DCBLD2 YXXP tyrosines Y655, Y666 and Y677, and DCBLD2 tyrosines outside CRK/CRKL-SH2 binding motifs Y569, Y597, Y649 and Y663. One DCBLD2 YXXP tyrosine residue, Y732, remained undetected. Interestingly, several serine and threonine phosphorylation sites were found to be differentially regulated by FYN or ABL expression (Fig. 1). A comparison with phosphorylation sites curated on PhosphoSitePlus (phosphosite.org (21)) confirmed that several novel phosphorylation sites on DCBLD1 and DCBLD2 were identified in this study, highlighted with green site locations (Fig. 1).

##### FYN- and ABL-Regulated DCBLD Interactome Defined by Quantitative LC–MS/MS

With the identification of additional FYN- and ABL-regulated tyrosine, serine, and threonine phosphorylation sites on DCBLD1 and DCBLD2, our hypothesis was strengthened that ABL- and FYN-regulated phosphorylation of DCBLD proteins could alter their binding partners. Therefore, we took a quantitative proteomics approach using stable isotope labeling of amino acids in cell culture (SILAC) to identify DCBLD interactors in the presence and absence of active FYN and/or ABL.

**Fig. 3:**
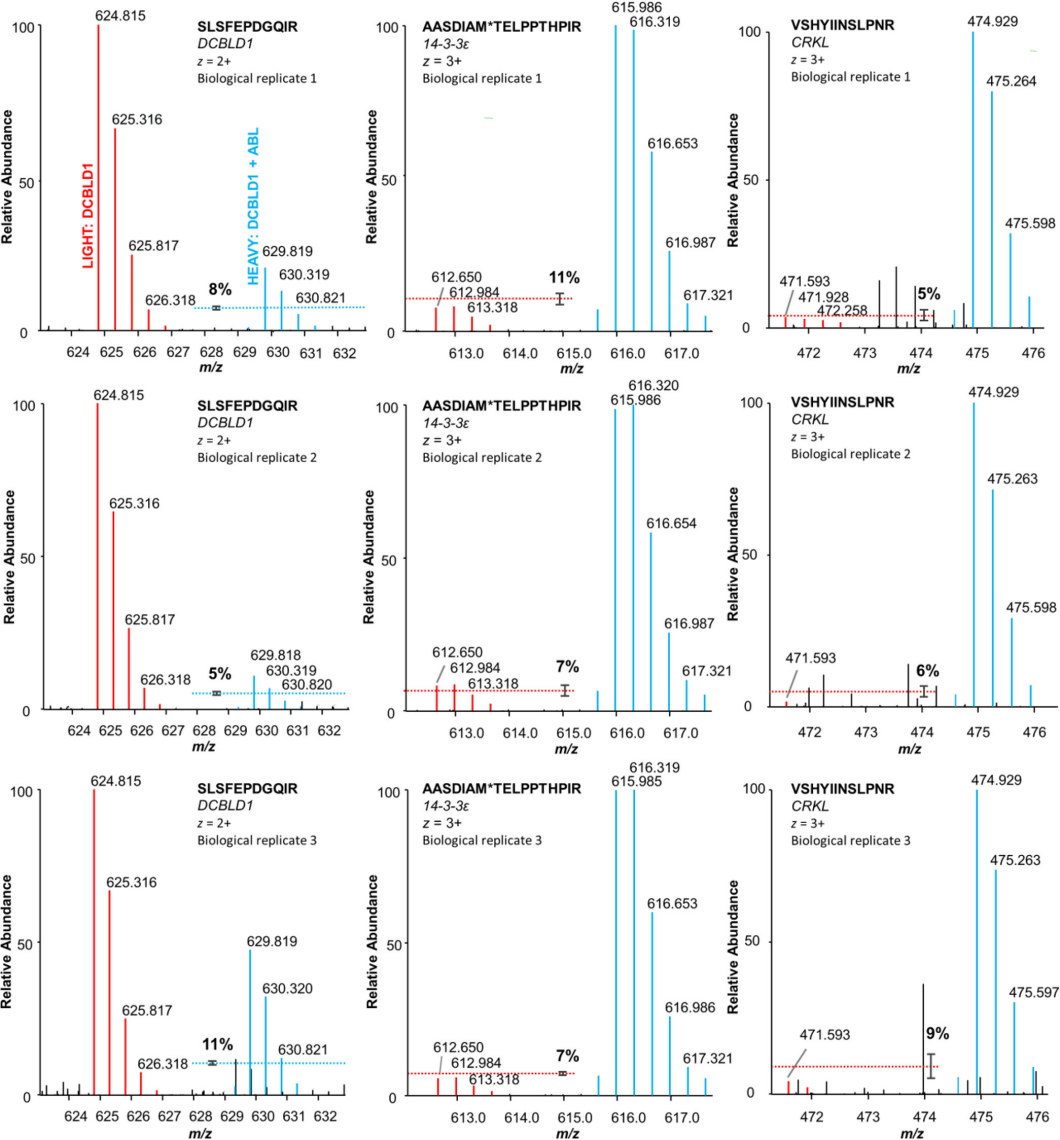
**MS1 spectra of select DCBLD1, 14-3-3ε, and CRKL peptides identified in the DCBLD1 α-FLAG immune complex in each biological replicate.** Light peptides (*red*) originated from 293 cells transiently expressing DCBLD1 alone, and heavy peptides (*blue*) originated from cells expressing ABL alongside DCBLD1. Dashed lines with error bars (standard error) indicate the average percent of the less abundant peptide in each heavy and light pair, relative to the base peak, across all peptides for the given protein. Although DCBLD1 protein levels decrease by an average of 10% with ABL co-expression, 14-3-3ε and CRKL proteins increase >10% in the DCBLD1 immune complex in the presence of ABL.

Cells were grown in medium containing lysine and arginine with only heavy or light stable isotopes of carbon (^13^C or ^12^C) and nitrogen (^15^N or ^14^N) for at least 1 week before transfection. The constructs were transiently expressed in labeled or unlabeled 293 cells for the following experimental SILAC pairs: DCBLD1 (light) and DCBLD1 + ABL (heavy), DCBLD2 (light) and DCBLD2 + ABL (heavy), DCBLD2 (light) and DCBLD2 + FYN (heavy). DCBLD family members were immunoprecipitated (α-FLAG) from cell lysates and SILAC pairs were combined before SDS-PAGE analysis and Coomassie staining (Fig. 2, supplemental Fig. S1). Immunoprecipitations from control SILAC pairs were carried out to identify nonspecific proteins bound to immune complexes: Mock (light) and Mock (heavy), Mock (light) and ABL (heavy), Mock (light) and FYN (heavy). To effectively separate and identify high and low molecular weight interactors, combined SILAC pairs were divided across 10 and 15% SDS-PAGE gels (Fig. 2, supplemental Fig. S1). Stained 10 and 15% gels were divided into 8 and 7 regions of molecular weight, respectively, all of which were individually digested into tryptic peptides and analyzed by LC–MS/MS (Fig. 2). Proteins identified in control conditions were removed from the data set unless significantly enriched (95% CL) in an experimental heavy or light condition. All heavy-to-light ratios (H/Ls) were normalized to those of DCBLD family members before statistical analysis across three experimental replicates. Notably, we observed a substantial loss of DCBLD1 protein when co-expressed with ABL. Fig. 3 shows unnormalized MS^1^ spectra of representative DCBLD1 peptides, demonstrating a 10-fold decrease of DCBLD1 levels on average in when ABL was co-expressed.

**Fig. 4:**
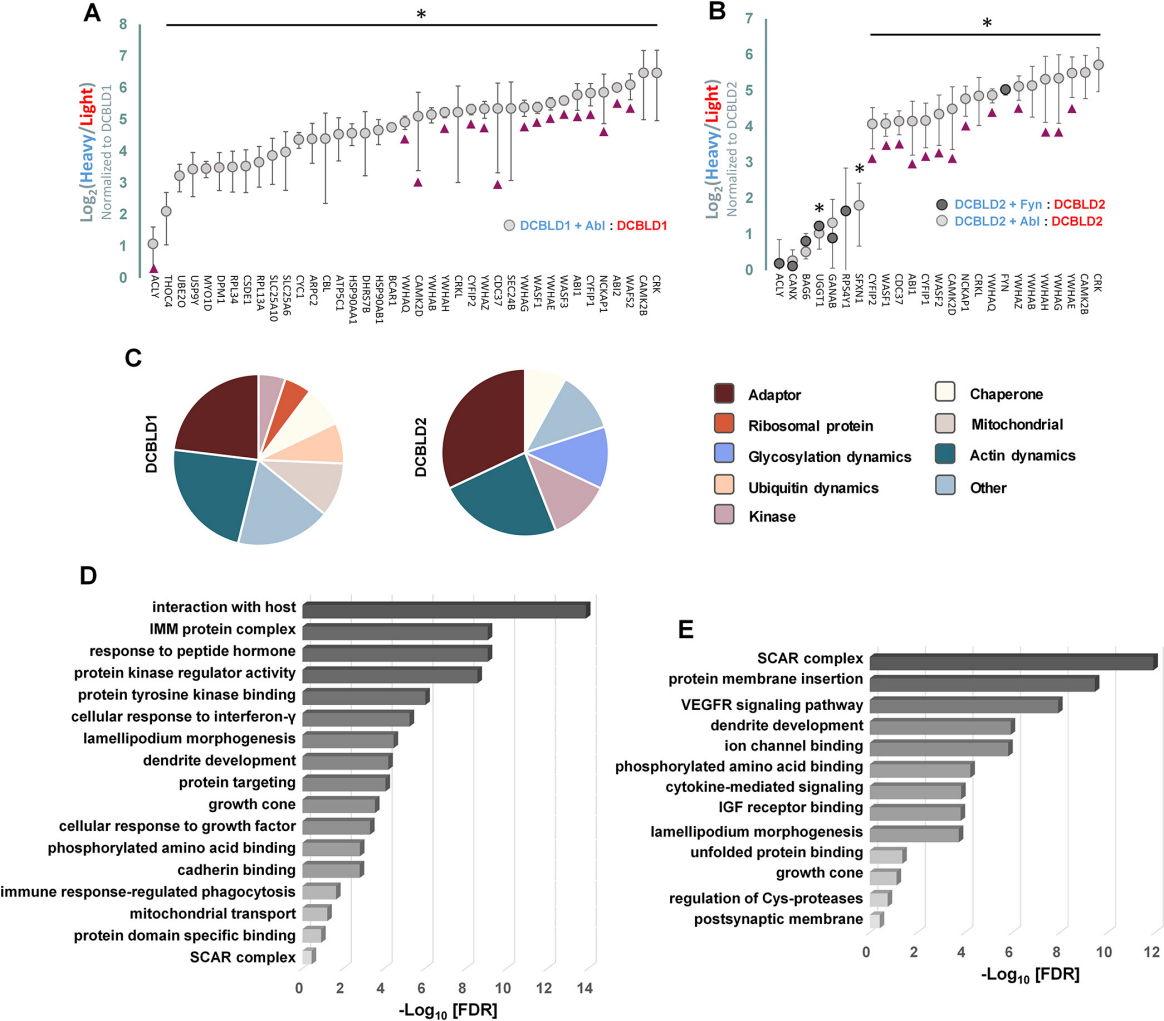
**FYN- and ABL-driven DCBLD1 and DCBLD2 interactors identified through a SILAC proteomics screen.***A*, DCBLD1 and *B*, DCBLD2 interacting proteins identified in the SILAC LC–MS/MS analysis described in Fig. 2.are plotted by their Log_2_-tranformed heavy-to-light ratios (H/L) in rank order. H/L of interactors were normalized to that of DCBLD1 or DCBLD2. Error bars represent the standard deviation of the mean H/L across three experimental replicates. Interactors exhibiting significantly increased (95% CL) binding to DCBLD proteins in the presence of FYN or ABL are denoted with an asterisk. Triangles denote potential DCBLD interactors, given that they were also detected in immune complex with ABL. Full peptide tables from each biological replicate are included in supplemental Table S4. *C*, General functional classifications of DCBLD1 and DCBLD2 interactors are outlined in pie charts. Each interactor is represented once. *D* and *E*, Gene Ontology (GO) term enrichment analysis of proteins identified in immune complex with (*D*) DCBLD1 and (*E*) DCBLD2. Uniprot accessions of DCBLD1 and DCBLD2 interactors identified in the SILAC proteomics screen were uploaded to the Metascape (metascape.org) platform. A GO term enrichment analysis of GO Molecular Function, GO Biological Process, and GO Cellular Compartment was conducted using the default statistical settings. Proteins with corrected *p*-values less than 0.05 were considered enriched in the data set. Negative Log_10_-transformed corrected *p*-values of enriched terms are plotted. Full lists of proteins annotated with enriched terms are included in supplemental Tables S5 and S6.

DCBLD1- and DCBLD2-specific interactors common across replicates are plotted by their Log_2_-transformed average H/L in Fig. 4*A*–4*B*. Interestingly, no interactors were found to dissociate from either DCBLD family member in the presence of FYN or ABL (Fig. 4*A*–4*B*). Those with significantly different (95% CL) H/Ls are marked with asterisks (Fig. 4*A*–4*B*). No proteins were induced to bind to the α-FLAG resin when FYN was expressed alone. ABL interactors identified in the ABL-alone controls are indicated (triangles in Fig. 4*A*–4*B*). Although these proteins were found to interact with ABL, they were maintained as potential DCBLD2 interactors given their potential to interact with DCBLD2 via ABL, or uniquely with DCBLD2. ABL is known to bind DCBLD2 through its SH2 domain (9), which could represent a mechanism by which additional DCBLD2-interacting proteins could be transported to DCBLD2 phosphotyrosine docking sites for ABL-SH2.

The pie charts in Fig. 4*C* indicate the proportion of DCBLD1 and DCBLD2 interactors, separately, with the indicated functional classifications, as presently curated in the major bioinformatic repositories. Each interactor is represented once. Strikingly, several adaptor proteins with phosphorylation-dependent binding domains, including members of the CRK and 14-3-3 protein families, were among the ABL-induced interactors of both DCBLD1 and DCBLD2, as well as several proteins related to actin dynamics (Fig. 4*A*–4*C*). Proteins involved in the regulation of ubiquitin modification, including the E3 ubiquitin ligase c-Cbl, the E3-independent E2 ubiquitin-conjugating enzyme (UBE2O), and the C-terminal ubiquitin hydrolase FAF-Y (USP9Y), were induced to bind to DCBLD1 by ABL (Fig. 4*A*, 4*C*). Among the DCBLD2 interacting partners identified in both FYN and ABL SILAC pairs were three proteins involved in glycosylation dynamics, calnexin (CANX), UDP-glucose:glycoprotein glucosyltransferase 1 (UGGT1), and neutral α-glucosidase (GANAB), although none exhibited marked changes in binding to DCBLD2 in the presence of either kinase (Fig. 4*B*–4*C*).

Several cytoplasmic kinases were also found in complex with DCBLD1 and DCBLD2 (Fig. 4*C*). Serine/threonine kinases of the Ca^2+^/calmodulin-dependent protein kinase type-II family CaMK-IIβ and CaMK-IIδ were induced to bind both DCBLD family members in the presence of ABL (Fig. 4*A*–4*B*). In accordance with our previous work that characterized the interaction between DCBLD2 and the FYN-SH2 domain (10), FYN co-immunoprecipitated with DCBLD2 when the two proteins were co-expressed (Fig. 4*B*).

Gene ontology (GO) term enrichment analyses of DCBLD1- and DCBLD2-interacting proteins are summarized in Fig. 4*D*–4*E*, revealing the enrichment of terms related to several signaling mechanisms and biological processes known to involve DCBLD family members. Full results of GO term enrichment analysis are included in supplemental Tables S5 and S6. Several GO terms related to cell motility were enriched among DCBLD1 and DCBLD2 interacting proteins, including GO:0031209 *SCAR complex* (LogFDR = −11.919 (DCBLD2), −13.886 (DCBLD1)), GO:0016358 *dendrite development* (LogFDR = −5.923 (DCBLD2), −3.552 (DCBLD1)), GO:0072673 *lamellopodium morphogenesis* (LogFDR = −3.740 (DCBLD2), −3.310 (DCBLD1)), and GO:0030426 *growth cone* (LogFDR = −4.206 (DCBLD1)). Also enriched in the DCBLD interactor datasets were GO terms related to receptor tyrosine kinase signaling, including general terms in the DCBLD1 data set (GO:0071363 *cellular response to growth factor stimulus* (LogFDR = −4.465) and GO:1990782 *protein tyrosine kinase binding* (LogFDR = −2.806)), and more specific terms related to VEGFR and IGFR signaling in the DCBLD2 datasets (GO:0048010 *vascular endothelial growth factor receptor signaling* (LogFDR = −7.916) and GO:0005159 *insulin-like growth factor receptor binding* (LogFDR = -3.818 (DCBLD2)). The association of these newly identified DCBLD interactors with biological processes related to changes in cell morphology and motility, as well as receptor tyrosine kinase (RTK) signaling, has been supported by reports in the literature concerning the implications of DCBLD family members in cell proliferation and migration downstream of RTK activation (1, 2, 3, 4, 5, 6, 7, 8).

Complimentary to the phosphorylation site map constructed from the double enzyme digest and label free quantification described above, heavy-to-light ratios of DCBLD1 and DCBLD2 post-translational modifications identified in the SILAC datasets, along with means and standard deviations across replicates where applicable, are tabulated in supplemental Table S3. Additional sites identified here include DCBLD1 T614, S619, and S683, as well as DCBLD2 S606 and S720. Phosphorylation of DCBLD1 S619 and DCBLD2 S720 was strongly induced by ABL (supplemental Table S3).

We then asked whether any of the DCBLD2 interacting partners identified in 293 cells were conserved across vertebrate species, and therefore likely serving important functional roles in vertebrate biology. Several investigations involving the modulation of DCBLD2 expression have been conducted in the model vertebrate systems of mouse and zebrafish. DCBLD2 is known to be up-regulated in the neointima of healing vasculature in mice. Further, vascular development and repair are impaired when DCBLD2 is knocked down in zebrafish and knocked out in mice (3). DCBLD2 is also involved in insulin signaling in mice; DCBLD2^−/−^ mice present improved insulin sensitivity and glucose uptake (6). We chose zebrafish as a system of study, given their evolutionary distance from humans in general vertebrate phylogeny, such that DCBLD2 interactions common to zebrafish and humans would represent those with strong evolutionary conservation.

To identify zebrafish proteins that would interact with human DCBLD2, DCBLD2 was first immunoprecipitated (α-FLAG) from 293 cells transiently expressing the protein in the presence or absence of ABL. ABL was chosen given its robustness in inducing DCBLD2 interactions in the SILAC screen (Fig. 4*A*–4*B*). Immunoprecipitations were then incubated with zebrafish extracts before SDS-PAGE, tryptic digest, and analysis via LC–MS/MS. Spectra were searched against a combined human and zebrafish proteome, and unique zebrafish peptides were extracted to distinguish zebrafish interactors from human (supplemental Tables S7 and S8). ABL-induced zebrafish interactors of DCBLD2 were extracted and compared with ABL-induced DCBLD1 and DCBLD2 human interactors identified in the SILAC screen (supplemental Fig. S2). Eight zebrafish homologs of the human interactors identified in the SILAC screen were detected in immune complex with DCBLD2 in the presence of ABL, including five members of the 14-3-3 adaptor family, CaMKIIδ, and the known DCBLD interactors CRK and CRKL (supplemental Fig. S2). Although these are not endogenous complexes that were extracted from fish, the interactions identified in both 293 cells and fish are likely relevant and signify evolutionarily conserved interactions.

##### ABL Regulates the Binding of 14-3-3 Adaptor Family to DCBLD1 and DCBLD2

In both SILAC and zebrafish co-immunoprecipitation screens described above, several members of the 14-3-3 family of adaptor proteins emerged as evolutionarily conserved ABL-induced DCBLD2 interactors (Fig. 4*A*–4*B*, supplemental Fig. S2). Members of the 14-3-3 adaptor are highly conserved across eukaryotes and canonically bind phosphorylated serine (pS) and phosphorylated threonine (pT) residues in RXXpS/pTXP (mode 1), RXXXpS/pTXP (mode 2), or C-terminal pS/pTX(X)-CO_2_^−^ (mode 3) motifs (15, 22). However, 14-3-3 proteins are also known to bind pS/pT residues outside the canonical motifs and are capable of binding certain target proteins independent of phosphorylation (15, 23). Central to signal transduction, these homo- and heterodimeric proteins are involved in cell cycle regulation, apoptosis, cell growth, differentiation, vesicular trafficking, and can regulate the subcellular localization, activity, or interactors of their target molecules. The identification of several members of the 14-3-3 family of adaptor proteins in immune complex with DCBLD1 and DCBLD2 was intriguing, in part because their heavy-to-light ratios suggested these canonically phosphoserine/phosphothreonine-dependent interactors were induced to bind DCBLD proteins in the presence of the tyrosine kinase ABL.

To further characterize this interaction, we first purified fusion proteins of GSH S-transferase (GST) with select members of the 14-3-3 family (14-3-3β, 14-3-3σ, 14-3-3ε, 14-3-3ζ) for use in pulldown assays and Western blotting. Members β, ε, and ζ were first chosen for biochemical validation as representative 14-3-3 members from the subset identified in the proteomics screen. Although unique peptides were identified from these three 14-3-3 proteins, several peptides that the SEQUEST algorithm mapped to select 14-3-3 proteins were common among multiple or all family members. As there were multiple redundancies of 14-3-3 peptides in human database used as input for SEQUEST, the H/Ls of common 14-3-3 peptides were manually quantified separately from peptides that were unique to 14-3-3 family members identified by SEQUEST. However, the H/Ls were not found to change considerably when quantified separately. The occurrence of multiple redundant peptides raised the possibility that additional 14-3-3 family members that were not included in the data set obtained from the SEQUEST analysis could also be interacting with DCBLD family members because they were not represented by unique peptides. Therefore, we chose to test an additional family member that was not represented by unique peptides, 14-3-3σ, for its potential to bind to DCBLD2. Given the strong loss of DCBLD1 protein levels when it was co-expressed with ABL in the SILAC experiments, we did not attempt to further investigate this interaction. However, this will be an area of further investigation once an expression system that can prevent DCBLD1 degradation is established.

**Fig. 5:**
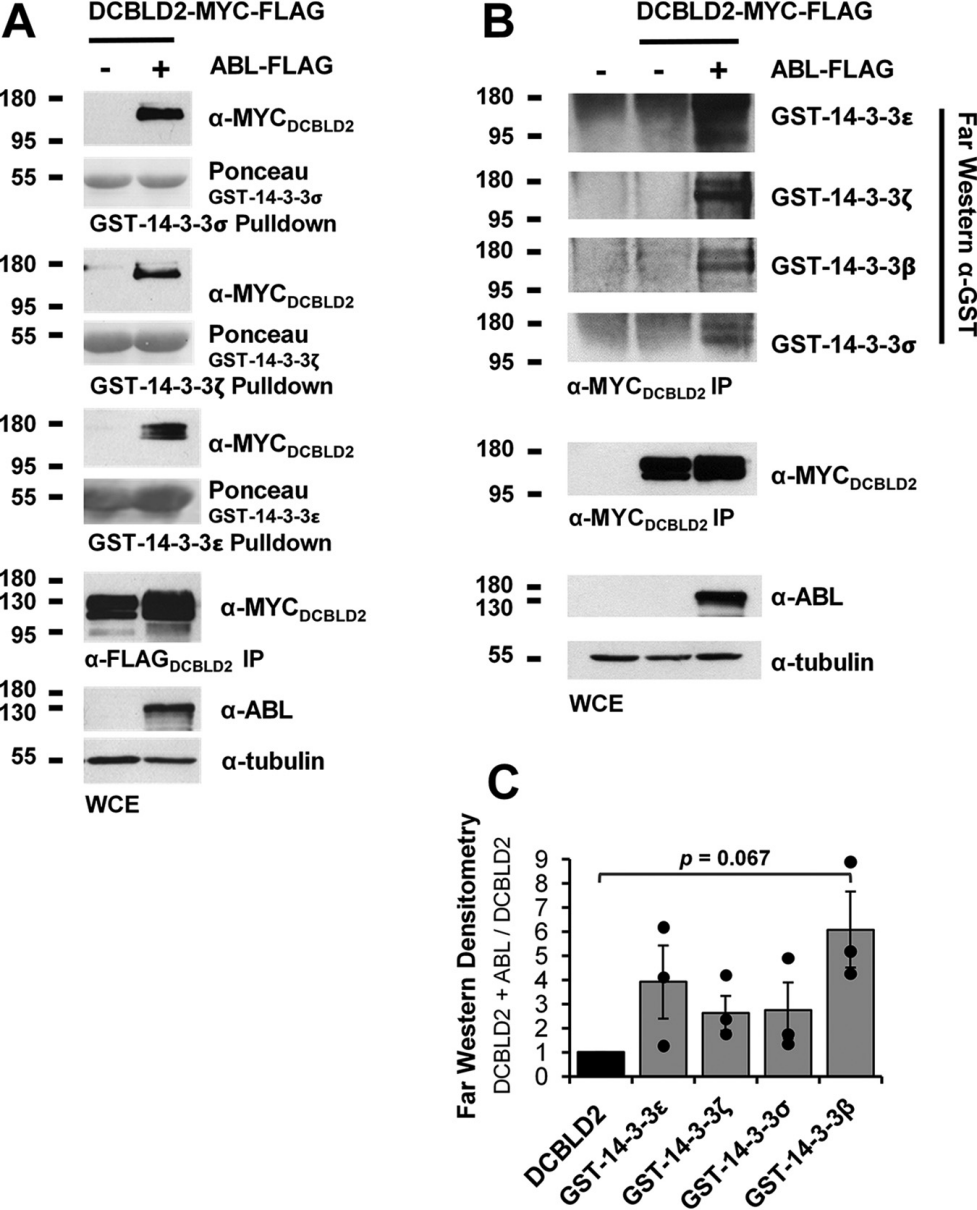
**14-3-3 family members bind to DCBLD2 in an ABL-induced manner.***A*, DCBLD2-FLAG-MYC was transiently expressed in 293 cells in the presence and absence of ABL. GST-fusions with 14-3-3σ, 14-3-3ε, and 14-3-3ζ on GSH resin were incubated with 293 extracts in pulldown assays. Bound proteins were denatured and analyzed by SDS-PAGE and Western blotting. Ponceau stains display levels of GST-14-3-3(X) and α-MYC blots of the pulldowns conditions in which DCBLD2 bind to the fusion protein. Immunoprecipitations (IP, α-FLAG) show DCBLD2 expression levels. Whole cell extracts (WCEs) show levels of ABL expression (α-ABL) and the tubulin blot serves as a loading control. *B*, DCBLD2-FLAG-MYC was transiently expressed in 293 cells in the presence and absence of ABL. Immunoprecipitations (α-MYC) from 293 cell lysates were subject to SDS-PAGE and far-Western blotting with eluted GST-14-3-3 fusions proteins, followed by α-GST primary antibodies. Signals in α-GST blots show relative levels of GST-fusions interacting with DCBLD2 in the presence and absence of ABL, and the α-MYC blot displays levels of DCBLD2 protein. *C*, α-GST signals from (*B*) were quantified across three replicates and normalized to DCBLD2 levels using densitometry. Fold changes of GST-14-3-3 binding in the presence of ABL are plotted. Error bars are representative of three trials.

To confirm that the purified GST-14-3-3 proteins were functional, resin-bound fusion proteins were incubated with 293 cell extracts treated with and without calyculin A, a serine/threonine phosphatase inhibitor. Proteins that bound to the GST-14-3-3 resin were denatured and subjected to SDS-PAGE and Western blotting for the canonical (mode-1) 14-3-3 binding motif, RXXpS/pTXP. Pulldowns from these same extracts with GST alone as well as a GST-14-3-3ε mutant incapable of phospho-dependent binding (GST-14-3-3_K49E_) served as negative controls. All WT 14-3-3 fusion proteins exhibited increased binding to RXXpS/pTXP-containing proteins in extracts from cells treated with calyculin A before lysis, whereas the GST and GST-14-3-3_K49E_ proteins did not (supplemental Fig. S3). Wild-type fusion proteins were then incubated with extracts from 293 cells transiently expressing DCBLD2 with C-terminal FLAG and MYC tags alone or alongside ABL-FLAG. Pulldowns were subjected to SDS-PAGE and Western blotting for DCBLD2 (α-MYC; Fig. 5*A*). The MYC tag was used because ABL was found to also bind to GST-14-3-3 fusion proteins (supplemental Fig. S4). In agreement with SILAC ratios obtained from the proteomics screen, DCBLD2 was induced to bind to all four GST-14-3-3 fusion proteins when ABL was co-expressed (Fig. 5*A*).

Several potential 14-3-3 docking sites (RXXS motifs) are housed within the DCBLD1 and DCBLD2 intracellular sequences. Several of these sites were found phosphorylated in the label-free phosphorylation site mapping and in the SILAC screen (Fig. 1, supplemental Table S3). Because of the loss of DCBLD1 protein on co-expression with ABL, the DCBLD1/14-3-3 interaction was not further validated, however, the DCBLD2/14-3-3 interaction was investigated further using biochemical methods.

**Fig. 6:**
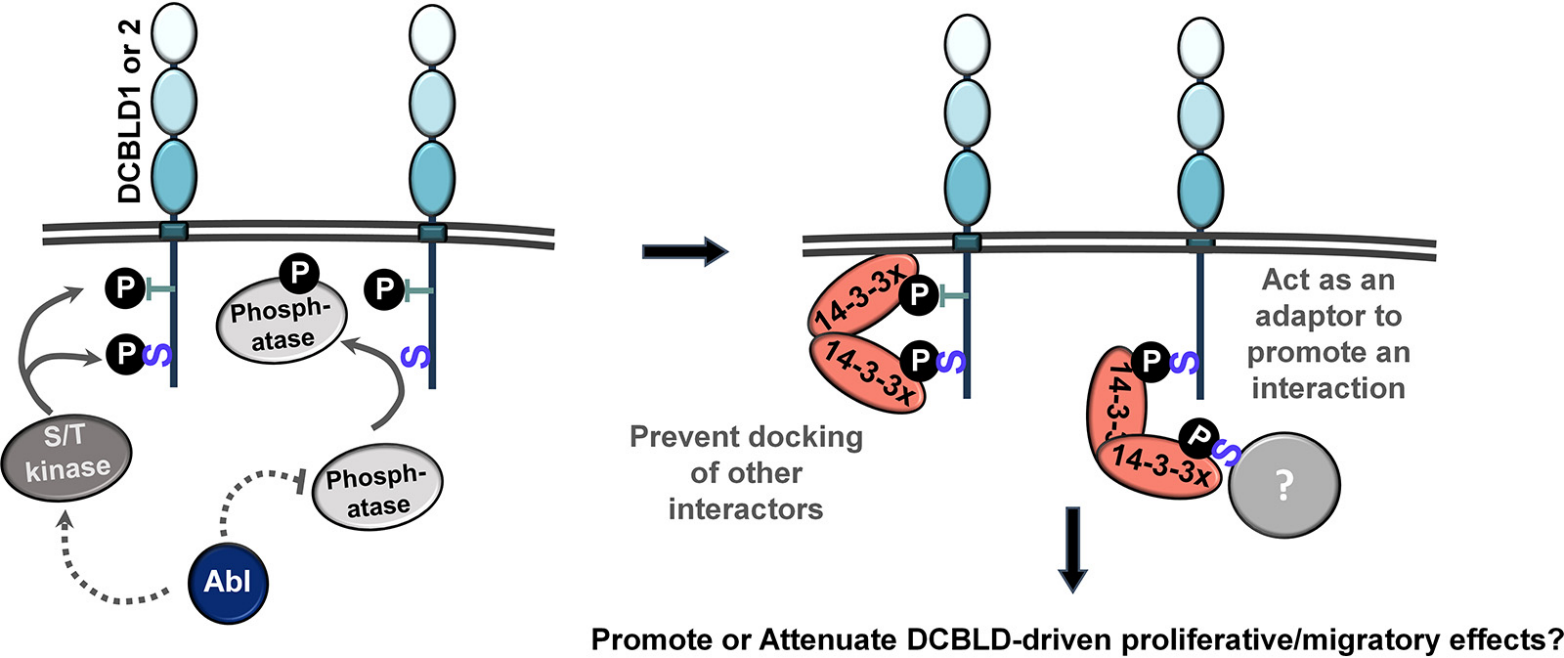
**Potential ABL-driven implications of the DCBLD/14-3-3 interaction.** The tyrosine kinase ABL could either directly or indirectly activate a serine/threonine kinase or inhibit a serine/threonine phosphatase, increasing levels of DCBLD1 or DCBLD2 phosphorylation in 14-3-3 binding motifs. This could recruit 14-3-3 family members to the membrane, to bind multiple motifs on DCBLD1 or 2, preventing docking of other DCBLD interactors. Alternatively, the binding of a 14-3-3 dimer to a single binding motif on DCBLD1 or 2 could facilitate interactions between DCBLD proteins and other 14-3-3 binding partners. The DCBLD/14-3-3 interaction could be either activating or inhibitory toward biological outcomes involving DCBLD family members, such as cell proliferation or migration.

To determine whether 14-3-3 family members were binding directly to DCBLD2, we conducted far-Western blotting assays (24). DCBLD2 was immunoprecipitated (α-MYC) from 293 cells expressing each family member with and without ABL. Immunoprecipitations were subjected to SDS-PAGE, transferred to nitrocellulose membranes, and incubated with eluted GST-14-3-3 fusion proteins overnight. Membranes were then subjected to immunoblotting (α-GST) to detect bound GST-14-3-3 proteins. Fusion proteins bound to the membrane at the same molecular weight of DCBLD2, suggesting that these interactions are likely direct (Fig. 5*B*). The far-Western blotting revealed a strongly ABL-induced DCBLD2/14-3-3 interaction, quantified in Fig. 5*C*, in agreement with the SILAC screen and the pulldown assays (Fig. 4*B*, Fig. 5*A*). This apparent regulation of a canonically pS/pT-mediated interaction by a tyrosine kinase suggests that either a serine/threonine phosphatase is inhibited downstream of ABL activity, or that ABL activation leads to the subsequent activation of a serine/threonine kinase (Fig. 6). It will be important for future studies to determine whether this interaction relates to known biological roles of DCBLD2, namely, cell proliferation and motility.

The DCBLD1/14-3-3 interaction was not further investigated biochemically because of the ∼10-fold decrease in DCBLD1 levels when ABL was co-expressed, however, the SILAC data suggests that the 14-3-3/DCBLD1 interaction increases with ABL co-expression (Fig. 3, Fig. 4). It remains possible that the observed increase in 14-3-3 binding in the SILAC experiments could be entirely because of the ABL/14-3-3 interaction (supplemental Fig. S4), however, 14-3-3 peptides were also observed in the “light” immune complex in which DCBLD1 was expressed alone (Fig. 3). Further investigations into this interaction will be an area of future study.

## DISCUSSION

The plasma membrane receptors DCBLD1 and DCBLD2 modulate basic cellular processes that are fundamental to vertebrate development, glucose homeostasis, and the progression of certain cancers (1, 2, 3, 4, 5, 6, 7, 8, 25, 26, 27, 28, 29, 30, 31). DCBLD2 expression affects the activation of downstream MAPKs or Akt following ligands binding to the receptor tyrosine kinases EGFR, VEGFR2, PDGFRβ, and INSR, altering phenotypes at the cellular level such as proliferation and migration (1, 2, 3, 4, 5, 6, 7, 8). EGFR and the cytoplasmic tyrosine kinases FYN and ABL, which can be activated downstream of RTKs, induce phosphorylation of DCBLD2 intracellular tyrosine residues leading to the recruitment of TNF Receptor Associated Factor 6 (TRAF6) or CRKL (8, 9, 10). Beyond these interactions, the molecular mechanisms of the DCBLD/RTK signaling interface that modulate the observed phenotypic outcomes in development and cancer are not well understood. Here, we further characterize the FYN- and ABL-regulated interactome of DCBLD family members, identifying and presenting several candidate protein interactors that could play roles in DCBLD signaling.

The known DCBLD interactor CRKL was identified in the SILAC screen, along with its homolog CRK (Fig. 4). This was not surprising, as the DCBLD/CRKL interaction is known to be mediated through the CRKL-SH2 domain, which binds DCBLD1 and DCBLD2 phosphotyrosine residues in YXXP motifs (9, 10). The SH2 domains of CRK family members are highly homologous. Therefore, the ABL-induced DCBLD/CRK interaction is likely similarly SH2/pYXXP-mediated. Although CRK and CRKL are likely involved in the proliferative or migratory effects of DCBLD signaling, the biological implications of this interaction are not yet understood.

DCBLD1 protein levels decreased considerably when WT ABL was co-transfected (Fig. 3). Phosphorylation by or downstream of activation of these cytoplasmic kinases appears to strongly regulate DCBLD family member degradation and/or solubility. The SILAC screen identified three ubiquitin-related enzymes that are recruited to the DCBLD1 scaffold downstream of active ABL, namely, the E3 ubiquitin ligase c-Cbl, the E3-independent E2 ubiquitin-conjugating enzyme (UBE2O), and the c-terminal ubiquitin hydrolase FAF-Y (USP9Y) (Fig. 4). c-Cbl possesses several potential interaction-mediating domains within its own sequence, including an SH2-like domain, but is often also brought to target protein via adaptors and is an important regulator of cell signaling. UBE2O and USP9Y could similarly contribute to the positive and negative regulation, respectively, of DCBLD1 ubiquitylation. Although no ubiquitin linker sequences were identified in the label free or SILAC analyses of DCBLD1 PTMs, ubiquitin-containing peptides were not targeted in the analyses and would be relatively rare, given the canonical role of ubiquitin as a degradation signal. DCBLD1 ubiquitylation could lead either to its proteasome-mediated degradation or endocytosis and subsequent degradation in lysosomes.

DCBLD2 protein loss was not strongly observed in this study, although we have previously reported evidence that suggests that FYN, in particular, is capable of inducing DCBLD2 degradation (9). Indeed, the deubiquitylating enzyme USP7 was identified in two biological replicates each of the DCBLD2/ABL and DCBLD2/FYN SILAC pairs (supplemental Table S4), however, proteins involved in ubiquitin dynamics were more highly concentrated in DCBLD1 immune complexes. Given that DCBLD1 levels are much more sensitive to kinase regulation than DCBLD2, we hypothesize that our ability to find ubiquitin-regulating proteins would be greater under conditions when DCBLD1 is being actively and highly degraded, such as with ABL co-expression. Therefore, that is likely the reason we found them bound to DCBLD1 at high abundance in the SILAC screen, and not in DCBLD2 immune complexes. This will be an area of future study, as we would like to understand how these proteins are getting degraded. We would start characterizing these interactors of DCBLD1, with the hope that they may help us determine whether similar pathways are involved with DCBLD2.

It should be noted that the loss of DCBLD1 protein could affect quantification. The label-free quantification to assess DCBLD1 phosphorylation was conducted with relatively higher protein levels to ensure that we had adequate intensities on DCBLD1 peptides to quantify phosphopeptide abundance. Cell extracts from several dishes were pooled to obtain signals of DCBLD1 phosphopeptide intensities that met signal-to-noise cutoffs for accurate label-free quantification. For the SILAC experiments, less protein was used to reduce nonspecific interactors, however, we still observed DCBLD1 peptides in all conditions that met the S/N cutoffs for quantification. Although the loss of DCBLD1 complicated biochemical assays that require lower protein quantities, such as Western blotting, the methods employed for mass spectrometric analysis of DCBLD1 phosphorylation sites and interacting partners permitted strong quantitative measurements. Interestingly, the mouse Dcbld1 construct that was initially characterize the Dcbld1/CRKL-SH2 interaction was not sensitive to ABL co-expression (9). Although it may be premature to make conclusions, we hypothesize that this is a difference between the human (use in the present study) and mouse DCBLD1 proteins, the most apparent difference between these sequences being the lack of the Discoidin domain in the mouse construct.

In addition, we identified and quantified both known and novel FYN- and/or ABL-regulated changes in phosphorylation along the intracellular sequence of DCBLD1 and DCBLD2, several of which are novel phosphorylation sites. The striking changes in population of serine, threonine, and tyrosine phosphorylation sites in the presence and absence FYN and ABL suggest that DCBLD signaling is strongly regulated by these cytoplasmic kinases. This is further supported by the DCBLD/14-3-3 interaction identified in the initial SILAC screen and, for DCBLD2, validated in biochemical assays as it is likely phosphorylation-dependent in nature. Indeed, several potential 14-3-3 binding sites (RXXS/T) that reside within DCBLD1 and DCBLD2 sequences were found phosphorylated and variably regulated by ABL (Fig. 1). Phosphorylation of DCBLD1 RXXS serine residues S513, S556, and S657 all increased in the presence of ABL, although S513 and S556 were also highly phosphorylated when DCBLD1 was expressed alone (Fig. 1). Although the DCBLD1/14-3-3 was not investigated further biochemically because of the loss of protein levels in the presence of ABL, the normalized SILAC ratios of 14-3-3 proteins in immune complex with DCBLD1 suggest that ABL increases the levels of 14-3-3 proteins bound to DCBLD1 (Fig. 3, Fig. 4). Two DCBLD2 serine residues in RXXS motifs (S599 and S724) were also found phosphorylated, although they were not strongly ABL-induced (Fig. 1). It remains possible that the DCBLD2/14-3-3 interaction could be mediated by phosphorylation outside the canonical motifs. It is still likely a phosphorylation-dependent interaction, given the strong evidence of a direct interaction between 14-3-3 proteins and DCBLD2 in the presence of ABL (Fig. 4, Fig. 5). These questions could be addressed with mutagenesis in future studies.

This observed ABL-induced DCBLD/14-3-3 interaction is likely mediated by ABL-regulated activity or localization of a serine/threonine kinase or phosphatase (Fig. 6). The SILAC screen for human DCBLD interactors identified members of the CaMKII holoenzyme, namely, the β and δ subunits (Fig. 4). An additional CaMKII subunit, γ, was identified alongside the δ subunit in zebrafish extracts (supplemental Fig. S2). Interestingly, CaMKII preferentially phosphorylates serine and threonine residues in RXXS/T motifs (supplemental Fig. S5) (32), the minimal canonical binding motif of 14-3-3 family members. CaMKII could phosphorylate DCBLD1 or DCBLD2, potentially through CaMKII activation downstream of ABL, in effect recruiting 14-3-3 family members to the DCBLD scaffold.

This work employed overexpression systems to elucidate phosphorylation sites and interactors of DCBLD family members, however, future work will aim to identify and characterize these interactions with endogenous proteins. Overexpression can drive interactions and phosphorylation that would not occur under normal conditions, however, it can also serve as a model for systems with elevated expression, such as at certain time points in development or in cancer biology. Future work will determine whether the phosphorylation sites and interactions identified in this screen are biologically relevant. It should be noted that the majority of FYN- and ABL-regulated phosphorylation sites identified in these studies have been previously reported on PhosphoSitePlus (Fig. 1). Our original identification of the DCBLD2/CRKL interaction identified endogenous DCBLD2 bound to the GST-CRKL-SH2 construct in cells with activated endogenous SFKs (10). With our later studies using endogenous kinases and SFK/ABL inhibitors, we were able to characterize phosphorylation at specific sites on DCBLD1 and 2, granted, under the overexpression of DCBLD proteins (9). We have yet to be able to purify sufficient levels of DCBLD proteins to conduct these same experiments at the endogenous level, although we hope to achieve this in the future. Even so, the data we have presented here represent the first step in identifying FYN- and ABL-regulated phosphorylation sites and interactors of DCBLD proteins, and future work will necessarily characterize biological systems in which these signaling events are relevant.

In summary, this work quantitatively characterizes the FYN- and ABL-regulated interacting proteins and maps regulated post-translational modifications of the scaffolding receptors DCBLD1 and DCBLD2. Insight provided by these studies will inform future work toward understanding the fundamental signaling mechanisms of DCBLD proteins, whose important roles are emerging in developmental processes, vascular repair, glucose homeostasis, oncogenesis and cancer progression.

## DATA AVAILABILITY

The MS proteomics data have been deposited to the ProteomeXchange Consortium via the PRIDE (33) partner repository with the data set identifier PXD017723.

10.13039/100000001National Science Foundation (NSF) (1656510) to Anna M. Schmoker, Jaye L. Weinert, Jacob M. Markwood, Kathryn S. Albretsen, Michelle L. Lunde, Marion E. Weir, Alicia M. Ebert, Karen L. Hinkle, and Bryan A. Ballif
10.13039/100000002HHS | National Institutes of Health (NIH) (8P20GM103449) to Anna M. Schmoker, Jaye L. Weinert, Marion E. Weir, and Bryan A. Ballif
