# Supplementary material for: FYN and ABL Regulate the Interaction Networks of the DCBLD Receptor Family
Source: Mol Cell Proteomics. 2020 Nov 25;19(10):1586–601. doi: 10.1074/mcp.RA120.002163 (PMC8015000; doi:10.1074/mcp.RA120.002163)
Supplement: Supplementary file 1 [file mmc1.zip › 161393_0_supp_544324_qbm51b.pdf]

Supplementary mass spectra of DCBLD1 and DCBLD2 phosphorylation sites. Spectra of peptides generated from the trypsin/GluC double digest of DCBLD family members housing serine, threonine, and tyrosine phosphorylation sites are preceded by a spectrum of the corresponding unphosphorylated peptide with the same charge state for comparison. In cases where spectra of the unphosphorylated and phosphorylated peptides were not obtained from the same charge state, a spectrum of an alternative charge state is included. Tables are included for each spectrum denoting expected fragment ion masses and mass accuracy (Δ Error, in ppm). Phosphorylated amino acids are denoted by @. Diagnostic ions confirming phosphorylated amino acids are starred (*). Experimental conditions in which each species was identified can found in Figure 1.

HEYALPLAPPEPEYATPIVER
z = 3+

DCBLD1 Y589, Y600, T602

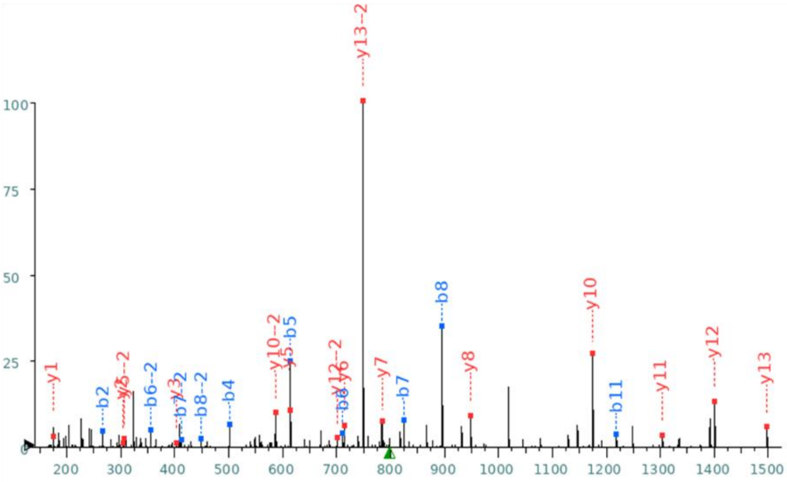

+1

Seq	#	b: Δ Error	b	y	y: Δ Error	+1
H	1	---	138.066	---	---	21
E	2	-2.118	267.109	2255.159	---	20
Y	3	---	430.172	2126.117	---	19
A	4	-1.207	501.209	1963.054	---	18
L	5	-1.214	614.293	1892.016	---	17
P	6	-1.690	711.346	1778.932	---	16
L	7	-2.221	824.430	1681.880	---	15
A	8	-1.641	895.467	1568.795	---	14
P	9	---	992.520	1497.758	-1.687	13
P	10	---	1089.573	1400.706	-0.650	12
E	11	0.807	1218.615	1303.653	-1.706	11
P	12	---	1315.668	1174.610	-1.485	10
E	13	---	1444.711	1077.558	---	9
Y	14	---	1607.774	948.515	-2.655	8
A	15	---	1678.811	785.452	0.258	7
T	16	---	1779.859	714.414	-2.615	6
P	17	---	1876.912	613.367	-1.437	5
I	18	---	1989.996	516.314	---	4
V	19	---	2089.064	403.230	-2.824	3
E	20	---	2218.107	304.162	-1.056	2
R	21	---	---	175.119	-1.801	1

+2

Seq	#	b: Δ Error	b	y	y: Δ Error	+1
H	1	---	69.537	---	---	21
E	2	---	134.058	1128.083	---	20
Y	3	---	215.590	1063.562	---	19
A	4	---	251.108	982.030	---	18
L	5	---	307.650	946.512	---	17
P	6	-3.334	356.177	889.970	---	16
L	7	-0.829	412.719	841.443	---	15
A	8	-3.015	448.237	784.901	---	14
P	9	---	496.764	749.383	-1.572	13
P	10	---	545.290	700.856	-1.137	12
E	11	---	609.811	652.330	---	11
P	12	---	658.338	587.809	-2.689	10
E	13	---	722.859	539.282	---	9
Y	14	---	804.391	474.761	---	8
A	15	---	839.909	393.229	---	7
T	16	---	890.433	357.711	---	6
P	17	---	938.959	307.187	-2.946	5
I	18	---	995.501	258.661	---	4
V	19	---	1045.036	202.119	---	3
E	20	---	1109.557	152.584	---	2
R	21	---	---	88.063	---	1

HEY@ALPLAPPEPEY@ATPIVER
z = 3+

DCBLD1 pY589, pY600

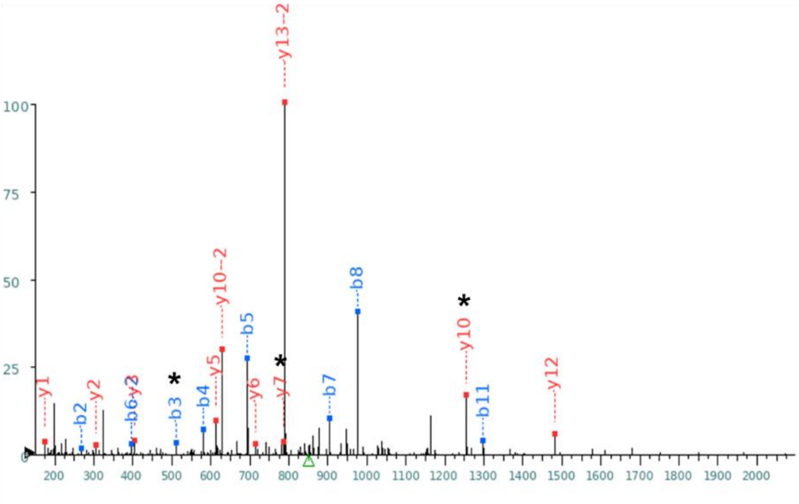

+1

Seq	#	b: Δ Error	b	y	y: Δ Error	+1
H	1	---	138.066	---	---	21
E	2	-0.975	267.109	2415.092	---	20
Y@	3	1.292	510.138	2286.049	---	19
A	4	-0.029	581.176	2043.020	---	18
L	5	-0.139	694.260	1971.983	---	17
P	6	---	791.312	1858.899	---	16
L	7	-0.227	904.396	1761.846	---	15
A	8	-0.340	975.434	1648.762	---	14
P	9	---	1072.486	1577.725	---	13
P	10	---	1169.539	1480.672	-1.866	12
E	11	1.022	1298.582	1383.619	---	11
P	12	---	1395.634	1254.577	0.343	10
E	13	---	1524.677	1157.524	---	9
Y@	14	---	1767.707	1028.481	---	8
A	15	---	1838.744	785.452	2.201	7
T	16	---	1939.791	714.414	1.742	6
P	17	---	2036.844	613.367	-0.044	5
I	18	---	2149.928	516.314	---	4
V	19	---	2248.997	403.230	-1.386	3
E	20	---	2378.039	304.162	-0.454	2
R	21	---	---	175.119	0.029	1

+2

Seq	#	b: Δ Error	b	y	y: Δ Error	+1
H	1	---	69.537	---	---	21
E	2	---	134.058	1208.050	---	20
Y@	3	---	255.573	1143.528	---	19
A	4	---	291.091	1022.014	---	18
L	5	---	347.633	986.495	---	17
P	6	-0.406	396.160	929.953	---	16
L	7	---	452.702	881.427	---	15
A	8	---	488.220	824.885	---	14
P	9	---	536.747	789.366	-0.192	13
P	10	---	585.273	740.840	---	12
E	11	---	649.794	692.313	---	11
P	12	---	698.321	627.792	-0.299	10
E	13	---	762.842	579.266	---	9
Y@	14	---	884.357	514.744	---	8
A	15	---	919.876	393.229	---	7
T	16	---	970.399	357.711	---	6
P	17	---	1018.926	307.187	---	5
I	18	---	1075.468	258.661	---	4
V	19	---	1125.002	202.119	---	3
E	20	---	1189.523	152.584	---	2
R	21	---	---	88.063	---	1

HEYALPLAPPEPEY@ATPIVER
z = 3+

DCBLD1 pY600

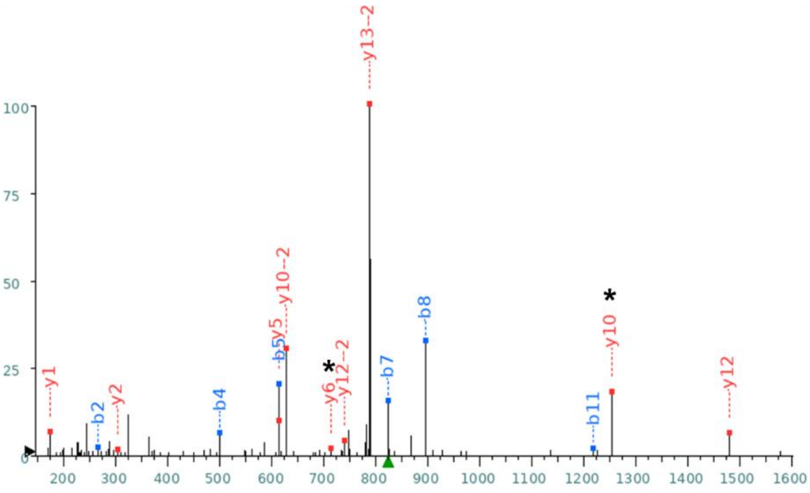

+1

Seq	#	b: Δ Error	b	y	y: Δ Error	+1
H	1	---	138.066	---	---	21
E	2	-0.632	267.109	2335.126	---	20
Y	3	---	430.172	2206.083	---	19
A	4	-0.842	501.209	2043.020	---	18
L	5	-0.519	614.293	1971.983	---	17
P	6	---	711.346	1858.899	---	16
L	7	-2.592	824.430	1761.846	---	15
A	8	-0.414	895.467	1648.762	---	14
P	9	---	992.520	1577.725	---	13
P	10	---	1089.573	1480.672	1.679	12
E	11	0.907	1218.615	1383.619	---	11
P	12	---	1315.668	1254.577	-0.435	10
E	13	---	1444.711	1157.524	---	9
Y@	14	---	1687.740	1028.481	---	8
A	15	---	1758.777	785.452	---	7
T	16	---	1859.825	714.414	0.290	6
P	17	---	1956.878	613.367	-0.641	5
I	18	---	2069.962	516.314	---	4
V	19	---	2169.030	403.230	---	3
E	20	---	2298.073	304.162	2.857	2
R	21	---	---	175.119	-1.279	1

+2

Seq	#	b: Δ Error	b	y	y: Δ Error	+1
H	1	---	69.537	---	---	21
E	2	---	134.058	1168.067	---	20
Y	3	---	215.590	1103.545	---	19
A	4	---	251.108	1022.014	---	18
L	5	---	307.650	986.495	---	17
P	6	---	356.177	929.953	---	16
L	7	---	412.719	881.427	---	15
A	8	---	448.237	824.885	---	14
P	9	---	496.764	789.366	-1.042	13
P	10	---	545.290	740.840	3.029	12
E	11	---	609.811	692.313	---	11
P	12	---	658.338	627.792	-1.563	10
E	13	---	722.859	579.266	---	9
Y@	14	---	844.374	514.744	---	8
A	15	---	879.892	393.229	---	7
T	16	---	930.416	357.711	---	6
P	17	---	978.943	307.187	---	5
I	18	---	1035.485	258.661	---	4
V	19	---	1085.019	202.119	---	3
E	20	---	1149.540	152.584	---	2
R	21	---	---	88.063	---	1

HEYALPLAPPEPEY@AT@PIVER
z = 3+

DCBLD1 pY600, pT602

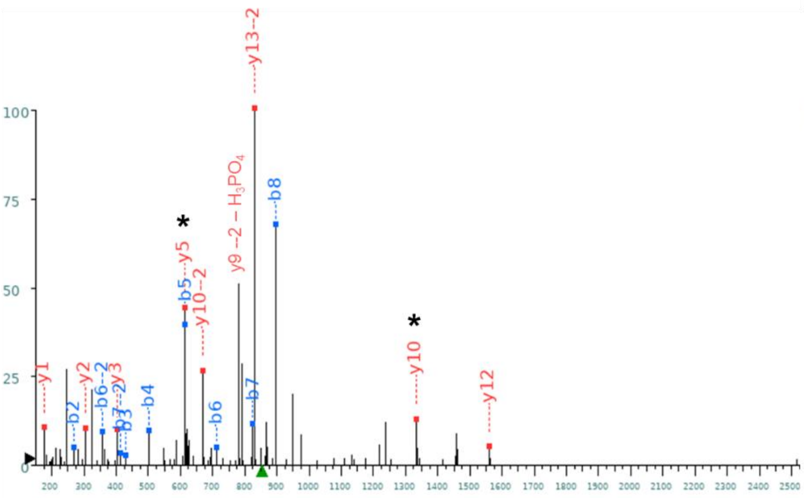

+1

Seq	#	b: Δ Error	b	y	y: Δ Error	+1
H	1	---	138.066	---	---	21
E	2	1.081	267.109	2415.092	---	20
Y	3	1.087	430.172	2286.049	---	19
A	4	-2.242	501.209	2122.986	---	18
L	5	-1.313	614.293	2051.949	---	17
P	6	-1.347	711.346	1938.865	---	16
L	7	-3.406	824.430	1841.812	---	15
A	8	-1.164	895.467	1728.728	---	14
P	9	---	992.520	1657.691	---	13
P	10	---	1089.573	1560.638	1.892	12
E	11	---	1218.615	1463.586	---	11
P	12	---	1315.668	1334.543	0.489	10
E	13	---	1444.711	1237.490	---	9
Y@	14	---	1687.740	1108.448	---	8
A	15	---	1758.777	865.418	---	7
T@	16	---	1939.791	794.381	---	6
P	17	---	2036.844	613.367	-0.641	5
I	18	---	2149.928	516.314	---	4
V	19	---	2248.997	403.230	-0.856	3
E	20	---	2378.039	304.162	-1.157	2
R	21	---	---	175.119	-2.237	1

+2

Seq	#	b: Δ Error	b	y	y: Δ Error	+1
H	1	---	69.537	---	---	21
E	2	---	134.058	1208.050	---	20
Y	3	---	215.590	1143.528	---	19
A	4	---	251.108	1061.997	---	18
L	5	---	307.650	1026.478	---	17
P	6	-1.106	356.177	969.936	---	16
L	7	-0.460	412.719	921.410	---	15
A	8	---	448.237	864.868	---	14
P	9	---	496.764	829.349	-1.226	13
P	10	---	545.290	780.823	---	12
E	11	---	609.811	732.296	---	11
P	12	---	658.338	667.775	-1.029	10
E	13	---	722.859	619.249	---	9
Y@	14	---	844.374	554.727	---	8
A	15	---	879.892	433.213	---	7
T@	16	---	970.399	397.694	---	6
P	17	---	1018.926	307.187	---	5
I	18	---	1075.468	258.661	---	4
V	19	---	1125.002	202.119	---	3
E	20	---	1189.523	152.584	---	2
R	21	---	---	88.063	---	1

HEYALPLAPPEPEYAT@PIVER

z = 3+

DCBLD1 pT602

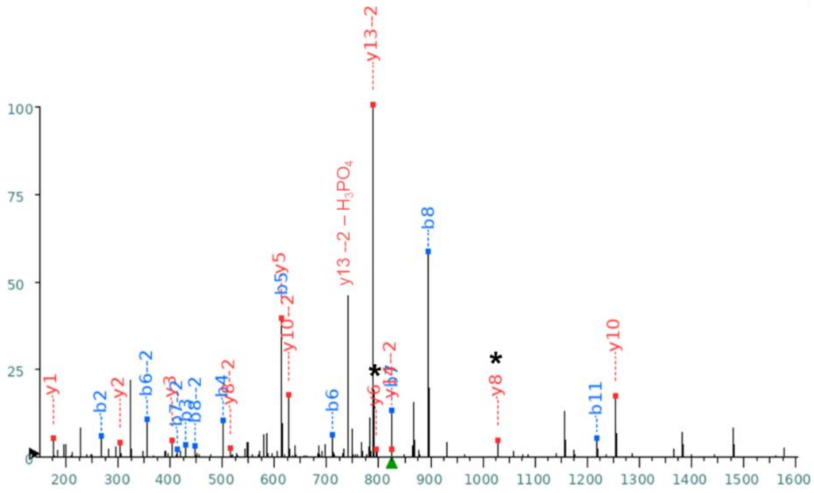

+1

Seq	#	b: Δ Error	b	y	y: Δ Error	+1
H	1	---	138.066	---	---	21
E	2	-2.575	**267.109**	2335.126	---	20
Y	3	-1.609	**430.172**	2206.083	---	19
A	4	-0.050	**501.209**	2043.020	---	18
L	5	-0.916	**614.293**	1971.983	---	17
P	6	-3.578	**711.346**	1858.899	---	16
L	7	-0.815	**824.430**	1761.846	---	15
A	8	-1.164	**895.467**	1648.762	---	14
P	9	---	992.520	1577.725	---	13
P	10	---	1089.573	1480.672	---	12
E	11	-0.996	**1218.615**	1383.619	---	11
P	12	---	1315.668	**1254.577**	-1.213	10
E	13	---	1444.711	1157.524	---	9
Y	14	---	1607.774	**1028.481**	-1.817	8
A	15	---	1678.811	865.418	---	7
T@	16	---	1859.825	**794.381**	-0.151	6
P	17	---	1956.878	**613.367**	-1.338	5
I	18	---	2069.962	516.314	---	4
V	19	---	2169.030	**403.230**	-1.083	3
E	20	---	2298.073	**304.162**	0.549	2
R	21	---	---	**175.119**	-2.063	1

+2

Seq	#	b: Δ Error	b	y	y: Δ Error	+1
H	1	---	69.537	---	---	21
E	2	---	134.058	1168.067	---	20
Y	3	---	215.590	1103.545	---	19
A	4	---	251.108	1022.014	---	18
L	5	---	307.650	986.495	---	17
P	6	-1.792	**356.177**	929.953	---	16
L	7	-1.421	**412.719**	881.427	---	15
A	8	-1.654	**448.237**	**824.885**	-2.332	14
P	9	---	496.764	**789.366**	-1.352	13
P	10	---	545.290	740.840	---	12
E	11	---	609.811	692.313	---	11
P	12	---	658.338	**627.792**	-1.368	10
E	13	---	722.859	579.266	---	9
Y	14	---	804.391	**514.744**	-1.412	8
A	15	---	839.909	433.213	---	7
T@	16	---	930.416	397.694	---	6
P	17	---	978.943	307.187	---	5
I	18	---	1035.485	258.661	---	4
V	19	---	1085.019	202.119	---	3
E	20	---	1149.540	152.584	---	2
R	21	---	---	88.063	---	1

KGSTFRPM*DTDAEEAGVSTDAGGHYDC^PQR

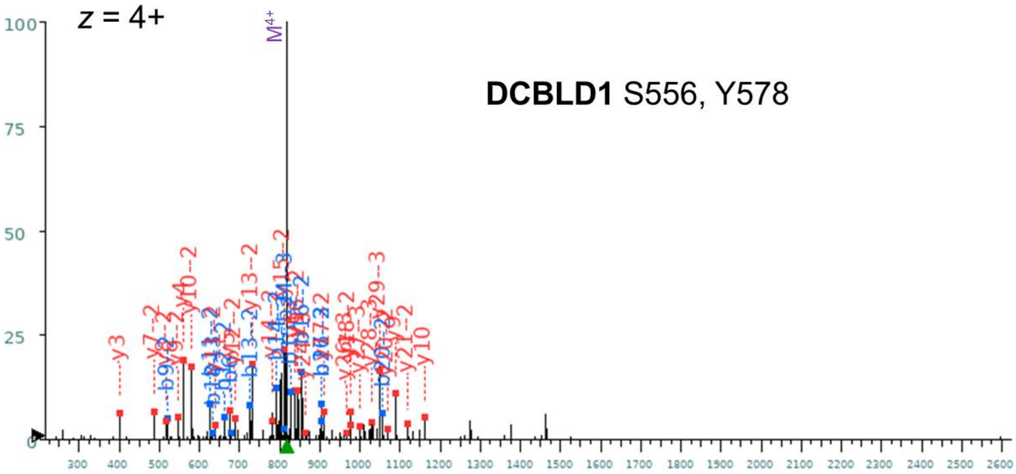

+1

Seq	#	b: Δ Error	b	y	y: Δ Error	+1
K	1	---	129.102	---	---	30
G	2	---	186.124	3143.307	---	29
S	3	---	273.156	3086.285	---	28
T	4	---	374.203	2999.253	---	27
F	5	---	521.272	2898.205	---	26
R	6	-0.195	677.373	2751.137	---	25
P	7	---	774.426	2595.036	---	24
M*	8	---	921.461	2497.983	---	23
D	9	---	1036.488	2350.948	---	22
T	10	---	1137.536	2235.921	---	21
D	11	---	1252.563	2134.873	---	20
A	12	---	1323.600	2019.846	---	19
E	13	---	1452.642	1948.809	---	18
E	14	---	1581.685	1819.766	---	17
A	15	---	1652.722	1690.724	---	16
G	16	---	1709.744	1619.687	---	15
V	17	---	1808.812	1562.665	---	14
S	18	---	1895.844	1463.597	---	13
T	19	---	1996.892	1376.565	---	12
D	20	---	2111.919	1275.517	---	11
A	21	---	2182.956	1160.490	0.374	10
G	22	---	2239.977	1089.453	-2.623	9
G	23	---	2296.999	1032.432	---	8
H	24	---	2434.058	975.410	0.470	7
Y	25	---	2597.121	838.351	-2.787	6
D	26	---	2712.148	675.288	1.111	5
C^	27	---	2872.178	560.261	-0.684	4
P	28	---	2969.231	400.230	-0.173	3
Q	29	---	3097.290	303.178	---	2
R	30	---	---	175.119	---	1

+2

Seq	#	b: Δ Error	b	y	y: Δ Error	+1
K	1	---	65.055	---	---	30
G	2	---	93.565	1572.157	---	29
S	3	---	137.082	1543.646	---	28
T	4	---	187.605	1500.130	---	27
F	5	---	261.140	1449.606	---	26
R	6	---	339.190	1376.072	---	25
P	7	---	387.716	1298.022	---	24
M*	8	---	461.234	1249.495	---	23
D	9	0.278	518.748	1175.977	---	22
T	10	---	569.271	1118.464	-1.213	21
D	11	0.299	626.785	1067.940	-2.266	20
A	12	-1.840	662.304	1010.427	---	19
E	13	-0.243	726.825	974.908	-0.431	18
E	14	-0.217	791.346	910.387	-2.612	17
A	15	-1.244	826.865	845.866	-1.590	16
G	16	-0.049	855.375	810.347	-0.528	15
V	17	-0.211	904.910	781.836	-0.950	14
S	18	---	948.426	732.302	-0.811	13
T	19	---	998.949	688.786	-1.803	12
D	20	-0.047	1056.463	638.262	1.170	11
A	21	---	1091.981	580.749	-0.321	10
G	22	---	1120.492	545.230	-0.897	9
G	23	---	1149.003	516.719	-4.510	8
H	24	---	1217.532	488.209	0.331	7
Y	25	---	1299.064	419.679	---	6
D	26	---	1356.578	338.148	---	5
C^	27	---	1436.593	280.634	---	4
P	28	---	1485.119	200.619	---	3
Q	29	---	1549.149	152.092	---	2
R	30	---	---	88.063	---	1

+3

Seq	#	b: Δ Error	b	y	y: Δ Error	+1
K	1	---	43.706	---	---	30
G	2	---	62.713	1048.440	0.298	29
S	3	---	91.723	1029.433	0.732	28
T	4	---	125.406	1000.423	2.334	27
F	5	---	174.429	966.740	-1.558	26
R	6	---	226.462	917.717	---	25
P	7	---	258.813	865.683	-1.893	24
M*	8	---	307.825	833.333	---	23
D	9	---	346.168	784.321	---	22
T	10	---	379.850	745.978	---	21
D	11	---	418.192	712.296	---	20
A	12	---	441.871	673.954	---	19
E	13	---	484.886	650.275	---	18
E	14	---	527.900	607.260	---	17
A	15	---	551.579	564.246	---	16
G	16	---	570.586	540.567	---	15
V	17	---	603.609	521.560	---	14
S	18	0.572	632.620	488.537	---	13
T	19	---	666.302	459.526	---	12
D	20	---	704.644	425.844	---	11
A	21	---	728.323	387.502	---	10
G	22	---	747.331	363.823	---	9
G	23	---	766.338	344.815	---	8
H	24	0.616	812.024	325.808	---	7
Y	25	---	866.378	280.122	---	6
D	26	3.206	904.721	225.767	---	5
C^	27	---	958.064	187.425	---	4
P	28	---	990.415	134.082	---	3
Q	29	---	1033.101	101.731	---	2
R	30	---	---	59.045	---	1

KGS@TFRPMDTDAEEAGVSTDAGGHYDC^PQR

z = 4+

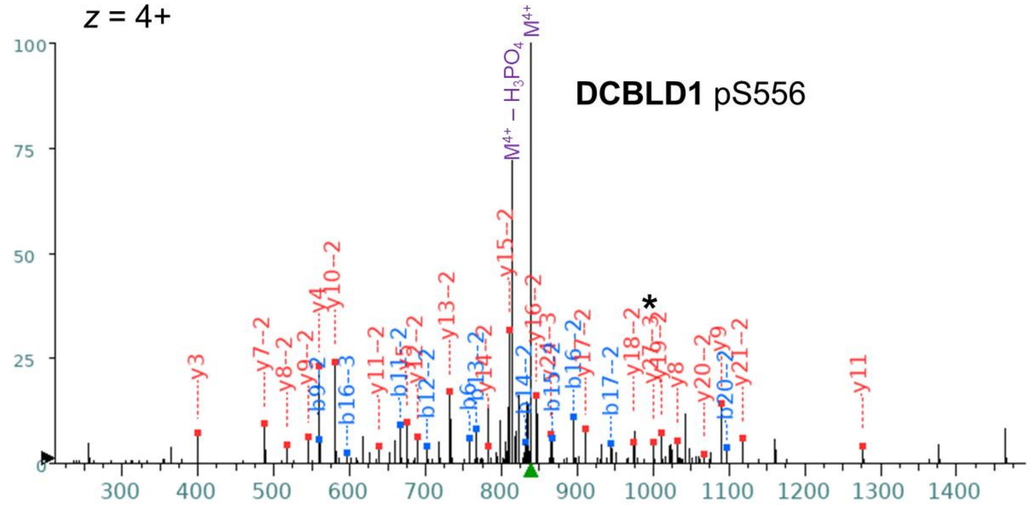

+1

eq	#	b: Δ Error	b	y	y: Δ Error	+1
K	1	---	129.102	---	---	30
G	2	---	186.124	3223.273	---	29
S@	3	---	353.122	3166.251	---	28
T	4	---	454.170	2999.253	---	27
F	5	---	601.238	2898.205	---	26
R	6	-1.895	757.339	2751.137	---	25
P	7	---	854.392	2595.036	---	24
M*	8	---	1001.427	2497.983	---	23
D	9	---	1116.454	2350.948	---	22
T	10	---	1217.502	2235.921	---	21
D	11	---	1332.529	2134.873	---	20
A	12	---	1403.566	2019.846	---	19
E	13	---	1532.609	1948.809	---	18
E	14	---	1661.651	1819.766	---	17
A	15	---	1732.688	1690.724	---	16
G	16	---	1789.710	1619.687	---	15
V	17	---	1888.778	1562.665	---	14
S	18	---	1975.810	1463.597	---	13
T	19	---	2076.858	1376.565	---	12
D	20	---	2191.885	1275.517	0.367	11
A	21	---	2262.922	1160.490	---	10
G	22	---	2319.944	1089.453	-1.391	9
G	23	---	2376.965	1032.432	0.523	8
H	24	---	2514.024	975.410	---	7
Y	25	---	2677.087	838.351	---	6
D	26	---	2792.114	675.288	0.117	5
C^	27	---	2952.145	560.261	0.624	4
P	28	---	3049.198	400.230	-0.249	3
Q	29	---	3177.256	303.178	---	2
R	30	---	---	175.119	---	1

+2

eq	#	b: Δ Error	b	y	y: Δ Error	+1
K	1	---	65.055	---	---	30
G	2	---	93.565	1612.140	---	29
S@	3	---	177.065	1583.629	---	28
T	4	---	227.589	1500.130	---	27
F	5	---	301.123	1449.606	---	26
R	6	---	379.173	1376.072	---	25
P	7	---	427.700	1298.022	---	24
M*	8	---	501.217	1249.495	---	23
D	9	0.020	558.731	1175.977	---	22
T	10	---	609.255	1118.464	2.389	21
D	11	2.187	666.768	1067.940	1.278	20
A	12	-1.143	702.287	1010.427	0.609	19
E	13	-2.394	766.808	974.908	-2.059	18
E	14	0.221	831.329	910.387	0.874	17
A	15	-0.706	866.848	845.866	-1.013	16
G	16	-2.581	895.359	810.347	1.581	15
V	17	-1.441	944.893	781.836	-2.512	14
S	18	---	988.409	732.302	0.606	13
T	19	---	1038.933	688.786	-1.183	12
D	20	0.279	1096.446	638.262	-1.412	11
A	21	---	1131.965	580.749	0.520	10
G	22	---	1160.475	545.230	-0.114	9
G	23	---	1188.986	516.719	-0.140	8
H	24	---	1257.516	488.209	-0.482	7
Y	25	---	1339.047	419.679	---	6
D	26	---	1396.561	338.148	---	5
C^	27	---	1476.576	280.634	---	4
P	28	---	1525.102	200.619	---	3
Q	29	---	1589.132	152.092	---	2
R	30	---	---	88.063	---	1

+3

eq	#	b: Δ Error	b	y	y: Δ Error	+1
K	1	---	43.706	---	---	30
G	2	---	62.713	1075.096	---	29
S@	3	---	118.379	1056.089	---	28
T	4	---	152.061	1000.423	-2.607	27
F	5	---	201.084	966.740	---	26
R	6	---	253.118	917.717	---	25
P	7	---	285.469	865.683	-0.624	24
M*	8	---	334.481	833.333	---	23
D	9	---	372.823	784.321	---	22
T	10	---	406.506	745.978	---	21
D	11	---	444.848	712.296	---	20
A	12	---	468.527	673.954	---	19
E	13	---	511.541	650.275	---	18
E	14	---	554.555	607.260	---	17
A	15	---	578.234	564.246	---	16
G	16	4.874	597.241	540.567	---	15
V	17	---	630.264	521.560	---	14
S	18	---	659.275	488.537	---	13
T	19	---	692.958	459.526	---	12
D	20	---	731.300	425.844	---	11
A	21	---	754.979	387.502	---	10
G	22	---	773.986	363.823	---	9
G	23	---	792.993	344.815	---	8
H	24	---	838.679	325.808	---	7
Y	25	---	893.034	280.122	---	6
D	26	---	931.376	225.767	---	5
C^	27	---	984.720	187.425	---	4
P	28	---	1017.071	134.082	---	3
Q	29	---	1059.757	101.731	---	2
R	30	---	---	59.045	---	1

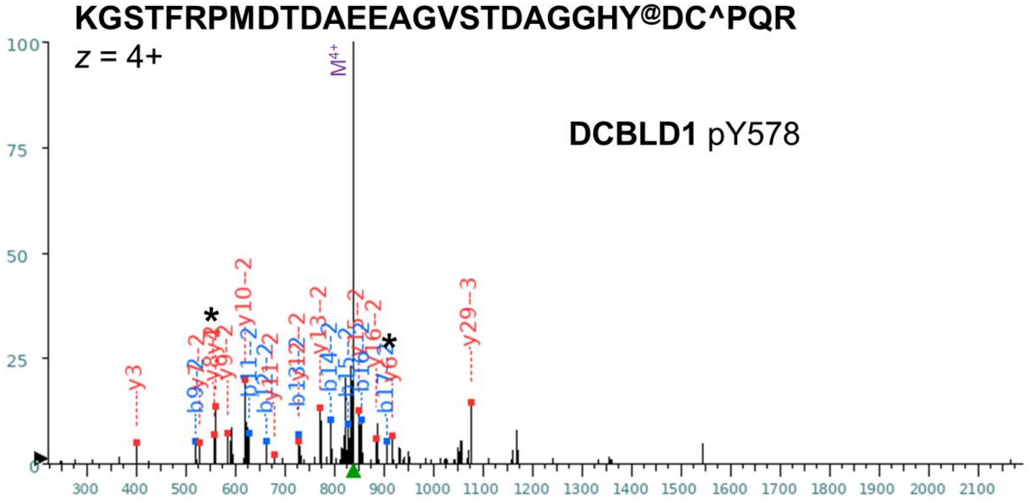

+1

Seq	#	b: Δ Error	b	y	y: Δ Error	+1
K	1	---	129.102	---	---	30
G	2	---	186.124	3223.273	---	29
S	3	---	273.156	3166.251	---	28
T	4	---	374.203	3079.219	---	27
F	5	---	521.272	2978.172	---	26
R	6	---	677.373	2831.103	---	25
P	7	---	774.426	2675.002	---	24
M*	8	---	921.461	2577.949	---	23
D	9	---	1036.488	2430.914	---	22
T	10	---	1137.536	2315.887	---	21
D	11	---	1252.563	2214.839	---	20
A	12	---	1323.600	2099.812	---	19
E	13	---	1452.642	2028.775	---	18
E	14	---	1581.685	1899.733	---	17
A	15	---	1652.722	1770.690	---	16
G	16	---	1709.744	1699.653	---	15
V	17	---	1808.812	1642.632	---	14
S	18	---	1895.844	1543.563	---	13
T	19	---	1996.892	1456.531	---	12
D	20	---	2111.919	1355.483	---	11
A	21	---	2182.956	1240.456	---	10
G	22	---	2239.977	1169.419	---	9
G	23	---	2296.999	1112.398	---	8
H	24	---	2434.058	1055.376	---	7
Y@	25	---	2677.087	**918.318**	0.157	6
D	26	---	2792.114	675.288	---	5
C^	27	---	2952.145	**560.261**	0.079	4
P	28	---	3049.198	**400.230**	-1.698	3
Q	29	---	3177.256	303.178	---	2
R	30	---	---	175.119	---	1

+2

Seq	#	b: Δ Error	b	y	y: Δ Error	+1
K	1	---	65.055	---	---	30
G	2	---	93.565	1612.140	---	29
S	3	---	137.082	1583.629	---	28
T	4	---	187.605	1540.113	---	27
F	5	---	261.140	1489.589	---	26
R	6	---	339.190	1416.055	---	25
P	7	---	387.716	1338.005	---	24
M*	8	---	461.234	1289.478	---	23
D	9	-0.663	**518.748**	1215.961	---	22
T	10	---	569.271	1158.447	---	21
D	11	-1.552	**626.785**	1107.923	---	20
A	12	0.556	**662.304**	1050.410	---	19
E	13	1.856	**726.825**	1014.891	---	18
E	14	-0.449	**791.346**	950.370	---	17
A	15	0.380	**826.865**	**885.849**	-0.360	16
G	16	0.379	**855.375**	**850.330**	-1.736	15
V	17	0.126	**904.910**	821.819	---	14
S	18	---	948.426	**772.285**	-0.862	13
T	19	---	998.949	**728.769**	-2.724	12
D	20	---	1056.463	**678.245**	-3.325	11
A	21	---	1091.981	**620.732**	-0.711	10
G	22	---	1120.492	**585.213**	-0.750	9
G	23	---	1149.003	**556.703**	-0.698	8
H	24	---	1217.532	**528.192**	-0.986	7
Y@	25	---	1339.047	459.662	---	6
D	26	---	1396.561	338.148	---	5
C^	27	---	1476.576	280.634	---	4
P	28	---	1525.102	200.619	---	3
Q	29	---	1589.132	152.092	---	2
R	30	---	---	88.063	---	1

+3

Seq	#	b: Δ Error	b	y	y: Δ Error	+1
K	1	---	43.706	---	---	30
G	2	---	62.713	**1075.096**	0.133	29
S	3	---	91.723	1056.089	---	28
T	4	---	125.406	1027.078	---	27
F	5	---	174.429	993.395	---	26
R	6	---	226.462	944.373	---	25
P	7	---	258.813	892.339	---	24
M*	8	---	307.825	859.988	---	23
D	9	---	346.168	810.976	---	22
T	10	---	379.850	772.634	---	21
D	11	---	418.192	738.951	---	20
A	12	---	441.871	700.609	---	19
E	13	---	484.886	676.930	---	18
E	14	---	527.900	633.916	---	17
A	15	---	551.579	590.902	---	16
G	16	---	570.586	567.223	---	15
V	17	---	603.609	548.215	---	14
S	18	---	632.620	515.193	---	13
T	19	---	666.302	486.182	---	12
D	20	---	704.644	452.499	---	11
A	21	---	728.323	414.157	---	10
G	22	---	747.331	390.478	---	9
G	23	---	766.338	371.471	---	8
H	24	---	812.024	352.464	---	7
Y@	25	---	893.034	306.777	---	6
D	26	---	931.376	225.767	---	5
C^	27	---	984.720	187.425	---	4
P	28	---	1017.071	134.082	---	3
Q	29	---	1059.757	101.731	---	2
R	30	---	---	59.045	---	1

KGS@TFRPMDTDAEEAGVSTDAGGHY@DC^PQR

$z = 4+$

DCBLD1 pS556, pY578

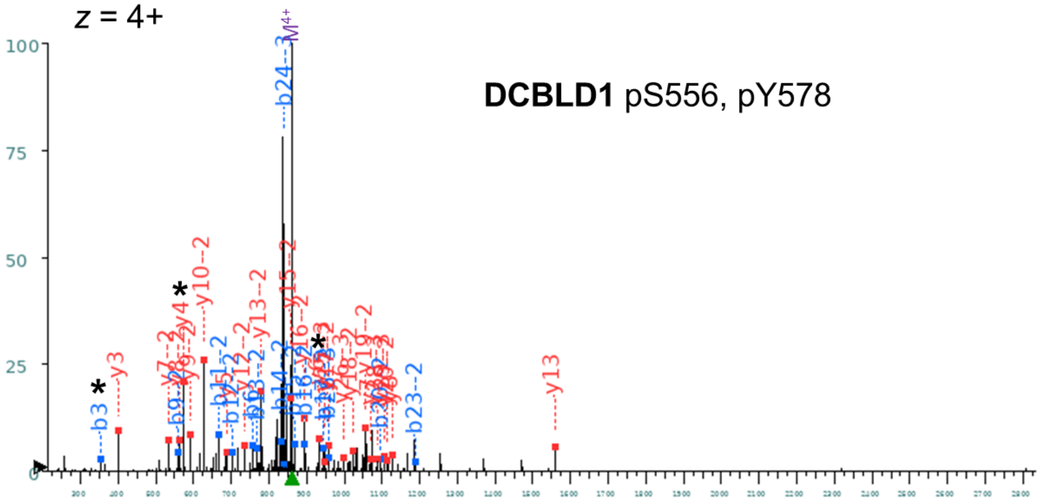

+1

Seq	#	b: Δ Error	b	y	y: Δ Error	+1
K	1	---	129.102	---	---	30
G	2	---	186.124	3317.255	---	29
S@	3	-2.741	**353.122**	3260.233	---	28
T	4	---	454.170	3093.235	---	27
F	5	---	601.238	2992.187	---	26
R	6	0.764	**757.339**	2845.119	---	25
P	7	---	854.392	2689.018	---	24
M*	8	---	1001.427	2591.965	---	23
D	9	---	1116.454	2444.930	---	22
T	10	---	1217.502	2329.903	---	21
D	11	---	1332.529	2228.855	---	20
A	12	---	1403.566	2113.828	---	19
E	13	---	1532.609	2042.791	---	18
E	14	---	1661.651	1913.748	---	17
A	15	---	1732.688	1784.706	---	16
G	16	---	1789.710	1713.669	---	15
V	17	---	1888.778	1656.647	---	14
S	18	---	1975.810	**1557.579**	-0.980	13
T	19	---	2076.858	1470.547	---	12
D	20	---	2191.885	1369.499	---	11
A	21	---	2262.922	1254.472	---	10
G	22	---	2319.944	1183.435	---	9
G	23	---	2376.965	**1126.414**	0.763	8
H	24	---	2514.024	**1069.392**	0.670	7
Y@	25	---	2757.054	**932.333**	-2.622	6
D	26	---	2872.080	**689.304**	-2.756	5
C#	27	---	3046.127	**574.277**	-0.711	4
P	28	---	3143.180	**400.230**	-2.842	3
Q	29	---	3271.238	303.178	---	2
R	30	---	---	175.119	---	1

+2

Seq	#	b: Δ Error	b	y	y: Δ Error	+1
K	1	---	65.055	---	---	30
G	2	---	93.565	1659.131	---	29
S@	3	---	177.065	1630.620	---	28
T	4	---	227.589	1547.121	---	27
F	5	---	301.123	1496.597	---	26
R	6	---	379.173	1423.063	---	25
P	7	---	427.700	1345.013	---	24
M*	8	---	501.217	1296.486	---	23
D	9	-1.181	**558.731**	1222.968	---	22
T	10	---	609.255	1165.455	---	21
D	11	-0.560	**666.768**	**1114.931**	-1.863	20
A	12	-1.925	**702.287**	**1057.418**	-1.461	19
E	13	-1.518	**766.808**	**1021.899**	0.163	18
E	14	-0.367	**831.329**	**957.378**	0.552	17
A	15	-2.959	**866.848**	**892.857**	-1.328	16
G	16	-2.513	**895.359**	**857.338**	-1.380	15
V	17	-2.022	**944.893**	828.827	---	14
S	18	---	988.409	**779.293**	-2.515	13
T	19	---	1038.933	**735.777**	0.354	12
D	20	-0.055	**1096.446**	685.253	---	11
A	21	---	1131.965	**627.740**	-1.112	10
G	22	---	1160.475	**592.221**	-1.793	9
G	23	-0.446	**1188.986**	**563.710**	-1.036	8
H	24	---	1257.516	**535.200**	-1.339	7
Y@	25	---	1379.030	466.670	---	6
D	26	---	1436.544	345.155	---	5
C#	27	---	1523.567	287.642	---	4
P	28	---	1572.093	200.619	---	3
Q	29	---	1636.123	152.092	---	2
R	30	---	---	88.063	---	1

+3

Seq	#	b: Δ Error	b	y	y: Δ Error	+1
K	1	---	43.706	---	---	30
G	2	---	62.713	**1106.423**	0.850	29
S@	3	---	118.379	**1087.416**	-0.413	28
T	4	---	152.061	1031.750	---	27
F	5	---	201.084	**998.067**	-2.361	26
R	6	---	253.118	**949.044**	-1.670	25
P	7	---	285.469	897.011	---	24
M*	8	---	334.481	864.660	---	23
D	9	---	372.823	815.648	---	22
T	10	---	406.506	777.306	---	21
D	11	---	444.848	743.623	---	20
A	12	---	468.527	705.281	---	19
E	13	---	511.541	681.602	---	18
E	14	---	554.555	638.588	---	17
A	15	---	578.234	595.573	---	16
G	16	---	597.241	571.894	---	15
V	17	---	630.264	552.887	---	14
S	18	---	659.275	519.864	---	13
T	19	---	692.958	490.854	---	12
D	20	---	731.300	457.171	---	11
A	21	---	754.979	418.829	---	10
G	22	---	773.986	395.150	---	9
G	23	---	792.993	376.143	---	8
H	24	-0.334	**838.679**	357.136	---	7
Y@	25	---	919.689	311.449	---	6
D	26	2.035	**958.032**	230.439	---	5
C#	27	---	1016.047	192.097	---	4
P	28	---	1048.398	134.082	---	3
Q	29	---	1091.084	101.731	---	2
R	30	---	---	59.045	---	1

HQSAEFTISYDNEKE
z = 3+

DCBLD1 S513, T517

+1

Seq	#	b: Δ Error	b	y	y: Δ Error	+1
H	1	---	138.066	---	---	15
Q	2	-1.871	**266.125**	1660.734	---	14
S	3	-0.330	**353.157**	1532.675	---	13
A	4	0.794	**424.194**	1445.643	---	12
E	5	-1.194	**553.237**	1374.606	---	11
F	6	-1.457	**700.305**	1245.563	---	10
T	7	-0.905	**801.353**	**1098.495**	-0.191	9
I	8	---	914.437	**997.447**	0.473	8
S	9	---	1001.469	**884.363**	-0.826	7
Y	10	---	1164.532	**797.331**	-0.705	6
D	11	---	1279.559	**634.268**	-0.061	5
N	12	---	1393.602	**519.241**	-0.494	4
E	13	---	1522.644	**405.198**	-2.844	3
K	14	---	1650.739	**276.155**	-0.339	2
E	15	---	---	**148.060**	-2.127	1

+2

Seq	#	b: Δ Error	b	y	y: Δ Error	+1
H	1	---	69.537	---	---	15
Q	2	---	133.566	830.870	---	14
S	3	---	177.082	766.841	---	13
A	4	---	212.601	723.325	---	12
E	5	-2.867	**277.122**	687.807	---	11
F	6	-1.038	**350.656**	623.285	---	10
T	7	0.449	**401.180**	549.751	---	9
I	8	---	457.722	499.227	---	8
S	9	---	501.238	442.685	---	7
Y	10	---	582.770	399.169	---	6
D	11	---	640.283	317.638	---	5
N	12	---	697.305	260.124	---	4
E	13	---	761.826	203.103	---	3
K	14	---	825.873	138.581	---	2
E	15	---	---	74.534	---	1

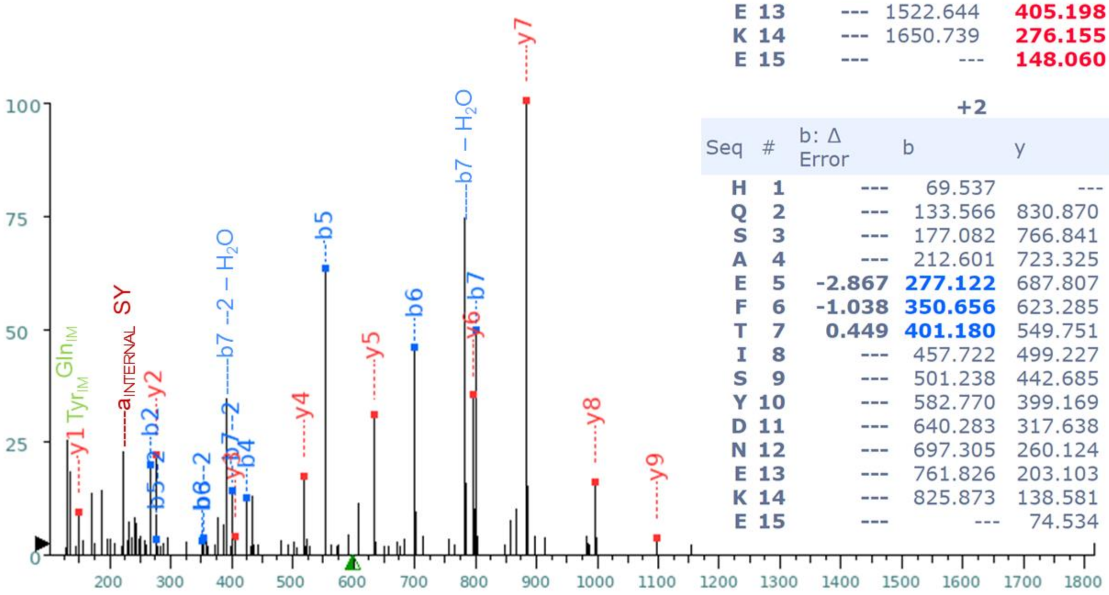

HQS@AEFTISYDNEKE
z = 3+

DCBLD1 pS513

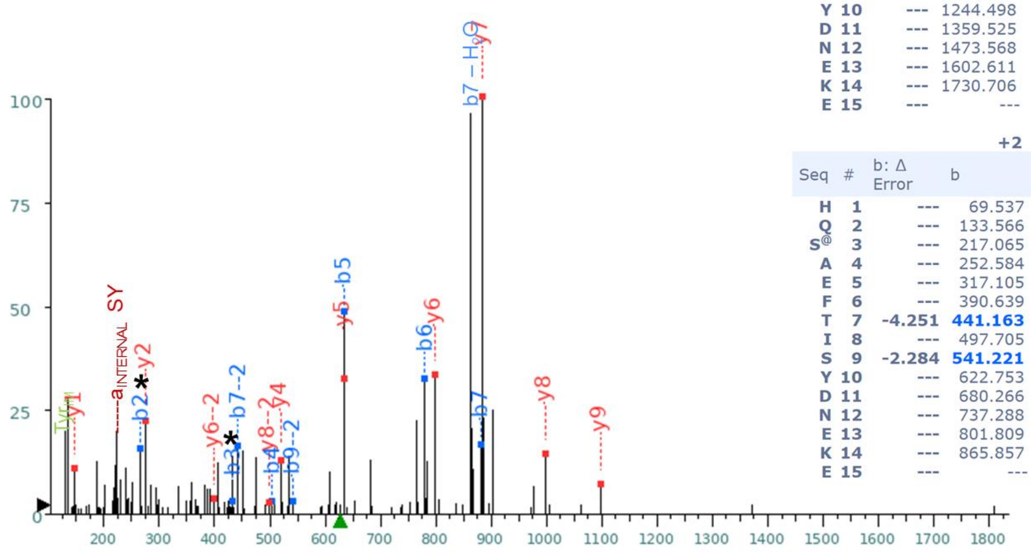

+1

Seq	#	b: Δ Error	b	y	y: Δ Error	+1
H	1	---	138.066	---	---	15
Q	2	-3.820	266.125	1740.700	---	14
S@	3	-4.970	433.123	1612.641	---	13
A	4	-2.583	504.160	1445.643	---	12
E	5	-0.789	633.203	1374.606	---	11
F	6	-3.526	780.271	1245.563	---	10
T	7	-0.848	881.319	1098.495	2.143	9
I	8	---	994.403	997.447	-0.628	8
S	9	---	1081.435	884.363	-1.585	7
Y	10	---	1244.498	797.331	-1.853	6
D	11	---	1359.525	634.268	-0.158	5
N	12	---	1473.568	519.241	-3.315	4
E	13	---	1602.611	405.198	---	3
K	14	---	1730.706	276.155	-2.881	2
E	15	---	---	148.060	-3.261	1

+2

Seq	#	b: Δ Error	b	y	y: Δ Error	+1
H	1	---	69.537	---	---	15
Q	2	---	133.566	870.854	---	14
S@	3	---	217.065	806.824	---	13
A	4	---	252.584	723.325	---	12
E	5	---	317.105	687.807	---	11
F	6	---	390.639	623.285	---	10
T	7	-4.251	441.163	549.751	---	9
I	8	---	497.705	499.227	-0.335	8
S	9	-2.284	541.221	442.685	---	7
Y	10	---	622.753	399.169	0.274	6
D	11	---	680.266	317.638	---	5
N	12	---	737.288	260.124	---	4
E	13	---	801.809	203.103	---	3
K	14	---	865.857	138.581	---	2
E	15	---	---	74.534	---	1

HQSAEFT@ISYDNEKE
z = 3+

DCBLD1 pT517

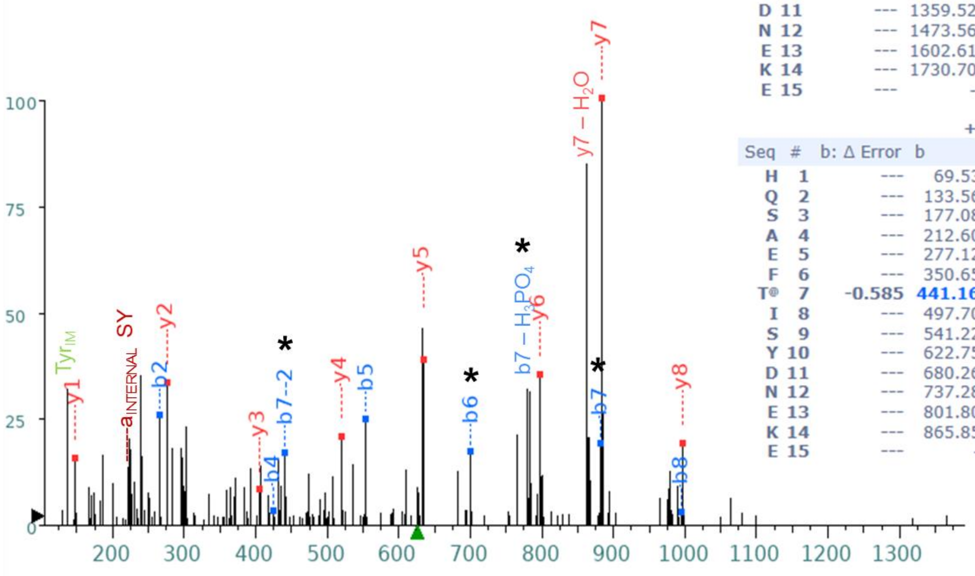

+1

Seq	#	b: Δ Error	b	y	y: Δ Error	+1
H	1	---	138.066	---	---	15
Q	2	-1.182	266.125	1740.700	---	14
S	3	---	353.157	1612.641	---	13
A	4	-2.371	424.194	1525.609	---	12
E	5	-1.525	553.237	1454.572	---	11
F	6	-1.806	700.305	1325.530	---	10
T@	7	-3.064	881.319	1178.461	---	9
I	8	1.746	994.403	997.447	-1.302	8
S	9	---	1081.435	884.363	-2.413	7
Y	10	---	1244.498	797.331	-2.466	6
D	11	---	1359.525	634.268	-2.563	5
N	12	---	1473.568	519.241	-1.317	4
E	13	---	1602.611	405.198	-0.735	3
K	14	---	1730.706	276.155	-2.770	2
E	15	---	---	148.060	-2.230	1

+2

Seq	#	b: Δ Error	b	y	y: Δ Error	+1
H	1	---	69.537	---	---	15
Q	2	---	133.566	870.854	---	14
S	3	---	177.082	806.824	---	13
A	4	---	212.601	763.308	---	12
E	5	---	277.122	727.790	---	11
F	6	---	350.656	663.268	---	10
T@	7	-0.585	441.163	589.734	---	9
I	8	---	497.705	499.227	---	8
S	9	---	541.221	442.685	---	7
Y	10	---	622.753	399.169	---	6
D	11	---	680.266	317.638	---	5
N	12	---	737.288	260.124	---	4
E	13	---	801.809	203.103	---	3
K	14	---	865.857	138.581	---	2
E	15	---	---	74.534	---	1

AHTFSAQSGYRVPGPQPGHK
z = 4+

DCBLD1 T614, S616, Y621

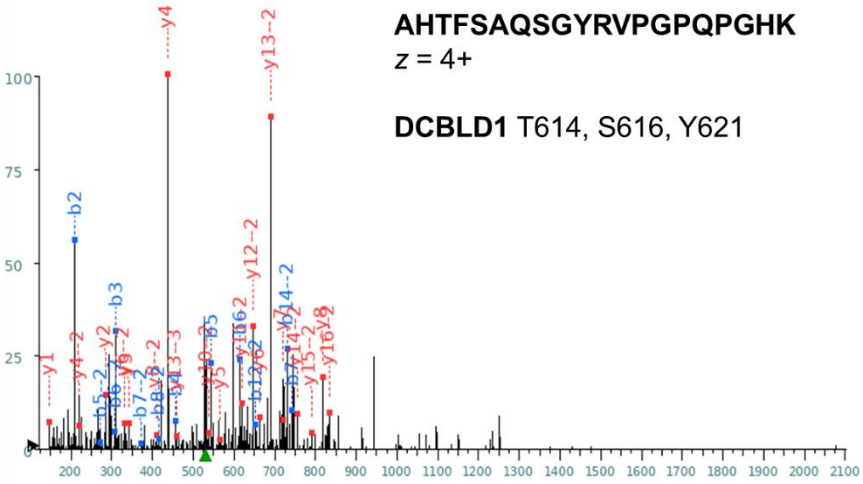

+1

Seq	#	b: Δ Error	b	y	y: Δ Error	+1
A	1	---	72.044	---	---	20
H	2	-0.436	**209.103**	2051.021	---	19
T	3	-0.818	**310.151**	1913.962	---	18
F	4	-2.143	**457.219**	1812.914	---	17
S	5	-0.259	**544.251**	1665.846	---	16
A	6	-0.732	**615.289**	1578.814	---	15
Q	7	-2.144	**743.347**	1507.776	---	14
S	8	---	830.379	1379.718	---	13
G	9	---	887.401	1292.686	---	12
Y	10	---	1050.464	1235.664	---	11
R	11	---	1206.565	1072.601	---	10
V	12	---	1305.633	916.500	---	9
P	13	---	1402.686	**817.432**	0.082	8
G	14	---	1459.708	**720.379**	0.472	7
P	15	---	1556.760	**663.357**	0.666	6
Q	16	---	1684.819	**566.305**	0.832	5
P	17	---	1781.872	**438.246**	-0.355	4
G	18	---	1838.893	**341.193**	-0.995	3
H	19	---	1975.952	**284.172**	-0.945	2
K	20	---	---	**147.113**	-0.805	1

+2

Seq	#	b: Δ Error	b	y	y: Δ Error	+1
A	1	---	36.526	---	---	20
H	2	---	105.055	1026.014	---	19
T	3	---	155.579	957.484	---	18
F	4	---	229.113	906.961	---	17
S	5	-1.177	**272.629**	**833.426**	-2.093	16
A	6	-1.841	**308.148**	**789.910**	-0.711	15
Q	7	-0.436	**372.177**	**754.392**	-0.337	14
S	8	-0.666	**415.693**	**690.363**	-0.646	13
G	9	---	444.204	**646.847**	-0.276	12
Y	10	---	525.736	**618.336**	1.965	11
R	11	---	603.786	**536.804**	-2.764	10
V	12	0.569	**653.320**	458.754	---	9
P	13	---	701.847	**409.219**	0.140	8
G	14	-0.499	**730.357**	360.693	---	7
P	15	---	778.884	**332.182**	-1.743	6
Q	16	---	842.913	283.656	---	5
P	17	---	891.440	**219.627**	-0.591	4
G	18	---	919.950	171.100	---	3
H	19	---	988.480	142.589	---	2
K	20	---	---	74.060	---	1

+3

Seq	#	b: Δ Error	b	y	y: Δ Error	+1
A	1	---	24.686	---	---	20
H	2	---	70.373	684.345	---	19
T	3	---	104.055	638.659	---	18
F	4	---	153.078	604.976	---	17
S	5	---	182.089	555.953	---	16
A	6	---	205.768	526.943	---	15
Q	7	---	248.454	503.264	---	14
S	8	---	277.465	**460.577**	-0.699	13
G	9	---	296.472	431.567	---	12
Y	10	---	350.826	412.560	---	11
R	11	---	402.860	358.205	---	10
V	12	---	435.883	306.171	---	9
P	13	---	468.234	273.149	---	8
G	14	---	487.241	240.798	---	7
P	15	---	519.592	221.791	---	6
Q	16	---	562.278	189.440	---	5
P	17	---	594.629	146.753	---	4
G	18	---	613.636	114.403	---	3
H	19	---	659.322	95.395	---	2
K	20	---	---	49.709	---	1

AHT@FSAQSGY@RVPGPQPGHK
z = 3+

DCBLD1 pT614, pY621

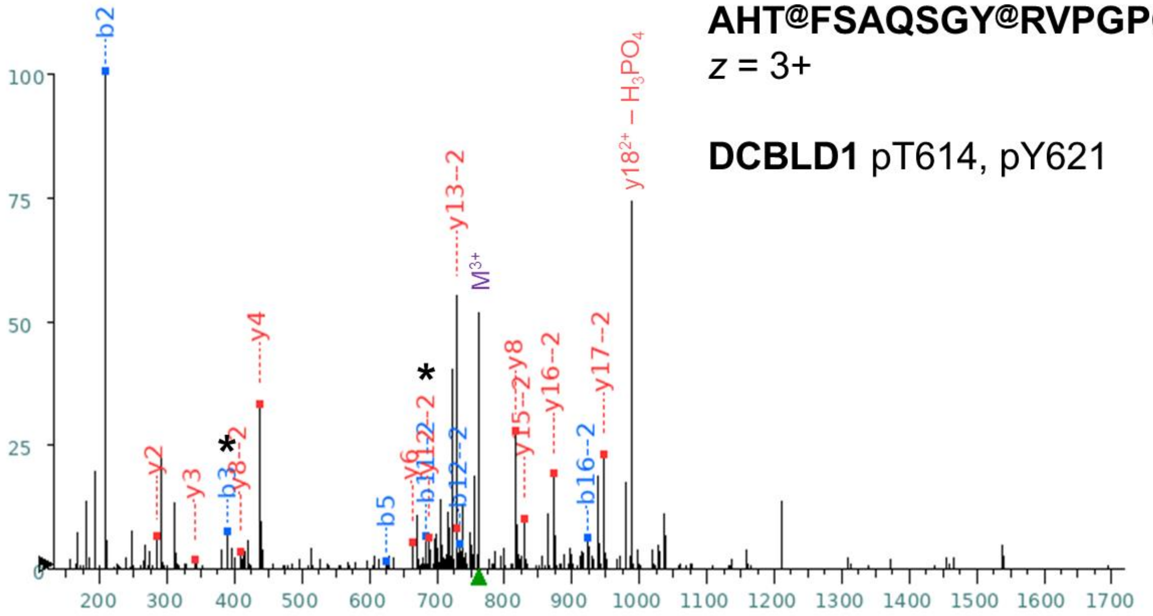

+1

Seq	#	b: Δ Error	b	y	y: Δ Error	+1
A	1	---	72.044	---	---	20
H	2	-0.801	**209.103**	2210.953	---	19
T@	3	-1.723	**390.117**	2073.894	---	18
F	4	---	537.186	1892.880	---	17
S	5	-2.998	**624.218**	1745.812	---	16
A	6	---	695.255	1658.780	---	15
Q	7	---	823.313	1587.743	---	14
S	8	---	910.345	1459.684	---	13
G	9	---	967.367	1372.652	---	12
Y@	10	---	1210.397	1315.631	---	11
R	11	---	1366.498	1072.601	---	10
V	12	---	1465.566	916.500	---	9
P	13	---	1562.619	**817.432**	-0.590	8
G	14	---	1619.640	720.379	---	7
P	15	---	1716.693	**663.357**	0.298	6
Q	16	---	1844.752	566.305	---	5
P	17	---	1941.804	**438.246**	-0.981	4
G	18	---	1998.826	**341.193**	-1.532	3
H	19	---	2135.885	**284.172**	-1.160	2
K	20	---	---	147.113	---	1

+2

Seq	#	b: Δ Error	b	y	y: Δ Error	+1
A	1	---	36.526	---	---	20
H	2	---	105.055	1105.980	---	19
T@	3	---	195.562	1037.451	---	18
F	4	---	269.097	**946.944**	-0.851	17
S	5	---	312.613	**873.410**	-2.010	16
A	6	---	348.131	**829.894**	-0.249	15
Q	7	---	412.160	794.375	---	14
S	8	---	455.676	**730.346**	-0.374	13
G	9	---	484.187	**686.830**	-0.009	12
Y@	10	---	605.702	658.319	---	11
R	11	0.374	**683.752**	536.804	---	10
V	12	-3.101	**733.287**	458.754	---	9
P	13	---	781.813	**409.219**	-1.948	8
G	14	---	810.324	360.693	---	7
P	15	---	858.850	332.182	---	6
Q	16	-0.492	**922.879**	283.656	---	5
P	17	---	971.406	219.627	---	4
G	18	---	999.917	171.100	---	3
H	19	---	1068.446	142.589	---	2
K	20	---	---	74.060	---	1

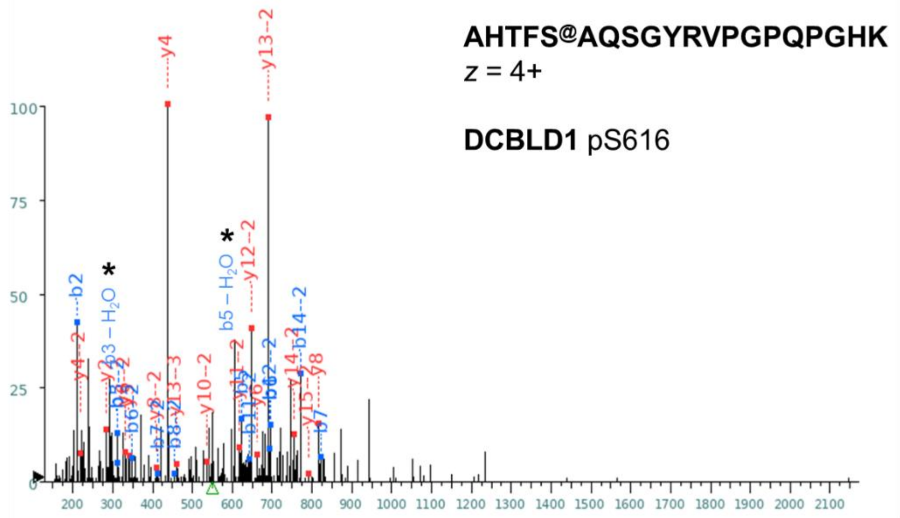

+1

Seq	#	b: Δ Error	b	y	y: Δ Error	+1
A	1	---	72.044	---	---	20
H	2	-1.823	**209.103**	2130.987	---	19
T	3	-1.999	**310.151**	1993.928	---	18
F	4	---	457.219	1892.880	---	17
S®	5	-2.314	**624.218**	1745.812	---	16
A	6	-1.996	**695.255**	1578.814	---	15
Q	7	0.113	**823.313**	1507.776	---	14
S	8	---	910.345	1379.718	---	13
G	9	---	967.367	1292.686	---	12
Y	10	---	1130.430	1235.664	---	11
R	11	---	1286.531	1072.601	---	10
V	12	---	1385.600	916.500	---	9
P	13	---	1482.653	**817.432**	-0.740	8
G	14	---	1539.674	720.379	---	7
P	15	---	1636.727	**663.357**	1.494	6
Q	16	---	1764.785	566.305	---	5
P	17	---	1861.838	**438.246**	-1.678	4
G	18	---	1918.860	**341.193**	-0.906	3
H	19	---	2055.918	**284.172**	-1.697	2
K	20	---	---	147.113	---	1

+2

Seq	#	b: Δ Error	b	y	y: Δ Error	+1
A	1	---	36.526	---	---	20
H	2	---	105.055	1065.997	---	19
T	3	---	155.579	997.468	---	18
F	4	---	229.113	946.944	---	17
S®	5	-1.940	**312.613**	873.410	---	16
A	6	-1.222	**348.131**	**789.910**	1.607	15
Q	7	-0.050	**412.160**	**754.392**	-0.580	14
S	8	-1.368	**455.676**	**690.363**	-1.706	13
G	9	---	484.187	**646.847**	-1.503	12
Y	10	---	565.719	**618.336**	0.978	11
R	11	-1.103	**643.769**	**536.804**	-2.195	10
V	12	-2.736	**693.304**	458.754	---	9
P	13	---	741.830	**409.219**	-2.171	8
G	14	-1.201	**770.341**	360.693	---	7
P	15	---	818.867	**332.182**	-3.397	6
Q	16	---	882.896	283.656	---	5
P	17	---	931.423	**219.627**	-0.522	4
G	18	---	959.933	171.100	---	3
H	19	---	1028.463	142.589	---	2
K	20	---	---	74.060	---	1

+3

Seq	#	b: Δ Error	b	y	y: Δ Error	+1
A	1	---	24.686	---	---	20
H	2	---	70.373	711.000	---	19
T	3	---	104.055	665.314	---	18
F	4	---	153.078	631.632	---	17
S®	5	---	208.744	582.609	---	16
A	6	---	232.423	526.943	---	15
Q	7	---	275.109	503.264	---	14
S	8	---	304.120	**460.577**	-1.096	13
G	9	---	323.127	431.567	---	12
Y	10	---	377.482	412.560	---	11
R	11	---	429.515	358.205	---	10
V	12	---	462.538	306.171	---	9
P	13	---	494.889	273.149	---	8
G	14	---	513.896	240.798	---	7
P	15	---	546.247	221.791	---	6
Q	16	---	588.933	189.440	---	5
P	17	---	621.284	146.753	---	4
G	18	---	640.291	114.403	---	3
H	19	---	685.978	95.395	---	2
K	20	---	---	49.709	---	1

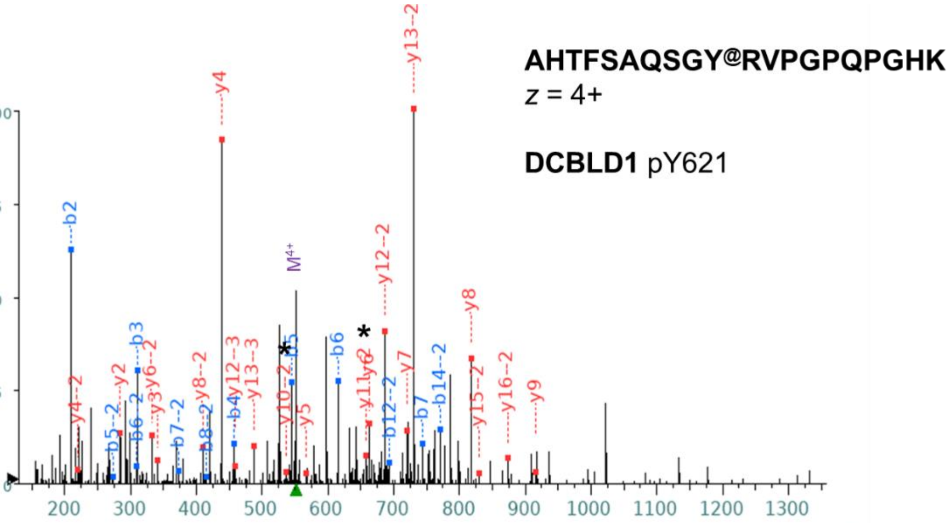

Seq	#	b: Δ Error	b	y	y: Δ Error	+1
			+1			
A	1	---	72.044	---	---	20
H	2	-0.144	209.103	2130.987	---	19
T	3	-0.523	310.151	1993.928	---	18
F	4	-0.407	457.219	1892.880	---	17
S	5	-0.259	544.251	1745.812	---	16
A	6	0.359	615.289	1658.780	---	15
Q	7	-1.241	743.347	1587.743	---	14
S	8	---	830.379	1459.684	---	13
G	9	---	887.401	1372.652	---	12
Y®	10	---	1130.430	1315.631	---	11
R	11	---	1286.531	1072.601	---	10
V	12	---	1385.600	916.500	2.544	9
P	13	---	1482.653	817.432	-0.590	8
G	14	---	1539.674	720.379	0.218	7
P	15	---	1636.727	663.357	-0.254	6
Q	16	---	1764.785	566.305	1.371	5
P	17	---	1861.838	438.246	-0.146	4
G	18	---	1918.860	341.193	2.493	3
H	19	---	2055.918	284.172	-0.838	2
K	20	---	---	147.113	---	1

Seq	#	b: Δ Error	b	y	y: Δ Error	+1
			+2			
A	1	---	36.526	---	---	20
H	2	---	105.055	1065.997	---	19
T	3	---	155.579	997.468	---	18
F	4	---	229.113	946.944	---	17
S	5	-1.737	272.629	873.410	-2.429	16
A	6	6.874	308.148	829.894	-0.837	15
Q	7	1.122	372.177	794.375	---	14
S	8	-1.327	415.693	730.346	0.127	13
G	9	---	444.204	686.830	-0.276	12
Y®	10	---	565.719	658.319	1.087	11
R	11	---	643.769	536.804	2.808	10
V	12	-1.240	693.304	458.754	---	9
P	13	---	741.830	409.219	-1.127	8
G	14	-0.805	770.341	360.693	---	7
P	15	---	818.867	332.182	-0.824	6
Q	16	---	882.896	283.656	---	5
P	17	---	931.423	219.627	-0.661	4
G	18	---	959.933	171.100	---	3
H	19	---	1028.463	142.589	---	2
K	20	---	---	74.060	---	1

Seq	#	b: Δ Error	b	y	y: Δ Error	+1
			+3			
A	1	---	24.686	---	---	20
H	2	---	70.373	711.000	---	19
T	3	---	104.055	665.314	---	18
F	4	---	153.078	631.632	---	17
S	5	---	182.089	582.609	---	16
A	6	---	205.768	553.598	---	15
Q	7	---	248.454	529.919	---	14
S	8	---	277.465	487.233	-0.320	13
G	9	---	296.472	458.222	2.712	12
Y®	10	---	377.482	439.215	---	11
R	11	---	429.515	358.205	---	10
V	12	---	462.538	306.171	---	9
P	13	---	494.889	273.149	---	8
G	14	---	513.896	240.798	---	7
P	15	---	546.247	221.791	---	6
Q	16	---	588.933	189.440	---	5
P	17	---	621.284	146.753	---	4
G	18	---	640.291	114.403	---	3
H	19	---	685.978	95.395	---	2
K	20	---	---	49.709	---	1

7

AHTFSAQSGYR

$z = 3+$

DCBLD1 T614

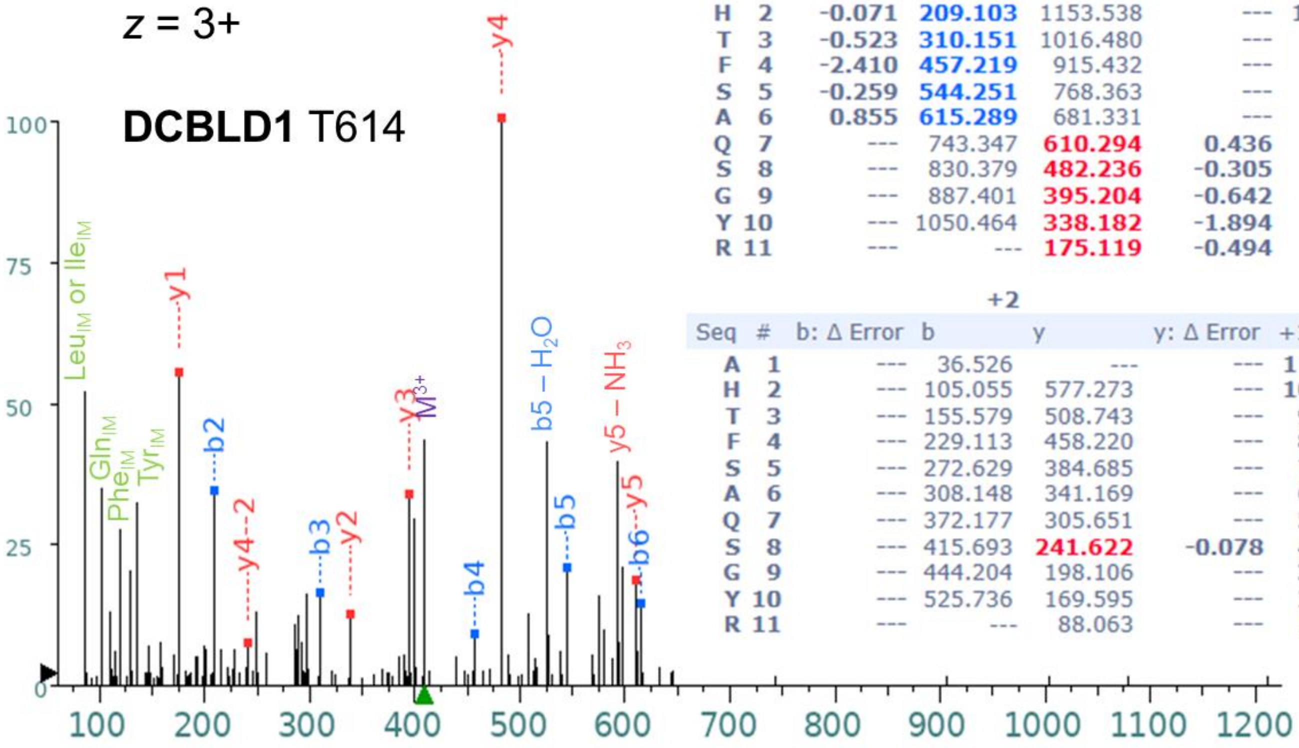

+1

Seq	#	b: Δ Error	b	y	y: Δ Error	+1
A	1	---	72.044	---	---	11
H	2	-0.071	**209.103**	1153.538	---	10
T	3	-0.523	**310.151**	1016.480	---	9
F	4	-2.410	**457.219**	915.432	---	8
S	5	-0.259	**544.251**	768.363	---	7
A	6	0.855	**615.289**	681.331	---	6
Q	7	---	743.347	**610.294**	0.436	5
S	8	---	830.379	**482.236**	-0.305	4
G	9	---	887.401	**395.204**	-0.642	3
Y	10	---	1050.464	**338.182**	-1.894	2
R	11	---	---	**175.119**	-0.494	1

+2

Seq	#	b: Δ Error	b	y	y: Δ Error	+1
A	1	---	36.526	---	---	11
H	2	---	105.055	577.273	---	10
T	3	---	155.579	508.743	---	9
F	4	---	229.113	458.220	---	8
S	5	---	272.629	384.685	---	7
A	6	---	308.148	341.169	---	6
Q	7	---	372.177	305.651	---	5
S	8	---	415.693	**241.622**	-0.078	4
G	9	---	444.204	198.106	---	3
Y	10	---	525.736	169.595	---	2
R	11	---	---	88.063	---	1

AHT@FSAQSGYR

$z = 3+$

DCBLD1 pT614

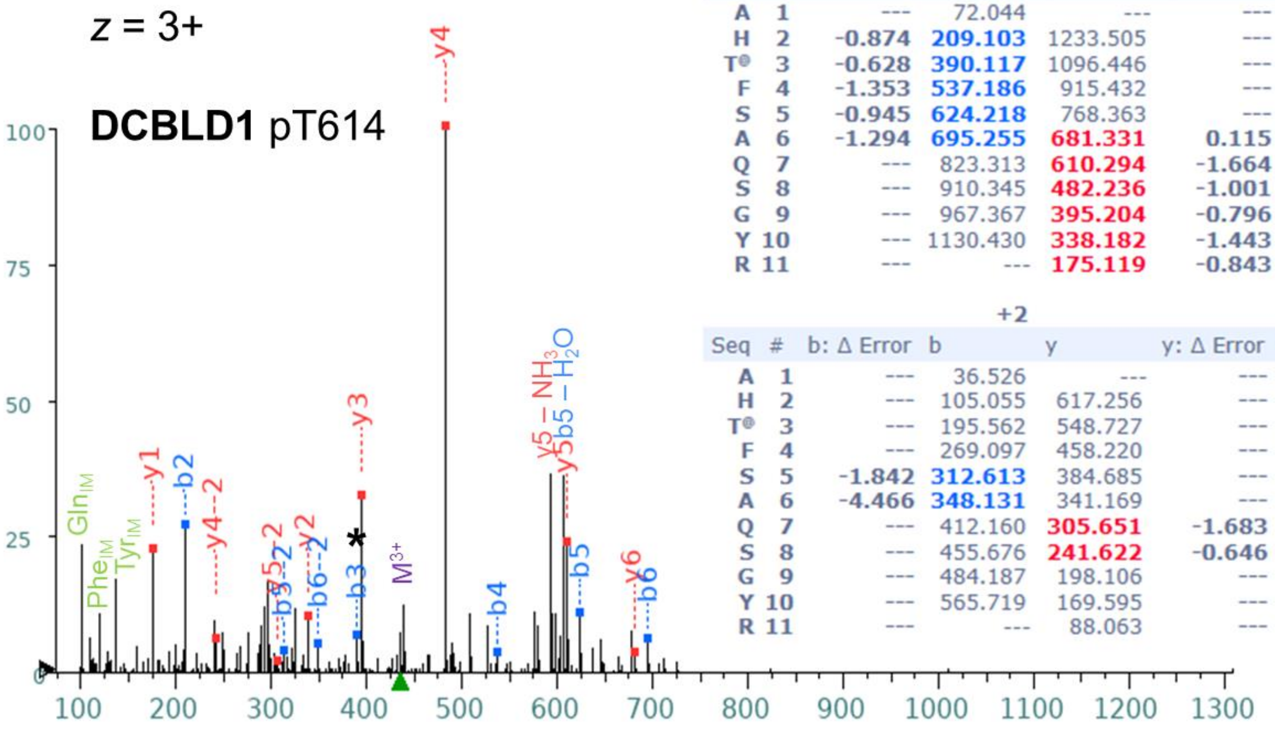

+1

Seq	#	b: Δ Error	b	y	y: Δ Error	+1
A	1	---	72.044	---	---	11
H	2	-0.874	**209.103**	1233.505	---	10
T@	3	-0.628	**390.117**	1096.446	---	9
F	4	-1.353	**537.186**	915.432	---	8
S	5	-0.945	**624.218**	768.363	---	7
A	6	-1.294	**695.255**	**681.331**	0.115	6
Q	7	---	823.313	**610.294**	-1.664	5
S	8	---	910.345	**482.236**	-1.001	4
G	9	---	967.367	**395.204**	-0.796	3
Y	10	---	1130.430	**338.182**	-1.443	2
R	11	---	---	**175.119**	-0.843	1

+2

Seq	#	b: Δ Error	b	y	y: Δ Error	+1
A	1	---	36.526	---	---	11
H	2	---	105.055	617.256	---	10
T@	3	---	195.562	548.727	---	9
F	4	---	269.097	458.220	---	8
S	5	-1.842	**312.613**	384.685	---	7
A	6	-4.466	**348.131**	341.169	---	6
Q	7	---	412.160	**305.651**	-1.683	5
S	8	---	455.676	**241.622**	-0.646	4
G	9	---	484.187	198.106	---	3
Y	10	---	565.719	169.595	---	2
R	11	---	---	88.063	---	1

LDLITSDM*ADYQQPLM*IGTGTVTRK
z = 3+

DCBLD1 S535, Y540

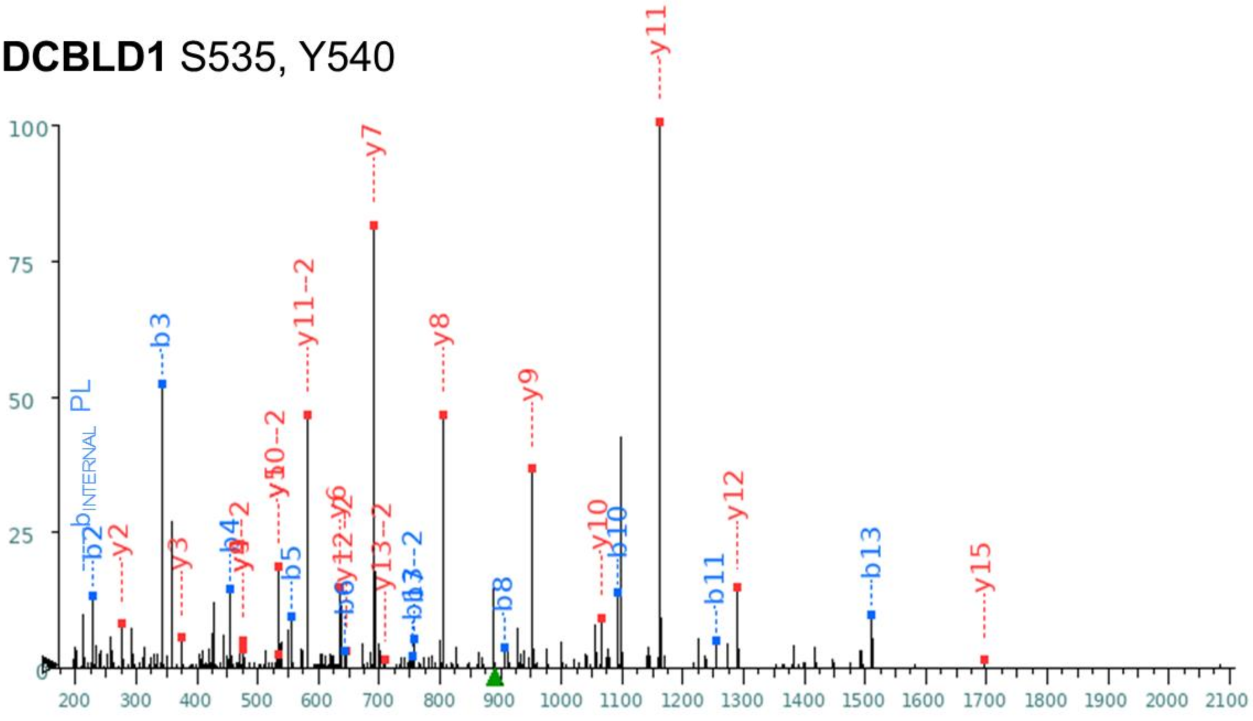

+1

Seq	#	b: Δ Error	b	y	y: Δ Error	+1
L	1	---	114.091	---	---	24
D	2	-1.052	**229.118**	2558.211	---	23
L	3	-0.937	**342.202**	2443.184	---	22
I	4	-0.611	**455.286**	2330.100	---	21
T	5	-0.737	**556.334**	2217.016	---	20
S	6	1.947	**643.366**	2115.969	---	19
D	7	0.893	**758.393**	2028.937	---	18
M*	8	-2.689	**905.428**	1913.910	---	17
A	9	---	976.466	1766.874	---	16
D	10	-0.078	**1091.493**	**1695.837**	-0.510	15
Y	11	0.634	**1254.556**	1580.810	---	14
Q	12	---	1382.614	1417.747	---	13
Q	13	0.063	**1510.673**	**1289.688**	-1.204	12
P	14	---	1607.726	**1161.630**	-0.720	11
L	15	---	1720.810	**1064.577**	-0.184	10
M*	16	---	1867.845	**951.493**	-0.508	9
I	17	---	1980.929	**804.457**	-0.754	8
G	18	---	2037.951	**691.373**	-0.585	7
T	19	---	2138.998	**634.352**	0.388	6
G	20	---	2196.020	**533.304**	-0.550	5
T	21	---	2297.068	**476.283**	-0.338	4
V	22	---	2396.136	**375.235**	-0.728	3
T	23	---	2497.184	**276.167**	0.424	2
R	24	---	---	175.119	---	1

+2

Seq	#	b: Δ Error	b	y	y: Δ Error	+1
L	1	---	57.549	---	---	24
D	2	---	115.063	1279.609	---	23
L	3	---	171.605	1222.096	---	22
I	4	---	228.147	1165.554	---	21
T	5	---	278.671	1109.012	---	20
S	6	---	322.187	1058.488	---	19
D	7	---	379.700	1014.972	---	18
M*	8	---	453.218	957.458	---	17
A	9	---	488.736	883.941	---	16
D	10	---	546.250	848.422	---	15
Y	11	---	627.782	790.909	---	14
Q	12	---	691.811	**709.377**	0.500	13
Q	13	3.325	**755.840**	**645.348**	1.767	12
P	14	---	804.367	**581.318**	-0.468	11
L	15	---	860.909	**532.792**	-1.170	10
M*	16	---	934.426	**476.250**	-1.033	9
I	17	---	990.968	402.732	---	8
G	18	---	1019.479	346.190	---	7
T	19	---	1070.003	317.680	---	6
G	20	---	1098.514	267.156	---	5
T	21	---	1149.037	238.645	---	4
V	22	---	1198.572	188.121	---	3
T	23	---	1249.095	138.587	---	2
R	24	---	---	88.063	---	1

LDLITS@DM*ADYQQPLM*IGTGTVTRK
z = 3+

DCBLD1 pS535

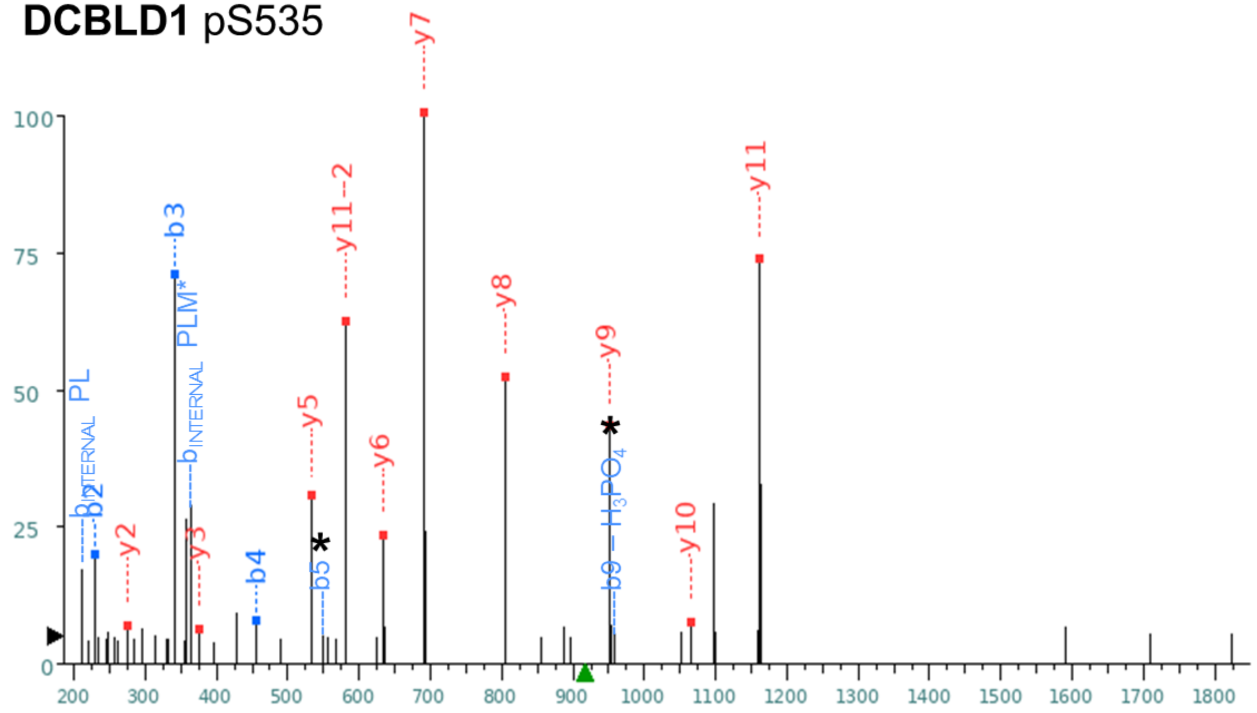

+1

Seq	#	b: Δ Error	b	y	y: Δ Error	+1
L	1	---	114.091	---	---	24
D	2	0.080	**229.118**	2638.178	---	23
L	3	-1.472	**342.202**	2523.151	---	22
I	4	-1.549	**455.286**	2410.067	---	21
T	5	---	556.334	2296.983	---	20
S@	6	---	723.332	2195.935	---	19
D	7	---	838.359	2028.937	---	18
M*	8	---	985.395	1913.910	---	17
A	9	---	1056.432	1766.874	---	16
D	10	---	1171.459	1695.837	---	15
Y	11	---	1334.522	1580.810	---	14
Q	12	---	1462.581	1417.747	---	13
Q	13	---	1590.639	1289.688	---	12
P	14	---	1687.692	**1161.630**	-2.086	11
L	15	---	1800.776	**1064.577**	-0.299	10
M*	16	---	1947.812	**951.493**	-3.138	9
I	17	---	2060.896	**804.457**	-0.678	8
G	18	---	2117.917	**691.373**	-1.291	7
T	19	---	2218.965	**634.352**	2.120	6
G	20	---	2275.986	**533.304**	4.143	5
T	21	---	2377.034	476.283	---	4
V	22	---	2476.102	**375.235**	-1.948	3
T	23	---	2577.150	**276.167**	0.534	2
R	24	---	---	175.119	---	1

+2

Seq	#	b: Δ Error	b	y	y: Δ Error	+1
L	1	---	57.549	---	---	24
D	2	---	115.063	1319.592	---	23
L	3	---	171.605	1262.079	---	22
I	4	---	228.147	1205.537	---	21
T	5	---	278.671	1148.995	---	20
S@	6	---	362.170	1098.471	---	19
D	7	---	419.683	1014.972	---	18
M*	8	---	493.201	957.458	---	17
A	9	---	528.720	883.941	---	16
D	10	---	586.233	848.422	---	15
Y	11	---	667.765	790.909	---	14
Q	12	---	731.794	709.377	---	13
Q	13	---	795.823	645.348	---	12
P	14	---	844.350	**581.318**	0.372	11
L	15	---	900.892	532.792	---	10
M*	16	---	974.409	476.250	---	9
I	17	---	1030.951	402.732	---	8
G	18	---	1059.462	346.190	---	7
T	19	---	1109.986	317.680	---	6
G	20	---	1138.497	267.156	---	5
T	21	---	1189.021	238.645	---	4
V	22	---	1238.555	188.121	---	3
T	23	---	1289.079	138.587	---	2
R	24	---	---	88.063	---	1

LDLITSDMADY@QQPLMIGTGTVTRK
z = 3+

DCBLD1 pY540

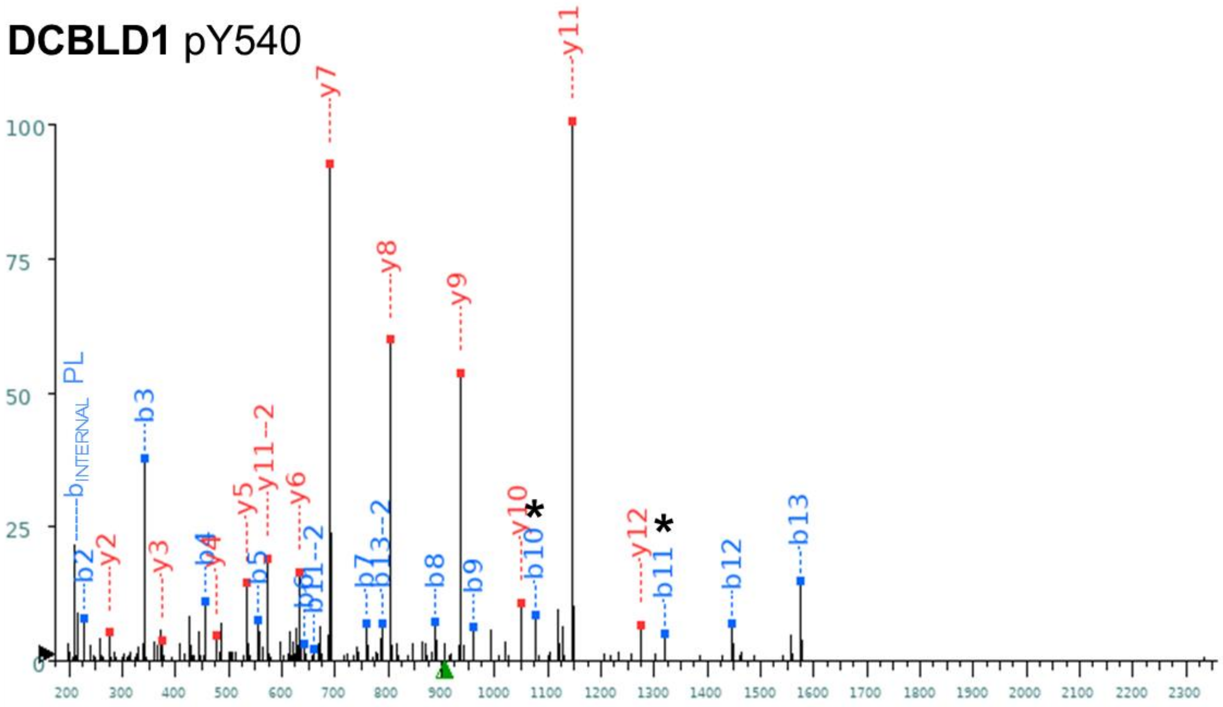

+1

Seq	#	b: Δ Error	b	y	y: Δ Error	+1
L	1	---	114.091	---	---	24
D	2	-0.719	**229.118**	2606.188	---	23
L	3	-0.313	**342.202**	2491.161	---	22
I	4	4.349	**455.286**	2378.077	---	21
T	5	0.250	**556.334**	2264.993	---	20
S	6	-2.796	**643.366**	2163.945	---	19
D	7	1.295	**758.393**	2076.913	---	18
M	8	3.005	**889.434**	1961.886	---	17
A	9	2.016	**960.471**	1830.846	---	16
D	10	0.300	**1075.498**	1759.808	---	15
Y@	11	-0.863	**1318.527**	1644.782	---	14
Q	12	0.743	**1446.586**	1401.752	---	13
Q	13	-1.632	**1574.644**	**1273.693**	-1.857	12
P	14	---	1671.697	**1145.635**	-0.907	11
L	15	---	1784.781	**1048.582**	-1.079	10
M	16	---	1915.822	**935.498**	-1.124	9
I	17	---	2028.906	**804.457**	-1.436	8
G	18	---	2085.927	**691.373**	-1.556	7
T	19	---	2186.975	**634.352**	-0.766	6
G	20	---	2243.996	**533.304**	-1.465	5
T	21	---	2345.044	**476.283**	0.366	4
V	22	---	2444.112	**375.235**	1.305	3
T	23	---	2545.160	**276.167**	-1.565	2
R	24	---	---	175.119	---	1

+2

Seq	#	b: Δ Error	b	y	y: Δ Error	+1
L	1	---	57.549	---	---	24
D	2	---	115.063	1303.598	---	23
L	3	---	171.605	1246.084	---	22
I	4	---	228.147	1189.542	---	21
T	5	---	278.671	1133.000	---	20
S	6	---	322.187	1082.476	---	19
D	7	---	379.700	1038.960	---	18
M	8	---	445.220	981.447	---	17
A	9	---	480.739	915.926	---	16
D	10	---	538.252	880.408	---	15
Y@	11	-0.734	**659.767**	822.894	---	14
Q	12	---	723.797	701.380	---	13
Q	13	-2.685	**787.826**	637.350	---	12
P	14	---	836.352	**573.321**	-0.864	11
L	15	---	892.894	524.795	---	10
M	16	---	958.415	468.253	---	9
I	17	---	1014.957	402.732	---	8
G	18	---	1043.467	346.190	---	7
T	19	---	1093.991	317.680	---	6
G	20	---	1122.502	267.156	---	5
T	21	---	1173.026	238.645	---	4
V	22	---	1222.560	188.121	---	3
T	23	---	1273.084	138.587	---	2
R	24	---	---	88.063	---	1

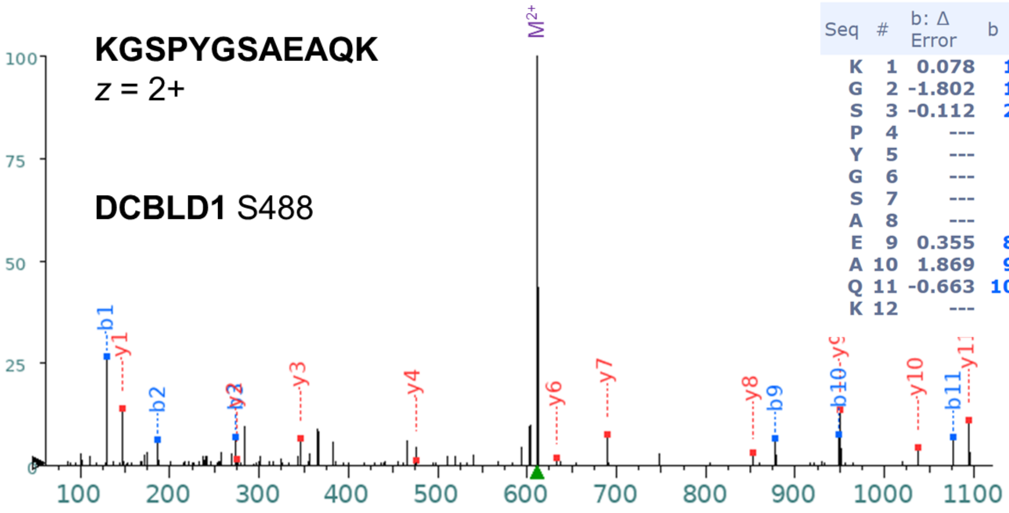

Seq	#	b: Δ Error	b	y	y: Δ Error	+1
K	1	0.078	129.102	---	---	12
G	2	-1.802	186.124	1094.511	-2.610	11
S	3	-0.112	273.156	1037.490	0.992	10
P	4	---	370.208	950.458	-1.116	9
Y	5	---	533.272	853.405	-1.996	8
G	6	---	590.293	690.342	-1.709	7
S	7	---	677.325	633.320	-2.088	6
A	8	---	748.362	546.288	---	5
E	9	0.355	877.405	475.251	2.011	4
A	10	1.869	948.442	346.208	-1.850	3
Q	11	-0.663	1076.501	275.171	-0.537	2
K	12	---	---	147.113	-0.908	1

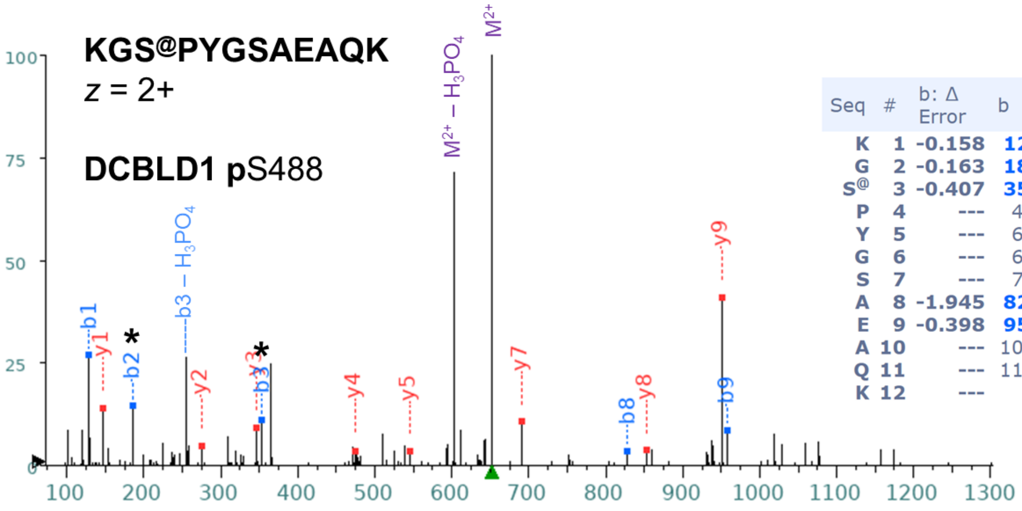

Seq	#	b: Δ Error	b	y	y: Δ Error	+1
K	1	-0.158	129.102	---	---	12
G	2	-0.163	186.124	1174.478	---	11
S@	3	-0.407	353.122	1117.456	---	10
P	4	---	450.175	950.458	-0.282	9
Y	5	---	613.238	853.405	-0.279	8
G	6	---	670.260	690.342	-0.206	7
S	7	---	757.292	633.320	---	6
A	8	-1.945	828.329	546.288	0.010	5
E	9	-0.398	957.371	475.251	0.534	4
A	10	---	1028.408	346.208	-1.233	3
Q	11	---	1156.467	275.171	-1.757	2
K	12	---	---	147.113	-0.286	1

HSLSSGGFSPVAGVGAQDGDYQRPHSAQPADRGYDRPK
z = 5+

DCBLD1 S636, S640, S657, Y665

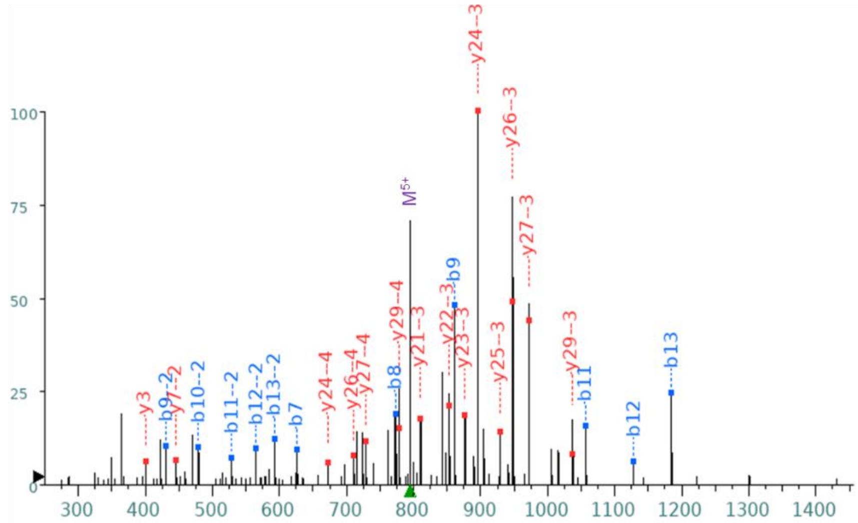

+1

Seq	#	b: Δ Error	b	y	y: Δ Error	+1
H	1	---	138.066	---	---	38
S	2	---	225.098	3830.823	---	37
L	3	---	338.182	3743.791	---	36
S	4	---	425.214	3630.707	---	35
S	5	---	512.246	3543.675	---	34
G	6	---	569.268	3456.643	---	33
G	7	0.847	**626.289**	3399.621	---	32
F	8	-2.778	**773.358**	3342.600	---	31
S	9	-0.635	**860.390**	3195.531	---	30
P	10	---	957.442	3108.499	---	29
V	11	-2.792	**1056.511**	3011.447	---	28
A	12	-1.105	**1127.548**	2912.378	---	27
G	13	-0.107	**1184.569**	2841.341	---	26
V	14	---	1283.638	2784.320	---	25
G	15	---	1340.659	2685.251	---	24
A	16	---	1411.696	2628.230	---	23
Q	17	---	1539.755	2557.193	---	22
D	18	---	1654.782	2429.134	---	21
G	19	---	1711.803	2314.107	---	20
D	20	---	1826.830	2257.086	---	19
Y	21	---	1989.894	2142.059	---	18
Q	22	---	2117.952	1978.995	---	17
R	23	---	2274.053	1850.937	---	16
P	24	---	2371.106	1694.836	---	15
H	25	---	2508.165	1597.783	---	14
S	26	---	2595.197	1460.724	---	13
A	27	---	2666.234	1373.692	---	12
Q	28	---	2794.293	1302.655	---	11
P	29	---	2891.346	1174.596	---	10
A	30	---	2962.383	1077.544	---	9
D	31	---	3077.410	1006.506	---	8
R	32	---	3233.511	891.480	---	7
G	33	---	3290.532	735.378	---	6
Y	34	---	3453.596	678.357	---	5
D	35	---	3568.622	515.294	---	4
R	36	---	3724.724	**400.267**	-0.194	3
P	37	---	3821.776	244.166	---	2
K	38	---	---	147.113	---	1

+2

Seq	#	b: Δ Error	b	y	y: Δ Error	+1
H	1	---	69.537	---	---	38
S	2	---	113.053	1915.915	---	37
L	3	---	169.595	1872.399	---	36
S	4	---	213.111	1815.857	---	35
S	5	---	256.627	1772.341	---	34
G	6	---	285.138	1728.825	---	33
G	7	---	313.648	1700.314	---	32
F	8	---	387.182	1671.804	---	31
S	9	-3.980	**430.698**	1598.269	---	30
P	10	0.022	**479.225**	1554.753	---	29
V	11	-0.205	**528.759**	1506.227	---	28
A	12	-0.845	**564.278**	1456.693	---	27
G	13	-1.198	**592.788**	1421.174	---	26
V	14	---	642.323	1392.663	---	25
G	15	---	670.833	1343.129	---	24
A	16	---	706.352	1314.619	---	23
Q	17	---	770.381	1279.100	---	22
D	18	---	827.895	1215.071	---	21
G	19	---	856.405	1157.557	---	20
D	20	---	913.919	1129.046	---	19
Y	21	---	995.450	1071.533	---	18
Q	22	---	1059.480	990.001	---	17
R	23	---	1137.530	925.972	---	16
P	24	---	1186.057	847.922	---	15
H	25	---	1254.586	799.395	---	14
S	26	---	1298.102	730.866	---	13
A	27	---	1333.621	687.350	---	12
Q	28	---	1397.650	651.831	---	11
P	29	---	1446.176	587.802	---	10
A	30	---	1481.695	539.275	---	9
D	31	---	1539.208	503.757	---	8
R	32	---	1617.259	**446.243**	-0.531	7
G	33	---	1645.770	368.193	---	6
Y	34	---	1727.301	339.682	---	5
D	35	---	1784.815	258.150	---	4
R	36	---	1862.865	200.637	---	3
P	37	---	1911.392	122.586	---	2
K	38	---	---	74.060	---	1

+3

Seq	#	b: Δ Error	b	y	y: Δ Error	+1
H	1	---	46.694	---	---	38
S	2	---	75.704	1277.613	---	37
L	3	---	113.399	1248.602	---	36
S	4	---	142.410	1210.907	---	35
S	5	---	171.420	1181.896	---	34
G	6	---	190.427	1152.886	---	33
G	7	---	209.435	1133.879	---	32
F	8	---	258.457	1114.871	---	31
S	9	---	287.468	1065.849	---	30
P	10	---	319.819	**1036.838**	0.135	29
V	11	---	352.842	1004.487	---	28
A	12	---	376.521	**971.464**	-0.162	27
G	13	---	395.528	**947.785**	-0.852	26
V	14	---	428.551	**928.778**	-0.066	25
G	15	---	447.558	**895.755**	-0.979	24
A	16	---	471.237	**876.748**	0.198	23
Q	17	---	513.923	**853.069**	-2.490	22
D	18	---	552.266	**810.383**	-1.598	21
G	19	---	571.273	772.041	---	20
D	20	---	609.615	753.033	---	19
Y	21	---	663.969	714.691	---	18
Q	22	---	706.656	660.337	---	17
R	23	---	758.689	617.650	---	16
P	24	---	791.040	565.617	---	15
H	25	---	836.727	533.266	---	14
S	26	---	865.737	487.580	---	13
A	27	---	889.416	458.569	---	12
Q	28	---	932.102	434.890	---	11
P	29	---	964.453	392.204	---	10
A	30	---	988.132	359.853	---	9
D	31	---	1026.475	336.174	---	8
R	32	---	1078.508	297.831	---	7
G	33	---	1097.516	245.798	---	6
Y	34	---	1151.870	226.791	---	5
D	35	---	1190.212	172.436	---	4
R	36	---	1242.246	134.094	---	3
P	37	---	1274.597	82.060	---	2
K	38	---	---	49.709	---	1

+4

Seq	#	b: Δ Error	b	y	y: Δ Error	+1
H	1	---	35.272	---	---	38
S	2	---	57.030	958.461	---	37
L	3	---	85.301	936.703	---	36
S	4	---	107.059	908.432	---	35
S	5	---	128.817	886.674	---	34
G	6	---	143.072	864.916	---	33
G	7	---	157.328	850.661	---	32
F	8	---	194.095	836.405	---	31
S	9	---	215.853	799.638	---	30
P	10	---	240.116	**777.880**	1.406	29
V	11	---	264.883	753.617	---	28
A	12	---	282.642	**728.850**	0.189	27
G	13	---	296.898	**711.091**	1.140	26
V	14	---	321.665	696.835	---	25
G	15	---	335.920	**672.068**	-0.144	24
A	16	---	353.680	657.813	---	23
Q	17	---	385.694	640.054	---	22
D	18	---	414.451	608.039	---	21
G	19	---	428.706	579.282	---	20
D	20	---	457.463	565.027	---	19
Y	21	---	498.229	536.270	---	18
Q	22	---	530.244	495.504	---	17
R	23	---	569.269	463.490	---	16
P	24	---	593.532	424.464	---	15
H	25	---	627.797	400.201	---	14
S	26	---	649.555	365.936	---	13
A	27	---	667.314	344.178	---	12
Q	28	---	699.329	326.419	---	11
P	29	---	723.592	294.405	---	10
A	30	---	741.351	270.141	---	9
D	31	---	770.108	252.382	---	8
R	32	---	809.133	223.625	---	7
G	33	---	823.389	184.600	---	6
Y	34	---	864.154	170.345	---	5
D	35	---	892.911	129.579	---	4
R	36	---	931.936	100.822	---	3
P	37	---	956.200	61.797	---	2
K	38	---	---	37.534	---	1

HSLSS@GGFSPVAGVGAQDGDYQRPHSAQPADRGY@DRPK
z = 5+

DCBLD1 pS636, pY665

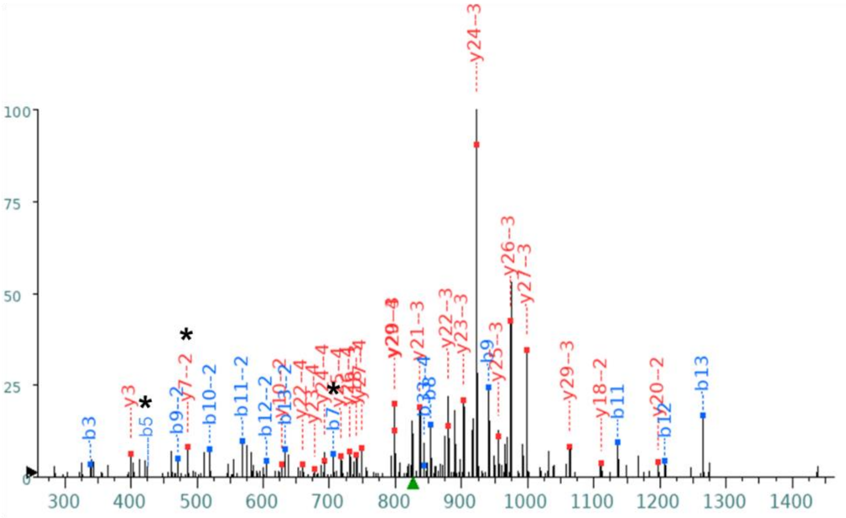

+1

Seq	#	b: Δ Error	b	y	y: Δ Error	+1
H	1	---	138.066	---	---	38
S	2	---	225.098	3990.756	---	37
L	3	-0.177	338.182	3903.724	---	36
S	4	---	425.214	3790.640	---	35
S●	5	---	592.213	3703.607	---	34
G	6	---	649.234	3536.609	---	33
G	7	4.091	706.256	3479.588	---	32
F	8	-1.328	853.324	3422.566	---	31
S	9	-1.708	940.356	3275.498	---	30
P	10	---	1037.409	3188.466	---	29
V	11	-0.789	1136.477	3091.413	---	28
A	12	2.084	1207.514	2992.345	---	27
G	13	-1.758	1264.536	2921.307	---	26
V	14	---	1363.604	2864.286	---	25
G	15	---	1420.626	2765.218	---	24
A	16	---	1491.663	2708.196	---	23
Q	17	---	1619.721	2637.159	---	22
D	18	---	1734.748	2509.100	---	21
G	19	---	1791.770	2394.073	---	20
D	20	---	1906.797	2337.052	---	19
Y	21	---	2069.860	2222.025	---	18
Q	22	---	2197.919	2058.962	---	17
R	23	---	2354.020	1930.903	---	16
P	24	---	2451.072	1774.802	---	15
H	25	---	2588.131	1677.749	---	14
S	26	---	2675.163	1540.690	---	13
A	27	---	2746.201	1453.658	---	12
Q	28	---	2874.259	1382.621	---	11
P	29	---	2971.312	1254.563	---	10
A	30	---	3042.349	1157.510	---	9
D	31	---	3157.376	1086.473	---	8
R	32	---	3313.477	971.446	---	7
G	33	---	3370.499	815.345	---	6
Y●	34	---	3613.528	758.323	---	5
D	35	---	3728.555	515.294	---	4
R	36	---	3884.656	400.267	-1.872	3
P	37	---	3981.709	244.166	---	2
K	38	---	---	147.113	---	1

+2

Seq	#	b: Δ Error	b	y	y: Δ Error	+1
H	1	---	69.537	---	---	38
S	2	---	113.053	1995.881	---	37
L	3	---	169.595	1952.365	---	36
S	4	---	213.111	1895.823	---	35
S●	5	---	296.610	1852.307	---	34
G	6	---	325.121	1768.808	---	33
G	7	---	353.631	1740.297	---	32
F	8	---	427.166	1711.787	---	31
S	9	1.392	470.682	1638.253	---	30
P	10	-1.352	519.208	1594.737	---	29
V	11	0.005	568.742	1546.210	---	28
A	12	0.506	604.261	1496.676	---	27
G	13	0.018	632.772	1461.157	---	26
V	14	---	682.306	1432.647	---	25
G	15	---	710.816	1383.112	---	24
A	16	---	746.335	1354.602	---	23
Q	17	---	810.364	1319.083	---	22
D	18	---	867.878	1255.054	---	21
G	19	---	896.389	1197.540	-2.023	20
D	20	---	953.902	1169.030	---	19
Y	21	---	1035.434	1111.516	-1.600	18
Q	22	---	1099.463	1029.985	---	17
R	23	---	1177.514	965.955	---	16
P	24	---	1226.040	887.905	---	15
H	25	---	1294.569	839.378	---	14
S	26	---	1338.085	770.849	---	13
A	27	---	1373.604	727.333	---	12
Q	28	---	1437.633	691.814	---	11
P	29	---	1486.160	627.785	0.481	10
A	30	---	1521.678	579.259	---	9
D	31	---	1579.192	543.740	---	8
R	32	---	1657.242	486.227	-1.200	7
G	33	---	1685.753	408.176	---	6
Y●	34	---	1807.268	379.665	---	5
D	35	---	1864.781	258.150	---	4
R	36	---	1942.832	200.637	---	3
P	37	---	1991.358	122.586	---	2
K	38	---	---	74.060	---	1

+3

Seq	#	b: Δ Error	b	y	y: Δ Error	+1
H	1	---	46.694	---	---	38
S	2	---	75.704	1330.923	---	37
L	3	---	113.399	1301.913	---	36
S	4	---	142.410	1264.218	---	35
S●	5	---	198.076	1235.207	---	34
G	6	---	217.083	1179.541	---	33
G	7	---	236.090	1160.534	---	32
F	8	---	285.113	1141.527	---	31
S	9	---	314.124	1092.504	---	30
P	10	---	346.474	1063.493	2.152	29
V	11	---	379.497	1031.143	---	28
A	12	---	403.176	998.120	-0.634	27
G	13	---	422.183	974.441	-1.128	26
V	14	---	455.206	955.434	-3.053	25
G	15	---	474.213	922.411	0.916	24
A	16	---	497.892	903.404	-0.401	23
Q	17	---	540.579	879.725	-3.301	22
D	18	---	578.921	837.038	-1.677	21
G	19	---	597.928	798.696	1.412	20
D	20	---	636.270	779.689	---	19
Y	21	---	690.625	741.347	0.344	18
Q	22	---	733.311	686.992	---	17
R	23	---	785.345	644.306	---	16
P	24	---	817.696	592.272	---	15
H	25	---	863.382	559.921	---	14
S	26	---	892.393	514.235	---	13
A	27	---	916.072	485.224	---	12
Q	28	---	958.758	461.545	---	11
P	29	---	991.109	418.859	---	10
A	30	---	1014.788	386.508	---	9
D	31	---	1053.130	362.829	---	8
R	32	---	1105.164	324.487	---	7
G	33	---	1124.171	272.453	---	6
Y●	34	---	1205.181	253.446	---	5
D	35	---	1243.523	172.436	---	4
R	36	---	1295.557	134.094	---	3
P	37	---	1327.908	82.060	---	2
K	38	---	---	49.709	---	1

+4

Seq	#	b: Δ Error	b	y	y: Δ Error	+1
H	1	---	35.272	---	---	38
S	2	---	57.030	998.444	---	37
L	3	---	85.301	976.686	---	36
S	4	---	107.059	948.415	---	35
S●	5	---	148.809	926.657	---	34
G	6	---	163.064	884.908	---	33
G	7	---	177.319	870.652	---	32
F	8	---	214.086	856.397	---	31
S	9	---	235.844	819.630	---	30
P	10	---	260.108	797.872	0.905	29
V	11	---	284.875	773.609	---	28
A	12	---	302.634	748.842	-0.475	27
G	13	---	316.889	731.082	-0.569	26
V	14	---	341.657	716.827	-3.227	25
G	15	---	355.912	692.060	3.204	24
A	16	---	373.671	677.804	-0.609	23
Q	17	---	405.686	660.045	-2.380	22
D	18	---	434.443	628.031	---	21
G	19	---	448.698	599.274	---	20
D	20	---	477.455	585.018	---	19
Y	21	---	518.220	556.262	---	18
Q	22	---	550.235	515.496	---	17
R	23	---	589.260	483.481	---	16
P	24	---	613.524	444.456	---	15
H	25	---	647.788	420.193	---	14
S	26	---	669.546	385.928	---	13
A	27	---	687.306	364.170	---	12
Q	28	---	719.320	346.411	---	11
P	29	---	743.583	314.396	---	10
A	30	---	761.343	290.133	---	9
D	31	---	790.099	272.374	---	8
R	32	---	829.125	243.617	---	7
G	33	2.146	843.380	204.592	---	6
Y●	34	---	904.138	190.336	---	5
D	35	---	932.894	129.579	---	4
R	36	---	971.920	100.822	---	3
P	37	---	996.183	61.797	---	2
K	38	---	---	37.534	---	1

HSLSSGGFS@PVAGVGAQDGDYQRPHSAQPADRGY@DRPK
z = 5+

DCBLD1 pS640, pY665

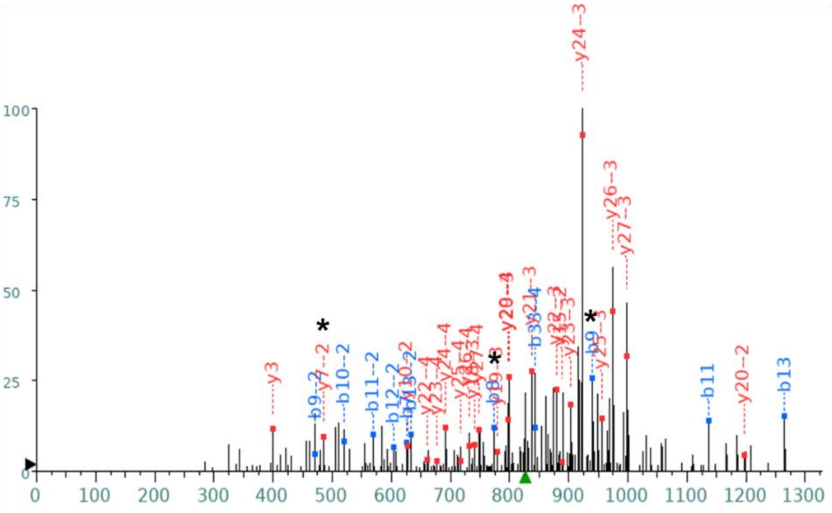

+1

Seq	#	b: Δ Error	b	y	y: Δ Error	+1
H	1	---	138.066	---	---	38
S	2	---	225.098	3990.756	---	37
L	3	---	338.182	3903.724	---	36
S	4	---	425.214	3790.640	---	35
S	5	---	512.246	3703.607	---	34
G	6	---	569.268	3616.575	---	33
G	7	1.821	**626.289**	3559.554	---	32
F	8	1.957	**773.358**	3502.533	---	31
S@	9	-0.475	**940.356**	3355.464	---	30
P	10	---	1037.409	3188.466	---	29
V	11	0.715	**1136.477**	3091.413	---	28
A	12	---	1207.514	2992.345	---	27
G	13	-1.758	**1264.536**	2921.307	---	26
V	14	---	1363.604	2864.286	---	25
G	15	---	1420.626	2765.218	---	24
A	16	---	1491.663	2708.196	---	23
Q	17	---	1619.721	2637.159	---	22
D	18	---	1734.748	2509.100	---	21
G	19	---	1791.770	2394.073	---	20
D	20	---	1906.797	2337.052	---	19
Y	21	---	2069.860	2222.025	---	18
Q	22	---	2197.919	2058.962	---	17
R	23	---	2354.020	1930.903	---	16
P	24	---	2451.072	1774.802	---	15
H	25	---	2588.131	1677.749	---	14
S	26	---	2675.163	1540.690	---	13
A	27	---	2746.201	1453.658	---	12
Q	28	---	2874.259	1382.621	---	11
P	29	---	2971.312	1254.563	---	10
A	30	---	3042.349	1157.510	---	9
D	31	---	3157.376	1086.473	---	8
R	32	---	3313.477	971.446	---	7
G	33	---	3370.499	815.345	---	6
Y@	34	---	3613.528	758.323	---	5
D	35	---	3728.555	515.294	---	4
R	36	---	3884.656	**400.267**	-2.024	3
P	37	---	3981.709	244.166	---	2
K	38	---	---	147.113	---	1

+2

Seq	#	b: Δ Error	b	y	y: Δ Error	+1
H	1	---	69.537	---	---	38
S	2	---	113.053	1995.881	---	37
L	3	---	169.595	1952.365	---	36
S	4	---	213.111	1895.823	---	35
S	5	---	256.627	1852.307	---	34
G	6	---	285.138	1808.791	---	33
G	7	---	313.648	1780.281	---	32
F	8	---	387.182	1751.770	---	31
S@	9	4.180	**470.682**	1678.236	---	30
P	10	0.176	**519.208**	1594.737	---	29
V	11	1.830	**568.742**	1546.210	---	28
A	12	1.920	**604.261**	1496.676	---	27
G	13	-0.754	**632.772**	1461.157	---	26
V	14	---	682.306	1432.647	---	25
G	15	---	710.816	1383.112	---	24
A	16	---	746.335	1354.602	---	23
Q	17	---	810.364	1319.083	---	22
D	18	---	867.878	1255.054	---	21
G	19	---	896.389	**1197.540**	-0.392	20
D	20	---	953.902	1169.030	---	19
Y	21	---	1035.434	1111.516	---	18
Q	22	---	1099.463	1029.985	---	17
R	23	---	1177.514	965.955	---	16
P	24	---	1226.040	**887.905**	2.187	15
H	25	---	1294.569	839.378	---	14
S	26	---	1338.085	770.849	---	13
A	27	---	1373.604	727.333	---	12
Q	28	---	1437.633	691.814	---	11
P	29	---	1486.160	**627.785**	0.773	10
A	30	---	1521.678	579.259	---	9
D	31	---	1579.192	543.740	---	8
R	32	---	1657.242	**486.227**	2.503	7
G	33	---	1685.753	408.176	---	6
Y@	34	---	1807.268	379.665	---	5
D	35	---	1864.781	258.150	---	4
R	36	---	1942.832	200.637	---	3
P	37	---	1991.358	122.586	---	2
K	38	---	---	74.060	---	1

+3

Seq	#	b: Δ Error	b	y	y: Δ Error	+1
H	1	---	46.694	---	---	38
S	2	---	75.704	1330.923	---	37
L	3	---	113.399	1301.913	---	36
S	4	---	142.410	1264.218	---	35
S	5	---	171.420	1235.207	---	34
G	6	---	190.427	1206.197	---	33
G	7	---	209.435	1187.190	---	32
F	8	---	258.457	1168.182	---	31
S@	9	---	314.124	1119.160	---	30
P	10	---	346.474	1063.493	---	29
V	11	---	379.497	1031.143	---	28
A	12	---	403.176	**998.120**	-0.084	27
G	13	---	422.183	**974.441**	-1.128	26
V	14	---	455.206	**955.434**	-0.881	25
G	15	---	474.213	**922.411**	2.371	24
A	16	---	497.892	**903.404**	0.072	23
Q	17	---	540.579	**879.725**	-2.677	22
D	18	---	578.921	**837.038**	-0.219	21
G	19	---	597.928	**798.696**	2.558	20
D	20	---	636.270	**779.689**	-0.102	19
Y	21	---	690.625	**741.347**	1.249	18
Q	22	---	733.311	686.992	---	17
R	23	---	785.345	644.306	---	16
P	24	---	817.696	592.272	---	15
H	25	---	863.382	559.921	---	14
S	26	---	892.393	514.235	---	13
A	27	---	916.072	485.224	---	12
Q	28	---	958.758	461.545	---	11
P	29	---	991.109	418.859	---	10
A	30	---	1014.788	386.508	---	9
D	31	---	1053.130	362.829	---	8
R	32	---	1105.164	324.487	---	7
G	33	---	1124.171	272.453	---	6
Y@	34	---	1205.181	253.446	---	5
D	35	---	1243.523	172.436	---	4
R	36	---	1295.557	134.094	---	3
P	37	---	1327.908	82.060	---	2
K	38	---	---	49.709	---	1

+4

Seq	#	b: Δ Error	b	y	y: Δ Error	+1
H	1	---	35.272	---	---	38
S	2	---	57.030	998.444	---	37
L	3	---	85.301	976.686	---	36
S	4	---	107.059	948.415	---	35
S	5	---	128.817	926.657	---	34
G	6	---	143.072	904.899	---	33
G	7	---	157.328	890.644	---	32
F	8	---	194.095	876.389	---	31
S@	9	---	235.844	839.621	---	30
P	10	---	260.108	**797.872**	0.599	29
V	11	---	284.875	773.609	---	28
A	12	---	302.634	**748.842**	-2.024	27
G	13	---	316.889	**731.082**	2.771	26
V	14	---	341.657	**716.827**	-2.205	25
G	15	---	355.912	**692.060**	-0.589	24
A	16	---	373.671	**677.804**	0.292	23
Q	17	---	405.686	**660.045**	3.076	22
D	18	---	434.443	628.031	---	21
G	19	---	448.698	599.274	---	20
D	20	---	477.455	585.018	---	19
Y	21	---	518.220	556.262	---	18
Q	22	---	550.235	515.496	---	17
R	23	---	589.260	483.481	---	16
P	24	---	613.524	444.456	---	15
H	25	---	647.788	420.193	---	14
S	26	---	669.546	385.928	---	13
A	27	---	687.306	364.170	---	12
Q	28	---	719.320	346.411	---	11
P	29	---	743.583	314.396	---	10
A	30	---	761.343	290.133	---	9
D	31	---	790.099	272.374	---	8
R	32	---	829.125	243.617	---	7
G	33	2.146	**843.380**	204.592	---	6
Y@	34	---	904.138	190.336	---	5
D	35	---	932.894	129.579	---	4
R	36	---	971.920	100.822	---	3
P	37	---	996.183	61.797	---	2
K	38	---	---	37.534	---	1

HSLSSGGFSPVAGVGAQDGDYQRPHS@AQPADRGYDRPK

z = 5+

DCBLD1 pS657

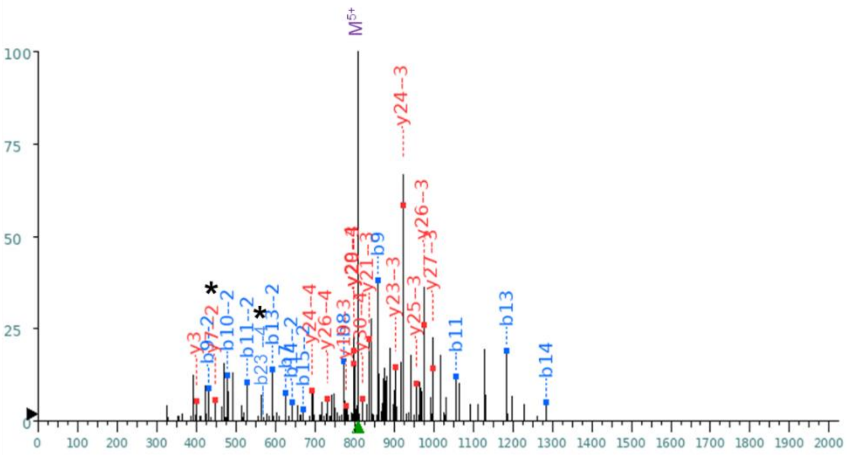

+1

Seq	#	b: Δ Error	b	y	y: Δ Error	+1
H	1	---	138.066	---	---	38
S	2	---	225.098	3910.789	---	37
L	3	---	338.182	3823.757	---	36
S	4	---	425.214	3710.673	---	35
S	5	---	512.246	3623.641	---	34
G	6	---	569.268	3536.609	---	33
G	7	-2.077	**626.289**	3479.588	---	32
F	8	-1.989	**773.358**	3422.566	---	31
S	9	-2.125	**860.390**	3275.498	---	30
P	10	---	957.442	3188.466	---	29
V	11	-1.752	**1056.511**	3091.413	---	28
A	12	---	1127.548	2992.345	---	27
G	13	-0.416	**1184.569**	2921.307	---	26
V	14	-0.141	**1283.638**	2864.286	---	25
G	15	---	1340.659	2765.218	---	24
A	16	---	1411.696	2708.196	---	23
Q	17	---	1539.755	2637.159	---	22
D	18	---	1654.782	2509.100	---	21
G	19	---	1711.803	2394.073	---	20
D	20	---	1826.830	2337.052	---	19
Y	21	---	1989.894	2222.025	---	18
Q	22	---	2117.952	2058.962	---	17
R	23	---	2274.053	1930.903	---	16
P	24	---	2371.106	1774.802	---	15
H	25	---	2508.165	1677.749	---	14
S®	26	---	2675.163	1540.690	---	13
A	27	---	2746.201	1373.692	---	12
Q	28	---	2874.259	1302.655	---	11
P	29	---	2971.312	1174.596	---	10
A	30	---	3042.349	1077.544	---	9
D	31	---	3157.376	1006.506	---	8
R	32	---	3313.477	891.480	---	7
G	33	---	3370.499	735.378	---	6
Y	34	---	3533.562	678.357	---	5
D	35	---	3648.589	515.294	---	4
R	36	---	3804.690	**400.267**	-2.558	3
P	37	---	3901.743	244.166	---	2
K	38	---	---	147.113	---	1

+2

Seq	#	b: Δ Error	b	y	y: Δ Error	+1
H	1	---	69.537	---	---	38
S	2	---	113.053	1955.898	---	37
L	3	---	169.595	1912.382	---	36
S	4	---	213.111	1855.840	---	35
S	5	---	256.627	1812.324	---	34
G	6	---	285.138	1768.808	---	33
G	7	---	313.648	1740.297	---	32
F	8	---	387.182	1711.787	---	31
S	9	-4.901	**430.698**	1638.253	---	30
P	10	0.341	**479.225**	1594.737	---	29
V	11	-3.667	**528.759**	1546.210	---	28
A	12	---	564.278	1496.676	---	27
G	13	-0.581	**592.788**	1461.157	---	26
V	14	-1.624	**642.323**	1432.647	---	25
G	15	-3.177	**670.833**	1383.112	---	24
A	16	---	706.352	1354.602	---	23
Q	17	---	770.381	1319.083	---	22
D	18	---	827.895	1255.054	---	21
G	19	---	856.405	1197.540	---	20
D	20	---	913.919	1169.030	---	19
Y	21	---	995.450	1111.516	---	18
Q	22	---	1059.480	1029.985	---	17
R	23	---	1137.530	965.955	---	16
P	24	---	1186.057	887.905	---	15
H	25	---	1254.586	839.378	---	14
S®	26	---	1338.085	770.849	---	13
A	27	---	1373.604	687.350	---	12
Q	28	---	1437.633	651.831	---	11
P	29	---	1486.160	587.802	---	10
A	30	---	1521.678	539.275	---	9
D	31	---	1579.192	503.757	---	8
R	32	---	1657.242	**446.243**	-2.377	7
G	33	---	1685.753	368.193	---	6
Y	34	---	1767.285	339.682	---	5
D	35	---	1824.798	258.150	---	4
R	36	---	1902.849	200.637	---	3
P	37	---	1951.375	122.586	---	2
K	38	---	---	74.060	---	1

+3

Seq	#	b: Δ Error	b	y	y: Δ Error	+1
H	1	---	46.694	---	---	38
S	2	---	75.704	1304.268	---	37
L	3	---	113.399	1275.257	---	36
S	4	---	142.410	1237.563	---	35
S	5	---	171.420	1208.552	---	34
G	6	---	190.427	1179.541	---	33
G	7	---	209.435	1160.534	---	32
F	8	---	258.457	1141.527	---	31
S	9	---	287.468	1092.504	---	30
P	10	---	319.819	1063.493	---	29
V	11	---	352.842	1031.143	---	28
A	12	---	376.521	**998.120**	2.118	27
G	13	---	395.528	**974.441**	-3.070	26
V	14	---	428.551	**955.434**	0.716	25
G	15	---	447.558	**922.411**	-1.268	24
A	16	---	471.237	**903.404**	-1.820	23
Q	17	---	513.923	879.725	---	22
D	18	---	552.266	**837.038**	-1.896	21
G	19	---	571.273	**798.696**	-1.721	20
D	20	---	609.615	**779.689**	-0.337	19
Y	21	---	663.969	741.347	---	18
Q	22	---	706.656	686.992	---	17
R	23	---	758.689	644.306	---	16
P	24	---	791.040	592.272	---	15
H	25	---	836.727	559.921	---	14
S®	26	---	892.393	514.235	---	13
A	27	---	916.072	458.569	---	12
Q	28	---	958.758	434.890	---	11
P	29	---	991.109	392.204	---	10
A	30	---	1014.788	359.853	---	9
D	31	---	1053.130	336.174	---	8
R	32	---	1105.164	297.831	---	7
G	33	---	1124.171	245.798	---	6
Y	34	---	1178.525	226.791	---	5
D	35	---	1216.868	172.436	---	4
R	36	---	1268.901	134.094	---	3
P	37	---	1301.252	82.060	---	2
K	38	---	---	49.709	---	1

+4

Seq	#	b: Δ Error	b	y	y: Δ Error	+1
H	1	---	35.272	---	---	38
S	2	---	57.030	978.453	---	37
L	3	---	85.301	956.695	---	36
S	4	---	107.059	928.424	---	35
S	5	---	128.817	906.666	---	34
G	6	---	143.072	884.908	---	33
G	7	---	157.328	870.652	---	32
F	8	---	194.095	856.397	---	31
S	9	---	215.853	**819.630**	-2.335	30
P	10	---	240.116	**797.872**	-1.620	29
V	11	---	264.883	773.609	---	28
A	12	---	282.642	748.842	---	27
G	13	---	296.898	**731.082**	-3.491	26
V	14	---	321.665	716.827	---	25
G	15	---	335.920	**692.060**	-0.236	24
A	16	---	353.680	677.804	---	23
Q	17	---	385.694	660.045	---	22
D	18	---	414.451	628.031	---	21
G	19	---	428.706	599.274	---	20
D	20	---	457.463	585.018	---	19
Y	21	---	498.229	556.262	---	18
Q	22	---	530.244	515.496	---	17
R	23	---	569.269	483.481	---	16
P	24	---	593.532	444.456	---	15
H	25	---	627.797	420.193	---	14
S®	26	---	669.546	385.928	---	13
A	27	---	687.306	344.178	---	12
Q	28	---	719.320	326.419	---	11
P	29	---	743.583	294.405	---	10
A	30	---	761.343	270.141	---	9
D	31	---	790.099	252.382	---	8
R	32	---	829.125	223.625	---	7
G	33	---	843.380	184.600	---	6
Y	34	---	884.146	170.345	---	5
D	35	---	912.903	129.579	---	4
R	36	---	951.928	100.822	---	3
P	37	---	976.191	61.797	---	2
K	38	---	---	37.534	---	1

HSLSSGGFSPVAGVGAQDGDYQRPHSAQPADRGY@DRPK
z = 5+

DCBLD1 pY665

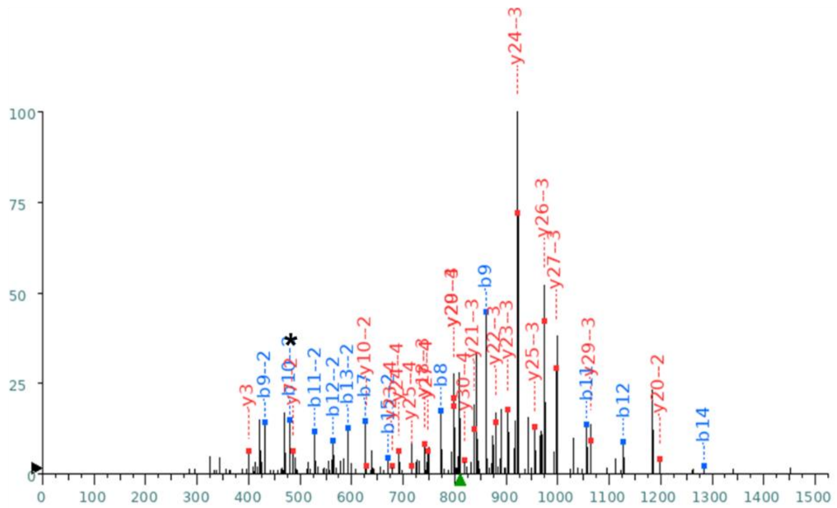

Seq	#	b: Δ Error	b	y	y: Δ Error	+1
			**+1**			
H	1	---	138.066	---	---	38
S	2	---	225.098	3910.789	---	37
L	3	---	338.182	3823.757	---	36
S	4	---	425.214	3710.673	---	35
S	5	---	512.246	3623.641	---	34
G	6	---	569.268	3536.609	---	33
G	7	-0.810	626.289	3479.588	---	32
F	8	0.221	773.358	3422.566	---	31
S	9	0.074	860.390	3275.498	---	30
P	10	---	957.442	3188.466	---	29
V	11	0.674	1056.511	3091.413	---	28
A	12	-2.512	1127.548	2992.345	---	27
G	13	---	1184.569	2921.307	---	26
V	14	-0.806	1283.638	2864.286	---	25
G	15	---	1340.659	2765.218	---	24
A	16	---	1411.696	2708.196	---	23
Q	17	---	1539.755	2637.159	---	22
D	18	---	1654.782	2509.100	---	21
G	19	---	1711.803	2394.073	---	20
D	20	---	1826.830	2337.052	---	19
Y	21	---	1989.894	2222.025	---	18
Q	22	---	2117.952	2058.962	---	17
R	23	---	2274.053	1930.903	---	16
P	24	---	2371.106	1774.802	---	15
H	25	---	2508.165	1677.749	---	14
S	26	---	2595.197	1540.690	---	13
A	27	---	2666.234	1453.658	---	12
Q	28	---	2794.293	1382.621	---	11
P	29	---	2891.346	1254.563	---	10
A	30	---	2962.383	1157.510	---	9
D	31	---	3077.410	1086.473	---	8
R	32	---	3233.511	971.446	---	7
G	33	---	3290.532	815.345	---	6
Y@	34	---	3533.562	758.323	---	5
D	35	---	3648.589	515.294	---	4
R	36	---	3804.690	400.267	2.245	3
P	37	---	3901.743	244.166	---	2
K	38	---	---	147.113	---	1

Seq	#	b: Δ Error	b	y	y: Δ Error	+1
			**+2**			
H	1	---	69.537	---	---	38
S	2	---	113.053	1955.898	---	37
L	3	---	169.595	1912.382	---	36
S	4	---	213.111	1855.840	---	35
S	5	---	256.627	1812.324	---	34
G	6	---	285.138	1768.808	---	33
G	7	---	313.648	1740.297	---	32
F	8	---	387.182	1711.787	---	31
S	9	-1.004	430.698	1638.253	---	30
P	10	0.213	479.225	1594.737	---	29
V	11	0.373	528.759	1546.210	---	28
A	12	-1.710	564.278	1496.676	---	27
G	13	0.140	592.788	1461.157	---	26
V	14	---	642.323	1432.647	---	25
G	15	1.190	670.833	1383.112	---	24
A	16	---	706.352	1354.602	---	23
Q	17	---	770.381	1319.083	---	22
D	18	---	827.895	1255.054	---	21
G	19	---	856.405	1197.540	0.220	20
D	20	---	913.919	1169.030	---	19
Y	21	---	995.450	1111.516	---	18
Q	22	---	1059.480	1029.985	---	17
R	23	---	1137.530	965.955	---	16
P	24	---	1186.057	887.905	---	15
H	25	---	1254.586	839.378	---	14
S	26	---	1298.102	770.849	---	13
A	27	---	1333.621	727.333	---	12
Q	28	---	1397.650	691.814	---	11
P	29	---	1446.176	627.785	0.773	10
A	30	---	1481.695	579.259	---	9
D	31	---	1539.208	543.740	---	8
R	32	---	1617.259	486.227	3.759	7
G	33	---	1645.770	408.176	---	6
Y@	34	---	1767.285	379.665	---	5
D	35	---	1824.798	258.150	---	4
R	36	---	1902.849	200.637	---	3
P	37	---	1951.375	122.586	---	2
K	38	---	---	74.060	---	1

Seq	#	b: Δ Error	b	y	y: Δ Error	+1
			**+3**			
H	1	---	46.694	---	---	38
S	2	---	75.704	1304.268	---	37
L	3	---	113.399	1275.257	---	36
S	4	---	142.410	1237.563	---	35
S	5	---	171.420	1208.552	---	34
G	6	---	190.427	1179.541	---	33
G	7	---	209.435	1160.534	---	32
F	8	---	258.457	1141.527	---	31
S	9	---	287.468	1092.504	---	30
P	10	---	319.819	1063.493	0.890	29
V	11	---	352.842	1031.143	---	28
A	12	---	376.521	998.120	-0.634	27
G	13	---	395.528	974.441	-0.063	26
V	14	---	428.551	955.434	0.013	25
G	15	---	447.558	922.411	0.254	24
A	16	---	471.237	903.404	-0.672	23
Q	17	---	513.923	879.725	-2.746	22
D	18	---	552.266	837.038	-0.073	21
G	19	---	571.273	798.696	2.329	20
D	20	---	609.615	779.689	---	19
Y	21	---	663.969	741.347	1.990	18
Q	22	---	706.656	686.992	---	17
R	23	---	758.689	644.306	---	16
P	24	---	791.040	592.272	---	15
H	25	---	836.727	559.921	---	14
S	26	---	865.737	514.235	---	13
A	27	---	889.416	485.224	---	12
Q	28	---	932.102	461.545	---	11
P	29	---	964.453	418.859	---	10
A	30	---	988.132	386.508	---	9
D	31	---	1026.475	362.829	---	8
R	32	---	1078.508	324.487	---	7
G	33	---	1097.516	272.453	---	6
Y@	34	---	1178.525	253.446	---	5
D	35	---	1216.868	172.436	---	4
R	36	---	1268.901	134.094	---	3
P	37	---	1301.252	82.060	---	2
K	38	---	---	49.709	---	1

Seq	#	b: Δ Error	b	y	y: Δ Error	+1
			**+4**			
H	1	---	35.272	---	---	38
S	2	---	57.030	978.453	---	37
L	3	---	85.301	956.695	---	36
S	4	---	107.059	928.424	---	35
S	5	---	128.817	906.666	---	34
G	6	---	143.072	884.908	---	33
G	7	---	157.328	870.652	---	32
F	8	---	194.095	856.397	---	31
S	9	---	215.853	819.630	2.952	30
P	10	---	240.116	797.872	1.364	29
V	11	---	264.883	773.609	---	28
A	12	---	282.642	748.842	2.948	27
G	13	---	296.898	731.082	---	26
V	14	---	321.665	716.827	1.371	25
G	15	---	335.920	692.060	1.352	24
A	16	---	353.680	677.804	1.553	23
Q	17	---	385.694	660.045	---	22
D	18	---	414.451	628.031	---	21
G	19	---	428.706	599.274	---	20
D	20	---	457.463	585.018	---	19
Y	21	---	498.229	556.262	---	18
Q	22	---	530.244	515.496	---	17
R	23	---	569.269	483.481	---	16
P	24	---	593.532	444.456	---	15
H	25	---	627.797	420.193	---	14
S	26	---	649.555	385.928	---	13
A	27	---	667.314	364.170	---	12
Q	28	---	699.329	346.411	---	11
P	29	---	723.592	314.396	---	10
A	30	---	741.351	290.133	---	9
D	31	---	770.108	272.374	---	8
R	32	---	809.133	243.617	---	7
G	33	---	823.389	204.592	---	6
Y@	34	---	884.146	190.336	---	5
D	35	---	912.903	129.579	---	4
R	36	---	951.928	100.822	---	3
P	37	---	976.191	61.797	---	2
K	38	---	---	37.534	---	1

HSL[SS]@GGFSPVAGVGAQDGDY@QRPHSAQPADRGY@DRPK
z = 5+

DCBLD1 pS[635 or 636], pY652, pY665

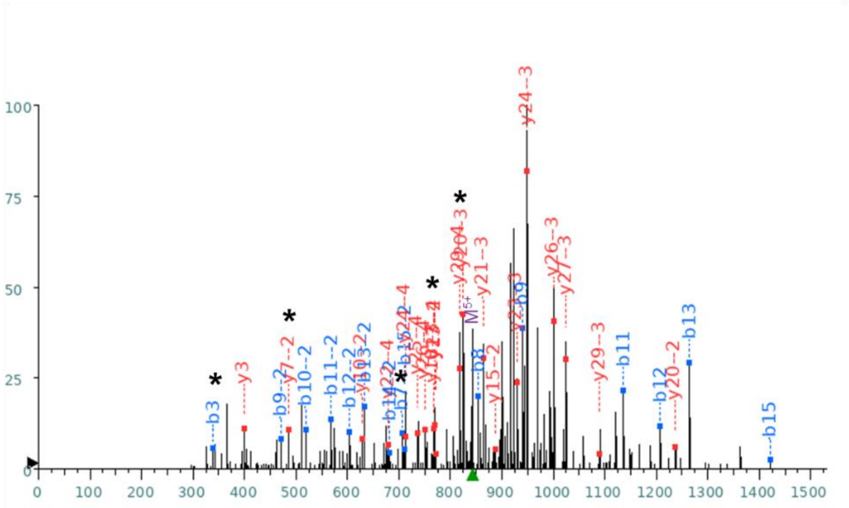

+1

Seq	#	b: Δ Error	b	y	y: Δ Error	+1
H	1	---	138.066	---	---	38
S	2	---	225.098	4070.722	---	37
L	3	0.455	**338.182**	3983.690	---	36
S	4	---	425.214	3870.606	---	35
S@	5	---	592.213	3783.574	---	34
G	6	---	649.234	3616.575	---	33
G	7	-0.231	**706.256**	3559.554	---	32
F	8	-1.828	**853.324**	3502.533	---	31
S	9	-0.929	**940.356**	3355.464	---	30
P	10	---	1037.409	3268.432	---	29
V	11	-0.359	**1136.477**	3171.379	---	28
A	12	-0.847	**1207.514**	3072.311	---	27
G	13	-0.407	**1264.536**	3001.274	---	26
V	14	---	1363.604	2944.252	---	25
G	15	1.591	**1420.626**	2845.184	---	24
A	16	---	1491.663	2788.162	---	23
Q	17	---	1619.721	2717.125	---	22
D	18	---	1734.748	2589.067	---	21
G	19	---	1791.770	2474.040	---	20
D	20	---	1906.797	2417.018	---	19
Y@	21	---	2149.826	2301.991	---	18
Q	22	---	2277.885	2058.962	---	17
R	23	---	2433.986	1930.903	---	16
P	24	---	2531.039	1774.802	---	15
H	25	---	2668.098	1677.749	---	14
S	26	---	2755.130	1540.690	---	13
A	27	---	2826.167	1453.658	---	12
Q	28	---	2954.225	1382.621	---	11
P	29	---	3051.278	1254.563	---	10
A	30	---	3122.315	1157.510	---	9
D	31	---	3237.342	1086.473	---	8
R	32	---	3393.443	971.446	---	7
G	33	---	3450.465	815.345	---	6
Y@	34	---	3693.495	758.323	---	5
D	35	---	3808.521	515.294	---	4
R	36	---	3964.623	**400.267**	-1.338	3
P	37	---	4061.675	244.166	---	2
K	38	---	---	147.113	---	1

+2

Seq	#	b: Δ Error	b	y	y: Δ Error	+1
H	1	---	69.537	---	---	38
S	2	---	113.053	2035.865	---	37
L	3	---	169.595	1992.349	---	36
S	4	---	213.111	1935.807	---	35
S@	5	---	296.610	1892.291	---	34
G	6	---	325.121	1808.791	---	33
G	7	---	353.631	1780.281	---	32
F	8	---	427.166	1751.770	---	31
S	9	0.614	**470.682**	1678.236	---	30
P	10	0.764	**519.208**	1634.720	---	29
V	11	-1.282	**568.742**	1586.193	---	28
A	12	-3.635	**604.261**	1536.659	---	27
G	13	-2.008	**632.772**	1501.141	---	26
V	14	-2.081	**682.306**	1472.630	---	25
G	15	0.679	**710.816**	1423.096	---	24
A	16	---	746.335	1394.585	---	23
Q	17	---	810.364	1359.066	---	22
D	18	---	867.878	1295.037	---	21
G	19	---	896.389	**1237.524**	-0.486	20
D	20	---	953.902	1209.013	---	19
Y@	21	---	1075.417	1151.499	---	18
Q	22	---	1139.446	1029.985	---	17
R	23	---	1217.497	965.955	---	16
P	24	---	1266.023	**887.905**	-0.012	15
H	25	---	1334.553	839.378	---	14
S	26	---	1378.069	**770.849**	2.587	13
A	27	---	1413.587	727.333	---	12
Q	28	---	1477.616	691.814	---	11
P	29	---	1526.143	**627.785**	-2.241	10
A	30	---	1561.661	579.259	---	9
D	31	---	1619.175	543.740	---	8
R	32	---	1697.225	**486.227**	-1.451	7
G	33	---	1725.736	408.176	---	6
Y@	34	---	1847.251	379.665	---	5
D	35	---	1904.764	258.150	---	4
R	36	---	1982.815	200.637	---	3
P	37	---	2031.341	122.586	---	2
K	38	---	---	74.060	---	1

+3

Seq	#	b: Δ Error	b	y	y: Δ Error	+1
H	1	---	46.694	---	---	38
S	2	---	75.704	1357.579	---	37
L	3	---	113.399	1328.568	---	36
S	4	---	142.410	1290.873	---	35
S@	5	---	198.076	1261.863	---	34
G	6	---	217.083	1206.197	---	33
G	7	---	236.090	1187.190	---	32
F	8	---	285.113	1168.182	---	31
S	9	---	314.124	1119.160	---	30
P	10	---	346.474	**1090.149**	0.600	29
V	11	---	379.497	1057.798	---	28
A	12	---	403.176	**1024.775**	-2.749	27
G	13	---	422.183	**1001.096**	-1.085	26
V	14	---	455.206	982.089	---	25
G	15	---	474.213	**949.066**	-0.640	24
A	16	---	497.892	**930.059**	-2.738	23
Q	17	---	540.579	906.380	---	22
D	18	---	578.921	**863.694**	-1.257	21
G	19	---	597.928	**825.351**	1.086	20
D	20	---	636.270	806.344	---	19
Y@	21	---	717.280	**768.002**	0.031	18
Q	22	---	759.966	686.992	---	17
R	23	---	812.000	644.306	---	16
P	24	---	844.351	592.272	---	15
H	25	---	890.037	559.921	---	14
S	26	---	919.048	514.235	---	13
A	27	---	942.727	485.224	---	12
Q	28	---	985.413	461.545	---	11
P	29	---	1017.764	418.859	---	10
A	30	---	1041.443	386.508	---	9
D	31	---	1079.786	362.829	---	8
R	32	---	1131.819	324.487	---	7
G	33	---	1150.826	272.453	---	6
Y@	34	---	1231.836	253.446	---	5
D	35	---	1270.179	172.436	---	4
R	36	---	1322.212	134.094	---	3
P	37	---	1354.563	82.060	---	2
K	38	---	---	49.709	---	1

+4

Seq	#	b: Δ Error	b	y	y: Δ Error	+1
H	1	---	35.272	---	---	38
S	2	---	57.030	1018.436	---	37
L	3	---	85.301	996.678	---	36
S	4	---	107.059	968.407	---	35
S@	5	---	148.809	946.649	---	34
G	6	---	163.064	904.899	---	33
G	7	---	177.319	890.644	---	32
F	8	---	214.086	876.389	---	31
S	9	---	235.844	839.621	---	30
P	10	---	260.108	**817.863**	-2.408	29
V	11	---	284.875	793.600	---	28
A	12	---	302.634	**768.833**	-2.137	27
G	13	---	316.889	**751.074**	-1.130	26
V	14	---	341.657	**736.819**	1.409	25
G	15	---	355.912	**712.051**	-1.865	24
A	16	---	373.671	697.796	---	23
Q	17	---	405.686	**680.037**	-0.972	22
D	18	---	434.443	648.022	---	21
G	19	---	448.698	619.265	---	20
D	20	---	477.455	605.010	---	19
Y@	21	---	538.212	576.253	---	18
Q	22	---	570.227	515.496	---	17
R	23	---	609.252	483.481	---	16
P	24	---	633.515	444.456	---	15
H	25	---	667.780	420.193	---	14
S	26	---	689.538	385.928	---	13
A	27	---	707.297	364.170	---	12
Q	28	---	739.312	346.411	---	11
P	29	---	763.575	314.396	---	10
A	30	---	781.334	290.133	---	9
D	31	---	810.091	272.374	---	8
R	32	---	849.116	243.617	---	7
G	33	---	863.372	204.592	---	6
Y@	34	---	924.129	190.336	---	5
D	35	---	952.886	129.579	---	4
R	36	---	991.911	100.822	---	3
P	37	---	1016.174	61.797	---	2
K	38	---	---	37.534	---	1

HSLSSGGFSPVAGVGAQDGDYQRPHSAQPADR
z = 4+

DCBLD1 S636, S640, Y652, S657

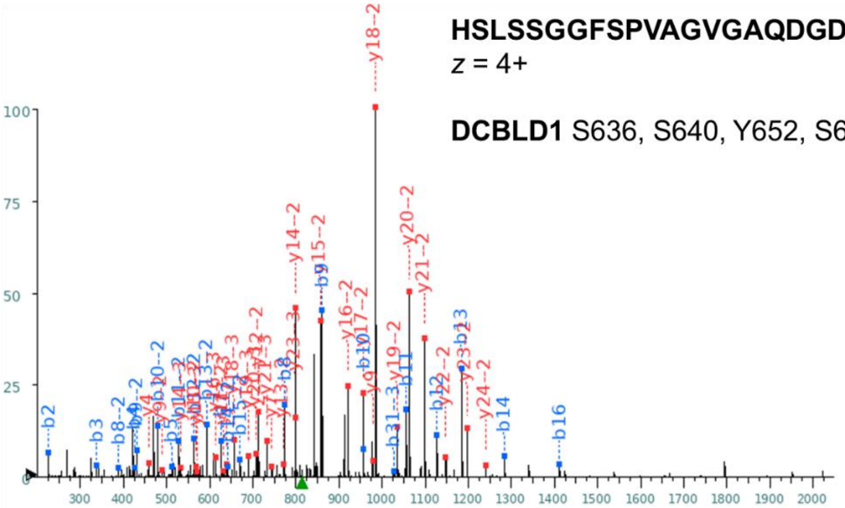

			+1			
Seq	#	b: Δ Error	b	y	y: Δ Error	+1
H	1	---	138.066	---	---	32
S	2	-0.793	225.098	3114.462	---	31
L	3	-2.704	338.182	3027.430	---	30
S	4	-2.043	425.214	2914.346	---	29
S	5	-0.356	512.246	2827.314	---	28
G	6	---	569.268	2740.282	---	27
G	7	-2.369	626.289	2683.261	---	26
F	8	-1.042	773.358	2626.239	---	25
S	9	-0.919	860.390	2479.171	---	24
P	10	-2.004	957.442	2392.139	---	23
V	11	-0.481	1056.511	2295.086	---	22
A	12	-1.321	1127.548	2196.018	---	21
G	13	-1.137	1184.569	2124.981	---	20
V	14	0.240	1283.638	2067.959	---	19
G	15	---	1340.659	1968.891	---	18
A	16	1.353	1411.696	1911.869	---	17
Q	17	---	1539.755	1840.832	---	16
D	18	---	1654.782	1712.774	---	15
G	19	---	1711.803	1597.747	---	14
D	20	---	1826.830	1540.725	---	13
Y	21	---	1989.894	1425.698	---	12
Q	22	---	2117.952	1262.635	---	11
R	23	---	2274.053	1134.576	---	10
P	24	---	2371.106	978.475	-0.384	9
H	25	---	2508.165	881.422	---	8
S	26	---	2595.197	744.363	-0.120	7
A	27	---	2666.234	657.331	---	6
Q	28	---	2794.293	586.294	---	5
P	29	---	2891.346	458.236	-0.920	4
A	30	---	2962.383	361.183	---	3
D	31	---	3077.410	290.146	---	2
R	32	---	---	175.119	---	1

			+2			
Seq	#	b: Δ Error	b	y	y: Δ Error	+1
H	1	---	69.537	---	---	32
S	2	---	113.053	1557.735	---	31
L	3	---	169.595	1514.219	---	30
S	4	---	213.111	1457.677	---	29
S	5	---	256.627	1414.161	---	28
G	6	---	285.138	1370.645	---	27
G	7	---	313.648	1342.134	---	26
F	8	-1.136	387.182	1313.623	---	25
S	9	-1.004	430.698	1240.089	0.910	24
P	10	-0.742	479.225	1196.573	-0.976	23
V	11	-0.320	528.759	1148.047	-1.643	22
A	12	-0.736	564.278	1098.512	-1.025	21
G	13	-1.301	592.788	1062.994	-0.828	20
V	14	-2.479	642.323	1034.483	-0.861	19
G	15	-0.629	670.833	984.949	-0.752	18
A	16	---	706.352	956.438	-0.786	17
Q	17	---	770.381	920.920	-0.880	16
D	18	---	827.895	856.890	-0.741	15
G	19	---	856.405	799.377	-0.664	14
D	20	---	913.919	770.866	-2.681	13
Y	21	---	995.450	713.353	-0.611	12
Q	22	---	1059.480	631.821	0.352	11
R	23	---	1137.530	567.792	-1.127	10
P	24	---	1186.057	489.741	-1.083	9
H	25	---	1254.586	441.215	---	8
S	26	---	1298.102	372.685	---	7
A	27	---	1333.621	329.169	---	6
Q	28	---	1397.650	293.651	---	5
P	29	---	1446.176	229.622	---	4
A	30	---	1481.695	181.095	---	3
D	31	---	1539.208	145.577	---	2
R	32	---	---	88.063	---	1

			+3			
Seq	#	b: Δ Error	b	y	y: Δ Error	+1
H	1	---	46.694	---	---	32
S	2	---	75.704	1038.826	---	31
L	3	---	113.399	1009.815	---	30
S	4	---	142.410	972.120	---	29
S	5	---	171.420	943.110	---	28
G	6	---	190.427	914.099	---	27
G	7	---	209.435	895.092	---	26
F	8	---	258.457	876.085	---	25
S	9	---	287.468	827.062	---	24
P	10	---	319.819	798.051	-0.217	23
V	11	---	352.842	765.700	---	22
A	12	---	376.521	732.677	-0.142	21
G	13	---	395.528	708.998	-0.633	20
V	14	---	428.551	689.991	-0.542	19
G	15	---	447.558	656.968	-1.719	18
A	16	---	471.237	637.961	-0.983	17
Q	17	---	513.923	614.282	-0.290	16
D	18	---	552.266	571.596	-2.706	15
G	19	---	571.273	533.254	-2.617	14
D	20	---	609.615	514.247	---	13
Y	21	---	663.969	475.904	---	12
Q	22	---	706.656	421.550	---	11
R	23	---	758.689	378.864	---	10
P	24	---	791.040	326.830	---	9
H	25	---	836.727	294.479	---	8
S	26	---	865.737	248.793	---	7
A	27	---	889.416	219.782	---	6
Q	28	---	932.102	196.103	---	5
P	29	---	964.453	153.417	---	4
A	30	---	988.132	121.066	---	3
D	31	0.366	1026.475	97.387	---	2
R	32	---	---	59.045	---	1

HSLSS@GGFSPVAGVGAQDGDY@QRPHSAQPADR

z = 4+

DCBLD1 pS636, pY652

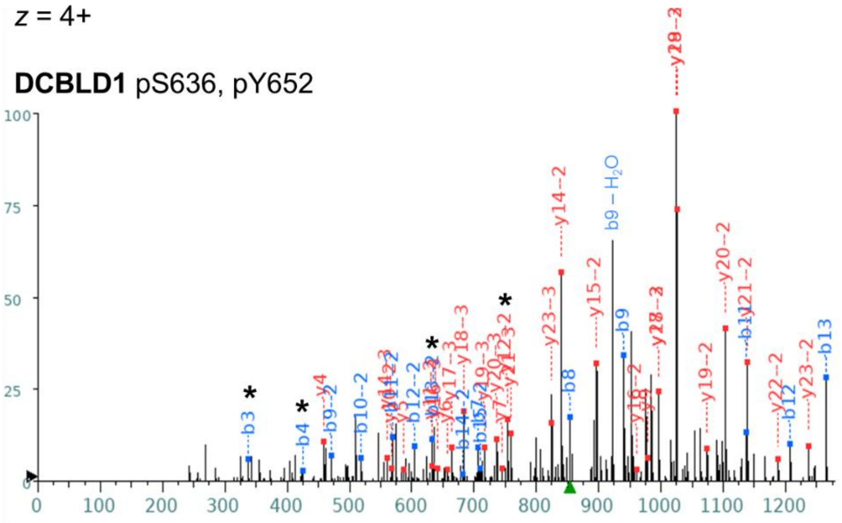

Seq	#	b: Δ Error	b	y	y: Δ Error	+1
**+1**						
H	1	---	138.066	---	---	32
S	2	---	225.098	3274.395	---	31
L	3	-0.808	338.182	3187.363	---	30
S	4	-1.182	425.214	3074.279	---	29
S@	5	---	592.213	2987.247	---	28
G	6	---	649.234	2820.249	---	27
G	7	0.807	706.256	2763.227	---	26
F	8	-2.615	853.324	2706.206	---	25
S	9	0.304	940.356	2559.137	---	24
P	10	---	1037.409	2472.105	---	23
V	11	-1.004	1136.477	2375.052	---	22
A	12	-0.039	1207.514	2275.984	---	21
G	13	-0.117	1264.536	2204.947	---	20
V	14	0.389	1363.604	2147.925	---	19
G	15	---	1420.626	2048.857	---	18
A	16	---	1491.663	1991.836	---	17
Q	17	---	1619.721	1920.798	---	16
D	18	---	1734.748	1792.740	---	15
G	19	---	1791.770	1677.713	---	14
D	20	---	1906.797	1620.691	---	13
Y@	21	---	2149.826	1505.665	---	12
Q	22	---	2277.885	1262.635	---	11
R	23	---	2433.986	1134.576	---	10
P	24	---	2531.039	978.475	0.052	9
H	25	---	2668.098	881.422	---	8
S	26	---	2755.130	744.363	3.405	7
A	27	---	2826.167	657.331	2.905	6
Q	28	---	2954.225	586.294	1.078	5
P	29	---	3051.278	458.236	-0.521	4
A	30	---	3122.315	361.183	---	3
D	31	---	3237.342	290.146	---	2
R	32	---	---	175.119	---	1

Seq	#	b: Δ Error	b	y	y: Δ Error	+1
**+2**						
H	1	---	69.537	---	---	32
S	2	---	113.053	1637.701	---	31
L	3	---	169.595	1594.185	---	30
S	4	---	213.111	1537.643	---	29
S@	5	---	296.610	1494.127	---	28
G	6	---	325.121	1410.628	---	27
G	7	---	353.631	1382.117	---	26
F	8	---	427.166	1353.606	---	25
S	9	3.727	470.682	1280.072	---	24
P	10	1.704	519.208	1236.556	-1.151	23
V	11	0.864	568.742	1188.030	2.205	22
A	12	0.809	604.261	1138.496	-1.106	21
G	13	-0.465	632.772	1102.977	-0.033	20
V	14	-0.471	682.306	1074.466	0.411	19
G	15	-1.124	710.816	1024.932	0.457	18
A	16	---	746.335	996.421	0.889	17
Q	17	---	810.364	960.903	1.877	16
D	18	---	867.878	896.874	-0.040	15
G	19	---	896.389	839.360	0.519	14
D	20	---	953.902	810.849	---	13
Y@	21	---	1075.417	753.336	0.136	12
Q	22	---	1139.446	631.821	1.028	11
R	23	---	1217.497	567.792	3.065	10
P	24	---	1266.023	489.741	---	9
H	25	---	1334.553	441.215	---	8
S	26	---	1378.069	372.685	---	7
A	27	---	1413.587	329.169	---	6
Q	28	---	1477.616	293.651	---	5
P	29	---	1526.143	229.622	---	4
A	30	---	1561.661	181.095	---	3
D	31	---	1619.175	145.577	---	2
R	32	---	---	88.063	---	1

Seq	#	b: Δ Error	b	y	y: Δ Error	+1
**+3**						
H	1	---	46.694	---	---	32
S	2	---	75.704	1092.137	---	31
L	3	---	113.399	1063.126	---	30
S	4	---	142.410	1025.431	1.415	29
S@	5	---	198.076	996.420	1.819	28
G	6	---	217.083	940.754	---	27
G	7	---	236.090	921.747	---	26
F	8	---	285.113	902.740	---	25
S	9	---	314.124	853.717	---	24
P	10	---	346.474	824.707	-0.934	23
V	11	---	379.497	792.356	---	22
A	12	---	403.176	759.333	0.764	21
G	13	---	422.183	735.654	1.980	20
V	14	---	455.206	716.647	-0.419	19
G	15	---	474.213	683.624	-0.025	18
A	16	---	497.892	664.617	1.004	17
Q	17	---	540.579	640.938	3.075	16
D	18	---	578.921	598.251	---	15
G	19	---	597.928	559.909	0.365	14
D	20	---	636.270	540.902	---	13
Y@	21	---	717.280	502.560	---	12
Q	22	---	759.966	421.550	---	11
R	23	---	812.000	378.864	---	10
P	24	---	844.351	326.830	---	9
H	25	---	890.037	294.479	---	8
S	26	---	919.048	248.793	---	7
A	27	---	942.727	219.782	---	6
Q	28	---	985.413	196.103	---	5
P	29	---	1017.764	153.417	---	4
A	30	---	1041.443	121.066	---	3
D	31	---	1079.786	97.387	---	2
R	32	---	---	59.045	---	1

HSLSSGGFS@PVAGVGAQDGDYQRPHSAQPADR

z = 4+

DCBLD1 pS640

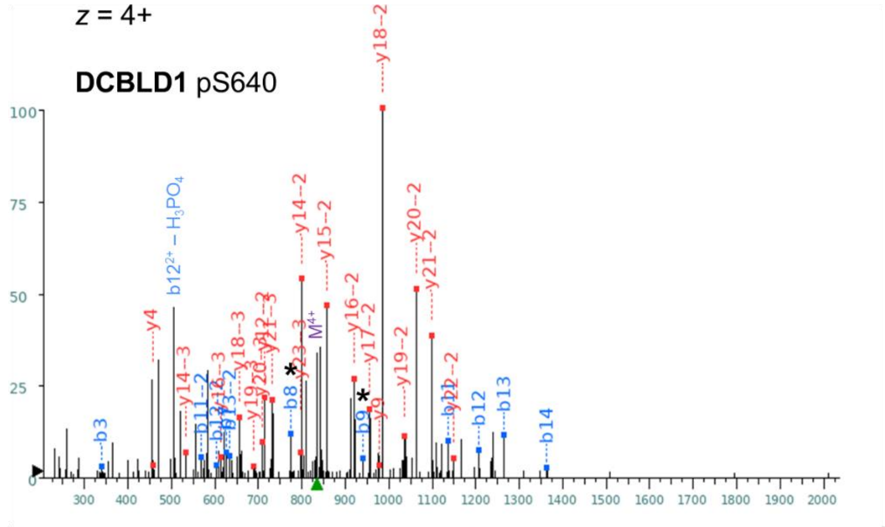

+1

Seq	#	b: Δ Error	b	y	y: Δ Error	+1
H	1	---	138.066	---	---	32
S	2	---	225.098	3194.429	---	31
L	3	-0.538	338.182	3107.397	---	30
S	4	---	425.214	2994.313	---	29
S	5	---	512.246	2907.281	---	28
G	6	---	569.268	2820.249	---	27
G	7	2.406	626.289	2763.227	---	26
F	8	-1.910	773.358	2706.206	---	25
S@	9	0.888	940.356	2559.137	---	24
P	10	---	1037.409	2392.139	---	23
V	11	2.433	1136.477	2295.086	---	22
A	12	-0.140	1207.514	2196.018	---	21
G	13	1.910	1264.536	2124.981	---	20
V	14	-0.775	1363.604	2067.959	---	19
G	15	---	1420.626	1968.891	---	18
A	16	---	1491.663	1911.869	---	17
Q	17	---	1619.721	1840.832	---	16
D	18	---	1734.748	1712.774	---	15
G	19	---	1791.770	1597.747	---	14
D	20	---	1906.797	1540.725	---	13
Y	21	---	2069.860	1425.698	---	12
Q	22	---	2197.919	1262.635	---	11
R	23	---	2354.020	1134.576	---	10
P	24	---	2451.072	978.475	0.863	9
H	25	---	2588.131	881.422	---	8
S	26	---	2675.163	744.363	---	7
A	27	---	2746.201	657.331	---	6
Q	28	---	2874.259	586.294	---	5
P	29	---	2971.312	458.236	1.011	4
A	30	---	3042.349	361.183	---	3
D	31	---	3157.376	290.146	---	2
R	32	---	---	175.119	---	1

+2

Seq	#	b: Δ Error	b	y	y: Δ Error	+1
H	1	---	69.537	---	---	32
S	2	---	113.053	1597.718	---	31
L	3	---	169.595	1554.202	---	30
S	4	---	213.111	1497.660	---	29
S	5	---	256.627	1454.144	---	28
G	6	---	285.138	1410.628	---	27
G	7	---	313.648	1382.117	---	26
F	8	---	387.182	1353.606	---	25
S@	9	---	470.682	1280.072	---	24
P	10	---	519.208	1196.573	---	23
V	11	2.903	568.742	1148.047	-0.261	22
A	12	0.708	604.261	1098.512	-0.025	21
G	13	-2.297	632.772	1062.994	2.158	20
V	14	---	682.306	1034.483	1.971	19
G	15	---	710.816	984.949	-1.000	18
A	16	---	746.335	956.438	0.554	17
Q	17	---	810.364	920.920	0.843	16
D	18	---	867.878	856.890	-0.456	15
G	19	---	896.389	799.377	-0.358	14
D	20	---	953.902	770.866	---	13
Y	21	---	1035.434	713.353	-0.868	12
Q	22	---	1099.463	631.821	---	11
R	23	---	1177.514	567.792	---	10
P	24	---	1226.040	489.741	---	9
H	25	---	1294.569	441.215	---	8
S	26	---	1338.085	372.685	---	7
A	27	---	1373.604	329.169	---	6
Q	28	---	1437.633	293.651	---	5
P	29	---	1486.160	229.622	---	4
A	30	---	1521.678	181.095	---	3
D	31	---	1579.192	145.577	---	2
R	32	---	---	88.063	---	1

+3

Seq	#	b: Δ Error	b	y	y: Δ Error	+1
H	1	---	46.694	---	---	32
S	2	---	75.704	1065.481	---	31
L	3	---	113.399	1036.470	---	30
S	4	---	142.410	998.776	---	29
S	5	---	171.420	969.765	---	28
G	6	---	190.427	940.754	---	27
G	7	---	209.435	921.747	---	26
F	8	---	258.457	902.740	---	25
S@	9	---	314.124	853.717	---	24
P	10	---	346.474	798.051	0.089	23
V	11	---	379.497	765.700	---	22
A	12	---	403.176	732.677	-1.475	21
G	13	---	422.183	708.998	0.142	20
V	14	---	455.206	689.991	-2.046	19
G	15	---	474.213	656.968	-1.904	18
A	16	---	497.892	637.961	---	17
Q	17	---	540.579	614.282	2.194	16
D	18	---	578.921	571.596	---	15
G	19	---	597.928	533.254	1.275	14
D	20	---	636.270	514.247	---	13
Y	21	---	690.625	475.904	---	12
Q	22	---	733.311	421.550	---	11
R	23	---	785.345	378.864	---	10
P	24	---	817.696	326.830	---	9
H	25	---	863.382	294.479	---	8
S	26	---	892.393	248.793	---	7
A	27	---	916.072	219.782	---	6
Q	28	---	958.758	196.103	---	5
P	29	---	991.109	153.417	---	4
A	30	---	1014.788	121.066	---	3
D	31	---	1053.130	97.387	---	2
R	32	---	---	59.045	---	1

HSLSSGGFS@PVAGVGAQDGDY@QRPHSAQPADR

z = 4+

DCBLD1 pS640, pY652

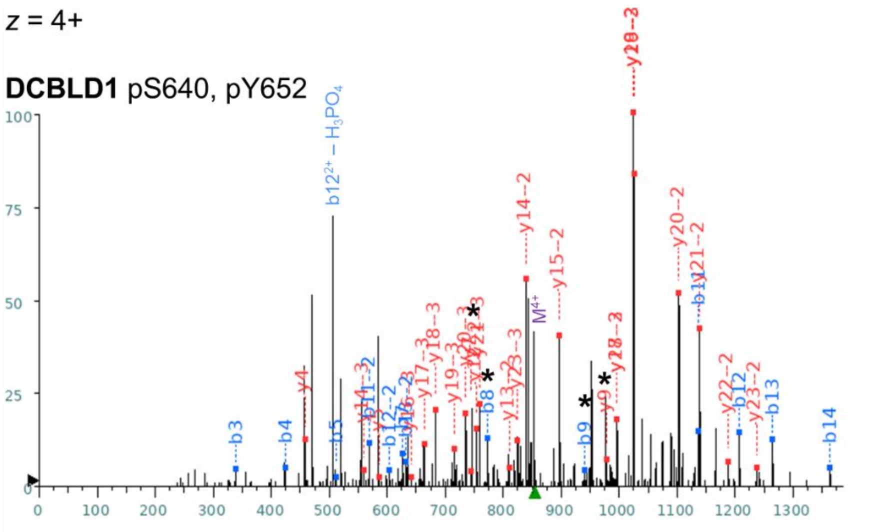

+1

Seq	#	b: Δ Error	b	y	y: Δ Error	+1
H	1	---	138.066	---	---	32
S	2	---	225.098	3274.395	---	31
L	3	-0.538	**338.182**	3187.363	---	30
S	4	1.545	**425.214**	3074.279	---	29
S	5	0.597	**512.246**	2987.247	---	28
G	6	---	569.268	2900.215	---	27
G	7	1.627	**626.289**	2843.193	---	26
F	8	0.300	**773.358**	2786.172	---	25
S@	9	2.316	**940.356**	2639.104	---	24
P	10	---	1037.409	2472.105	---	23
V	11	1.467	**1136.477**	2375.052	---	22
A	12	-0.746	**1207.514**	2275.984	---	21
G	13	-1.469	**1264.536**	2204.947	---	20
V	14	1.015	**1363.604**	2147.925	---	19
G	15	---	1420.626	2048.857	---	18
A	16	---	1491.663	1991.836	---	17
Q	17	---	1619.721	1920.798	---	16
D	18	---	1734.748	1792.740	---	15
G	19	---	1791.770	1677.713	---	14
D	20	---	1906.797	1620.691	---	13
Y@	21	---	2149.826	1505.665	---	12
Q	22	---	2277.885	1262.635	---	11
R	23	---	2433.986	1134.576	---	10
P	24	---	2531.039	**978.475**	0.988	9
H	25	---	2668.098	881.422	---	8
S	26	---	2755.130	**744.363**	2.094	7
A	27	---	2826.167	657.331	---	6
Q	28	---	2954.225	**586.294**	2.328	5
P	29	---	3051.278	**458.236**	0.079	4
A	30	---	3122.315	361.183	---	3
D	31	---	3237.342	290.146	---	2
R	32	---	---	175.119	---	1

+2

Seq	#	b: Δ Error	b	y	y: Δ Error	+1
H	1	---	69.537	---	---	32
S	2	---	113.053	1637.701	---	31
L	3	---	169.595	1594.185	---	30
S	4	---	213.111	1537.643	---	29
S	5	---	256.627	1494.127	---	28
G	6	---	285.138	1450.611	---	27
G	7	---	313.648	1422.100	---	26
F	8	---	387.182	1393.590	---	25
S@	9	---	470.682	1320.055	---	24
P	10	---	519.208	**1236.556**	0.724	23
V	11	0.005	**568.742**	**1188.030**	1.897	22
A	12	1.920	**604.261**	**1138.496**	-1.428	21
G	13	-1.622	**632.772**	**1102.977**	-0.586	20
V	14	---	682.306	1074.466	---	19
G	15	---	710.816	**1024.932**	0.100	18
A	16	---	746.335	**996.421**	-0.643	17
Q	17	---	810.364	960.903	---	16
D	18	---	867.878	**896.874**	-0.993	15
G	19	---	896.389	**839.360**	0.010	14
D	20	---	953.902	**810.849**	2.556	13
Y@	21	---	1075.417	**753.336**	1.108	12
Q	22	---	1139.446	631.821	---	11
R	23	---	1217.497	567.792	---	10
P	24	---	1266.023	489.741	---	9
H	25	---	1334.553	441.215	---	8
S	26	---	1378.069	372.685	---	7
A	27	---	1413.587	329.169	---	6
Q	28	---	1477.616	293.651	---	5
P	29	---	1526.143	229.622	---	4
A	30	---	1561.661	181.095	---	3
D	31	---	1619.175	145.577	---	2
R	32	---	---	88.063	---	1

+3

Seq	#	b: Δ Error	b	y	y: Δ Error	+1
H	1	---	46.694	---	---	32
S	2	---	75.704	1092.137	---	31
L	3	---	113.399	1063.126	---	30
S	4	---	142.410	**1025.431**	1.415	29
S	5	---	171.420	**996.420**	0.287	28
G	6	---	190.427	967.410	---	27
G	7	---	209.435	948.403	---	26
F	8	---	258.457	929.396	---	25
S@	9	---	314.124	880.373	---	24
P	10	---	346.474	**824.707**	2.026	23
V	11	---	379.497	792.356	---	22
A	12	---	403.176	**759.333**	-0.040	21
G	13	---	422.183	**735.654**	-0.758	20
V	14	---	455.206	**716.647**	0.603	19
G	15	---	474.213	**683.624**	0.778	18
A	16	---	497.892	**664.617**	-2.026	17
Q	17	---	540.579	**640.938**	-0.734	16
D	18	---	578.921	598.251	---	15
G	19	---	597.928	**559.909**	2.654	14
D	20	---	636.270	540.902	---	13
Y@	21	---	717.280	502.560	---	12
Q	22	---	759.966	421.550	---	11
R	23	---	812.000	378.864	---	10
P	24	---	844.351	326.830	---	9
H	25	---	890.037	294.479	---	8
S	26	---	919.048	248.793	---	7
A	27	---	942.727	219.782	---	6
Q	28	---	985.413	196.103	---	5
P	29	---	1017.764	153.417	---	4
A	30	---	1041.443	121.066	---	3
D	31	---	1079.786	97.387	---	2
R	32	---	---	59.045	---	1

HSLSSGGFSPVAGVGAQDGDY@QRPHSAQPADR

z = 4+

DCBLD1 pY652

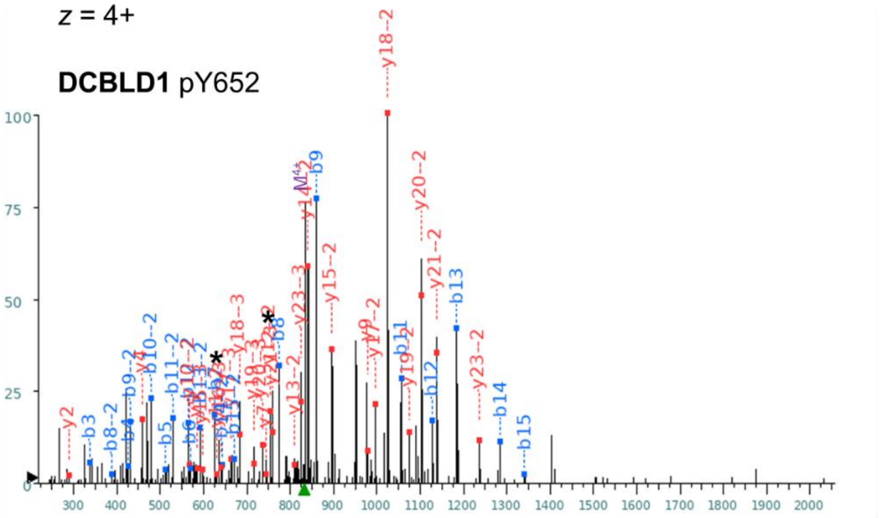

+1

Seq	#	b: Δ Error	b	y	y: Δ Error	+1
H	1	---	138.066	---	---	32
S	2	---	225.098	3194.429	---	31
L	3	**-0.718**	**338.182**	3107.397	---	30
S	4	**0.254**	**425.214**	2994.313	---	29
S	5	**-2.143**	**512.246**	2907.281	---	28
G	6	**-2.214**	**569.268**	2820.249	---	27
G	7	**-1.297**	**626.289**	2763.227	---	26
F	8	**-0.647**	**773.358**	2706.206	---	25
S	9	**-1.132**	**860.390**	2559.137	---	24
P	10	---	957.442	2472.105	---	23
V	11	**0.674**	**1056.511**	2375.052	---	22
A	12	**-0.563**	**1127.548**	2275.984	---	21
G	13	**-0.003**	**1184.569**	2204.947	---	20
V	14	**1.095**	**1283.638**	2147.925	---	19
G	15	**0.245**	**1340.659**	2048.857	---	18
A	16	---	1411.696	1991.836	---	17
Q	17	---	1539.755	1920.798	---	16
D	18	---	1654.782	1792.740	---	15
G	19	---	1711.803	1677.713	---	14
D	20	---	1826.830	1620.691	---	13
Y®	21	---	2069.860	1505.665	---	12
Q	22	---	2197.919	1262.635	---	11
R	23	---	2354.020	1134.576	---	10
P	24	---	2451.072	**978.475**	**1.175**	9
H	25	---	2588.131	881.422	---	8
S	26	---	2675.163	**744.363**	**-1.104**	7
A	27	---	2746.201	657.331	---	6
Q	28	---	2874.259	**586.294**	**2.640**	5
P	29	---	2971.312	**458.236**	**-0.188**	4
A	30	---	3042.349	361.183	---	3
D	31	---	3157.376	**290.146**	**-0.810**	2
R	32	---	---	175.119	---	1

+2

Seq	#	b: Δ Error	b	y	y: Δ Error	+1
H	1	---	69.537	---	---	32
S	2	---	113.053	1597.718	---	31
L	3	---	169.595	1554.202	---	30
S	4	---	213.111	1497.660	---	29
S	5	---	256.627	1454.144	---	28
G	6	---	285.138	1410.628	---	27
G	7	---	313.648	1382.117	---	26
F	8	**1.229**	**387.182**	1353.606	---	25
S	9	**-1.146**	**430.698**	1280.072	---	24
P	10	**-0.997**	**479.225**	**1236.556**	**2.205**	23
V	11	**-0.089**	**528.759**	1188.030	---	22
A	12	**-0.520**	**564.278**	**1138.496**	**-1.535**	21
G	13	**-0.787**	**592.788**	**1102.977**	**-0.254**	20
V	14	**-0.008**	**642.323**	**1074.466**	**0.070**	19
G	15	**2.373**	**670.833**	**1024.932**	**-0.972**	18
A	16	---	706.352	**996.421**	**-1.500**	17
Q	17	---	770.381	960.903	---	16
D	18	---	827.895	**896.874**	**-0.720**	15
G	19	---	856.405	**839.360**	**-0.281**	14
D	20	---	913.919	**810.849**	**-0.680**	13
Y®	21	---	1035.434	**753.336**	**-1.808**	12
Q	22	---	1099.463	**631.821**	**1.125**	11
R	23	---	1177.514	**567.792**	**-2.202**	10
P	24	---	1226.040	489.741	---	9
H	25	---	1294.569	441.215	---	8
S	26	---	1338.085	372.685	---	7
A	27	---	1373.604	329.169	---	6
Q	28	---	1437.633	293.651	---	5
P	29	---	1486.160	229.622	---	4
A	30	---	1521.678	181.095	---	3
D	31	---	1579.192	145.577	---	2
R	32	---	---	88.063	---	1

+3

Seq	#	b: Δ Error	b	y	y: Δ Error	+1
H	1	---	46.694	---	---	32
S	2	---	75.704	1065.481	---	31
L	3	---	113.399	1036.470	---	30
S	4	---	142.410	998.776	---	29
S	5	---	171.420	969.765	---	28
G	6	---	190.427	940.754	---	27
G	7	---	209.435	921.747	---	26
F	8	---	258.457	902.740	---	25
S	9	---	287.468	853.717	---	24
P	10	---	319.819	**824.707**	**0.176**	23
V	11	---	352.842	792.356	---	22
A	12	---	376.521	**759.333**	**-0.924**	21
G	13	---	395.528	**735.654**	**1.233**	20
V	14	---	428.551	**716.647**	**1.966**	19
G	15	---	447.558	**683.624**	**-1.097**	18
A	16	---	471.237	**664.617**	**-0.557**	17
Q	17	---	513.923	**640.938**	**0.695**	16
D	18	---	552.266	**598.251**	**-2.258**	15
G	19	---	571.273	559.909	---	14
D	20	---	609.615	540.902	---	13
Y®	21	---	690.625	502.560	---	12
Q	22	---	733.311	421.550	---	11
R	23	---	785.345	378.864	---	10
P	24	---	817.696	326.830	---	9
H	25	---	863.382	294.479	---	8
S	26	---	892.393	248.793	---	7
A	27	---	916.072	219.782	---	6
Q	28	---	958.758	196.103	---	5
P	29	---	991.109	153.417	---	4
A	30	---	1014.788	121.066	---	3
D	31	---	1053.130	97.387	---	2
R	32	---	---	59.045	---	1

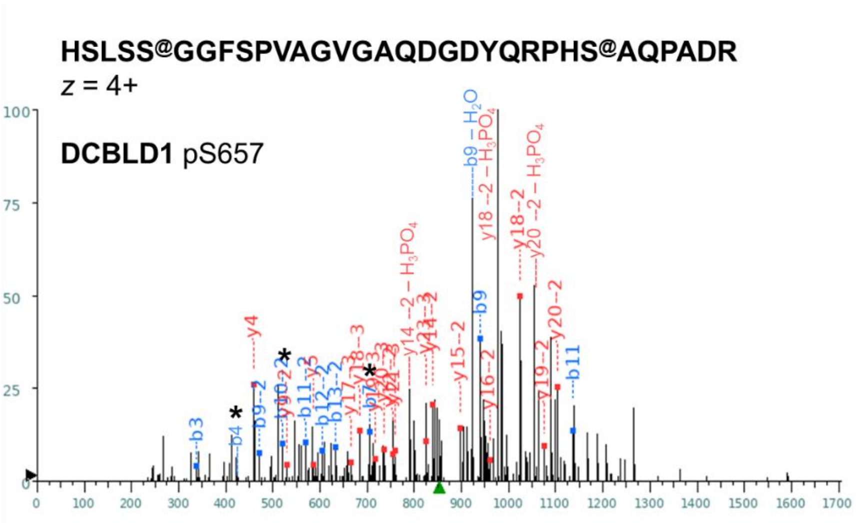

+1						
Seq #	b: Δ Error	b	y	y: Δ Error	+1	
H 1	---	138.066	---	---	32	
S 2	---	225.098	3274.395	---	31	
L 3	-1.440	**338.182**	3187.363	---	30	
S 4	---	425.214	3074.279	---	29	
S● 5	---	592.213	2987.247	---	28	
G 6	---	649.234	2820.249	---	27	
G 7	-1.700	**706.256**	2763.227	---	26	
F 8	---	853.324	2706.206	---	25	
S 9	-1.254	**940.356**	2559.137	---	24	
P 10	---	1037.409	2472.105	---	23	
V 11	-1.111	**1136.477**	2375.052	---	22	
A 12	---	1207.514	2275.984	---	21	
G 13	---	1264.536	2204.947	---	20	
V 14	---	1363.604	2147.925	---	19	
G 15	---	1420.626	2048.857	---	18	
A 16	---	1491.663	1991.836	---	17	
Q 17	---	1619.721	1920.798	---	16	
D 18	---	1734.748	1792.740	---	15	
G 19	---	1791.770	1677.713	---	14	
D 20	---	1906.797	1620.691	---	13	
Y 21	---	2069.860	1505.665	---	12	
Q 22	---	2197.919	1342.601	---	11	
R 23	---	2354.020	1214.543	---	10	
P 24	---	2451.072	1058.441	---	9	
H 25	---	2588.131	961.389	---	8	
S● 26	---	2755.130	824.330	---	7	
A 27	---	2826.167	657.331	---	6	
Q 28	---	2954.225	**586.294**	-0.171	5	
P 29	---	3051.278	**458.236**	-1.320	4	
A 30	---	3122.315	361.183	---	3	
D 31	---	3237.342	290.146	---	2	
R 32	---	---	175.119	---	1	

+2					
Seq #	b: Δ Error	b	y	y: Δ Error	+1
H 1	---	69.537	---	---	32
S 2	---	113.053	1637.701	---	31
L 3	---	169.595	1594.185	---	30
S 4	---	213.111	1537.643	---	29
S● 5	---	296.610	1494.127	---	28
G 6	---	325.121	1410.628	---	27
G 7	---	353.631	1382.117	---	26
F 8	---	427.166	1353.606	---	25
S 9	0.874	**470.682**	1280.072	---	24
P 10	-0.882	**519.208**	1236.556	---	23
V 11	-1.819	**568.742**	1188.030	---	22
A 12	-2.524	**604.261**	1138.496	---	21
G 13	-2.587	**632.772**	**1102.977**	-0.918	20
V 14	---	682.306	**1074.466**	-0.725	19
G 15	---	710.816	**1024.932**	-1.091	18
A 16	---	746.335	996.421	---	17
Q 17	---	810.364	**960.903**	1.559	16
D 18	---	867.878	**896.874**	-1.401	15
G 19	---	896.389	**839.360**	-0.063	14
D 20	---	953.902	810.849	---	13
Y 21	---	1035.434	**753.336**	-2.052	12
Q 22	---	1099.463	671.804	---	11
R 23	---	1177.514	607.775	---	10
P 24	---	1226.040	**529.724**	0.764	9
H 25	---	1294.569	481.198	---	8
S● 26	---	1378.069	412.669	---	7
A 27	---	1413.587	329.169	---	6
Q 28	---	1477.616	293.651	---	5
P 29	---	1526.143	229.622	---	4
A 30	---	1561.661	181.095	---	3
D 31	---	1619.175	145.577	---	2
R 32	---	---	88.063	---	1

+3					
Seq #	b: Δ Error	b	y	y: Δ Error	+1
H 1	---	46.694	---	---	32
S 2	---	75.704	1092.137	---	31
L 3	---	113.399	1063.126	---	30
S 4	---	142.410	1025.431	---	29
S● 5	---	198.076	996.420	---	28
G 6	---	217.083	940.754	---	27
G 7	---	236.090	921.747	---	26
F 8	---	285.113	902.740	---	25
S 9	---	314.124	853.717	---	24
P 10	---	346.474	**824.707**	0.694	23
V 11	---	379.497	792.356	---	22
A 12	---	403.176	**759.333**	-1.486	21
G 13	---	422.183	**735.654**	-0.177	20
V 14	---	455.206	**716.647**	-0.248	19
G 15	---	474.213	**683.624**	0.600	18
A 16	---	497.892	**664.617**	-0.098	17
Q 17	---	540.579	640.938	---	16
D 18	---	578.921	598.251	---	15
G 19	---	597.928	559.909	---	14
D 20	---	636.270	540.902	---	13
Y 21	---	690.625	502.560	---	12
Q 22	---	733.311	448.205	---	11
R 23	---	785.345	405.519	---	10
P 24	---	817.696	353.485	---	9
H 25	---	863.382	321.134	---	8
S● 26	---	919.048	275.448	---	7
A 27	---	942.727	219.782	---	6
Q 28	---	985.413	196.103	---	5
P 29	---	1017.764	153.417	---	4
A 30	---	1041.443	121.066	---	3
D 31	---	1079.786	97.387	---	2
R 32	---	---	59.045	---	1

HS@L[SS]@GGFSPVAGVGAQDGDYQRPHSAQPADR

z = 4+

DCBLD1 pS633, pS[635 or 636]

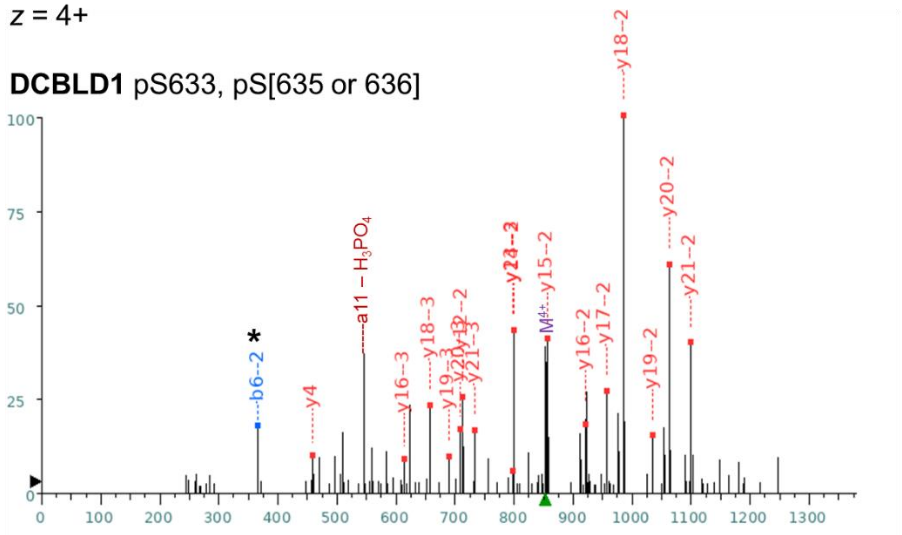

+1

Seq	#	b: Δ Error	b	y	y: Δ Error	+1
H	1	---	138.066	---	---	32
S@	2	---	305.065	3274.395	---	31
L	3	---	418.149	3107.397	---	30
S@	4	---	585.147	2994.313	---	29
S	5	---	672.179	2827.314	---	28
G	6	---	729.200	2740.282	---	27
G	7	---	786.222	2683.261	---	26
F	8	---	933.290	2626.239	---	25
S	9	---	1020.322	2479.171	---	24
P	10	---	1117.375	2392.139	---	23
V	11	---	1216.444	2295.086	---	22
A	12	---	1287.481	2196.018	---	21
G	13	---	1344.502	2124.981	---	20
V	14	---	1443.571	2067.959	---	19
G	15	---	1500.592	1968.891	---	18
A	16	---	1571.629	1911.869	---	17
Q	17	---	1699.688	1840.832	---	16
D	18	---	1814.715	1712.774	---	15
G	19	---	1871.736	1597.747	---	14
D	20	---	1986.763	1540.725	---	13
Y	21	---	2149.826	1425.698	---	12
Q	22	---	2277.885	1262.635	---	11
R	23	---	2433.986	1134.576	---	10
P	24	---	2531.039	978.475	---	9
H	25	---	2668.098	881.422	---	8
S	26	---	2755.130	744.363	---	7
A	27	---	2826.167	657.331	---	6
Q	28	---	2954.225	586.294	---	5
P	29	---	3051.278	**458.236**	1.877	4
A	30	---	3122.315	361.183	---	3
D	31	---	3237.342	290.146	---	2
R	32	---	---	175.119	---	1

+2

Seq	#	b: Δ Error	b	y	y: Δ Error	+1
H	1	---	69.537	---	---	32
S@	2	---	153.036	1637.701	---	31
L	3	---	209.578	1554.202	---	30
S@	4	---	293.077	1497.660	---	29
S	5	---	336.593	1414.161	---	28
G	6	3.964	**365.104**	1370.645	---	27
G	7	---	393.615	1342.134	---	26
F	8	---	467.149	1313.623	---	25
S	9	---	510.665	1240.089	---	24
P	10	---	559.191	1196.573	---	23
V	11	---	608.725	1148.047	---	22
A	12	---	644.244	**1098.512**	-0.470	21
G	13	---	672.755	**1062.994**	-0.254	20
V	14	---	722.289	**1034.483**	-0.389	19
G	15	---	750.800	**984.949**	0.053	18
A	16	---	786.318	**956.438**	-0.211	17
Q	17	---	850.347	**920.920**	-0.548	16
D	18	---	907.861	**856.890**	0.398	15
G	19	---	936.372	**799.377**	0.482	14
D	20	---	993.885	770.866	---	13
Y	21	---	1075.417	**713.353**	0.929	12
Q	22	---	1139.446	631.821	---	11
R	23	---	1217.497	567.792	---	10
P	24	---	1266.023	489.741	---	9
H	25	---	1334.553	441.215	---	8
S	26	---	1378.069	372.685	---	7
A	27	---	1413.587	329.169	---	6
Q	28	---	1477.616	293.651	---	5
P	29	---	1526.143	229.622	---	4
A	30	---	1561.661	181.095	---	3
D	31	---	1619.175	145.577	---	2
R	32	---	---	88.063	---	1

+3

Seq	#	b: Δ Error	b	y	y: Δ Error	+1
H	1	---	46.694	---	---	32
S@	2	---	102.360	1092.137	---	31
L	3	---	140.054	1036.470	---	30
S@	4	---	195.721	998.776	---	29
S	5	---	224.731	943.110	---	28
G	6	---	243.738	914.099	---	27
G	7	---	262.745	895.092	---	26
F	8	---	311.768	876.085	---	25
S	9	---	340.779	827.062	---	24
P	10	---	373.130	**798.051**	0.472	23
V	11	---	406.153	765.700	---	22
A	12	---	429.832	**732.677**	1.274	21
G	13	---	448.839	**708.998**	0.228	20
V	14	---	481.862	**689.991**	0.873	19
G	15	---	500.869	**656.968**	-1.719	18
A	16	---	524.548	637.961	---	17
Q	17	---	567.234	**614.282**	2.889	16
D	18	---	605.576	571.596	---	15
G	19	---	624.584	533.254	---	14
D	20	---	662.926	514.247	---	13
Y	21	---	717.280	475.904	---	12
Q	22	---	759.966	421.550	---	11
R	23	---	812.000	378.864	---	10
P	24	---	844.351	326.830	---	9
H	25	---	890.037	294.479	---	8
S	26	---	919.048	248.793	---	7
A	27	---	942.727	219.782	---	6
Q	28	---	985.413	196.103	---	5
P	29	---	1017.764	153.417	---	4
A	30	---	1041.443	121.066	---	3
D	31	---	1079.786	97.387	---	2
R	32	---	---	59.045	---	1

HSLS@S@GGFSPVAGVGAQDGDYQRPHSAQPADR

z = 4+

DCBLD1 pS635, pS636

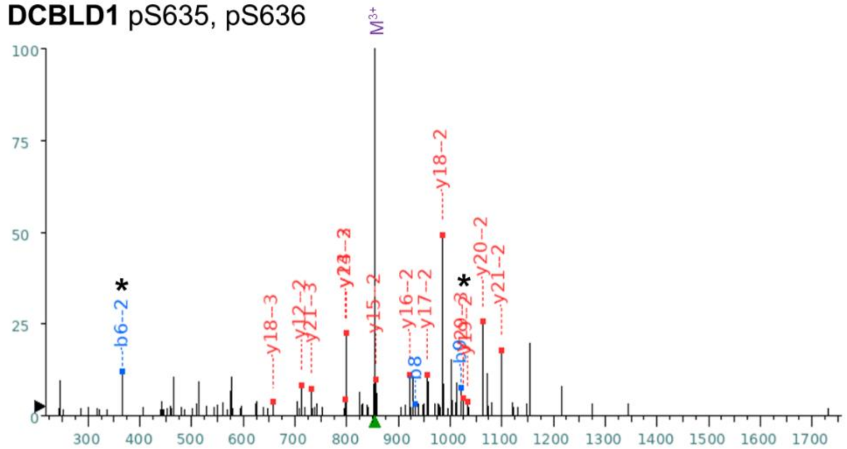

+1

Seq	#	b: Δ Error	b	y	y: Δ Error	+1
H	1	---	138.066	---	---	32
S	2	---	225.098	3274.395	---	31
L	3	---	338.182	3187.363	---	30
S@	4	---	505.181	3074.279	---	29
S@	5	---	672.179	2907.281	---	28
G	6	---	729.200	2740.282	---	27
G	7	---	786.222	2683.261	---	26
F	8	-1.957	**933.290**	2626.239	---	25
S	9	0.199	**1020.322**	2479.171	---	24
P	10	---	1117.375	2392.139	---	23
V	11	---	1216.444	2295.086	---	22
A	12	---	1287.481	2196.018	---	21
G	13	---	1344.502	2124.981	---	20
V	14	---	1443.571	2067.959	---	19
G	15	---	1500.592	1968.891	---	18
A	16	---	1571.629	1911.869	---	17
Q	17	---	1699.688	1840.832	---	16
D	18	---	1814.715	1712.774	---	15
G	19	---	1871.736	1597.747	---	14
D	20	---	1986.763	1540.725	---	13
Y	21	---	2149.826	1425.698	---	12
Q	22	---	2277.885	1262.635	---	11
R	23	---	2433.986	1134.576	---	10
P	24	---	2531.039	978.475	---	9
H	25	---	2668.098	881.422	---	8
S	26	---	2755.130	744.363	---	7
A	27	---	2826.167	657.331	---	6
Q	28	---	2954.225	586.294	---	5
P	29	---	3051.278	458.236	---	4
A	30	---	3122.315	361.183	---	3
D	31	---	3237.342	290.146	---	2
R	32	---	---	175.119	---	1

+2

Seq	#	b: Δ Error	b	y	y: Δ Error	+1
H	1	---	69.537	---	---	32
S	2	---	113.053	1637.701	---	31
L	3	---	169.595	1594.185	---	30
S@	4	---	253.094	1537.643	---	29
S@	5	---	336.593	1454.144	---	28
G	6	0.621	**365.104**	1370.645	---	27
G	7	---	393.615	1342.134	---	26
F	8	---	467.149	1313.623	---	25
S	9	---	510.665	1240.089	---	24
P	10	---	559.191	1196.573	---	23
V	11	---	608.725	1148.047	---	22
A	12	---	644.244	**1098.512**	-0.914	21
G	13	---	672.755	**1062.994**	-0.828	20
V	14	---	722.289	**1034.483**	1.145	19
G	15	---	750.800	**984.949**	-1.000	18
A	16	---	786.318	**956.438**	-0.786	17
Q	17	---	850.347	**920.920**	-0.151	16
D	18	---	907.861	**856.890**	-2.380	15
G	19	---	936.372	**799.377**	-1.503	14
D	20	---	993.885	770.866	---	13
Y	21	---	1075.417	**713.353**	0.073	12
Q	22	---	1139.446	631.821	---	11
R	23	---	1217.497	567.792	---	10
P	24	---	1266.023	489.741	---	9
H	25	---	1334.553	441.215	---	8
S	26	---	1378.069	372.685	---	7
A	27	---	1413.587	329.169	---	6
Q	28	---	1477.616	293.651	---	5
P	29	---	1526.143	229.622	---	4
A	30	---	1561.661	181.095	---	3
D	31	---	1619.175	145.577	---	2
R	32	---	---	88.063	---	1

+3

Seq	#	b: Δ Error	b	y	y: Δ Error	+1
H	1	---	46.694	---	---	32
S	2	---	75.704	1092.137	---	31
L	3	---	113.399	1063.126	---	30
S@	4	---	169.065	**1025.431**	-0.133	29
S@	5	---	224.731	969.765	---	28
G	6	---	243.738	914.099	---	27
G	7	---	262.745	895.092	---	26
F	8	---	311.768	876.085	---	25
S	9	---	340.779	827.062	---	24
P	10	---	373.130	**798.051**	0.319	23
V	11	---	406.153	765.700	---	22
A	12	---	429.832	**732.677**	-0.892	21
G	13	---	448.839	708.998	---	20
V	14	---	481.862	689.991	---	19
G	15	---	500.869	**656.968**	0.697	18
A	16	---	524.548	637.961	---	17
Q	17	---	567.234	614.282	---	16
D	18	---	605.576	571.596	---	15
G	19	---	624.584	533.254	---	14
D	20	---	662.926	514.247	---	13
Y	21	---	717.280	475.904	---	12
Q	22	---	759.966	421.550	---	11
R	23	---	812.000	378.864	---	10
P	24	---	844.351	326.830	---	9
H	25	---	890.037	294.479	---	8
S	26	---	919.048	248.793	---	7
A	27	---	942.727	219.782	---	6
Q	28	---	985.413	196.103	---	5
P	29	---	1017.764	153.417	---	4
A	30	---	1041.443	121.066	---	3
D	31	---	1079.786	97.387	---	2
R	32	---	---	59.045	---	1

GSTFRPMDTDAEEAGVSTDAGGHYDC^PQR

z = 3+

DCBLD1 Y578

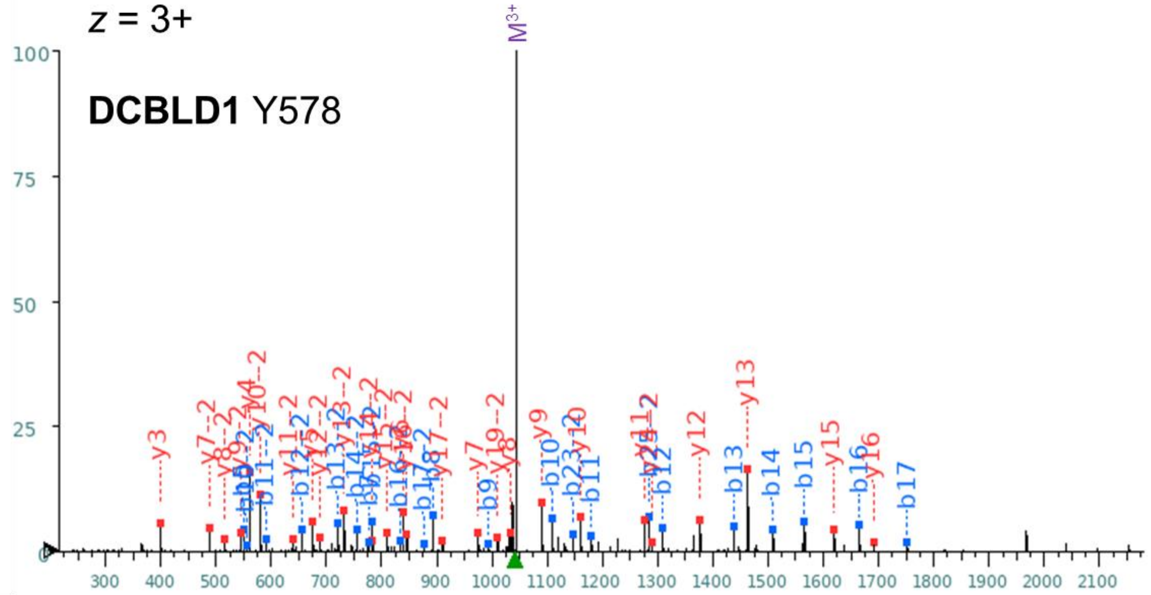

+1

Seq	#	b: Δ Error	b	y	y: Δ Error	+1
G	1	---	58.029	---	---	29
S	2	---	145.061	3070.290	---	28
T	3	---	246.108	2983.258	---	27
F	4	---	393.177	2882.210	---	26
R	5	-0.476	**549.278**	2735.142	---	25
P	6	---	646.331	2579.041	---	24
M	7	-1.811	**777.371**	2481.988	---	23
D	8	-0.034	**892.398**	2350.948	---	22
T	9	-0.410	**993.446**	2235.921	---	21
D	10	-1.107	**1108.473**	2134.873	---	20
A	11	0.198	**1179.510**	2019.846	---	19
E	12	0.559	**1308.552**	1948.809	---	18
E	13	-1.183	**1437.595**	1819.766	---	17
A	14	-1.697	**1508.632**	**1690.724**	0.763	16
G	15	0.094	**1565.654**	**1619.687**	-1.462	15
V	16	-0.678	**1664.722**	1562.665	---	14
S	17	-0.392	**1751.754**	**1463.597**	-0.594	13
T	18	---	1852.802	**1376.565**	-0.598	12
D	19	---	1967.829	**1275.517**	0.367	11
A	20	---	2038.866	**1160.490**	0.584	10
G	21	---	2095.887	**1089.453**	-0.830	9
G	22	---	2152.909	**1032.432**	0.050	8
H	23	---	2289.968	**975.410**	0.157	7
Y	24	---	2453.031	**838.351**	0.125	6
D	25	---	2568.058	**675.288**	-0.245	5
C^	26	---	2728.089	**560.261**	-0.466	4
P	27	---	2825.141	**400.230**	-0.783	3
Q	28	---	2953.200	303.178	---	2
R	29	---	---	175.119	---	1

+2

Seq	#	b: Δ Error	b	y	y: Δ Error	+1
G	1	---	29.518	---	---	29
S	2	---	73.034	1535.649	---	28
T	3	---	123.558	1492.133	---	27
F	4	---	197.092	1441.609	---	26
R	5	---	275.143	1368.075	---	25
P	6	---	323.669	**1290.024**	1.191	24
M	7	---	389.189	1241.498	---	23
D	8	---	446.703	1175.977	---	22
T	9	---	497.227	1118.464	---	21
D	10	1.468	**554.740**	1067.940	---	20
A	11	-0.692	**590.259**	**1010.427**	-2.532	19
E	12	0.595	**654.780**	974.908	---	18
E	13	0.633	**719.301**	**910.387**	-0.534	17
A	14	-2.068	**754.820**	**845.866**	-0.292	16
G	15	0.202	**783.330**	**810.347**	0.075	15
V	16	-0.575	**832.865**	**781.836**	-0.482	14
S	17	-1.827	**876.381**	**732.302**	0.606	13
T	18	---	926.905	**688.786**	-1.537	12
D	19	---	984.418	**638.262**	0.978	11
A	20	---	1019.937	**580.749**	0.100	10
G	21	---	1048.447	**545.230**	0.110	9
G	22	---	1076.958	**516.719**	-0.494	8
H	23	-0.257	**1145.487**	**488.209**	-0.357	7
Y	24	---	1227.019	419.679	---	6
D	25	-0.491	**1284.533**	338.148	---	5
C^	26	---	1364.548	280.634	---	4
P	27	---	1413.074	200.619	---	3
Q	28	---	1477.104	152.092	---	2
R	29	---	---	88.063	---	1

GSTFRPMDTDAEEAGVSTDAGGHY@DC^PQR

z = 3+

DCBLD1 pY578

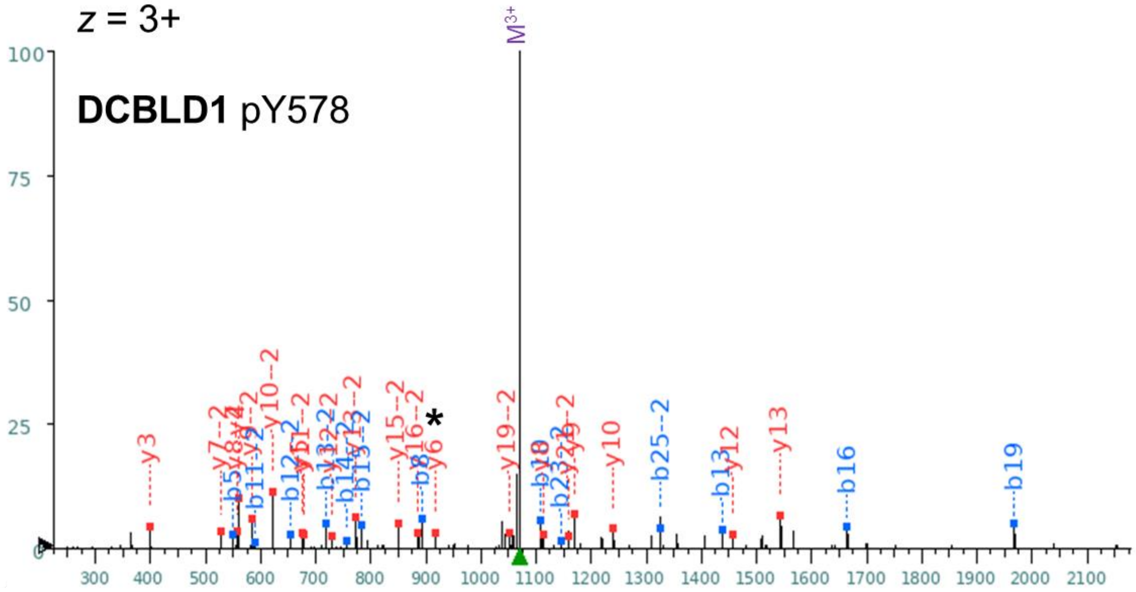

+1

Seq	#	b: Δ Error	b	y	y: Δ Error	+1
G	1	---	58.029	---	---	29
S	2	---	145.061	3150.256	---	28
T	3	---	246.108	3063.224	---	27
F	4	---	393.177	2962.177	---	26
R	5	-0.254	**549.278**	2815.108	---	25
P	6	---	646.331	2659.007	---	24
M	7	---	777.371	2561.954	---	23
D	8	-0.308	**892.398**	2430.914	---	22
T	9	---	993.446	2315.887	---	21
D	10	-0.116	**1108.473**	2214.839	---	20
A	11	---	1179.510	2099.812	---	19
E	12	---	1308.552	2028.775	---	18
E	13	-0.079	**1437.595**	1899.733	---	17
A	14	---	1508.632	1770.690	---	16
G	15	---	1565.654	1699.653	---	15
V	16	0.789	**1664.722**	1642.632	---	14
S	17	---	1751.754	**1543.563**	1.004	13
T	18	---	1852.802	**1456.531**	-1.335	12
D	19	1.245	**1967.829**	1355.483	---	11
A	20	---	2038.866	**1240.456**	-1.931	10
G	21	---	2095.887	**1169.419**	0.982	9
G	22	---	2152.909	**1112.398**	-1.071	8
H	23	---	2289.968	1055.376	---	7
Y@	24	---	2532.997	**918.318**	2.284	6
D	25	---	2648.024	**675.288**	2.557	5
C^	26	---	2808.055	**560.261**	-0.684	4
P	27	---	2905.108	**400.230**	0.208	3
Q	28	---	3033.166	303.178	---	2
R	29	---	---	175.119	---	1

+2

Seq	#	b: Δ Error	b	y	y: Δ Error	+1
G	1	---	29.518	---	---	29
S	2	---	73.034	1575.632	---	28
T	3	---	123.558	1532.116	---	27
F	4	---	197.092	1481.592	---	26
R	5	---	275.143	1408.058	---	25
P	6	---	323.669	1330.007	---	24
M	7	---	389.189	1281.481	---	23
D	8	---	446.703	1215.961	---	22
T	9	---	497.227	**1158.447**	1.876	21
D	10	---	554.740	1107.923	---	20
A	11	-0.278	**590.259**	**1050.410**	0.052	19
E	12	-0.244	**654.780**	1014.891	---	18
E	13	-1.064	**719.301**	950.370	---	17
A	14	3.026	**754.820**	**885.849**	1.363	16
G	15	-1.434	**783.330**	**850.330**	0.561	15
V	16	---	832.865	821.819	---	14
S	17	---	876.381	**772.285**	-0.388	13
T	18	---	926.905	**728.769**	1.547	12
D	19	---	984.418	**678.245**	1.715	11
A	20	---	1019.937	**620.732**	-0.023	10
G	21	---	1048.447	**585.213**	0.084	9
G	22	---	1076.958	**556.703**	0.728	8
H	23	0.275	**1145.487**	**528.192**	-1.448	7
Y@	24	---	1267.002	459.662	---	6
D	25	1.267	**1324.516**	338.148	---	5
C^	26	---	1404.531	280.634	---	4
P	27	---	1453.057	200.619	---	3
Q	28	---	1517.087	152.092	---	2
R	29	---	---	88.063	---	1

AVSALATESGHPDSQKPPTHPGTSDSYSAPR

z = 4+

DCBLD1 S672, S693, S695, Y696, S697

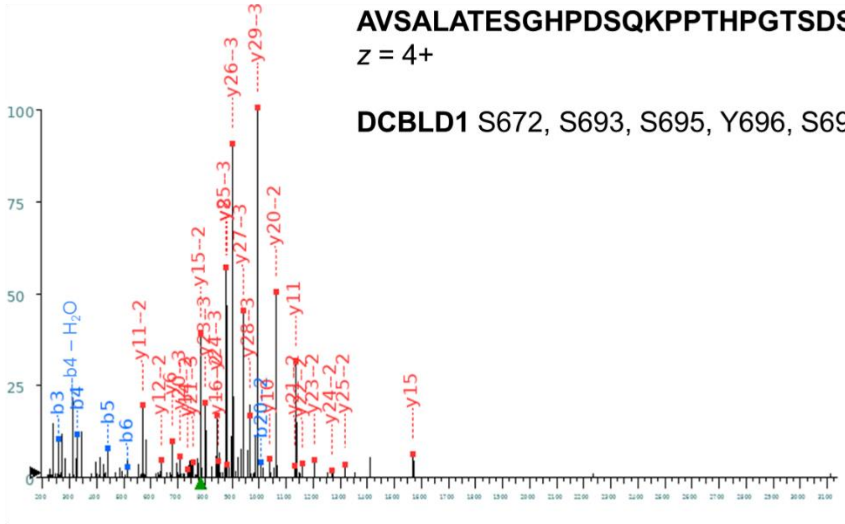

+1

Seq	#	b: Δ Error	b	y	y: Δ Error	+1
A	1	---	72.044	---	---	31
V	2	---	171.113	3077.456	---	30
S	3	-1.402	**258.145**	2978.387	---	29
A	4	-1.762	**329.182**	2891.355	---	28
L	5	-1.422	**442.266**	2820.318	---	27
A	6	-2.066	**513.303**	2707.234	---	26
T	7	---	614.351	2636.197	---	25
E	8	---	743.393	2535.149	---	24
S	9	---	830.425	2406.107	---	23
G	10	---	887.447	2319.075	---	22
H	11	---	1024.506	2262.053	---	21
P	12	---	1121.559	2124.994	---	20
D	13	---	1236.586	2027.942	---	19
S	14	---	1323.618	1912.915	---	18
Q	15	---	1451.676	1825.883	---	17
K	16	---	1579.771	1697.824	---	16
P	17	---	1676.824	**1569.729**	1.268	15
P	18	---	1773.877	1472.676	---	14
T	19	---	1874.924	1375.624	---	13
H	20	---	2011.983	1274.576	---	12
P	21	---	2109.036	**1137.517**	-1.612	11
G	22	---	2166.057	**1040.464**	1.550	10
T	23	---	2267.105	983.443	---	9
S	24	---	2354.137	**882.395**	-0.674	8
D	25	---	2469.164	795.363	---	7
S	26	---	2556.196	**680.336**	-0.498	6
Y	27	---	2719.259	593.304	---	5
S	28	---	2806.291	430.241	---	4
A	29	---	2877.329	343.209	---	3
P	30	---	2974.381	272.172	---	2
R	31	---	---	175.119	---	1

+2

Seq	#	b: Δ Error	b	y	y: Δ Error	+1
A	1	---	36.526	---	---	31
V	2	---	86.060	1539.232	---	30
S	3	---	129.576	1489.697	---	29
A	4	---	165.095	1446.181	---	28
L	5	---	221.637	1410.663	---	27
A	6	---	257.155	1354.121	---	26
T	7	---	307.679	**1318.602**	-0.313	25
E	8	---	372.200	**1268.078**	0.473	24
S	9	---	415.716	**1203.557**	-2.295	23
G	10	---	444.227	**1160.041**	-0.467	22
H	11	---	512.757	**1131.530**	-1.783	21
P	12	---	561.283	**1063.001**	-0.944	20
D	13	---	618.796	1014.474	---	19
S	14	---	662.312	956.961	---	18
Q	15	---	726.342	913.445	---	17
K	16	---	790.389	**849.416**	-0.736	16
P	17	---	838.916	**785.368**	-1.111	15
P	18	---	887.442	**736.842**	2.149	14
T	19	---	937.966	688.315	---	13
H	20	-2.300	**1006.495**	**637.792**	1.126	12
P	21	---	1055.022	**569.262**	-0.497	11
G	22	---	1083.532	520.736	---	10
T	23	---	1134.056	492.225	---	9
S	24	---	1177.572	441.701	---	8
D	25	---	1235.086	398.185	---	7
S	26	---	1278.602	340.672	---	6
Y	27	---	1360.133	297.156	---	5
S	28	---	1403.649	215.624	---	4
A	29	---	1439.168	172.108	---	3
P	30	---	1487.694	136.589	---	2
R	31	---	---	88.063	---	1

+3

Seq	#	b: Δ Error	b	y	y: Δ Error	+1
A	1	---	24.686	---	---	31
V	2	---	57.709	1026.490	---	30
S	3	---	86.720	**993.467**	-0.797	29
A	4	---	110.399	**964.457**	-0.573	28
L	5	---	148.094	**940.778**	-0.954	27
A	6	---	171.773	**903.083**	-1.009	26
T	7	---	205.455	**879.404**	-0.595	25
E	8	---	248.469	**845.721**	-0.615	24
S	9	---	277.480	**802.707**	-1.818	23
G	10	---	296.487	773.696	---	22
H	11	---	342.173	**754.689**	-0.224	21
P	12	---	374.524	**709.003**	-2.184	20
D	13	---	412.867	676.652	---	19
S	14	---	441.877	638.310	---	18
Q	15	---	484.564	609.299	---	17
K	16	---	527.262	566.613	---	16
P	17	---	559.613	523.915	---	15
P	18	---	591.964	491.564	---	14
T	19	---	625.646	459.213	---	13
H	20	---	671.333	425.530	---	12
P	21	---	703.684	379.844	---	11
G	22	---	722.691	347.493	---	10
T	23	---	756.373	328.486	---	9
S	24	---	785.384	294.803	---	8
D	25	---	823.726	265.793	---	7
S	26	---	852.737	227.450	---	6
Y	27	---	907.091	198.440	---	5
S	28	---	936.102	144.085	---	4
A	29	---	959.781	115.074	---	3
P	30	---	992.132	91.395	---	2
R	31	---	---	59.045	---	1

AVS@ALATESGHPDSQKPPTHPGTSDSYSAPR

z = 4+

DCBLD1 pS672

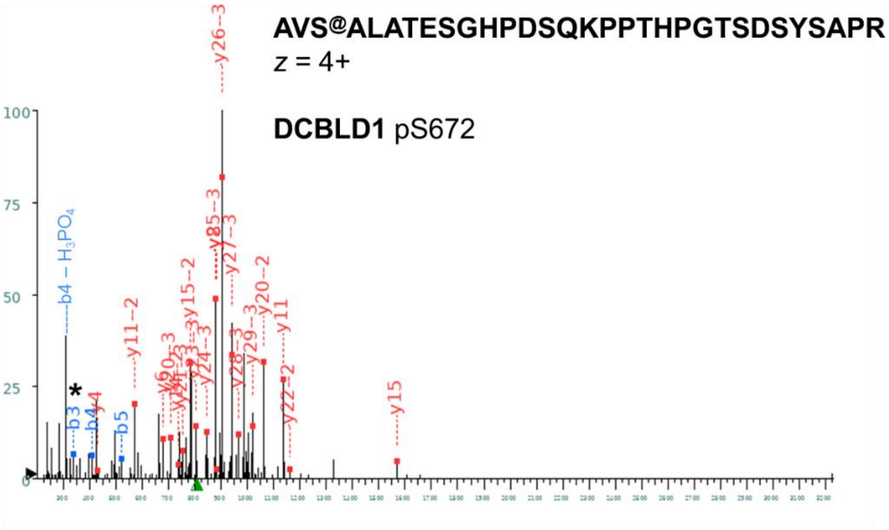

+1

Seq	#	b: Δ Error	b	y	y: Δ Error	+1
A	1	---	72.044	---	---	31
V	2	---	171.113	3157.422	---	30
S@	3	-1.496	**338.111**	3058.354	---	29
A	4	-0.352	**409.148**	2891.355	---	28
L	5	0.624	**522.232**	2820.318	---	27
A	6	---	593.269	2707.234	---	26
T	7	---	694.317	2636.197	---	25
E	8	---	823.360	2535.149	---	24
S	9	---	910.392	2406.107	---	23
G	10	---	967.413	2319.075	---	22
H	11	---	1104.472	2262.053	---	21
P	12	---	1201.525	2124.994	---	20
D	13	---	1316.552	2027.942	---	19
S	14	---	1403.584	1912.915	---	18
Q	15	---	1531.642	1825.883	---	17
K	16	---	1659.737	1697.824	---	16
P	17	---	1756.790	**1569.729**	-0.910	15
P	18	---	1853.843	1472.676	---	14
T	19	---	1954.891	1375.624	---	13
H	20	---	2091.950	1274.576	---	12
P	21	---	2189.002	**1137.517**	-1.076	11
G	22	---	2246.024	1040.464	---	10
T	23	---	2347.071	983.443	---	9
S	24	---	2434.103	**882.395**	2.923	8
D	25	---	2549.130	795.363	---	7
S	26	---	2636.162	**680.336**	-1.485	6
Y	27	---	2799.226	593.304	---	5
S	28	---	2886.258	**430.241**	-0.529	4
A	29	---	2957.295	343.209	---	3
P	30	---	3054.348	272.172	---	2
R	31	---	---	175.119	---	1

+2

Seq	#	b: Δ Error	b	y	y: Δ Error	+1
A	1	---	36.526	---	---	31
V	2	---	86.060	1579.215	---	30
S@	3	---	169.559	1529.681	---	29
A	4	---	205.078	1446.181	---	28
L	5	---	261.620	1410.663	---	27
A	6	---	297.138	1354.121	---	26
T	7	---	347.662	1318.602	---	25
E	8	---	412.184	1268.078	---	24
S	9	---	455.700	1203.557	---	23
G	10	---	484.210	**1160.041**	-1.940	22
H	11	---	552.740	1131.530	---	21
P	12	---	601.266	**1063.001**	-1.059	20
D	13	---	658.780	1014.474	---	19
S	14	---	702.296	956.961	---	18
Q	15	---	766.325	913.445	---	17
K	16	---	830.372	849.416	---	16
P	17	---	878.899	**785.368**	-0.490	15
P	18	---	927.425	**736.842**	2.728	14
T	19	---	977.949	688.315	---	13
H	20	---	1046.478	637.792	---	12
P	21	---	1095.005	**569.262**	-0.175	11
G	22	---	1123.516	520.736	---	10
T	23	---	1174.039	492.225	---	9
S	24	---	1217.555	441.701	---	8
D	25	---	1275.069	398.185	---	7
S	26	---	1318.585	340.672	---	6
Y	27	---	1400.117	297.156	---	5
S	28	---	1443.633	215.624	---	4
A	29	---	1479.151	172.108	---	3
P	30	---	1527.677	136.589	---	2
R	31	---	---	88.063	---	1

+3

Seq	#	b: Δ Error	b	y	y: Δ Error	+1
A	1	---	24.686	---	---	31
V	2	---	57.709	1053.146	---	30
S@	3	---	113.375	**1020.123**	-1.003	29
A	4	---	137.054	**964.457**	-0.763	28
L	5	---	174.749	**940.778**	-0.046	27
A	6	---	198.428	**903.083**	-0.469	26
T	7	---	232.111	**879.404**	0.099	25
E	8	---	275.125	**845.721**	-0.182	24
S	9	---	304.135	**802.707**	-3.643	23
G	10	---	323.143	773.696	---	22
H	11	---	368.829	**754.689**	2.930	21
P	12	---	401.180	**709.003**	1.088	20
D	13	---	439.522	676.652	---	19
S	14	---	468.533	638.310	---	18
Q	15	---	511.219	609.299	---	17
K	16	---	553.917	566.613	---	16
P	17	---	586.268	523.915	---	15
P	18	---	618.619	491.564	---	14
T	19	---	652.302	459.213	---	13
H	20	---	697.988	425.530	---	12
P	21	---	730.339	379.844	---	11
G	22	---	749.346	347.493	---	10
T	23	---	783.029	328.486	---	9
S	24	---	812.039	294.803	---	8
D	25	---	850.382	265.793	---	7
S	26	---	879.392	227.450	---	6
Y	27	---	933.747	198.440	---	5
S	28	---	962.757	144.085	---	4
A	29	---	986.436	115.074	---	3
P	30	---	1018.787	91.395	---	2
R	31	---	---	59.045	---	1

AVSALATESGHPDSQKPPTHPGTSDS@YSAPR

z = 4+

DCBLD1 pS695

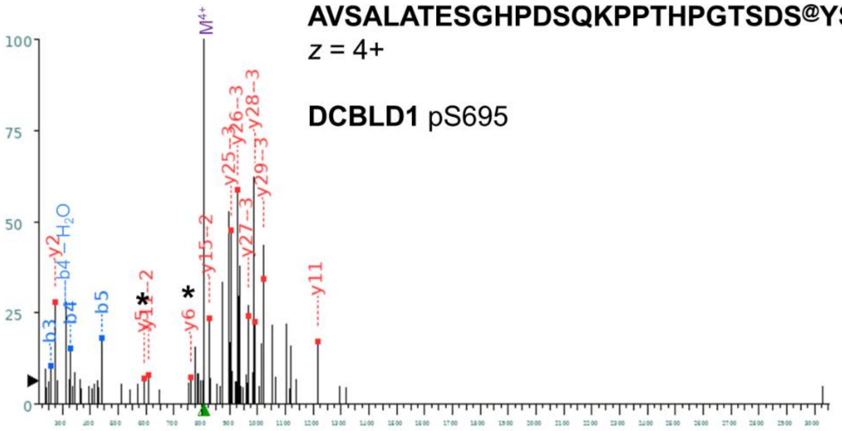

+1						
Seq #	b: Δ Error	b	y	y: Δ Error	+1	
A 1	---	72.044	---	---	31	
V 2	---	171.113	3157.422	---	30	
S 3	-2.821	**258.145**	3058.354	---	29	
A 4	-3.152	**329.182**	2971.322	---	28	
L 5	-1.008	**442.266**	2900.285	---	27	
A 6	---	513.303	2787.201	---	26	
T 7	---	614.351	2716.163	---	25	
E 8	---	743.393	2615.116	---	24	
S 9	---	830.425	2486.073	---	23	
G 10	---	887.447	2399.041	---	22	
H 11	---	1024.506	2342.020	---	21	
P 12	---	1121.559	2204.961	---	20	
D 13	---	1236.586	2107.908	---	19	
S 14	---	1323.618	1992.881	---	18	
Q 15	---	1451.676	1905.849	---	17	
K 16	---	1579.771	1777.791	---	16	
P 17	---	1676.824	1649.696	---	15	
P 18	---	1773.877	1552.643	---	14	
T 19	---	1874.924	1455.590	---	13	
H 20	---	2011.983	1354.542	---	12	
P 21	---	2109.036	**1217.483**	1.383	11	
G 22	---	2166.057	1120.431	---	10	
T 23	---	2267.105	1063.409	---	9	
S 24	---	2354.137	962.362	---	8	
D 25	---	2469.164	875.329	---	7	
S@ 26	---	2636.162	**760.303**	-2.562	6	
Y 27	---	2799.226	**593.304**	-1.111	5	
S 28	---	2886.258	430.241	---	4	
A 29	---	2957.295	343.209	---	3	
P 30	---	3054.348	**272.172**	2.265	2	
R 31	---	---	175.119	---	1	

+2					
Seq #	b: Δ Error	b	y	y: Δ Error	+1
A 1	---	36.526	---	---	31
V 2	---	86.060	1579.215	---	30
S 3	---	129.576	1529.681	---	29
A 4	---	165.095	1486.165	---	28
L 5	---	221.637	1450.646	---	27
A 6	---	257.155	1394.104	---	26
T 7	---	307.679	1358.585	---	25
E 8	---	372.200	1308.062	---	24
S 9	---	415.716	1243.540	---	23
G 10	---	444.227	1200.024	---	22
H 11	---	512.757	1171.514	---	21
P 12	---	561.283	1102.984	---	20
D 13	---	618.796	1054.458	---	19
S 14	---	662.312	996.944	---	18
Q 15	---	726.342	953.428	---	17
K 16	---	790.389	889.399	---	16
P 17	---	838.916	**825.351**	1.222	15
P 18	---	887.442	776.825	---	14
T 19	---	937.966	728.299	---	13
H 20	---	1006.495	677.775	---	12
P 21	---	1055.022	**609.245**	-0.181	11
G 22	---	1083.532	560.719	---	10
T 23	---	1134.056	532.208	---	9
S 24	---	1177.572	481.684	---	8
D 25	---	1235.086	438.168	---	7
S@ 26	---	1318.585	380.655	---	6
Y 27	---	1400.117	297.156	---	5
S 28	---	1443.633	215.624	---	4
A 29	---	1479.151	172.108	---	3
P 30	---	1527.677	136.589	---	2
R 31	---	---	88.063	---	1

+3					
Seq #	b: Δ Error	b	y	y: Δ Error	+1
A 1	---	24.686	---	---	31
V 2	---	57.709	1053.146	---	30
S 3	---	86.720	**1020.123**	-1.960	29
A 4	---	110.399	**991.112**	-1.099	28
L 5	---	148.094	**967.433**	-1.229	27
A 6	---	171.773	**929.738**	0.084	26
T 7	---	205.455	**906.059**	-1.978	25
E 8	---	248.469	872.377	---	24
S 9	---	277.480	829.363	---	23
G 10	---	296.487	800.352	---	22
H 11	---	342.173	781.345	---	21
P 12	---	374.524	735.658	---	20
D 13	---	412.867	703.308	---	19
S 14	---	441.877	664.965	---	18
Q 15	---	484.564	635.955	---	17
K 16	---	527.262	593.268	---	16
P 17	---	559.613	550.570	---	15
P 18	---	591.964	518.219	---	14
T 19	---	625.646	485.868	---	13
H 20	---	671.333	452.186	---	12
P 21	---	703.684	406.499	---	11
G 22	---	722.691	374.148	---	10
T 23	---	756.373	355.141	---	9
S 24	---	785.384	321.459	---	8
D 25	---	823.726	292.448	---	7
S@ 26	---	879.392	254.106	---	6
Y 27	---	933.747	198.440	---	5
S 28	---	962.757	144.085	---	4
A 29	---	986.436	115.074	---	3
P 30	---	1018.787	91.395	---	2
R 31	---	---	59.045	---	1

AVSALATESGHPDSQKPPTHPGTSD[SYS]@APR

z = 4+

DCBLD1 p[S695, Y696, or S697]

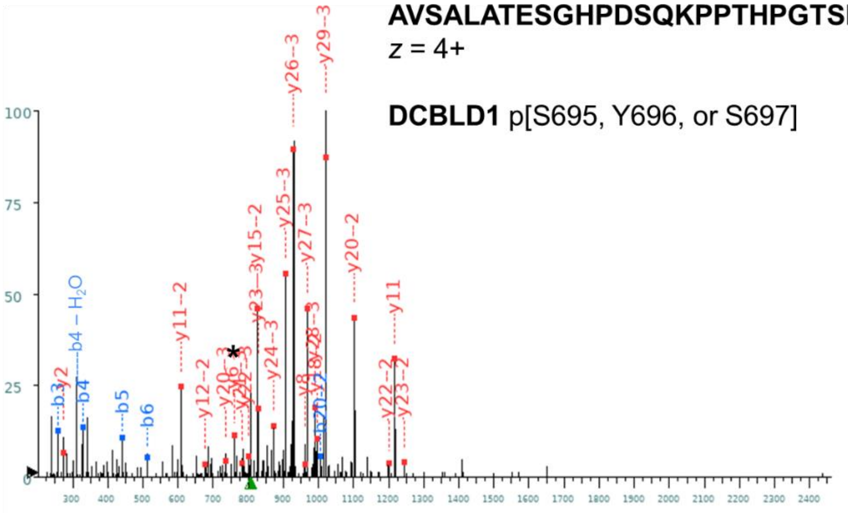

+1

Seq	#	b: Δ Error	b	y	y: Δ Error	+1
A	1	---	72.044	---	---	31
V	2	---	171.113	3157.422	---	30
S	3	-1.165	**258.145**	3058.354	---	29
A	4	-0.835	**329.182**	2971.322	---	28
L	5	-1.353	**442.266**	2900.285	---	27
A	6	-3.018	**513.303**	2787.201	---	26
T	7	---	614.351	2716.163	---	25
E	8	---	743.393	2615.116	---	24
S	9	---	830.425	2486.073	---	23
G	10	---	887.447	2399.041	---	22
H	11	---	1024.506	2342.020	---	21
P	12	---	1121.559	2204.961	---	20
D	13	---	1236.586	2107.908	---	19
S	14	---	1323.618	1992.881	---	18
Q	15	---	1451.676	1905.849	---	17
K	16	---	1579.771	1777.791	---	16
P	17	---	1676.824	1649.696	---	15
P	18	---	1773.877	1552.643	---	14
T	19	---	1874.924	1455.590	---	13
H	20	---	2011.983	1354.542	---	12
P	21	---	2109.036	**1217.483**	-1.224	11
G	22	---	2166.057	1120.431	---	10
T	23	---	2267.105	1063.409	---	9
S	24	---	2354.137	**962.362**	-3.051	8
D	25	---	2469.164	875.329	---	7
S	26	---	2556.196	**760.303**	-0.635	6
Y®	27	---	2799.226	673.271	---	5
S	28	---	2886.258	430.241	---	4
A	29	---	2957.295	343.209	---	3
P	30	---	3054.348	**272.172**	7.647	2
R	31	---	---	175.119	---	1

+2

Seq	#	b: Δ Error	b	y	y: Δ Error	+1
A	1	---	36.526	---	---	31
V	2	---	86.060	1579.215	---	30
S	3	---	129.576	1529.681	---	29
A	4	---	165.095	1486.165	---	28
L	5	---	221.637	1450.646	---	27
A	6	---	257.155	1394.104	---	26
T	7	---	307.679	1358.585	---	25
E	8	---	372.200	1308.062	---	24
S	9	---	415.716	**1243.540**	2.286	23
G	10	---	444.227	**1200.024**	-0.461	22
H	11	---	512.757	1171.514	---	21
P	12	---	561.283	**1102.984**	-0.920	20
D	13	---	618.796	1054.458	---	19
S	14	---	662.312	**996.944**	-0.714	18
Q	15	---	726.342	953.428	---	17
K	16	---	790.389	889.399	---	16
P	17	---	838.916	**825.351**	-2.402	15
P	18	---	887.442	776.825	---	14
T	19	---	937.966	728.299	---	13
H	20	-2.967	**1006.495**	**677.775**	-1.478	12
P	21	---	1055.022	**609.245**	-1.584	11
G	22	---	1083.532	560.719	---	10
T	23	---	1134.056	532.208	---	9
S	24	---	1177.572	481.684	---	8
D	25	---	1235.086	438.168	---	7
S	26	---	1278.602	380.655	---	6
Y®	27	---	1400.117	337.139	---	5
S	28	---	1443.633	215.624	---	4
A	29	---	1479.151	172.108	---	3
P	30	---	1527.677	136.589	---	2
R	31	---	---	88.063	---	1

+3

Seq	#	b: Δ Error	b	y	y: Δ Error	+1
A	1	---	24.686	---	---	31
V	2	---	57.709	1053.146	---	30
S	3	---	86.720	**1020.123**	-1.242	29
A	4	---	110.399	**991.112**	-1.961	28
L	5	---	148.094	**967.433**	-0.535	27
A	6	---	171.773	**929.738**	-1.098	26
T	7	---	205.455	**906.059**	-0.967	25
E	8	---	248.469	**872.377**	-0.022	24
S	9	---	277.480	**829.363**	-1.744	23
G	10	---	296.487	**800.352**	-3.643	22
H	11	---	342.173	**781.345**	-1.762	21
P	12	---	374.524	**735.658**	2.891	20
D	13	---	412.867	703.308	---	19
S	14	---	441.877	664.965	---	18
Q	15	---	484.564	635.955	---	17
K	16	---	527.262	593.268	---	16
P	17	---	559.613	550.570	---	15
P	18	---	591.964	518.219	---	14
T	19	---	625.646	485.868	---	13
H	20	---	671.333	452.186	---	12
P	21	---	703.684	406.499	---	11
G	22	---	722.691	374.148	---	10
T	23	---	756.373	355.141	---	9
S	24	---	785.384	321.459	---	8
D	25	---	823.726	292.448	---	7
S	26	---	852.737	254.106	---	6
Y®	27	---	933.747	225.095	---	5
S	28	---	962.757	144.085	---	4
A	29	---	986.436	115.074	---	3
P	30	---	1018.787	91.395	---	2
R	31	---	---	59.045	---	1

SGHPDSQKPPTHPGTSDSYSAPR
z = 3+

DCBLD1 S693, Y696

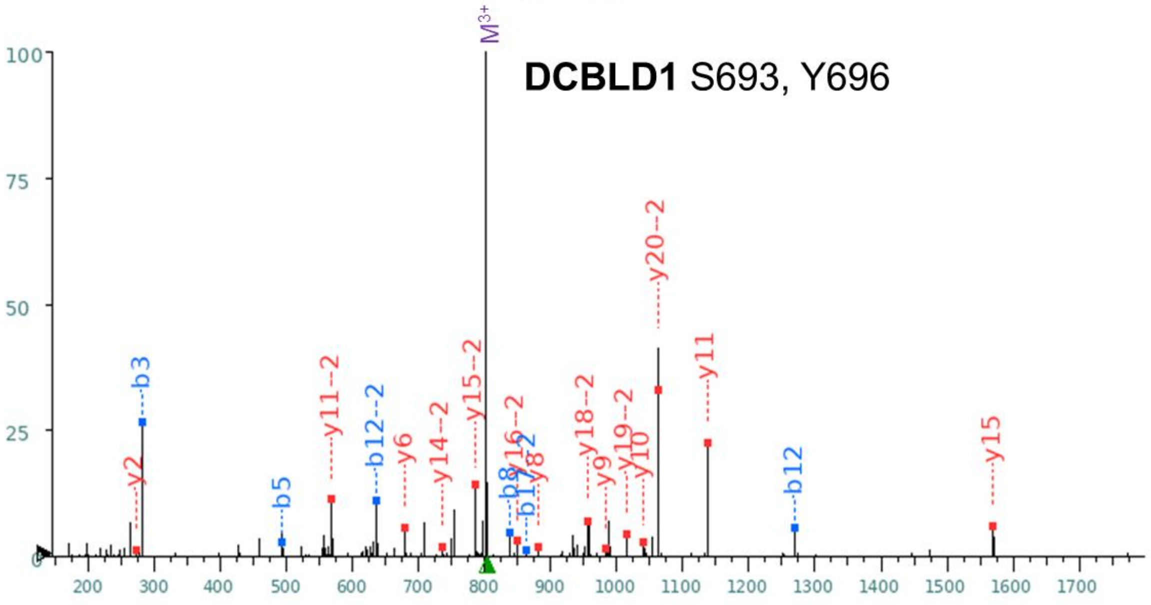

+1

Seq	#	b: Δ Error	b	y	y: Δ Error	+1
S	1	---	88.039	---	---	23
G	2	---	145.061	2319.075	---	22
H	3	-0.397	**282.120**	2262.053	---	21
P	4	---	379.172	2124.994	---	20
D	5	0.030	**494.199**	2027.942	---	19
S	6	---	581.231	1912.915	---	18
Q	7	---	709.290	1825.883	---	17
K	8	3.126	**837.385**	1697.824	---	16
P	9	---	934.438	**1569.729**	0.723	15
P	10	---	1031.490	1472.676	---	14
T	11	---	1132.538	1375.624	---	13
H	12	-1.368	**1269.597**	1274.576	---	12
P	13	---	1366.650	**1137.517**	-0.969	11
G	14	---	1423.671	**1040.464**	-1.735	10
T	15	---	1524.719	**983.443**	2.302	9
S	16	---	1611.751	**882.395**	0.986	8
D	17	---	1726.778	795.363	---	7
S	18	---	1813.810	**680.336**	1.027	6
Y	19	---	1976.873	593.304	---	5
S	20	---	2063.905	430.241	---	4
A	21	---	2134.942	343.209	---	3
P	22	---	2231.995	**272.172**	-0.651	2
R	23	---	---	175.119	---	1

+2

Seq	#	b: Δ Error	b	y	y: Δ Error	+1
S	1	---	44.523	---	---	23
G	2	---	73.034	1160.041	---	22
H	3	---	141.563	1131.530	---	21
P	4	---	190.090	**1063.001**	-0.600	20
D	5	---	247.603	**1014.474**	-1.516	19
S	6	---	291.119	**956.961**	0.607	18
Q	7	---	355.149	913.445	---	17
K	8	---	419.196	**849.416**	-1.814	16
P	9	---	467.722	**785.368**	-1.267	15
P	10	---	516.249	**736.842**	-2.242	14
T	11	---	566.773	688.315	---	13
H	12	-0.368	**635.302**	637.792	---	12
P	13	---	683.829	**569.262**	-0.925	11
G	14	---	712.339	520.736	---	10
T	15	---	762.863	492.225	---	9
S	16	---	806.379	441.701	---	8
D	17	-0.748	**863.893**	398.185	---	7
S	18	---	907.409	340.672	---	6
Y	19	---	988.940	297.156	---	5
S	20	---	1032.456	215.624	---	4
A	21	---	1067.975	172.108	---	3
P	22	---	1116.501	136.589	---	2
R	23	---	---	88.063	---	1

SGHPDSQKPPTHPGTS@DSYSAPR
z = 3+

DCBLD1 pS693

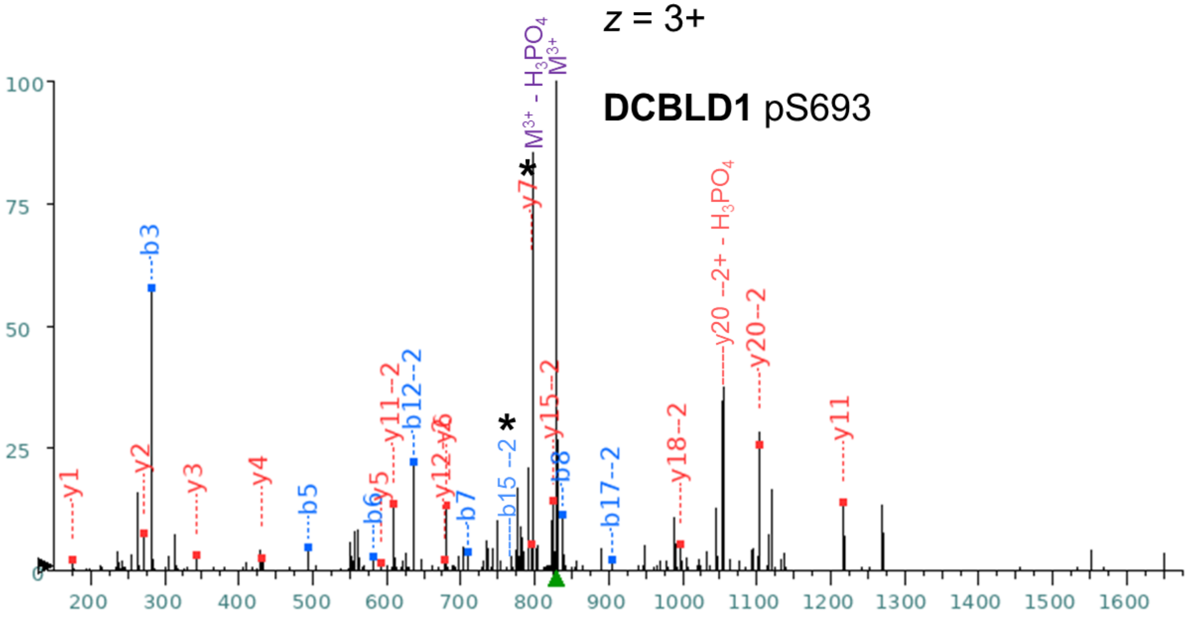

Seq	#	b: Δ Error	b	y	y: Δ Error	+1
			**+1**			
S	1	---	88.039	---	---	23
G	2	---	145.061	2399.041	---	22
H	3	-1.696	**282.120**	2342.020	---	21
P	4	---	379.172	2204.961	---	20
D	5	-1.328	**494.199**	2107.908	---	19
S	6	1.942	**581.231**	1992.881	---	18
Q	7	-2.344	**709.290**	1905.849	---	17
K	8	-3.216	**837.385**	1777.791	---	16
P	9	---	934.438	1649.696	---	15
P	10	---	1031.490	1552.643	---	14
T	11	---	1132.538	1455.590	---	13
H	12	---	1269.597	1354.542	---	12
P	13	---	1366.650	**1217.483**	-1.123	11
G	14	---	1423.671	1120.431	---	10
T	15	---	1524.719	1063.409	---	9
S@	16	---	1691.717	962.362	---	8
D	17	---	1806.744	**795.363**	3.454	7
S	18	---	1893.776	**680.336**	-1.396	6
Y	19	---	2056.840	**593.304**	0.226	5
S	20	---	2143.872	**430.241**	-1.380	4
A	21	---	2214.909	**343.209**	-2.307	3
P	22	---	2311.962	**272.172**	-1.099	2
R	23	---	---	**175.119**	-2.150	1

Seq	#	b: Δ Error	b	y	y: Δ Error	+1
			**+2**			
S	1	---	44.523	---	---	23
G	2	---	73.034	1200.024	---	22
H	3	---	141.563	1171.514	---	21
P	4	---	190.090	**1102.984**	-0.477	20
D	5	---	247.603	1054.458	---	19
S	6	---	291.119	**996.944**	1.919	18
Q	7	---	355.149	953.428	---	17
K	8	---	419.196	889.399	---	16
P	9	---	467.722	**825.351**	-1.810	15
P	10	---	516.249	776.825	---	14
T	11	---	566.773	728.299	---	13
H	12	-2.194	**635.302**	**677.775**	-2.109	12
P	13	---	683.829	**609.245**	-1.484	11
G	14	---	712.339	560.719	---	10
T	15	---	762.863	532.208	---	9
S@	16	---	846.362	481.684	---	8
D	17	0.691	**903.876**	398.185	---	7
S	18	---	947.392	340.672	---	6
Y	19	---	1028.923	297.156	---	5
S	20	---	1072.439	215.624	---	4
A	21	---	1107.958	172.108	---	3
P	22	---	1156.484	136.589	---	2
R	23	---	---	88.063	---	1

SGHPDSQKPPTHPGTS@DSY@SAPR
z = 3+

DCBLD1 pS693. pY696

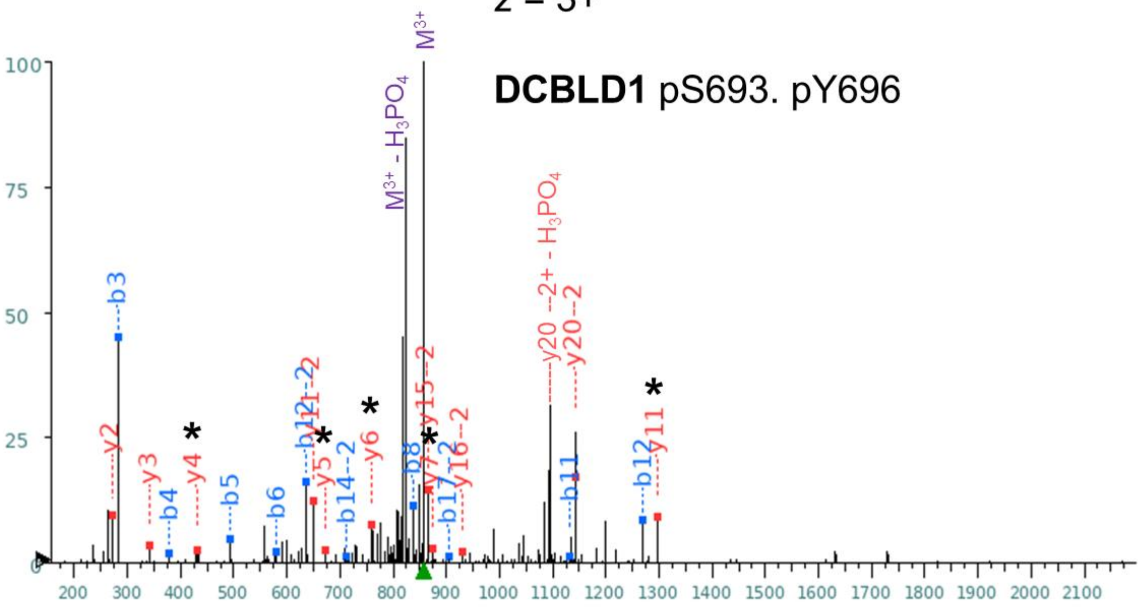

			+1			
Seq	#	b: Δ Error	b	y	y: Δ Error	+1
S	1	---	88.039	---	---	23
G	2	---	145.061	2479.008	---	22
H	3	-0.938	**282.120**	2421.986	---	21
P	4	2.202	**379.172**	2284.927	---	20
D	5	0.277	**494.199**	2187.874	---	19
S	6	2.572	**581.231**	2072.847	---	18
Q	7	---	709.290	1985.815	---	17
K	8	-1.248	**837.385**	1857.757	---	16
P	9	---	934.438	1729.662	---	15
P	10	---	1031.490	1632.609	---	14
T	11	-2.115	**1132.538**	1535.556	---	13
H	12	-1.080	**1269.597**	1434.509	---	12
P	13	---	1366.650	**1297.450**	-0.883	11
G	14	---	1423.671	1200.397	---	10
T	15	---	1524.719	1143.376	---	9
S@	16	---	1691.717	1042.328	---	8
D	17	---	1806.744	**875.329**	0.603	7
S	18	---	1893.776	**760.303**	0.328	6
Y@	19	---	2136.806	**673.271**	-1.556	5
S	20	---	2223.838	**430.241**	1.954	4
A	21	---	2294.875	**343.209**	-1.062	3
P	22	---	2391.928	**272.172**	-0.763	2
R	23	---	---	175.119	---	1

			+2			
Seq	#	b: Δ Error	b	y	y: Δ Error	+1
S	1	---	44.523	---	---	23
G	2	---	73.034	1240.007	---	22
H	3	---	141.563	1211.497	---	21
P	4	---	190.090	**1142.967**	0.064	20
D	5	---	247.603	1094.441	---	19
S	6	---	291.119	1036.927	---	18
Q	7	---	355.149	993.411	---	17
K	8	---	419.196	**929.382**	1.143	16
P	9	---	467.722	**865.335**	-1.105	15
P	10	---	516.249	816.808	---	14
T	11	---	566.773	768.282	---	13
H	12	-0.080	**635.302**	717.758	---	12
P	13	---	683.829	**649.229**	-0.845	11
G	14	-0.335	**712.339**	600.702	---	10
T	15	---	762.863	572.191	---	9
S@	16	---	846.362	521.668	---	8
D	17	1.772	**903.876**	438.168	---	7
S	18	---	947.392	380.655	---	6
Y@	19	---	1068.907	337.139	---	5
S	20	---	1112.423	215.624	---	4
A	21	---	1147.941	172.108	---	3
P	22	---	1196.468	136.589	---	2
R	23	---	---	88.063	---	1

AVDHEETPVRYS@SSEVNHLSPR
z = 3+

DCBLD2 pS598

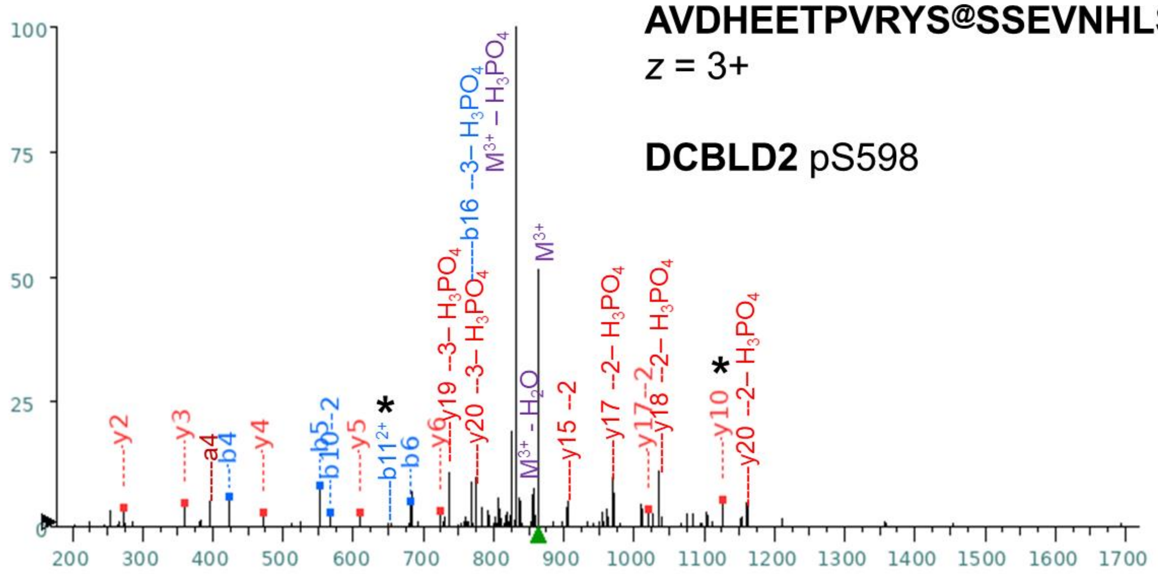

+1						
Seq	#	b: Δ Error	b	y	y: Δ Error	+1
A	1	---	72.044	---	---	22
V	2	---	171.113	2518.136	---	21
D	3	---	286.140	2419.067	---	20
H	4	-4.229	**423.199**	2304.040	---	19
E	5	0.921	**552.241**	2166.982	---	18
E	6	0.581	**681.284**	2037.939	---	17
T	7	---	782.332	1908.896	---	16
P	8	---	879.384	1807.849	---	15
V	9	---	978.453	1710.796	---	14
R	10	---	1134.554	1611.728	---	13
Y	11	---	1297.617	1455.626	---	12
S@	12	---	1464.615	1292.563	---	11
S	13	---	1551.648	**1125.565**	1.722	10
S	14	---	1638.680	1038.533	---	9
E	15	---	1767.722	951.501	---	8
V	16	---	1866.791	822.458	---	7
N	17	---	1980.833	**723.390**	-0.759	6
H	18	---	2117.892	**609.347**	1.135	5
L	19	---	2230.976	**472.288**	-1.352	4
S	20	---	2318.008	**359.204**	1.078	3
P	21	---	2415.061	**272.172**	-0.090	2
R	22	---	---	175.119	---	1

+2						
Seq	#	b: Δ Error	b	y	y: Δ Error	+1
A	1	---	36.526	---	---	22
V	2	---	86.060	1259.572	---	21
D	3	---	143.574	1210.037	---	20
H	4	---	212.103	1152.524	---	19
E	5	---	276.624	1083.994	---	18
E	6	---	341.146	**1019.473**	0.026	17
T	7	---	391.669	954.952	---	16
P	8	---	440.196	904.428	---	15
V	9	---	489.730	855.902	---	14
R	10	-0.799	**567.781**	806.367	---	13
Y	11	---	649.312	728.317	---	12
S@	12	---	732.811	646.785	---	11
S	13	---	776.327	563.286	---	10
S	14	---	819.843	519.770	---	9
E	15	---	884.365	476.254	---	8
V	16	---	933.899	411.733	---	7
N	17	---	990.920	362.198	---	6
H	18	---	1059.450	305.177	---	5
L	19	---	1115.992	236.648	---	4
S	20	---	1159.508	180.106	---	3
P	21	---	1208.034	136.589	---	2
R	22	---	---	88.063	---	1

AVDHEETPVRY@SSSEVNHLSPR
z = 4+

DCBLD2 pY597

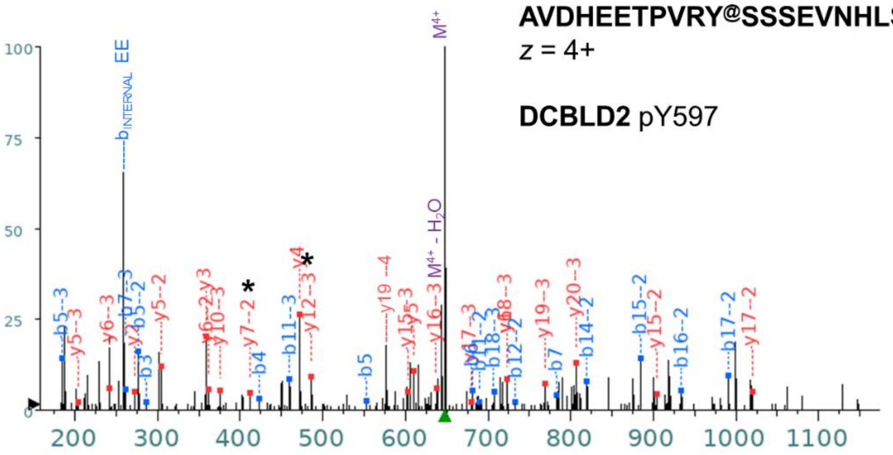

			+1			
Seq	#	b: Δ Error	b	y	y: Δ Error	+1
A	1	---	72.044	---	---	22
V	2	---	171.113	2518.136	---	21
D	3	-4.716	**286.140**	2419.067	---	20
H	4	3.343	**423.199**	2304.040	---	19
E	5	4.016	**552.241**	2166.982	---	18
E	6	-0.763	**681.284**	2037.939	---	17
T	7	1.273	**782.332**	1908.896	---	16
P	8	---	879.384	1807.849	---	15
V	9	---	978.453	1710.796	---	14
R	10	---	1134.554	1611.728	---	13
Y@	11	---	1377.583	1455.626	---	12
S	12	---	1464.615	1212.597	---	11
S	13	---	1551.648	1125.565	---	10
S	14	---	1638.680	1038.533	---	9
E	15	---	1767.722	951.501	---	8
V	16	---	1866.791	822.458	---	7
N	17	---	1980.833	723.390	---	6
H	18	---	2117.892	**609.347**	1.335	5
L	19	---	2230.976	**472.288**	-2.967	4
S	20	---	2318.008	**359.204**	-1.046	3
P	21	---	2415.061	**272.172**	-4.687	2
R	22	---	---	175.119	---	1

			+2			
Seq	#	b: Δ Error	b	y	y: Δ Error	+1
A	1	---	36.526	---	---	22
V	2	---	86.060	1259.572	---	21
D	3	---	143.574	1210.037	---	20
H	4	---	212.103	1152.524	---	19
E	5 1	---	276.624	1083.994	---	18
E	6	---	341.146	**1019.473**	13.796	17
T	7	---	391.669	954.952	---	16
P	8	---	440.196	**904.428**	-4.120	15
V	9	---	489.730	855.902	---	14
R	10	---	567.781	806.367	---	13
Y@	11	---	689.295	728.317	---	12
S	12	6.515	**732.811**	606.802	---	11
S	13	---	776.327	563.286	---	10
S	14	-5.623	**819.843**	519.770	---	9
E	15	-2.654	**884.365**	476.254	---	8
V	16	-8.555	**933.899**	**411.733**	-3.194	7
N	17	-4.408	**990.920**	**362.198**	-6.674	6
H	18	---	1059.450	**305.177**	-1.588	5
L	19	---	1115.992	236.648	---	4
S	20	---	1159.508	180.106	---	3
P	21	---	1208.034	136.589	---	2
R	22	---	---	88.063	---	1

			+3			
Seq	#	b: Δ Error	b	y	y: Δ Error	+1
A	1	---	24.686	---	---	22
V	2	---	57.709	840.050	---	21
D	3	---	96.051	**807.027**	-1.786	20
H	4	---	141.738	768.685	---	.9
E	5	---	184.752	**722.999**	1.627	18
E	6	---	227.766	679.985	---	.7
T	7 -	---	261.449	636.970	---	.6
P	8	---	293.800	**603.288**	-3.383	15
V	9	---	326.822	570.937	---	14
R	10	---	378.856	537.914	---	13
Y@	11	---	459.866	485.880	---	.2
S	12	---	488.877	404.870	---	11
S	13	---	517.887	375.860	---	.0
S	14	---	546.898	346.849	---	9
E	15	---	589.912	317.838	---	8
V	16	---	622.935	274.824	---	7
N	17	---	660.949	241.801	---	6
H	18	---	706.636	203.787	---	5
L	19	---	744.330	158.101	---	4
S	20	---	773.341	120.406	---	3
P	21	---	805.692	91.395	---	2
R	22	---	---	59.045	---	1

YSSSEVNHLSPR
z = 3+

DCBLD2 S599

+1

Seq	#	b: Δ Error	b	y	y: Δ Error	+1
Y	1	---	164.071	---	---	12
S	2	-2.379	251.103	1212.597	---	11
S	3	---	338.135	1125.565	---	10
S	4	---	425.167	1038.533	-1.850	9
E	5	---	554.209	951.501	0.723	8
V	6	---	653.278	822.458	-0.585	7
N	7	---	767.321	723.390	0.338	6
H	8	---	904.380	609.347	0.434	5
L	9	---	1017.464	472.288	-2.709	4
S	10	---	1104.496	359.204	-1.895	3
P	11	---	1201.548	272.172	-1.099	2
R	12	---	---	175.119	-0.494	1

+2

Seq	#	b: Δ Error	b	y	y: Δ Error	+1
Y	1	---	82.539	---	---	12
S	2	---	126.055	606.802	-0.915	11
S	3	---	169.571	563.286	-2.137	10
S	4	---	213.087	519.770	-0.628	9
E	5	---	277.608	476.254	-1.726	8
V	6	---	327.142	411.733	-2.453	7
N	7	---	384.164	362.198	-0.101	6
H	8	---	452.693	305.177	-1.788	5
L	9	---	509.235	236.648	---	4
S	10	---	552.751	180.106	---	3
P	11	---	601.278	136.589	---	2
R	12	---	---	88.063	---	1

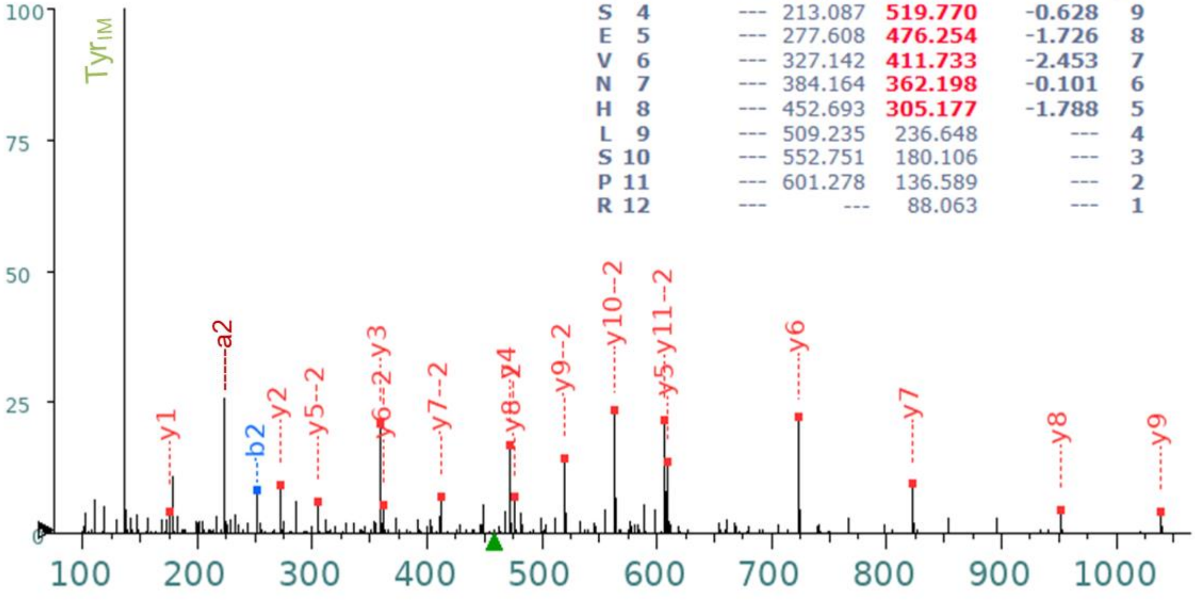

YSS@SEVNHLSPR
z = 3+

DCBLD2 pS599

		+1				
Seq	#	b: Δ Error	b	y	y: Δ Error	+1
Y	1	0.452	**164.071**	---	---	12
S	2	-0.860	**251.103**	1292.563	---	11
S@	3	---	418.101	1205.531	---	10
S	4	---	505.133	1038.533	---	9
E	5	---	634.176	**951.501**	-2.292	8
V	6	---	733.244	**822.458**	-1.179	7
N	7	---	847.287	**723.390**	1.097	6
H	8	---	984.346	**609.347**	0.434	5
L	9	---	1097.430	**472.288**	-0.899	4
S	10	---	1184.462	**359.204**	-1.895	3
P	11	---	1281.515	**272.172**	-1.323	2
R	12	---	---	**175.119**	-0.059	1

		+2				
Seq	#	b: Δ Error	b	y	y: Δ Error	+1
Y	1	---	82.539	---	---	12
S	2	1.877	**126.055**	646.785	---	11
S@	3	---	209.554	603.269	---	10
S	4	---	253.070	519.770	---	9
E	5	---	317.591	**476.254**	1.221	8
V	6	---	367.126	**411.733**	-6.604	7
N	7	---	424.147	**362.198**	-2.798	6
H	8	---	492.677	**305.177**	1.812	5
L	9	---	549.219	236.648	---	4
S	10	---	592.735	180.106	---	3
P	11	---	641.261	136.589	---	2
R	12	---	---	88.063	---	1

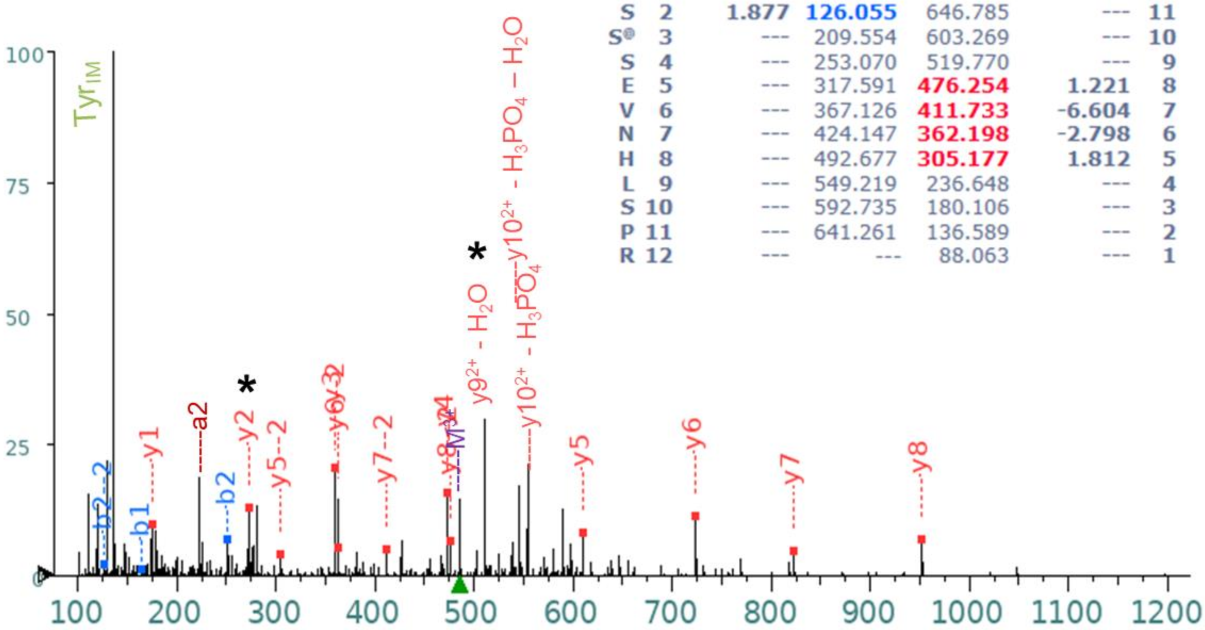

YSSSEVNHLSPR
z = 2+

DCBLD2 S600

Seq	#	b: Δ Error	b	y	y: Δ Error	+1
Y	1	---	164.071	---	---	12
S	2	-1.164	251.103	1212.597	---	11
S	3	---	338.135	1125.565	-0.989	10
S	4	---	425.167	1038.533	-1.615	9
E	5	---	554.209	951.501	-1.073	8
V	6	-1.194	653.278	822.458	-1.031	7
N	7	---	767.321	723.390	0.169	6
H	8	1.396	904.380	609.347	-2.471	5
L	9	2.182	1017.464	472.288	-0.964	4
S	10	1.526	1104.496	359.204	-1.640	3
P	11	---	1201.548	272.172	-1.548	2
R	12	---	---	175.119	0.638	1

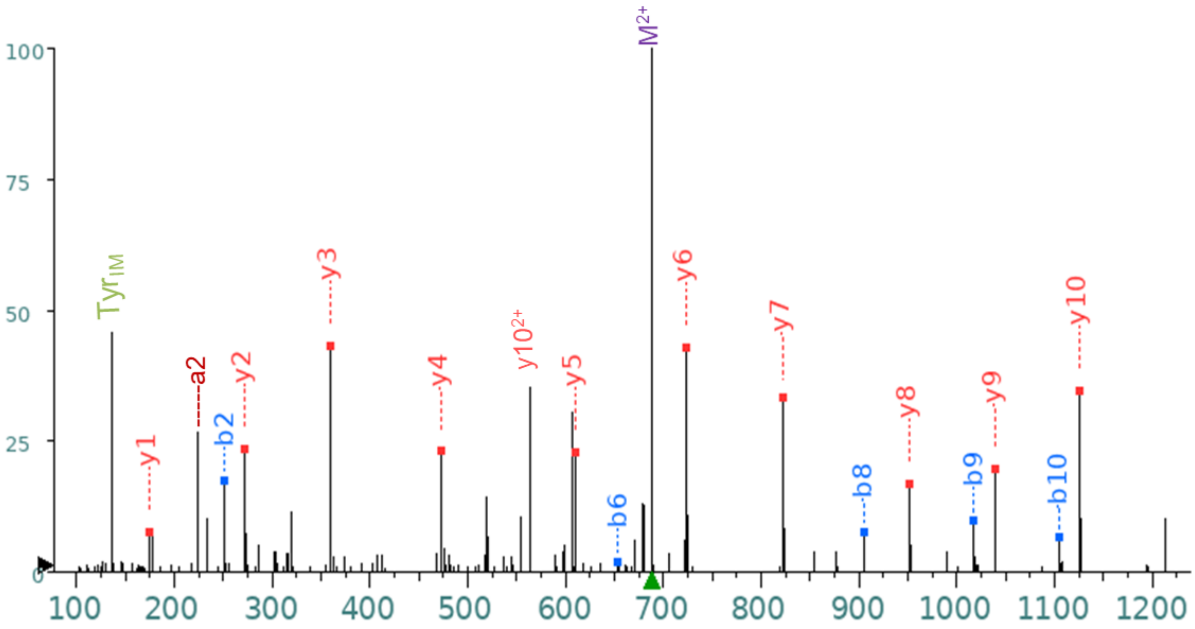

YSSS@EVNHLSPR
z = 2+

DCBLD2 pS600

Seq	#	b: Δ Error	b	y	y: Δ Error	+1
Y	1	---	164.071	---	---	12
S	2	0.051	251.103	1292.563	---	11
S	3	---	338.135	1205.531	---	10
S®	4	---	505.133	1118.499	---	9
E	5	---	634.176	951.501	-0.496	8
V	6	---	733.244	822.458	-0.660	7
N	7	---	847.287	723.390	0.422	6
H	8	-2.336	984.346	609.347	-0.568	5
L	9	-2.335	1097.430	472.288	-0.318	4
S	10	---	1184.462	359.204	-0.536	3
P	11	---	1281.515	272.172	-1.211	2
R	12	---	---	175.119	1.597	1

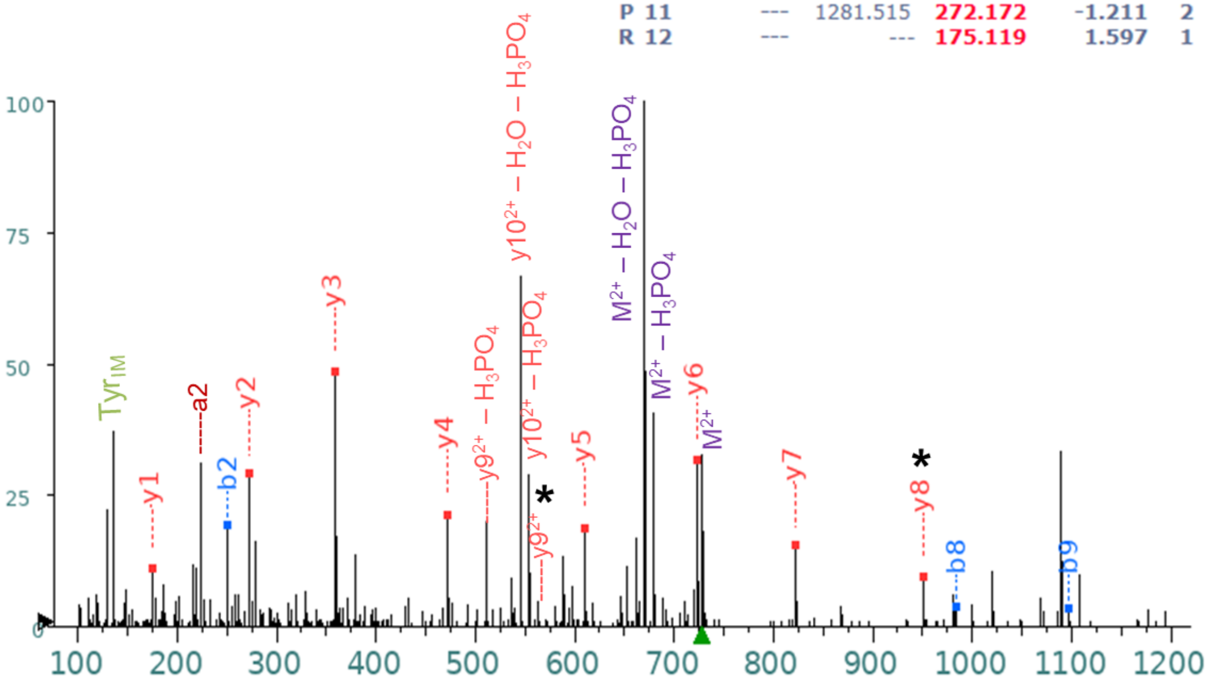

TDSC^SSQAQYDTPK
z = 2+

DCBLD2 S724, S727

Seq	#	b: Δ Error	b	y	y: Δ Error	+1
T	1	---	102.055	---	---	15
D	2	-0.719	217.082	1571.664	---	14
S	3	-0.715	304.114	1456.637	---	13
C^	4	---	478.160	1369.605	0.957	12
S	5	3.578	565.192	1195.559	0.512	11
S	6	---	652.224	1108.527	1.145	10
A	7	---	723.261	1021.495	-0.147	9
Q	8	---	851.320	950.458	-1.052	8
A	9	-0.232	922.357	822.399	-1.310	7
Q	10	---	1050.416	751.362	-0.210	6
Y	11	---	1213.479	623.304	-0.377	5
D	12	---	1328.506	460.240	---	4
T	13	---	1429.554	345.213	-1.209	3
P	14	---	1526.606	244.166	0.019	2
K	15	---	---	147.113	-0.182	1

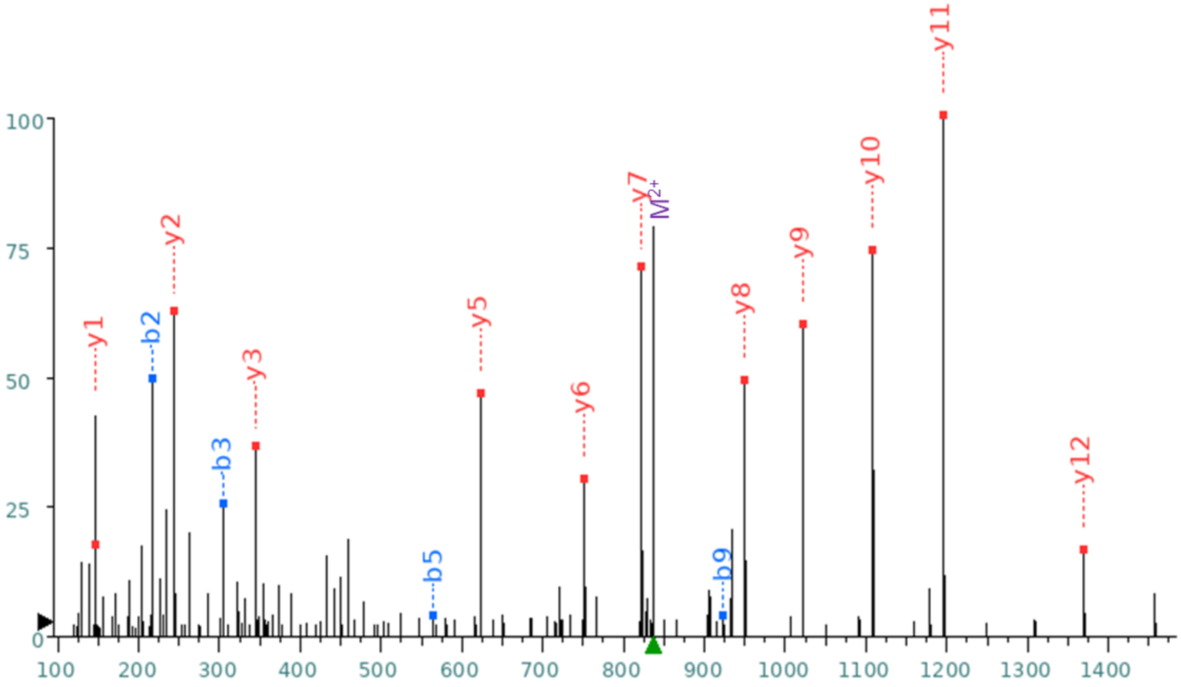

TDS@C^SSQAQYDTPK
z = 2+

DCBLD2 pS724

Seq	#	b: Δ Error	b	y	y: Δ Error	+1
T	1	---	102.055	---	---	15
D	2	-2.406	**217.082**	1651.631	---	14
S@	3	1.443	**384.080**	1536.604	---	13
C^	4	---	558.127	**1369.605**	-0.736	12
S	5	---	645.159	**1195.559**	1.533	11
S	6	---	732.191	**1108.527**	1.145	10
A	7	---	803.228	**1021.495**	-1.461	9
Q	8	---	931.286	**950.458**	1.067	8
A	9	---	1002.323	**822.399**	-1.087	7
Q	10	---	1130.382	**751.362**	0.115	6
Y	11	---	1293.445	**623.304**	-1.258	5
D	12	---	1408.472	460.240	---	4
T	13	---	1509.520	**345.213**	-0.590	3
P	14	---	1606.573	**244.166**	-0.106	2
K	15	---	---	**147.113**	-1.531	1

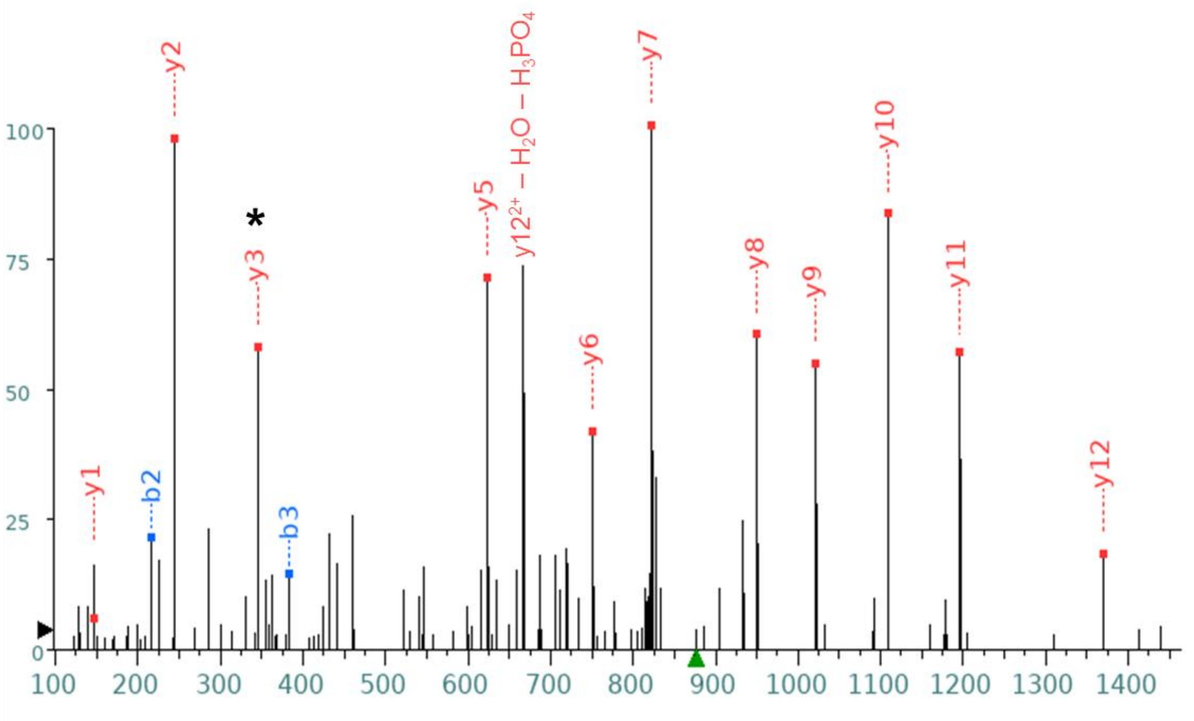

TDSC^SS@QAQYDTPK
z = 2+

DCBLD2 pS727

Seq	#	b: Δ Error	b	y	y: Δ Error	+1
T	1	---	102.055	---	---	15
D	2	0.194	217.082	1651.631	---	14
S	3	-2.822	304.114	1536.604	---	13
C^	4	---	478.160	1449.572	---	12
S	5	---	565.192	1275.525	---	11
S@	6	---	732.191	1188.493	0.844	10
A	7	---	803.228	1021.495	-0.266	9
Q	8	0.801	931.286	950.458	-0.025	8
A	9	-0.478	1002.323	822.399	-0.791	7
Q	10	---	1130.382	751.362	-0.534	6
Y	11	---	1293.445	623.304	0.896	5
D	12	---	1408.472	460.240	0.296	4
T	13	---	1509.520	345.213	-0.501	3
P	14	---	1606.573	244.166	-0.668	2
K	15	---	---	147.113	2.515	1

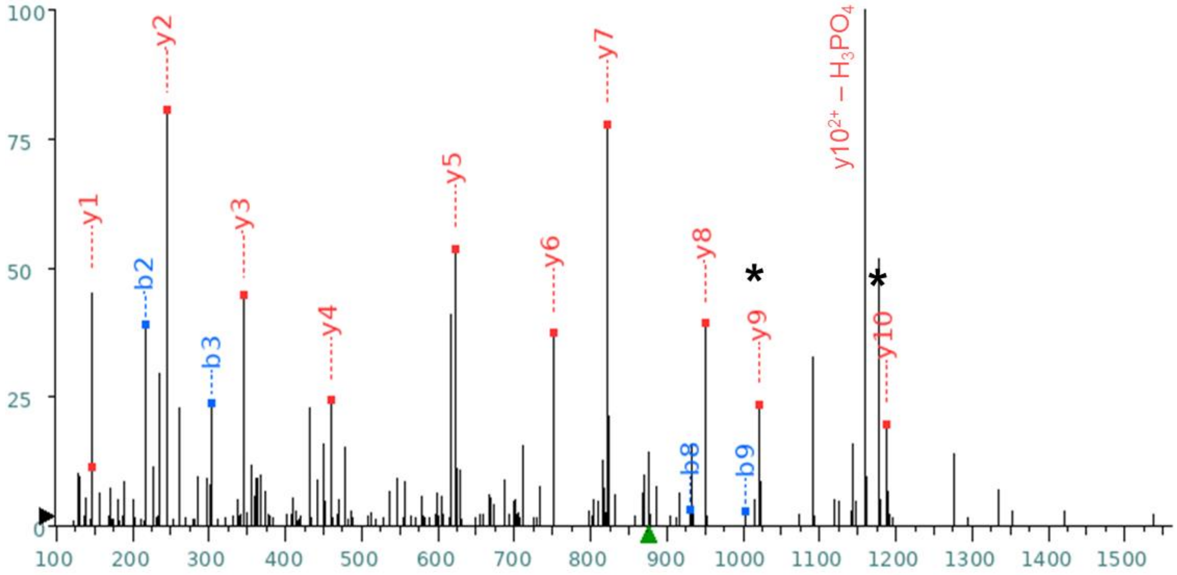

EVTTVLQADSAEYAQPLVGGIVGTLHQR

z = 3+

DCBLD2 S618, Y621

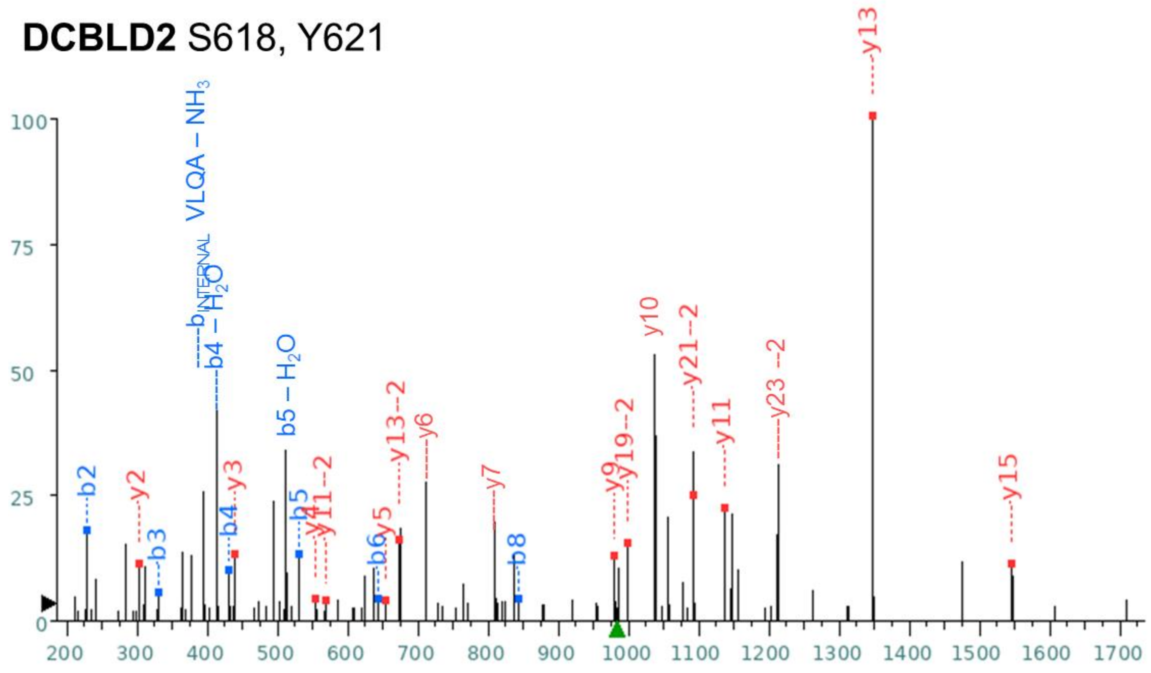

+1

Seq	#	b: Δ Error	b	y	y: Δ Error	+1
E	1	---	130.050	---	---	28
V	2	-2.251	**229.118**	2823.500	---	27
T	3	-2.886	**330.166**	2724.432	---	26
T	4	4.844	**431.214**	2623.384	---	25
V	5	0.901	**530.282**	2522.336	---	24
L	6	1.663	**643.366**	2423.268	---	23
Q	7	---	771.425	2310.184	---	22
A	8	-1.976	**842.462**	2182.125	---	21
D	9	---	957.489	2111.088	---	20
S	10	---	1044.521	1996.061	---	19
A	11	---	1115.558	1909.029	---	18
E	12	---	1244.600	1837.992	---	17
Y	13	---	1407.664	1708.949	---	16
A	14	---	1478.701	**1545.886**	0.790	15
Q	15	---	1606.760	1474.849	---	14
P	16	---	1703.812	**1346.790**	0.807	13
L	17	---	1816.896	1249.738	---	12
V	18	---	1915.965	**1136.653**	-0.129	11
G	19	---	1972.986	1037.585	---	10
G	20	---	2030.008	**980.564**	0.818	9
I	21	---	2143.092	923.542	---	8
V	22	---	2242.160	810.458	---	7
G	23	---	2299.182	711.390	---	6
T	24	---	2400.229	**654.368**	1.275	5
L	25	---	2513.313	**553.321**	2.188	4
H	26	---	2650.372	**440.236**	1.752	3
Q	27	---	2778.431	**303.178**	-0.534	2
R	28	---	---	175.119	---	1

+2

Seq	#	b: Δ Error	b	y	y: Δ Error	+1
E	1	---	65.529	---	---	28
V	2	---	115.063	1412.254	---	27
T	3	---	165.587	1362.719	---	26
T	4	---	216.110	1312.196	---	25
V	5	---	265.645	1261.672	---	24
L	6	---	322.187	1212.138	---	23
Q	7	---	386.216	1155.595	---	22
A	8	---	421.735	**1091.566**	-0.474	21
D	9	---	479.248	1056.048	---	20
S	10	---	522.764	**998.534**	0.078	19
A	11	---	558.283	955.018	---	18
E	12	---	622.804	919.500	---	17
Y	13	---	704.336	854.978	---	16
A	14	---	739.854	773.447	---	15
Q	15	---	803.883	737.928	---	14
P	16	---	852.410	**673.899**	-0.517	13
L	17	---	908.952	625.372	---	12
V	18	---	958.486	**568.830**	-2.769	11
G	19	---	986.997	519.296	---	10
G	20	---	1015.507	490.785	---	9
I	21	---	1072.050	462.275	---	8
V	22	---	1121.584	405.733	---	7
G	23	---	1150.094	356.198	---	6
T	24	---	1200.618	327.688	---	5
L	25	---	1257.160	277.164	---	4
H	26	---	1325.690	220.622	---	3
Q	27	---	1389.719	152.092	---	2
R	28	---	---	88.063	---	1

EVTTVLQADS@AEYAQPLVGGIVGTLHQR
z = 3+

DCBLD2 pS618

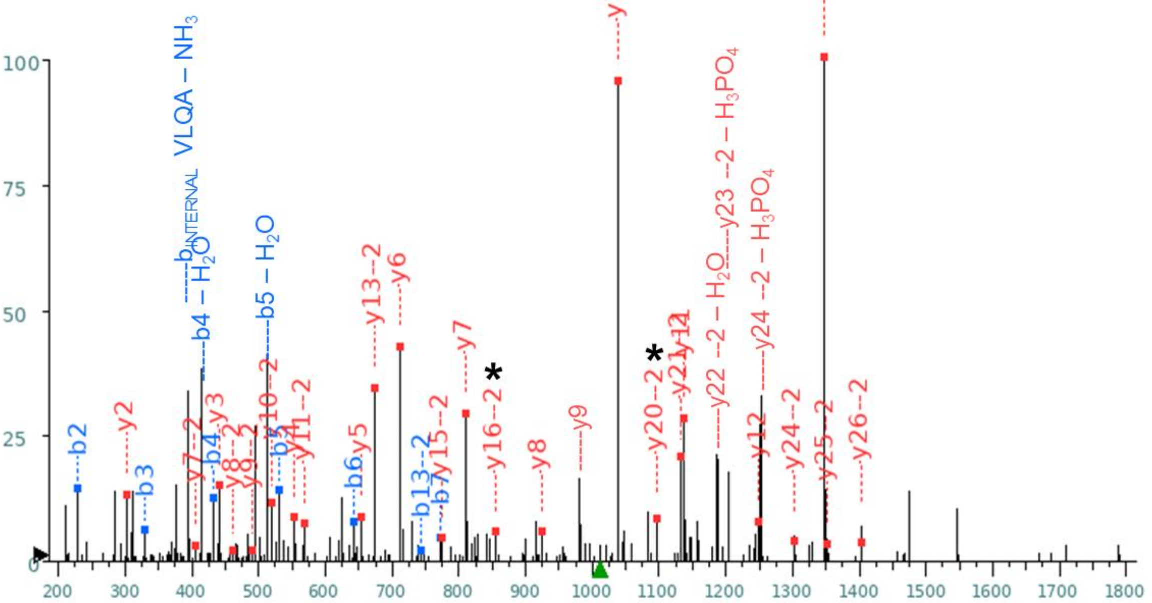

+1

Seq	#	b: Δ Error	b	y	y: Δ Error	+1
E	1	---	130.050	---	---	28
V	2	0.413	**229.118**	2903.466	---	27
T	3	-2.332	**330.166**	2804.398	---	26
T	4	-1.879	**431.214**	2703.350	---	25
V	5	0.671	**530.282**	2602.302	---	24
L	6	2.137	**643.366**	2503.234	---	23
Q	7	2.832	**771.425**	2390.150	---	22
A	8	---	842.462	2262.091	---	21
D	9	---	957.489	2191.054	---	20
S@	10	---	1124.487	2076.027	---	19
A	11	---	1195.524	1909.029	---	18
E	12	---	1324.567	1837.992	---	17
Y	13	---	1487.630	1708.949	---	16
A	14	---	1558.667	1545.886	---	15
Q	15	---	1686.726	1474.849	---	14
P	16	---	1783.779	**1346.790**	1.532	13
L	17	---	1896.863	**1249.738**	0.698	12
V	18	---	1995.931	**1136.653**	2.341	11
G	19	---	2052.953	**1037.585**	0.617	10
G	20	---	2109.974	980.564	---	9
I	21	---	2223.058	**923.542**	-2.194	8
V	22	---	2322.126	**810.458**	0.008	7
G	23	---	2379.148	**711.390**	-0.257	6
T	24	---	2480.196	**654.368**	-4.228	5
L	25	---	2593.280	**553.321**	-2.114	4
H	26	---	2730.339	**440.236**	1.683	3
Q	27	---	2858.397	**303.178**	-1.339	2
R	28	---	---	175.119	---	1

+2

Seq	#	b: Δ Error	b	y	y: Δ Error	+1
E	1	---	65.529	---	---	28
V	2	---	115.063	1452.237	---	27
T	3	---	165.587	**1402.703**	0.227	26
T	4	---	216.110	**1352.179**	-0.822	25
V	5	---	265.645	**1301.655**	-2.139	24
L	6	---	322.187	1252.121	---	23
Q	7	---	386.216	1195.579	---	22
A	8	---	421.735	**1131.549**	1.367	21
D	9	---	479.248	**1096.031**	1.079	20
S@	10	---	562.747	1038.517	---	19
A	11	---	598.266	955.018	---	18
E	12	---	662.787	919.500	---	17
Y	13	0.265	**744.319**	**854.978**	2.042	16
A	14	---	779.837	**773.447**	0.820	15
Q	15	---	843.867	737.928	---	14
P	16	---	892.393	**673.899**	0.299	13
L	17	---	948.935	625.372	---	12
V	18	---	998.469	**568.830**	0.450	11
G	19	---	1026.980	**519.296**	-0.983	10
G	20	---	1055.491	**490.785**	-0.874	9
I	21	---	1112.033	**462.275**	1.954	8
V	22	---	1161.567	**405.733**	1.723	7
G	23	---	1190.078	356.198	---	6
T	24	---	1240.601	327.688	---	5
L	25	---	1297.143	277.164	---	4
H	26	---	1365.673	220.622	---	3
Q	27	---	1429.702	152.092	---	2
R	28	---	---	88.063	---	1

EVTTVLQADSAEY@AQPLVGGIVGTLHQR

z = 3+

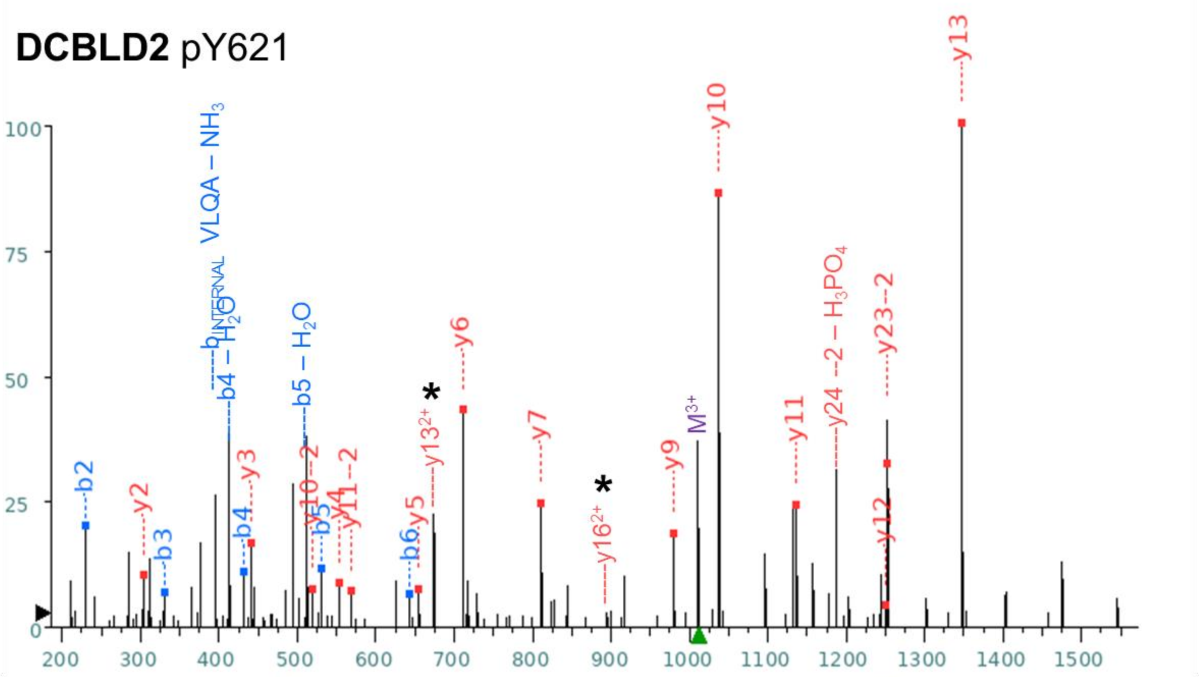

+1

Seq	#	b: Δ Error	b	y	y: Δ Error	+1
E	1	---	130.050	---	---	28
V	2	-1.252	**229.118**	2903.466	---	27
T	3	-2.054	**330.166**	2804.398	---	26
T	4	2.650	**431.214**	2703.350	---	25
V	5	-0.135	**530.282**	2602.302	---	24
L	6	4.603	**643.366**	2503.234	---	23
Q	7	---	771.425	2390.150	---	22
A	8	---	842.462	2262.091	---	21
D	9	---	957.489	2191.054	---	20
S	10	---	1044.521	2076.027	---	19
A	11	---	1115.558	1988.995	---	18
E	12	---	1244.600	1917.958	---	17
Y@	13	---	1487.630	1788.916	---	16
A	14	---	1558.667	1545.886	---	15
Q	15	---	1686.726	1474.849	---	14
P	16	---	1783.779	**1346.790**	0.535	13
L	17	---	1896.863	**1249.738**	0.698	12
V	18	---	1995.931	**1136.653**	0.515	11
G	19	---	2052.953	**1037.585**	-0.089	10
G	20	---	2109.974	**980.564**	1.690	9
I	21	---	2223.058	923.542	---	8
V	22	---	2322.126	**810.458**	-1.573	7
G	23	---	2379.148	**711.390**	-0.428	6
T	24	---	2480.196	**654.368**	2.674	5
L	25	---	2593.280	**553.321**	1.967	4
H	26	---	2730.339	**440.236**	-1.437	3
Q	27	---	2858.397	**303.178**	-1.742	2
R	28	---	---	175.119	---	1

+2

Seq	#	b: Δ Error	b	y	y: Δ Error	+1
E	1	---	65.529	---	---	28
V	2	---	115.063	1452.237	---	27
T	3	---	165.587	1402.703	---	26
T	4	---	216.110	1352.179	---	25
V	5	---	265.645	1301.655	---	24
L	6	---	322.187	**1252.121**	-0.545	23
Q	7	---	386.216	1195.579	---	22
A	8	---	421.735	1131.549	---	21
D	9	---	479.248	1096.031	---	20
S	10	---	522.764	1038.517	---	19
A	11	---	558.283	995.001	---	18
E	12	---	622.804	959.483	---	17
Y@	13	---	744.319	894.961	---	16
A	14	---	779.837	773.447	---	15
Q	15	---	843.867	737.928	---	14
P	16	---	892.393	673.899	---	13
L	17	---	948.935	625.372	---	12
V	18	---	998.469	**568.830**	2.596	11
G	19	---	1026.980	**519.296**	2.073	10
G	20	---	1055.491	490.785	---	9
I	21	---	1112.033	462.275	---	8
V	22	---	1161.567	405.733	---	7
G	23	---	1190.078	356.198	---	6
T	24	---	1240.601	327.688	---	5
L	25	---	1297.143	277.164	---	4
H	26	---	1365.673	220.622	---	3
Q	27	---	1429.702	152.092	---	2
R	28	---	---	88.063	---	1

STFKPEEGKEAGYADLDPYNSPGQE
z = 3+

DCBLD2 Y649, Y655

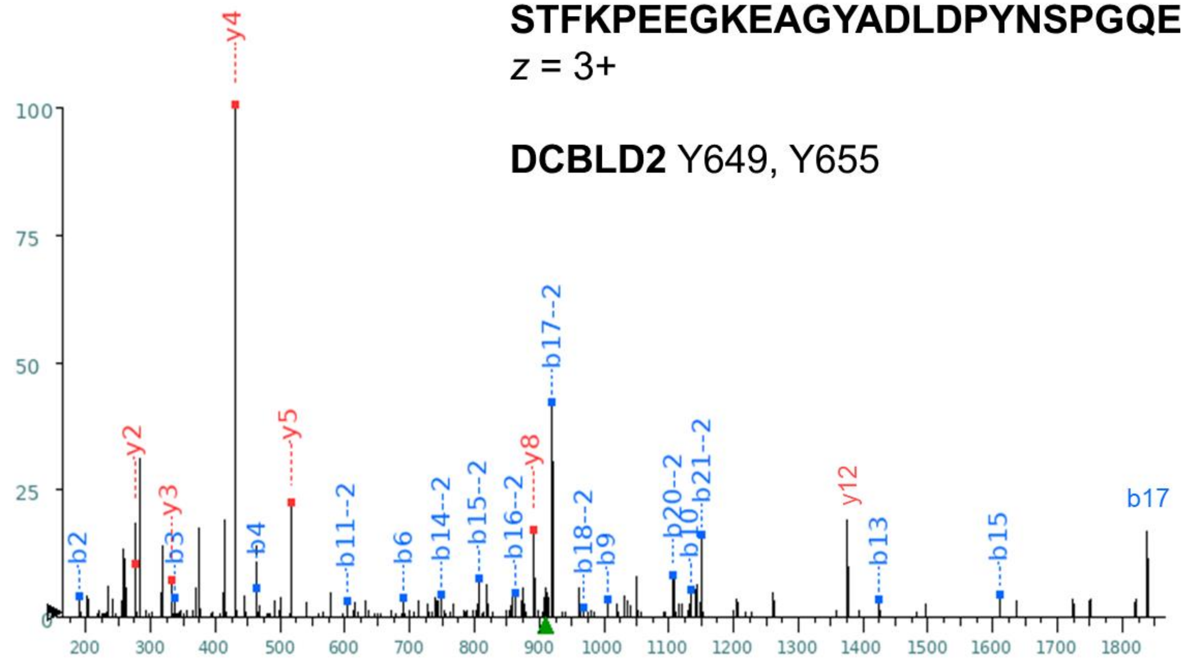

+1

Seq	#	b: Δ Error	b	y	y: Δ Error	+1
S	1	---	88.039	---	---	25
T	2	-1.817	**189.087**	2642.189	---	24
F	3	2.629	**336.155**	2541.142	---	23
K	4	0.737	**464.250**	2394.073	---	22
P	5	---	561.303	2265.978	---	21
E	6	-1.523	**690.346**	2168.925	---	20
E	7	---	819.388	2039.883	---	19
G	8	---	876.410	1910.840	---	18
K	9	-1.860	**1004.505**	1853.819	---	17
E	10	0.030	**1133.547**	1725.724	---	16
A	11	---	1204.584	1596.681	---	15
G	12	---	1261.606	1525.644	---	14
Y	13	-1.146	**1424.669**	1468.623	---	13
A	14	---	1495.706	1305.559	---	12
D	15	0.899	**1610.733**	1234.522	---	11
L	16	---	1723.817	1119.495	---	10
D	17	---	1838.844	1006.411	---	9
P	18	---	1935.897	**891.384**	-1.450	8
Y	19	---	2098.960	794.332	---	7
N	20	---	2213.003	631.268	---	6
S	21	---	2300.035	**517.225**	-0.802	5
P	22	---	2397.088	**430.193**	-0.928	4
G	23	---	2454.110	**333.140**	0.997	3
Q	24	---	2582.168	**276.119**	4.334	2
E	25	---	---	148.060	---	1

+2

Seq	#	b: Δ Error	b	y	y: Δ Error	+1
S	1	---	44.523	---	---	25
T	2	---	95.047	1321.598	---	24
F	3	---	168.581	1271.074	---	23
K	4	---	232.629	1197.540	---	22
P	5	---	281.155	1133.493	---	21
E	6	---	345.676	1084.966	---	20
E	7	---	410.198	1020.445	---	19
G	8	---	438.709	955.924	---	18
K	9	---	502.756	927.413	---	17
E	10	---	567.277	863.366	---	16
A	11	-0.442	**602.796**	798.844	---	15
G	12	---	631.307	763.326	---	14
Y	13	---	712.838	734.815	---	13
A	14	0.977	**748.357**	653.283	---	12
D	15	1.989	**805.870**	617.765	---	11
L	16	-0.098	**862.412**	560.251	---	10
D	17	-0.405	**919.926**	503.709	---	9
P	18	2.058	**968.452**	446.196	---	8
Y	19	---	1049.984	397.669	---	7
N	20	-0.706	**1107.005**	316.138	---	6
S	21	0.468	**1150.521**	259.116	---	5
P	22	---	1199.048	215.600	---	4
G	23	---	1227.558	167.074	---	3
Q	24	---	1291.588	138.563	---	2
E	25	---	---	74.534	---	1

STFKPEEGKEAGY@ADLDPY@NSPGQE
z = 3+

DCBLD2 pY649, pY655

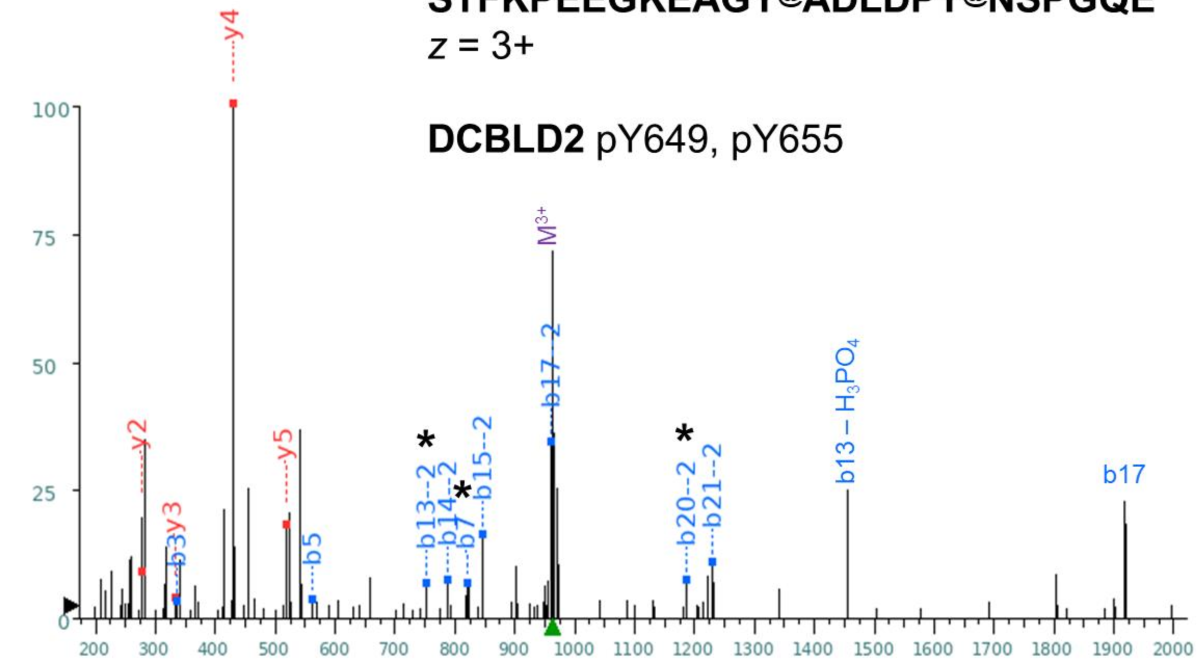

			+1			
Seq	#	b: Δ Error	b	y	y: Δ Error	+1
S	1	---	88.039	---	---	25
T	2	---	189.087	2802.122	---	24
F	3	3.355	**336.155**	2701.074	---	23
K	4	---	464.250	2554.006	---	22
P	5	-0.259	**561.303**	2425.911	---	21
E	6	---	690.346	2328.858	---	20
E	7	-0.303	**819.388**	2199.816	---	19
G	8	---	876.410	2070.773	---	18
K	9	---	1004.505	2013.751	---	17
E	10	---	1133.547	1885.657	---	16
A	11	---	1204.584	1756.614	---	15
G	12	---	1261.606	1685.577	---	14
Y@	13	---	1504.636	1628.555	---	13
A	14	---	1575.673	1385.526	---	12
D	15	---	1690.700	1314.489	---	11
L	16	---	1803.784	1199.462	---	10
D	17	---	1918.811	1086.378	---	9
P	18	---	2015.863	971.351	---	8
Y@	19	---	2258.893	874.298	---	7
N	20	---	2372.936	631.268	---	6
S	21	---	2459.968	**517.225**	1.441	5
P	22	---	2557.021	**430.193**	-1.779	4
G	23	---	2614.042	**333.140**	1.639	3
Q	24	---	2742.101	**276.119**	0.686	2
E	25	---	---	148.060	---	1

			+2			
Seq	#	b: Δ Error	b	y	y: Δ Error	+1
S	1	---	44.523	---	---	25
T	2	---	95.047	1401.565	---	24
F	3	---	168.581	1351.041	---	23
K	4	---	232.629	1277.507	---	22
P	5	---	281.155	1213.459	---	21
E	6	---	345.676	1164.933	---	20
E	7	---	410.198	1100.411	---	19
G	8	---	438.709	1035.890	---	18
K	9	---	502.756	1007.379	---	17
E	10	---	567.277	943.332	---	16
A	11	---	602.796	878.811	---	15
G	12	---	631.307	843.292	---	14
Y@	13	0.230	**752.821**	814.781	---	13
A	14	-1.177	**788.340**	693.266	---	12
D	15	-0.788	**845.853**	657.748	---	11
L	16	---	902.395	600.234	---	10
D	17	0.045	**959.909**	543.692	---	9
P	18	---	1008.435	486.179	---	8
Y@	19	---	1129.950	437.653	---	7
N	20	1.175	**1186.972**	316.138	---	6
S	21	-0.076	**1230.488**	259.116	---	5
P	22	---	1279.014	215.600	---	4
G	23	---	1307.525	167.074	---	3
Q	24	---	1371.554	138.563	---	2
E	25	---	---	74.534	---	1

AGYADLDPYNSPGQE
z = 2+

DCBLD2 Y649, Y655, S657

Seq	#	b: Δ Error	b	y	y: Δ Error	+1
A	1	---	72.044	---	---	15
G	2	0.618	129.066	1525.644	---	14
Y	3	-1.049	292.129	1468.623	---	13
A	4	-0.604	363.166	1305.559	---	12
D	5	-1.726	478.193	1234.522	---	11
L	6	1.411	591.277	1119.495	---	10
D	7	-0.757	706.304	1006.411	---	9
P	8	-3.286	803.357	891.384	-1.381	8
Y	9	---	966.420	794.332	---	7
N	10	0.670	1080.463	631.268	-1.268	6
S	11	-1.824	1167.495	517.225	-2.100	5
P	12	---	1264.548	430.193	-0.999	4
G	13	---	1321.570	333.140	-3.216	3
Q	14	---	1449.628	276.119	-0.419	2
E	15	---	---	148.060	-0.066	1

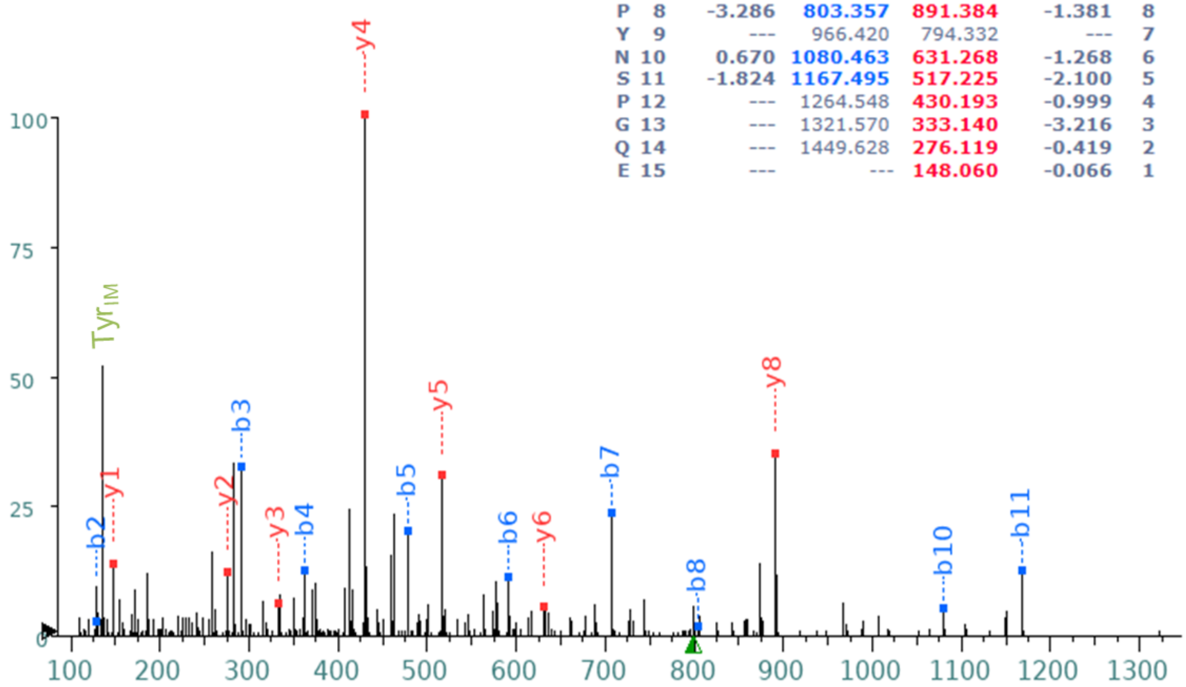

AGY@ADLDPYNSPGQE
z = 2+

DCBLD2 pY649

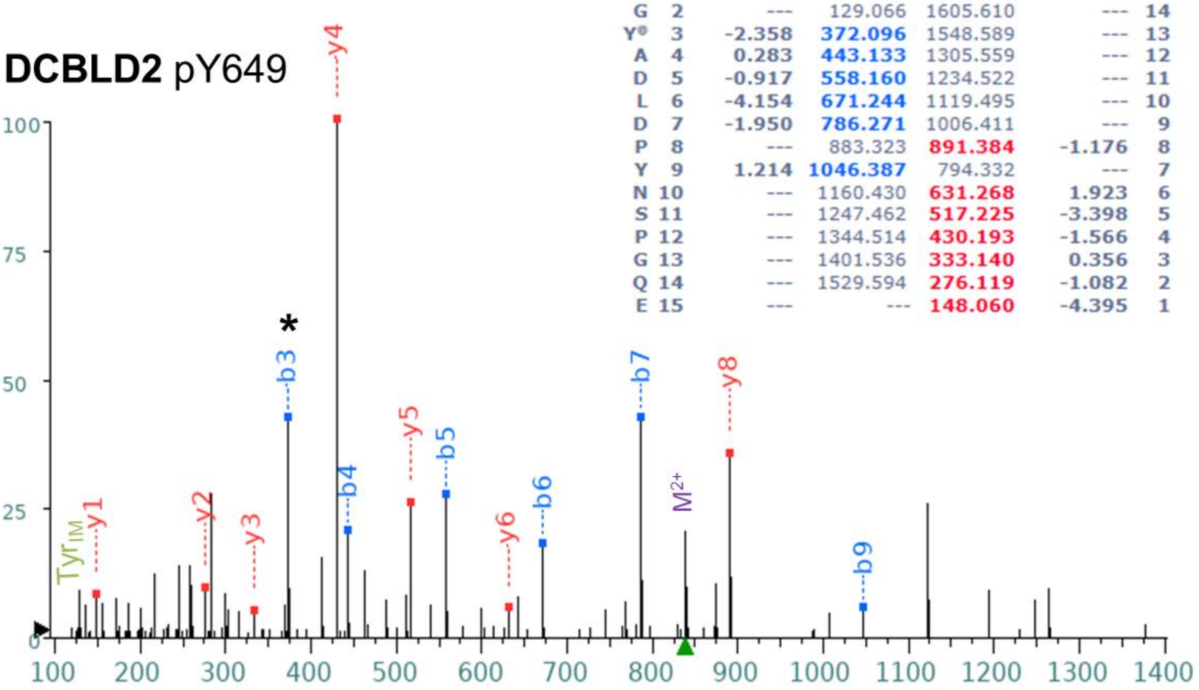

Seq	#	b: Δ Error	b	y	y: Δ Error	+1
A	1	---	72.044	---	---	15
G	2	---	129.066	1605.610	---	14
Y@	3	-2.358	**372.096**	1548.589	---	13
A	4	0.283	**443.133**	1305.559	---	12
D	5	-0.917	**558.160**	1234.522	---	11
L	6	-4.154	**671.244**	1119.495	---	10
D	7	-1.950	**786.271**	1006.411	---	9
P	8	---	883.323	**891.384**	-1.176	8
Y	9	1.214	**1046.387**	794.332	---	7
N	10	---	1160.430	**631.268**	1.923	6
S	11	---	1247.462	**517.225**	-3.398	5
P	12	---	1344.514	**430.193**	-1.566	4
G	13	---	1401.536	**333.140**	0.356	3
Q	14	---	1529.594	**276.119**	-1.082	2
E	15	---	---	**148.060**	-4.395	1

AGYADLDPY@NSPGQE
z = 2+

DCBLD2 pY655

Seq	#	b: Δ Error	b	y	y: Δ Error	+1
A	1	---	72.044	---	---	15
G	2	---	129.066	1605.610	---	14
Y	3	-0.735	292.129	1548.589	---	13
A	4	-3.881	363.166	1385.526	---	12
D	5	-3.577	478.193	1314.489	---	11
L	6	-1.892	591.277	1199.462	---	10
D	7	-1.103	706.304	1086.378	1.463	9
P	8	---	803.357	971.351	-1.918	8
Y®	9	---	1046.387	874.298	---	7
N	10	-0.026	1160.430	631.268	2.213	6
S	11	-0.942	1247.462	517.225	-1.982	5
P	12	---	1344.514	430.193	-1.070	4
G	13	---	1401.536	333.140	0.173	3
Q	14	---	1529.594	276.119	-1.192	2
E	15	---	---	148.060	0.655	1

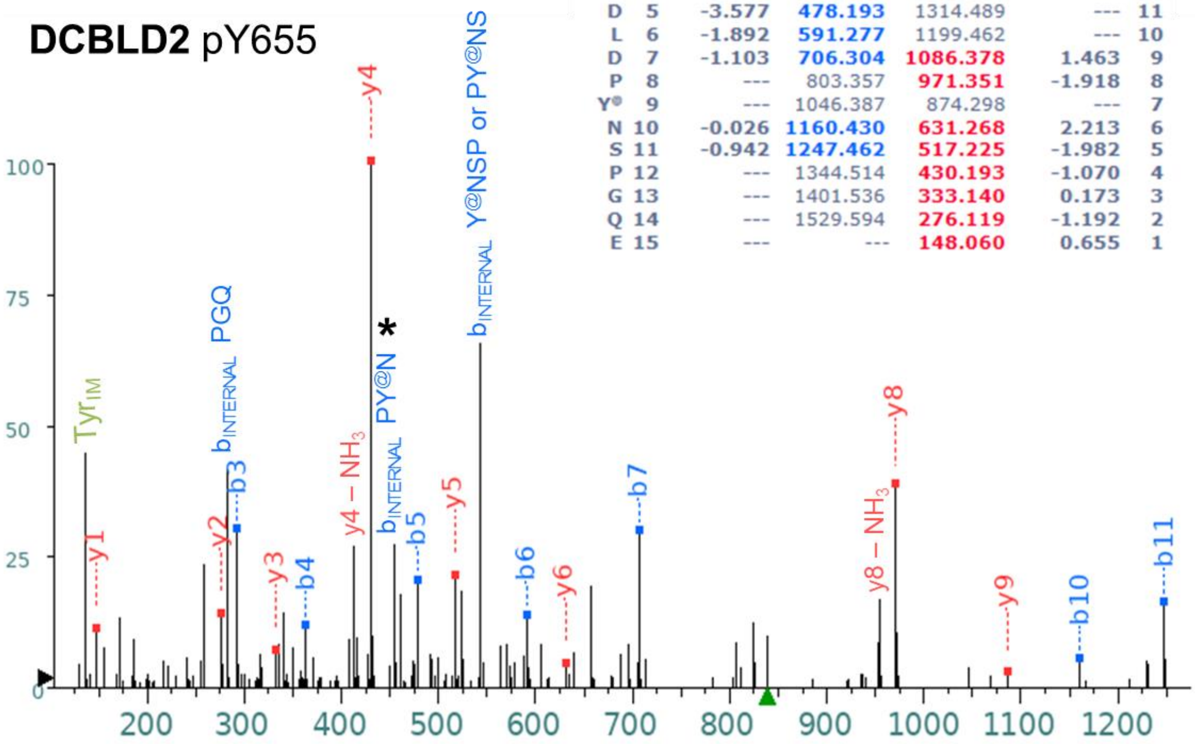

AGYADLDPYNS@PGQE
z = 2+

DCBLD2 pS657

Seq	#	b: Δ Error	b	y	y: Δ Error	+1
A	1	---	72.044	---	---	15
G	2	---	129.066	1605.610	---	14
Y	3	-1.466	292.129	1548.589	---	13
A	4	-1.108	363.166	1385.526	---	12
D	5	-4.088	478.193	1314.489	---	11
L	6	0.379	591.277	1199.462	---	10
D	7	-2.313	706.304	1086.378	---	9
P	8	---	803.357	971.351	0.029	8
Y	9	0.580	966.420	874.298	---	7
N	10	---	1080.463	711.235	0.131	6
S@	11	---	1247.462	597.192	3.460	5
P	12	---	1344.514	430.193	-2.276	4
G	13	---	1401.536	333.140	---	3
Q	14	---	1529.594	276.119	0.134	2
E	15	---	---	148.060	0.758	1

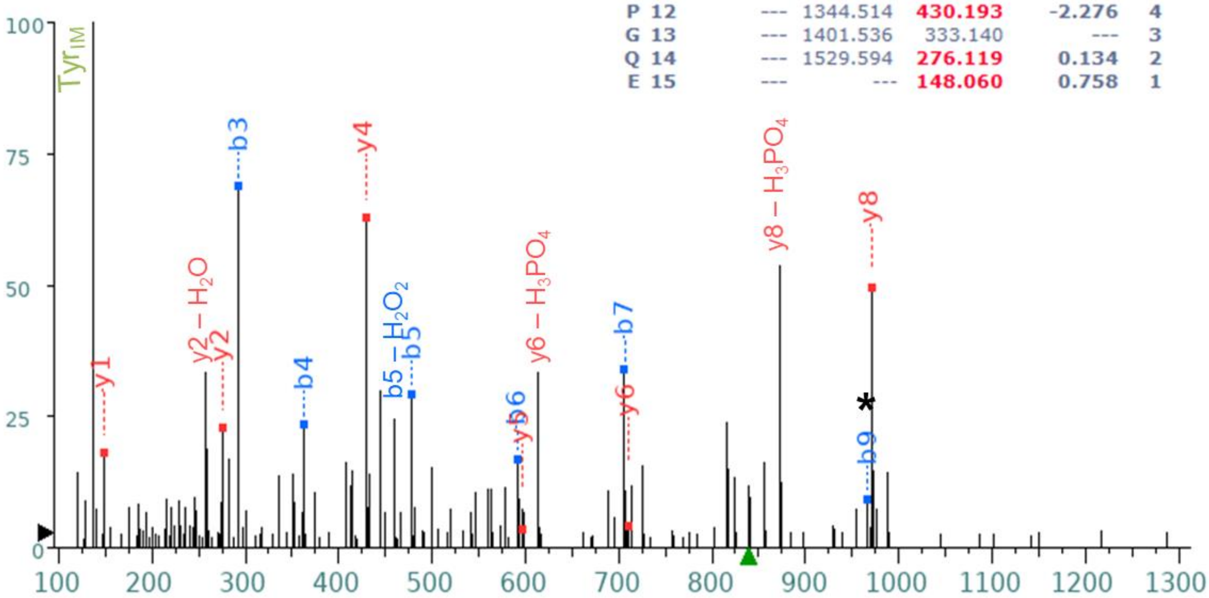

TEGTYDLPYWDR
z = 2+

DCBLD2 Y565, Y569

Seq	#	b: Δ Error	b	y	y: Δ Error	+1
T	1	---	102.055	---	---	12
E	2	-0.916	231.098	1414.627	---	11
G	3	-1.564	288.119	1285.585	0.237	10
T	4	-0.948	389.167	1228.563	-2.054	9
Y	5	-2.832	552.230	1127.516	-0.118	8
D	6	---	667.257	964.452	-1.178	7
L	7	-2.140	780.341	849.425	-1.019	6
P	8	---	877.394	736.341	-1.813	5
Y	9	---	1040.457	639.289	-1.374	4
W	10	---	1226.536	476.225	-1.771	3
D	11	---	1341.563	290.146	-2.283	2
R	12	---	---	175.119	-0.581	1

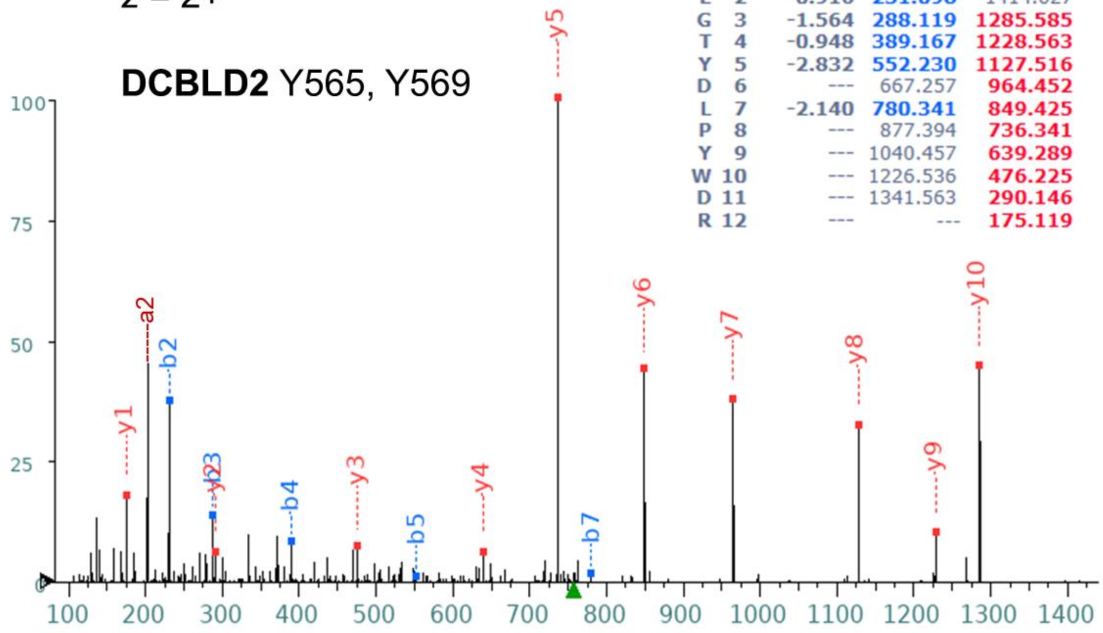

TEGTY@DLPYWDR
z = 2+

DCBLD2 pY565

Seq	#	b: Δ Error	b	y	y: Δ Error	+1
T	1	---	102.055	---	---	12
E	2	-0.190	231.098	1494.594	---	11
G	3	-2.093	288.119	1365.551	-1.492	10
T	4	---	389.167	1308.530	0.200	9
Y@	5	---	632.196	1207.482	-0.432	8
D	6	-0.769	747.223	964.452	0.088	7
L	7	---	860.307	849.425	1.424	6
P	8	---	957.360	736.341	-1.399	5
Y	9	---	1120.423	639.289	-1.278	4
W	10	---	1306.503	476.225	0.088	3
D	11	---	1421.530	290.146	0.241	2
R	12	---	---	175.119	-0.146	1

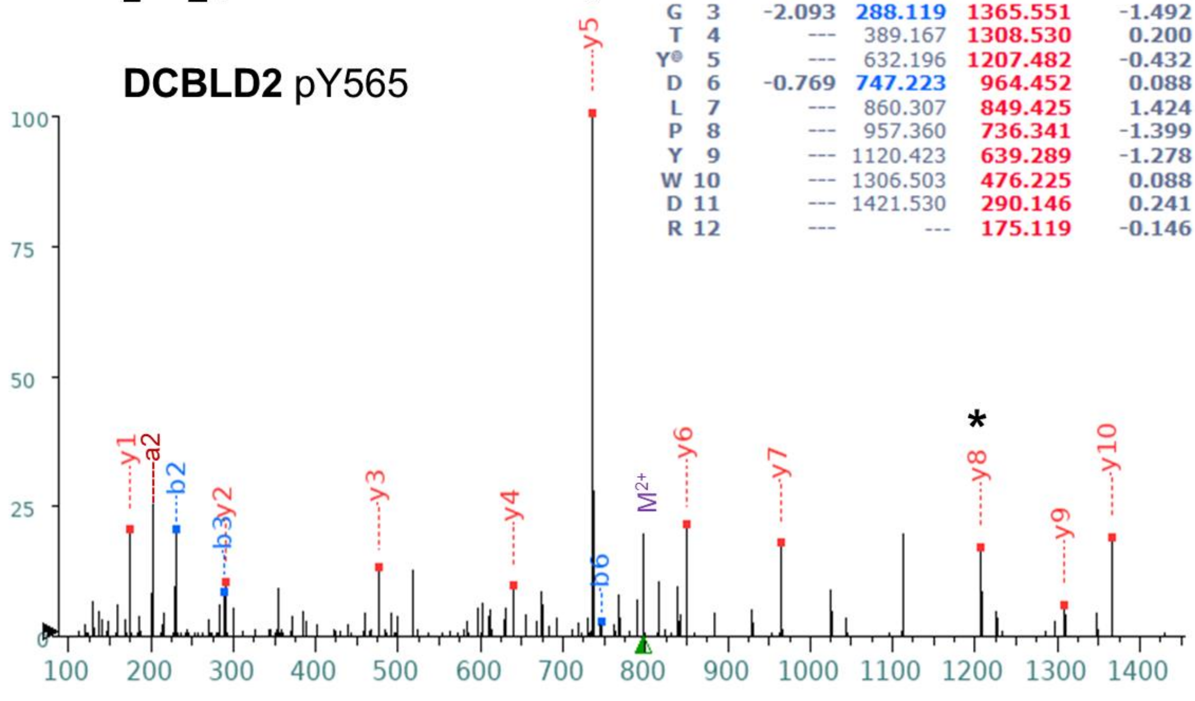

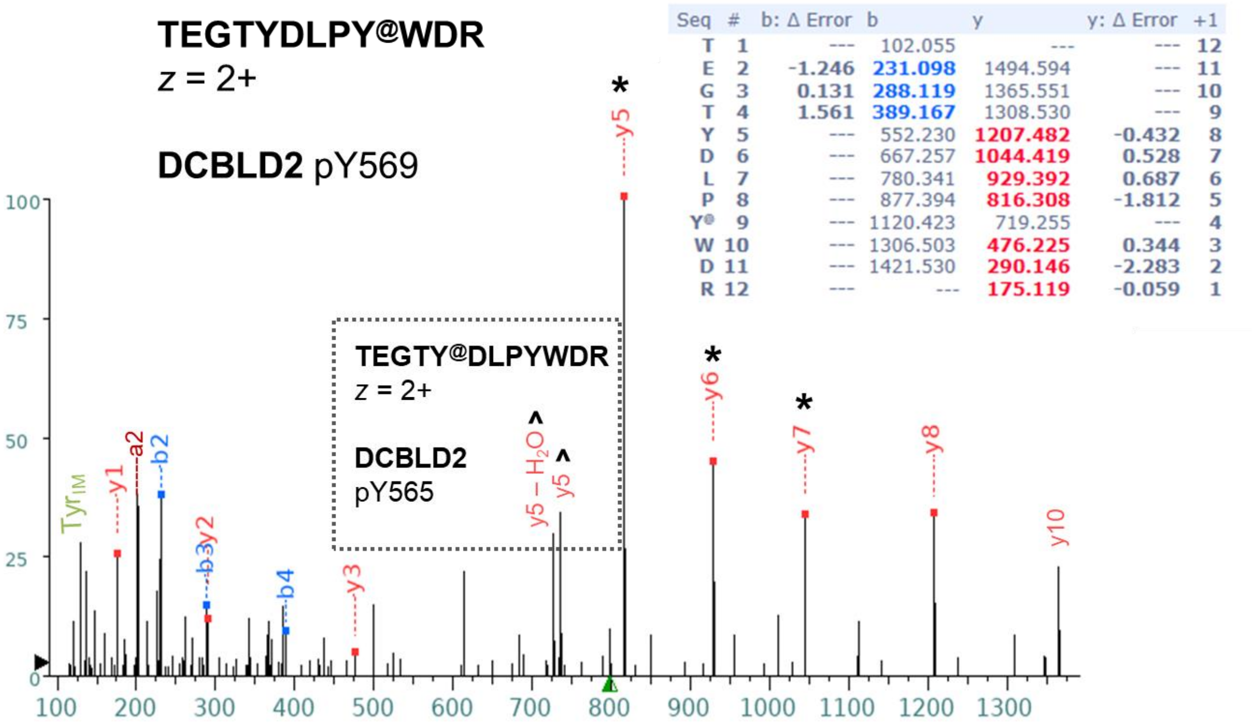

Seq	#	b: Δ Error	b	y	y: Δ Error	+1
T	1	---	102.055	---	---	12
E	2	-1.246	231.098	1494.594	---	11
G	3	0.131	288.119	1365.551	---	10
T	4	1.561	389.167	1308.530	---	9
Y	5	---	552.230	1207.482	-0.432	8
D	6	---	667.257	1044.419	0.528	7
L	7	---	780.341	929.392	0.687	6
P	8	---	877.394	816.308	-1.812	5
Y@	9	---	1120.423	719.255	---	4
W	10	---	1306.503	476.225	0.344	3
D	11	---	1421.530	290.146	-2.283	2
R	12	---	---	175.119	-0.059	1

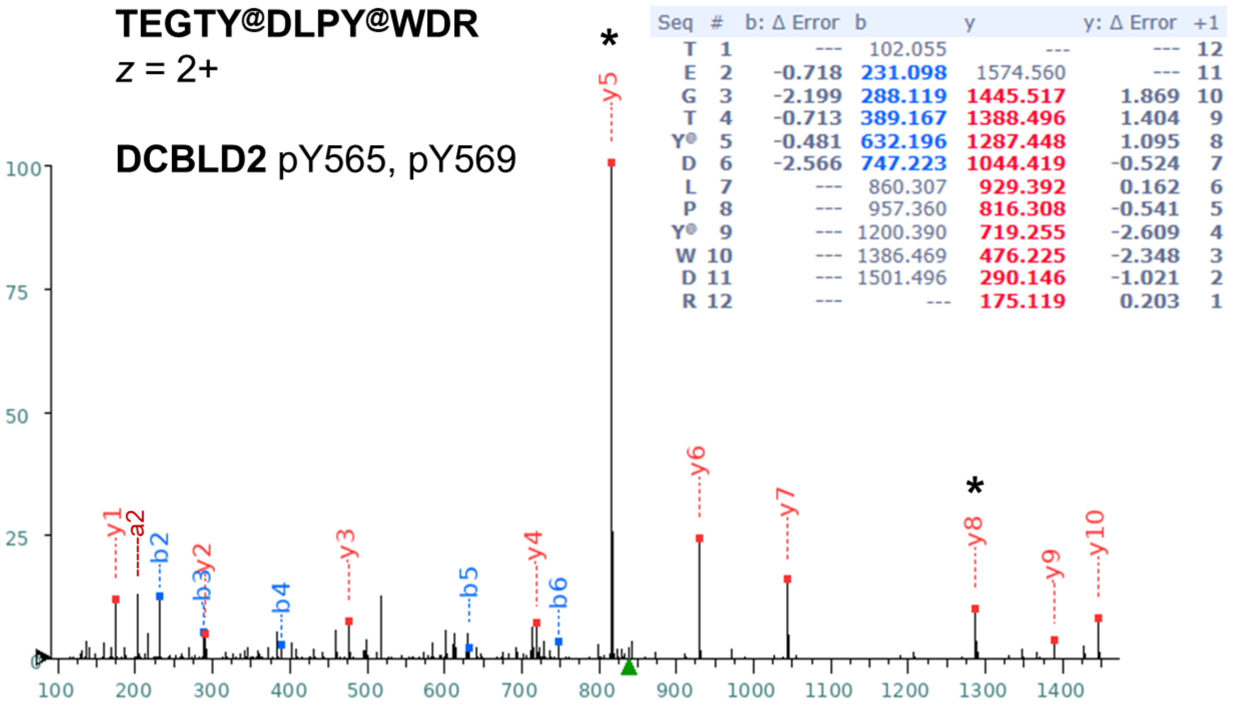

Seq	#	b: Δ Error	b	y	y: Δ Error	+1
T	1	---	102.055	---	---	12
E	2	-0.718	231.098	1574.560	---	11
G	3	-2.199	288.119	1445.517	1.869	10
T	4	-0.713	389.167	1388.496	1.404	9
Y@	5	-0.481	632.196	1287.448	1.095	8
D	6	-2.566	747.223	1044.419	-0.524	7
L	7	---	860.307	929.392	0.162	6
P	8	---	957.360	816.308	-0.541	5
Y@	9	---	1200.390	719.255	-2.609	4
W	10	---	1386.469	476.225	-2.348	3
D	11	---	1501.496	290.146	-1.021	2
R	12	---	---	175.119	0.203	1

ATGNQPPPLVGTYNTLLSR
z = 3+

DCBLD2 T714, Y715

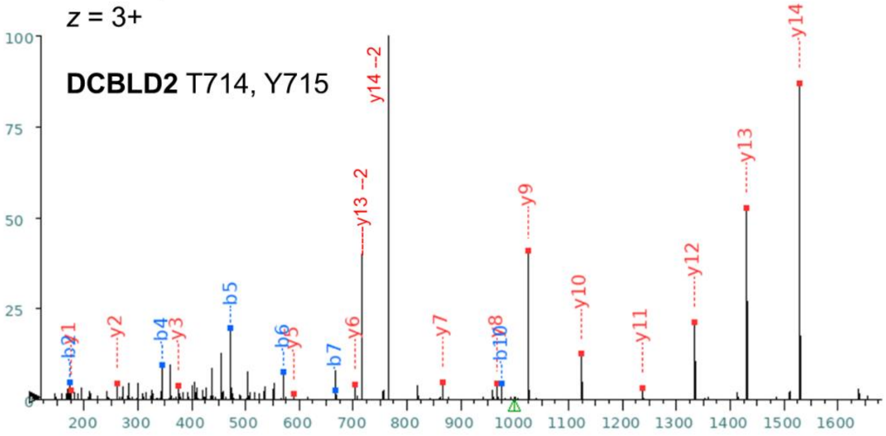

Seq	#	b: Δ Error	b	y	y: Δ Error	+1
A	1	---	72.044	---	---	19
T	2	1.165	173.092	1928.024	---	18
G	3	---	230.114	1826.976	---	17
N	4	-4.067	344.156	1769.954	---	16
Q	5	-0.538	472.215	1655.912	---	15
P	6	-2.214	569.268	1527.853	0.367	14
P	7	-0.745	666.321	1430.800	0.498	13
P	8	---	763.373	1333.747	-0.268	12
L	9	---	876.457	1236.695	0.920	11
V	10	-2.178	975.526	1123.611	-1.307	10
G	11	---	1032.547	1024.542	-0.784	9
T	12	---	1133.595	967.521	-0.095	8
Y	13	---	1296.658	866.473	0.399	7
N	14	---	1410.701	703.410	2.103	6
T	15	---	1511.749	589.367	-0.564	5
L	16	---	1624.833	488.319	---	4
L	17	---	1737.917	375.235	-1.541	3
S	18	---	1824.949	262.151	-0.040	2
R	19	---	---	175.119	-10.428	1

ATGNQPPPLVGT@YNTLLSR
z = 3+

DCBLD2 pT714

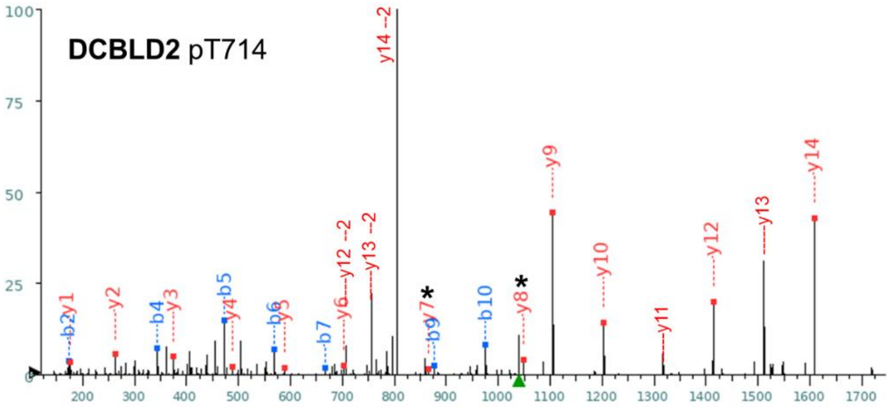

Seq	#	b: Δ Error	b	y	y: Δ Error	+1
A	1	---	72.044	---	---	19
T	2	-0.069	173.092	2007.990	---	18
G	3	---	230.114	1906.942	---	17
N	4	-2.471	344.156	1849.921	---	16
Q	5	-1.896	472.215	1735.878	---	15
P	6	-1.785	569.268	1607.819	0.867	14
P	7	4.110	666.321	1510.767	---	13
P	8	---	763.373	1413.714	2.063	12
L	9	-0.621	876.457	1316.661	---	11
V	10	-0.301	975.526	1203.577	-0.934	10
G	11	---	1032.547	1104.509	0.026	9
T@	12	---	1213.561	1047.487	0.241	8
Y	13	---	1376.625	866.473	-2.137	7
N	14	---	1490.668	703.410	1.842	6
T	15	---	1591.715	589.367	-4.603	5
L	16	---	1704.799	488.319	-4.222	4
L	17	---	1817.883	375.235	0.817	3
S	18	---	1904.915	262.151	-2.368	2
R	19	---	---	175.119	0.726	1

ATGNQPPPLVGTY@NTLLSR
z = 3+

DCBLD2 pY715

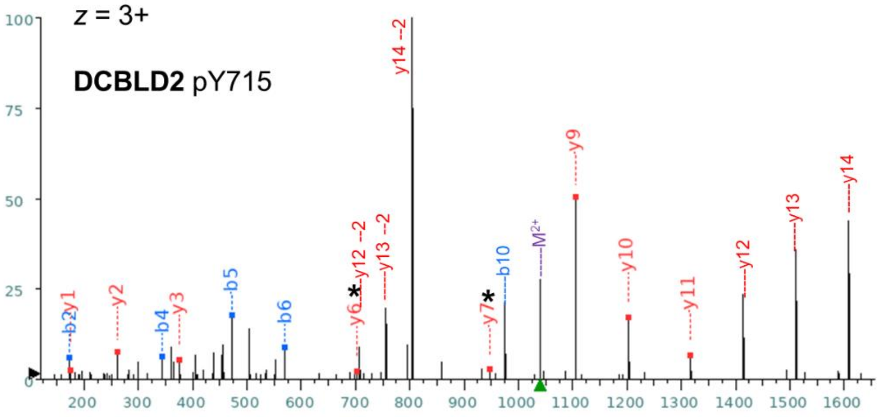

Seq	#	b: Δ Error	b	y	y: Δ Error	+1
A	1	---	72.044	---	---	19
T	2	1.518	173.092	2007.990	---	18
G	3	---	230.114	1906.942	---	17
N	4	2.495	344.156	1849.921	---	16
Q	5	-0.797	472.215	1735.878	---	15
P	6	-0.284	569.268	1607.819	---	14
P	7	---	666.321	1510.767	---	13
P	8	---	763.373	1413.714	---	12
L	9	---	876.457	1316.661	1.218	11
V	10	---	975.526	1203.577	1.094	10
G	11	---	1032.547	1104.509	0.800	9
T	12	---	1133.595	1047.487	---	8
Y@	13	---	1376.625	946.439	-1.528	7
N	14	---	1490.668	703.410	1.842	6
T	15	---	1591.715	589.367	---	5
L	16	---	1704.799	488.319	---	4
L	17	---	1817.883	375.235	-1.053	3
S	18	---	1904.915	262.151	-0.622	2
R	19	---	---	175.119	-1.017	1

AGKPGLPAPDELVYQVPQSTQEVSGAGR

z = 3+

DCBLD2 Y750, T756, S760

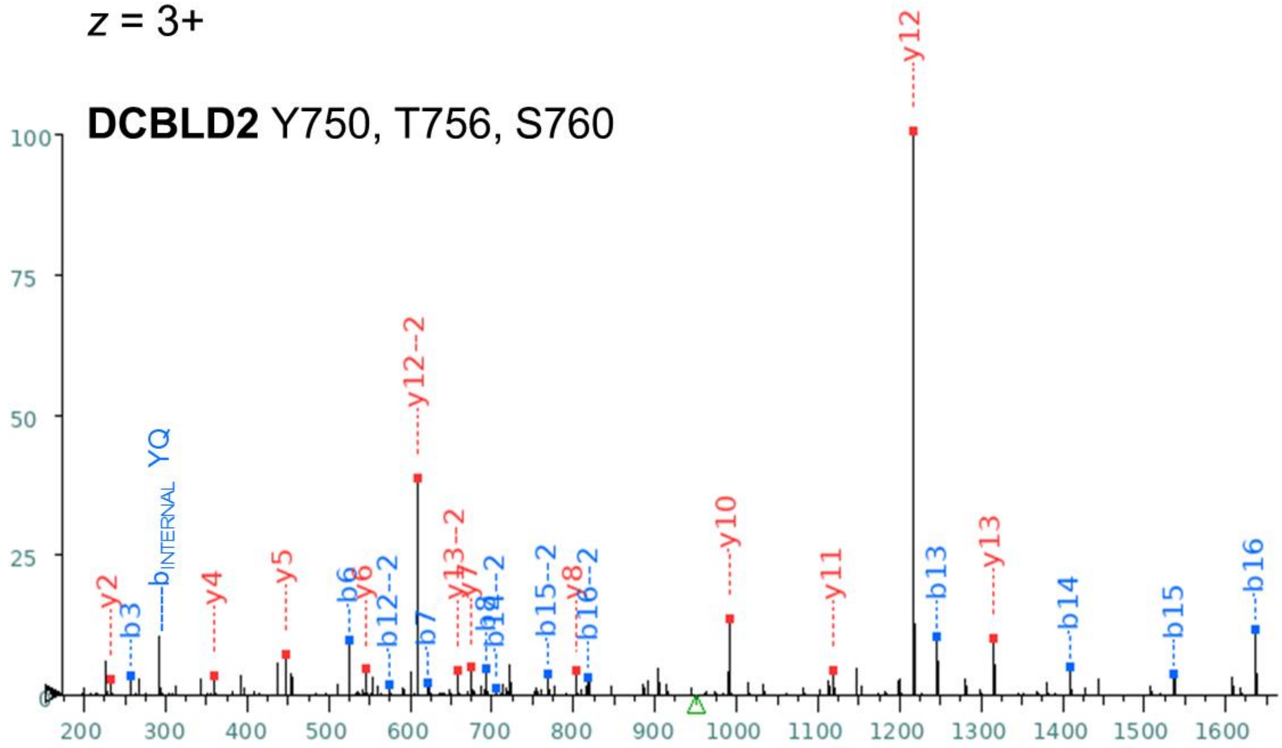

+1

Seq	#	b: Δ Error	b	y	y: Δ Error	+1
A	1	---	72.044	---	---	28
G	2	---	129.066	2780.421	---	27
K	3	-1.974	**257.161**	2723.400	---	26
P	4	---	354.214	2595.305	---	25
G	5	---	411.235	2498.252	---	24
L	6	-1.544	**524.319**	2441.231	---	23
P	7	4.641	**621.372**	2328.147	---	22
A	8	-2.012	**692.409**	2231.094	---	21
P	9	---	789.462	2160.057	---	20
D	10	---	904.489	2063.004	---	19
E	11	---	1033.531	1947.977	---	18
L	12	---	1146.615	1818.934	---	17
V	13	-2.096	**1245.684**	1705.850	---	16
Y	14	-0.622	**1408.747**	1606.782	---	15
Q	15	1.744	**1536.806**	1443.719	---	14
V	16	1.605	**1635.874**	**1315.660**	0.438	13
P	17	---	1732.927	**1216.592**	0.217	12
Q	18	---	1860.985	**1119.539**	0.153	11
S	19	---	1948.017	**991.480**	0.218	10
T	20	---	2049.065	904.448	---	9
Q	21	---	2177.124	**803.401**	-2.700	8
E	22	---	2306.166	**675.342**	-2.332	7
V	23	---	2405.235	**546.299**	-2.118	6
S	24	---	2492.267	**447.231**	0.332	5
G	25	---	2549.288	**360.199**	-2.171	4
A	26	---	2620.325	303.178	---	3
G	27	---	2677.347	**232.140**	0.505	2
R	28	---	---	175.119	---	1

+2

Seq	#	b: Δ Error	b	y	y: Δ Error	+1
A	1	---	36.526	---	---	28
G	2	---	65.037	1390.714	---	27
K	3	---	129.084	1362.204	---	26
P	4	---	177.610	1298.156	---	25
G	5	---	206.121	1249.630	---	24
L	6	---	262.663	1221.119	---	23
P	7	---	311.190	1164.577	---	22
A	8	---	346.708	1116.051	---	21
P	9	---	395.235	1080.532	---	20
D	10	---	452.748	1032.006	---	19
E	11	---	517.269	974.492	---	18
L	12	4.941	**573.811**	909.971	---	17
V	13	---	623.346	853.429	---	16
Y	14	1.491	**704.877**	803.895	---	15
Q	15	2.409	**768.906**	722.363	---	14
V	16	-3.587	**818.441**	**658.334**	0.288	13
P	17	---	866.967	**608.799**	-1.247	12
Q	18	---	930.996	560.273	---	11
S	19	---	974.512	496.244	---	10
T	20	---	1025.036	452.728	---	9
Q	21	---	1089.065	402.204	---	8
E	22	---	1153.587	338.175	---	7
V	23	---	1203.121	273.653	---	6
S	24	---	1246.637	224.119	---	5
G	25	---	1275.148	180.603	---	4
A	26	---	1310.666	152.092	---	3
G	27	---	1339.177	116.574	---	2
R	28	---	---	88.063	---	1

AGKPGLPAPDELVY@QVPQSTQEVSGAGR

z = 3+

DCBLD2 pY750

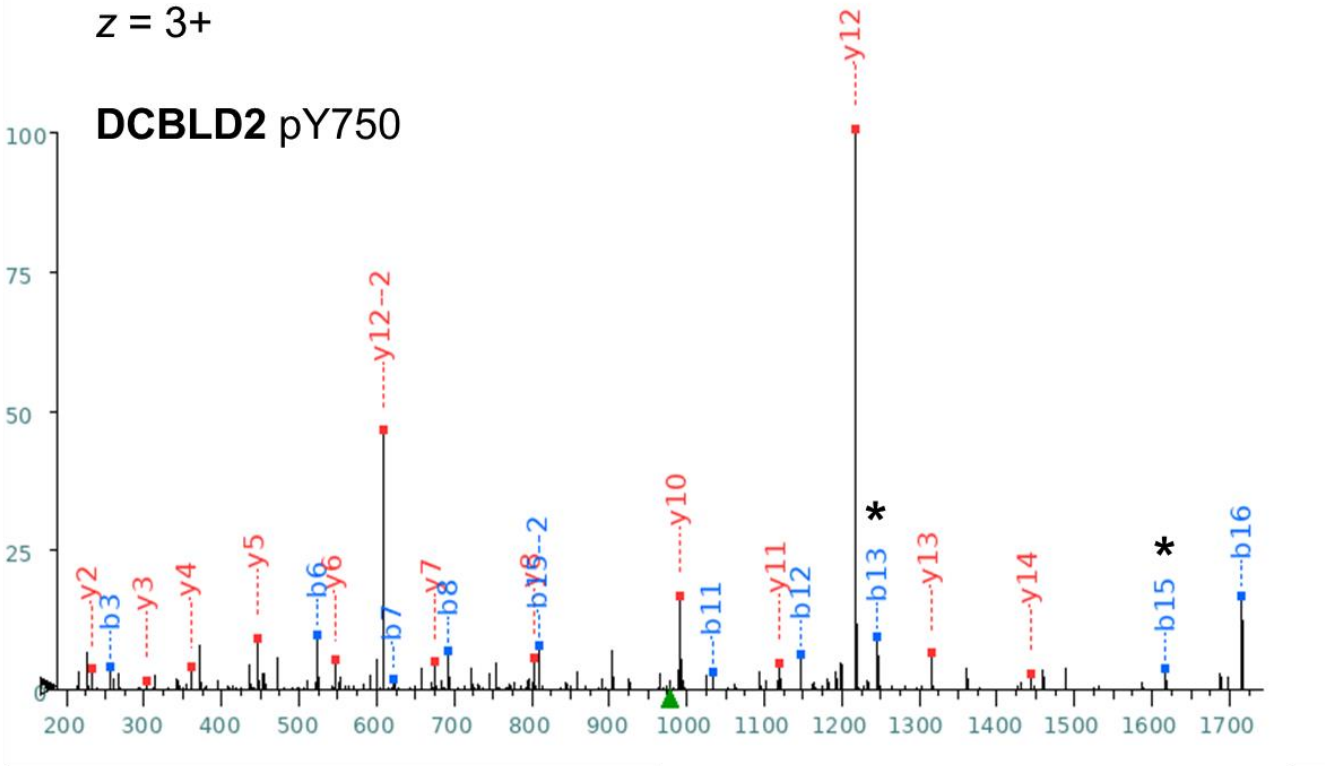

+1						
Seq	#	b: Δ Error	b	y	y: Δ Error	+1
A	1	---	72.044	---	---	28
G	2	---	129.066	2860.388	---	27
K	3	0.162	**257.161**	2803.366	---	26
P	4	---	354.214	2675.271	---	25
G	5	---	411.235	2578.218	---	24
L	6	-1.312	**524.319**	2521.197	---	23
P	7	-0.663	**621.372**	2408.113	---	22
A	8	-1.924	**692.409**	2311.060	---	21
P	9	---	789.462	2240.023	---	20
D	10	---	904.489	2142.970	---	19
E	11	-1.924	**1033.531**	2027.943	---	18
L	12	1.283	**1146.615**	1898.901	---	17
V	13	-1.018	**1245.684**	1785.817	---	16
Y®	14	---	1488.713	1686.748	---	15
Q	15	-0.923	**1616.772**	**1443.719**	-1.197	14
V	16	0.379	**1715.840**	**1315.660**	1.922	13
P	17	---	1812.893	**1216.592**	-0.586	12
Q	18	---	1940.952	**1119.539**	-1.046	11
S	19	---	2027.984	**991.480**	-0.582	10
T	20	---	2129.031	904.448	---	9
Q	21	---	2257.090	**803.401**	-1.560	8
E	22	---	2386.133	**675.342**	1.283	7
V	23	---	2485.201	**546.299**	-2.677	6
S	24	---	2572.233	**447.231**	-1.988	5
G	25	---	2629.255	**360.199**	-0.562	4
A	26	---	2700.292	**303.178**	-2.748	3
G	27	---	2757.313	**232.140**	-0.218	2
R	28	---	---	175.119	---	1

+2						
Seq	#	b: Δ Error	b	y	y: Δ Error	+1
A	1	---	36.526	---	---	28
G	2	---	65.037	1430.697	---	27
K	3	---	129.084	1402.187	---	26
P	4	---	177.610	1338.139	---	25
G	5	---	206.121	1289.613	---	24
L	6	---	262.663	1261.102	---	23
P	7	---	311.190	1204.560	---	22
A	8	---	346.708	1156.034	---	21
P	9	---	395.235	1120.515	---	20
D	10	---	452.748	1071.989	---	19
E	11	---	517.269	1014.475	---	18
L	12	---	573.811	949.954	---	17
V	13	---	623.346	893.412	---	16
Y®	14	---	744.860	843.878	---	15
Q	15	-2.402	**808.890**	722.363	---	14
V	16	---	858.424	658.334	---	13
P	17	---	906.950	**608.799**	-1.749	12
Q	18	---	970.980	560.273	---	11
S	19	---	1014.496	496.244	---	10
T	20	---	1065.019	452.728	---	9
Q	21	---	1129.049	402.204	---	8
E	22	---	1193.570	338.175	---	7
V	23	---	1243.104	273.653	---	6
S	24	---	1286.620	224.119	---	5
G	25	---	1315.131	180.603	---	4
A	26	---	1350.649	152.092	---	3
G	27	---	1379.160	116.574	---	2
R	28	---	---	88.063	---	1

AGKPGLPAPDELVYQVPQST@QEVSGAGR

z = 3+

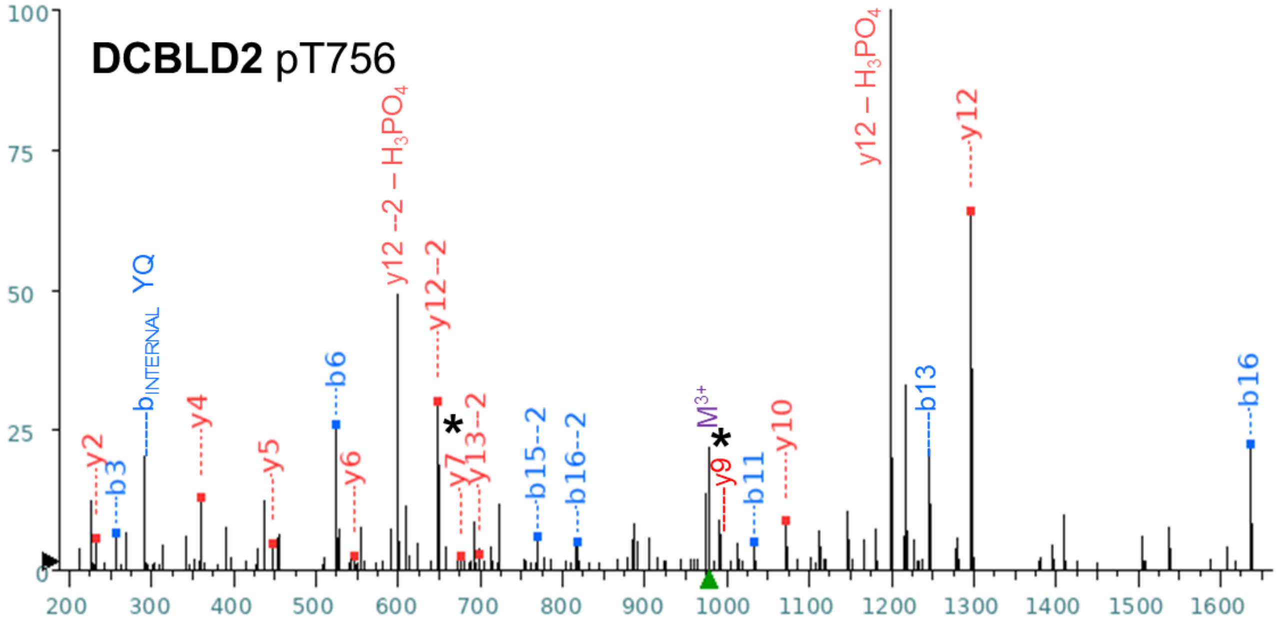

+1

Seq	#	b: Δ Error	b	y	y: Δ Error	+1
A	1	---	72.044	---	---	28
G	2	---	129.066	2860.388	---	27
K	3	0.043	**257.161**	2803.366	---	26
P	4	---	354.214	2675.271	---	25
G	5	---	411.235	2578.218	---	24
L	6	0.900	**524.319**	2521.197	---	23
P	7	---	621.372	2408.113	---	22
A	8	---	692.409	2311.060	---	21
P	9	---	789.462	2240.023	---	20
D	10	---	904.489	2142.970	---	19
E	11	-0.625	**1033.531**	2027.943	---	18
L	12	---	1146.615	1898.901	---	17
V	13	---	1245.684	1785.817	---	16
Y	14	---	1408.747	1686.748	---	15
Q	15	---	1536.806	1523.685	---	14
V	16	1.456	**1635.874**	1395.626	---	13
P	17	---	1732.927	**1296.558**	1.034	12
Q	18	---	1860.985	1199.505	---	11
S	19	---	1948.017	**1071.447**	-2.040	10
T@	20	---	2129.031	984.415	---	9
Q	21	---	2257.090	803.401	---	8
E	22	---	2386.133	**675.342**	3.000	7
V	23	---	2485.201	**546.299**	0.228	6
S	24	---	2572.233	**447.231**	2.993	5
G	25	---	2629.255	**360.199**	1.811	4
A	26	---	2700.292	303.178	---	3
G	27	---	2757.313	**232.140**	-2.387	2
R	28	---	---	175.119	---	1

+2

Seq	#	b: Δ Error	b	y	y: Δ Error	+1
A	1	---	36.526	---	---	28
G	2	---	65.037	1430.697	---	27
K	3	---	129.084	1402.187	---	26
P	4	---	177.610	1338.139	---	25
G	5	---	206.121	1289.613	---	24
L	6	---	262.663	1261.102	---	23
P	7	---	311.190	1204.560	---	22
A	8	---	346.708	1156.034	---	21
P	9	---	395.235	1120.515	---	20
D	10	---	452.748	1071.989	---	19
E	11	---	517.269	1014.475	---	18
L	12	---	573.811	949.954	---	17
V	13	---	623.346	893.412	---	16
Y	14	---	704.877	843.878	---	15
Q	15	0.425	**768.906**	762.346	---	14
V	16	3.423	**818.441**	**698.317**	1.043	13
P	17	---	866.967	**648.783**	0.036	12
Q	18	---	930.996	600.256	---	11
S	19	---	974.512	536.227	---	10
T@	20	---	1065.019	492.711	---	9
Q	21	---	1129.049	402.204	---	8
E	22	---	1193.570	338.175	---	7
V	23	---	1243.104	273.653	---	6
S	24	---	1286.620	224.119	---	5
G	25	---	1315.131	180.603	---	4
A	26	---	1350.649	152.092	---	3
G	27	---	1379.160	116.574	---	2
R	28	---	---	88.063	---	1

AGKPGLPAPDELVY@QVPQSTQEVS@GAGR
z = 3+

DCBLD2 pY750, pS760

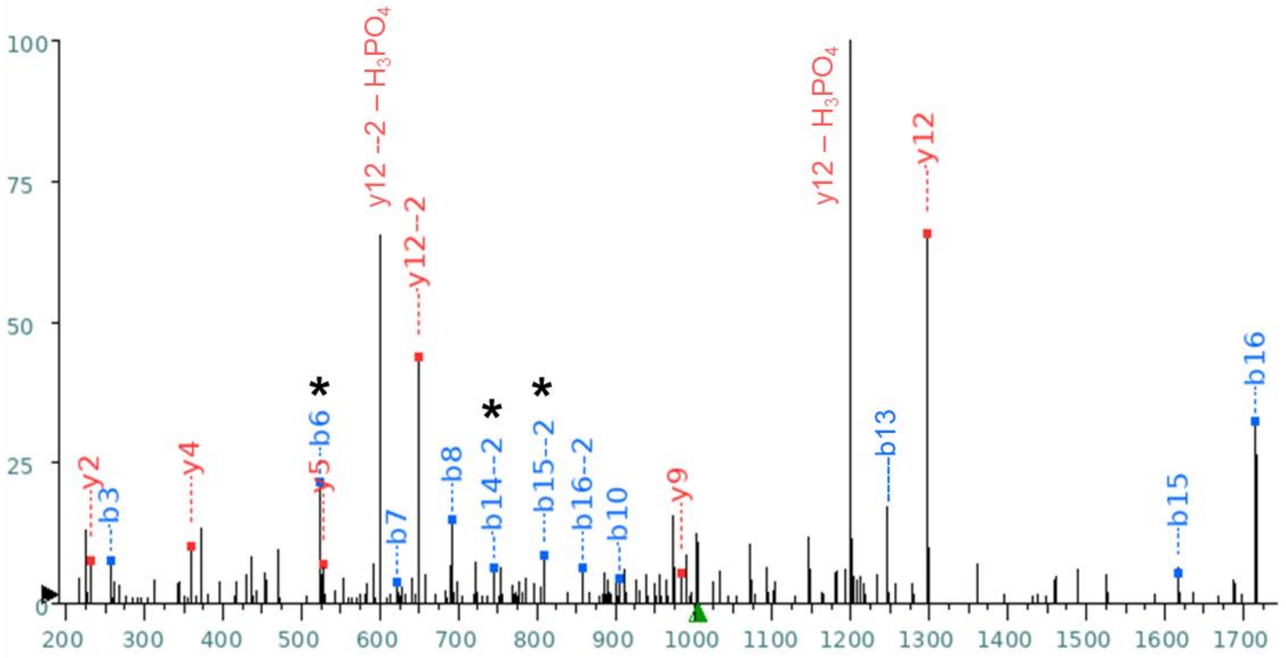

+1

Seq	#	b: Δ Error	b	y	y: Δ Error	+1
A	1	---	72.044	---	---	28
G	2	---	129.066	2940.354	---	27
K	3	-0.431	**257.161**	2883.333	---	26
P	4	---	354.214	2755.238	---	25
G	5	---	411.235	2658.185	---	24
L	6	-0.264	**524.319**	2601.163	---	23
P	7	3.561	**621.372**	2488.079	---	22
A	8	-0.690	**692.409**	2391.027	---	21
P	9	---	789.462	2319.989	---	20
D	10	-2.344	**904.489**	2222.937	---	19
E	11	---	1033.531	2107.910	---	18
L	12	---	1146.615	1978.867	---	17
V	13	---	1245.684	1865.783	---	16
Y®	14	---	1488.713	1766.715	---	15
Q	15	-1.829	**1616.772**	1523.685	---	14
V	16	0.877	**1715.840**	1395.626	---	13
P	17	---	1812.893	**1296.558**	0.846	12
Q	18	---	1940.952	1199.505	---	11
S	19	---	2027.984	1071.447	---	10
T	20	---	2129.031	**984.415**	-0.500	9
Q	21	---	2257.090	883.367	---	8
E	22	---	2386.133	755.308	---	7
V	23	---	2485.201	626.266	---	6
S®	24	---	2652.199	**527.197**	-1.786	5
G	25	---	2709.221	**360.199**	-0.900	4
A	26	---	2780.258	303.178	---	3
G	27	---	2837.279	**232.140**	-0.021	2
R	28	---	---	175.119	---	1

+2

Seq	#	b: Δ Error	b	y	y: Δ Error	+1
A	1	---	36.526	---	---	28
G	2	---	65.037	1470.681	---	27
K	3	---	129.084	1442.170	---	26
P	4	---	177.610	1378.122	---	25
G	5	---	206.121	1329.596	---	24
L	6	---	262.663	1301.085	---	23
P	7	---	311.190	1244.543	---	22
A	8	---	346.708	1196.017	---	21
P	9	---	395.235	1160.498	---	20
D	10	---	452.748	1111.972	---	19
E	11	---	517.269	1054.458	---	18
L	12	---	573.811	989.937	---	17
V	13	---	623.346	933.395	---	16
Y®	14	0.904	**744.860**	883.861	---	15
Q	15	-1.044	**808.890**	762.346	---	14
V	16	2.682	**858.424**	698.317	---	13
P	17	---	906.950	**648.783**	-0.999	12
Q	18	---	970.980	600.256	---	11
S	19	---	1014.496	536.227	---	10
T	20	---	1065.019	492.711	---	9
Q	21	---	1129.049	442.187	---	8
E	22	---	1193.570	378.158	---	7
V	23	---	1243.104	313.637	---	6
S®	24	---	1326.603	264.102	---	5
G	25	---	1355.114	180.603	---	4
A	26	---	1390.633	152.092	---	3
G	27	---	1419.143	116.574	---	2
R	28	---	---	88.063	---	1

VYHAYAEPLPITGPE
z = 3+

DCBLD2 Y663

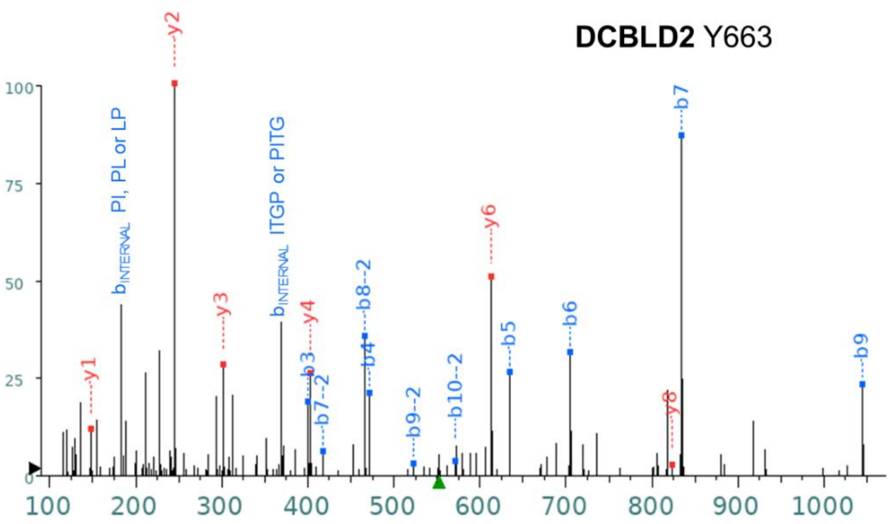

+1

Seq	#	b: Δ Error	b	y	y: Δ Error	+1
V	1	---	100.076	---	---	15
Y	2	---	263.139	1557.758	---	14
H	3	6.193	**400.198**	1394.695	---	13
A	4	2.207	**471.235**	1257.636	---	12
Y	5	-2.746	**634.298**	1186.599	---	11
A	6	-0.312	**705.335**	1023.536	---	10
E	7	-0.472	**834.378**	952.499	---	9
P	8	---	931.431	**823.456**	2.665	8
L	9	-1.066	**1044.515**	726.403	---	7
P	10	---	1141.568	**613.319**	-1.120	6
I	11	---	1254.652	516.266	---	5
T	12	---	1355.699	**403.182**	-2.190	4
G	13	---	1412.721	**302.135**	-3.092	3
P	14	---	1509.774	**245.113**	-0.658	2
E	15	---	---	**148.060**	-0.891	1

+2

Seq	#	b: Δ Error	b	y	y: Δ Error	+1
V	1	---	50.541	---	---	15
Y	2	---	132.073	779.383	---	14
H	3	---	200.603	697.851	---	13
A	4	---	236.121	629.322	---	12
Y	5	---	317.653	593.803	---	11
A	6	---	353.171	512.271	---	10
E	7	-3.775	**417.693**	476.753	---	9
P	8	-5.574	**466.219**	412.232	---	8
L	9	-4.521	**522.761**	363.705	---	7
P	10	-1.492	**571.287**	307.163	---	6
I	11	---	627.830	258.637	---	5
T	12	---	678.353	202.095	---	4
G	13	---	706.864	151.571	---	3
P	14	---	755.390	123.060	---	2
E	15	---	---	74.534	---	1

VY@HAYAEPLPITGPE
z = 3+

DCBLD2 pY663

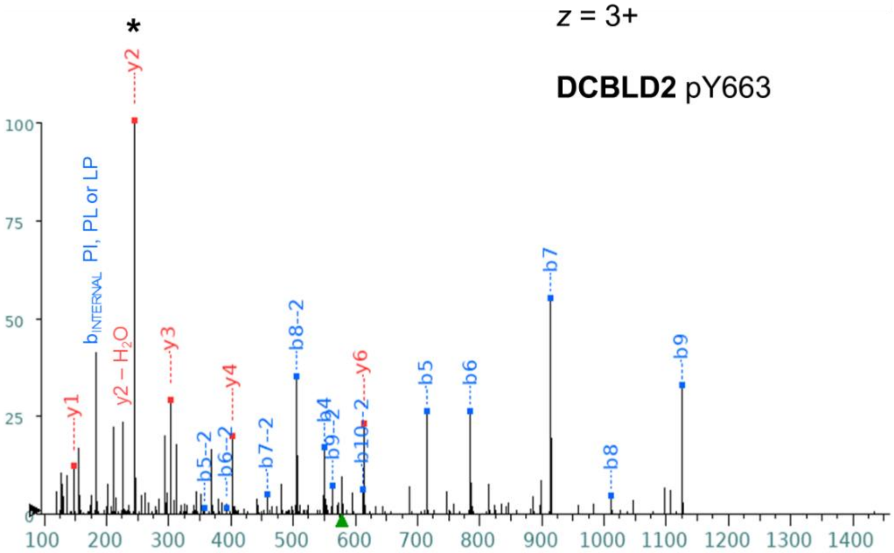

+1

Seq	#	b: Δ Error	b	y	y: Δ Error	+1
V	1	---	100.076	---	---	15
Y@	2	---	343.105	1637.725	---	14
H	3	---	480.164	1394.695	---	13
A	4	-0.589	**551.201**	1257.636	---	12
Y	5	-1.700	**714.265**	1186.599	---	11
A	6	1.635	**785.302**	1023.536	---	10
E	7	-0.989	**914.344**	952.499	---	9
P	8	2.879	**1011.397**	823.456	---	8
L	9	-0.250	**1124.481**	726.403	---	7
P	10	---	1221.534	**613.319**	-1.717	6
I	11	---	1334.618	516.266	---	5
T	12	---	1435.666	**403.182**	-2.342	4
G	13	---	1492.687	**302.135**	-2.082	3
P	14	---	1589.740	**245.113**	-0.969	2
E	15	---	---	**148.060**	0.243	1

+2

Seq	#	b: Δ Error	b	y	y: Δ Error	+1
V	1	---	50.541	---	---	15
Y@	2	---	172.056	819.366	---	14
H	3	---	240.586	697.851	---	13
A	4	---	276.104	629.322	---	12
Y	5	-2.654	**357.636**	593.803	---	11
A	6	7.360	**393.155**	512.271	---	10
E	7	-1.135	**457.676**	476.753	---	9
P	8	-2.804	**506.202**	412.232	---	8
L	9	-2.376	**562.744**	363.705	---	7
P	10	-1.113	**611.271**	307.163	---	6
I	11	---	667.813	258.637	---	5
T	12	---	718.337	202.095	---	4
G	13	---	746.847	151.571	---	3
P	14	---	795.374	123.060	---	2
E	15	---	---	74.534	---	1

VYHAYAEPLPITGPEYATPIIMDMSGHPTTSVGQPSTSTFK

z = 4+

DCBLD2 Y677, T679

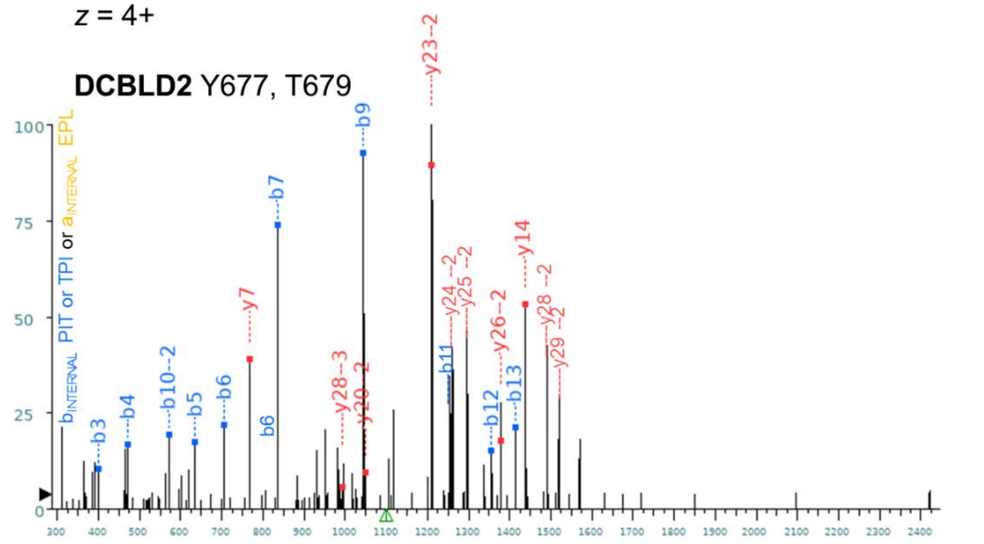

+1

Seq	#	b: Δ Error	b	y	y: Δ Error	+1
V	1	---	100.076	---	---	41
Y	2	---	263.139	4293.052	---	40
H	3	---	400.198	4129.989	---	39
A	4	---	471.235	3992.930	---	38
Y	5	-1.784	**634.298**	3921.893	---	37
A	6	---	705.335	3758.829	---	36
E	7	0.918	**834.378**	3687.792	---	35
P	8	---	931.431	3558.749	---	34
L	9	-2.001	**1044.515**	3461.697	---	33
P	10	---	1141.568	3348.613	---	32
I	11	---	1254.652	3251.560	---	31
T	12	---	1355.699	3138.476	---	30
G	13	1.086	**1412.721**	3037.428	---	29
P	14	---	1509.774	2980.407	---	28
E	15	---	1638.816	2883.354	---	27
Y	16	---	1801.880	2754.311	---	26
A	17	---	1872.917	2591.248	---	25
T	18	---	1973.964	2520.211	---	24
P	19	---	2071.017	2419.163	---	23
I	20	---	2184.101	2322.110	---	22
I	21	---	2297.185	2209.026	---	21
M	22	---	2428.226	2095.942	---	20
D	23	---	2543.253	1964.902	---	19
M	24	---	2674.293	1849.875	---	18
S	25	---	2761.325	1718.834	---	17
G	26	---	2818.347	1631.802	---	16
H	27	---	2955.406	1574.781	---	15
P	28	---	3052.458	1437.722	---	14
T	29	---	3153.506	1340.669	---	13
T	30	---	3254.554	1239.622	---	12
S	31	---	3341.586	1138.574	---	11
V	32	---	3440.654	1051.542	---	10
G	33	---	3497.676	**952.473**	0.398	9
Q	34	---	3625.734	895.452	---	8
P	35	---	3722.787	767.393	---	7
S	36	---	3809.819	670.341	---	6
T	37	---	3910.867	583.309	---	5
S	38	---	3997.899	482.261	---	4
T	39	---	4098.946	395.229	---	3
F	40	---	4246.015	294.181	---	2
K	41	---	---	147.113	---	1

+2

Seq	#	b: Δ Error	b	y	y: Δ Error	+1
V	1	---	50.541	---	---	41
Y	2	---	132.073	2147.030	---	40
H	3	---	200.603	2065.498	---	39
A	4	---	236.121	1996.968	---	38
Y	5	---	317.653	1961.450	---	37
A	6	---	353.171	1879.918	---	36
E	7	---	417.693	1844.400	---	35
P	8	-1.188	**466.219**	1779.878	---	34
L	9	---	522.761	1731.352	---	33
P	10	1.286	**571.287**	1674.810	---	32
I	11	---	627.830	1626.284	---	31
T	12	---	678.353	1569.742	---	30
G	13	---	706.864	1519.218	---	29
P	14	---	755.390	**1490.707**	-0.621	28
E	15	---	819.912	1442.181	---	27
Y	16	---	901.443	1377.659	---	26
A	17	---	936.962	1296.128	---	25
T	18	---	987.486	**1260.609**	1.008	24
P	19	---	1036.012	**1210.085**	2.289	23
I	20	---	1092.554	1161.559	---	22
I	21	---	1149.096	1105.017	---	21
M	22	---	1214.617	1048.475	---	20
D	23	---	1272.130	982.955	---	19
M	24	---	1337.650	925.441	---	18
S	25	---	1381.166	859.921	---	17
G	26	---	1409.677	816.405	---	16
H	27	---	1478.206	787.894	---	15
P	28	---	1526.733	719.365	---	14
T	29	---	1577.257	670.838	---	13
T	30	---	1627.780	620.314	---	12
S	31	---	1671.296	569.791	---	11
V	32	---	1720.831	526.275	---	10
G	33	---	1749.341	476.740	---	9
Q	34	---	1813.371	448.230	---	8
P	35	---	1861.897	384.200	---	7
S	36	---	1905.413	335.674	---	6
T	37	---	1955.937	292.158	---	5
S	38	---	1999.453	241.634	---	4
T	39	---	2049.977	198.118	---	3
F	40	---	2123.511	147.594	---	2
K	41	---	---	74.060	---	1

+3

Seq	#	b: Δ Error	b	y	y: Δ Error	+1
V	1	---	34.030	---	---	41
Y	2	---	88.385	1431.689	---	40
H	3	---	134.071	1377.334	---	39
A	4	---	157.750	1331.648	---	38
Y	5	---	212.104	1307.969	---	37
A	6	---	235.783	1253.615	---	36
E	7	---	278.798	1229.936	---	35
P	8	---	311.148	1186.921	---	34
L	9	---	348.843	1154.570	---	33
P	10	---	381.194	1116.876	---	32
I	11	---	418.889	1084.525	---	31
T	12	---	452.571	1046.830	---	30
G	13	---	471.578	1013.148	---	29
P	14	---	503.929	994.140	---	28
E	15	---	546.944	961.789	---	27
Y	16	---	601.298	918.775	---	26
A	17	---	624.977	864.421	---	25
T	18	---	658.660	840.742	---	24
P	19	---	691.011	807.059	---	23
I	20	---	728.705	774.708	---	22
I	21	---	766.400	737.014	---	21
M	22	---	810.080	699.319	---	20
D	23	---	848.422	655.639	---	19
M	24	---	892.103	617.296	---	18
S	25	---	921.113	573.616	---	17
G	26	---	940.120	544.606	---	16
H	27	---	985.807	525.598	---	15
P	28	---	1018.158	479.912	---	14
T	29	---	1051.840	447.561	---	13
T	30	---	1085.523	413.879	---	12
S	31	---	1114.533	380.196	---	11
V	32	---	1147.556	351.185	---	10
G	33	---	1166.563	318.163	---	9
Q	34	---	1209.250	299.156	---	8
P	35	---	1241.600	256.469	---	7
S	36	---	1270.611	224.118	---	6
T	37	---	1304.294	195.108	---	5
S	38	---	1333.304	161.425	---	4
T	39	---	1366.987	132.414	---	3
F	40	---	1416.010	98.732	---	2
K	41	---	---	49.709	---	1

VYHAYAEPLPITGPEY@ATPIIMDMSGHPTTSVGQPSTSTFK

z = 4+

DCBLD2 pY677

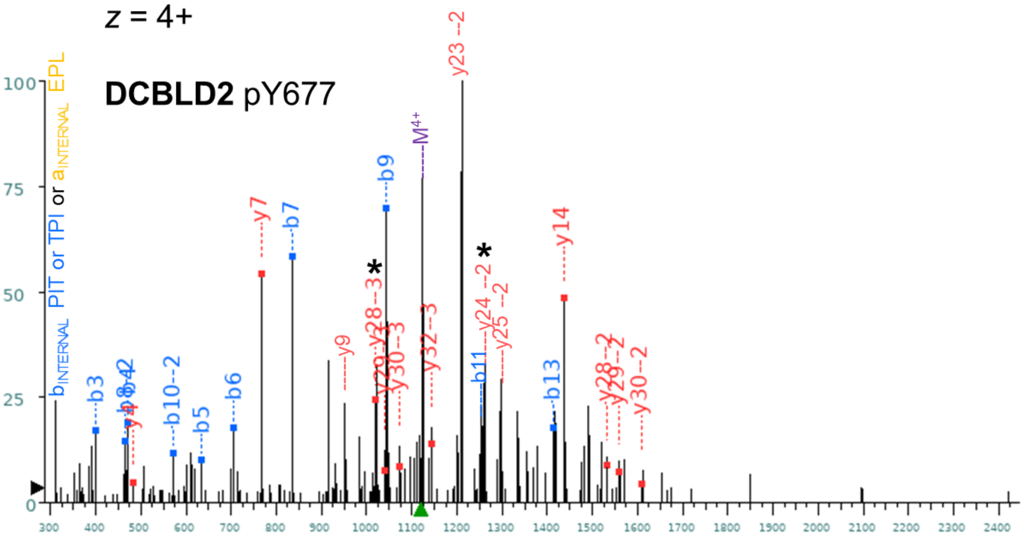

+1

Seq	#	b: Δ Error	b	y	y: Δ Error	+1
V	1	---	100.076	---	---	41
Y	2	---	263.139	4373.018	---	40
H	3	5.126	**400.198**	4209.955	---	39
A	4	-2.262	**471.235**	4072.896	---	38
Y	5	1.488	**634.298**	4001.859	---	37
A	6	-1.524	**705.335**	3838.796	---	36
E	7	-1.057	**834.378**	3767.758	---	35
P	8	---	931.431	3638.716	---	34
L	9	-2.001	**1044.515**	3541.663	---	33
P	10	---	1141.568	3428.579	---	32
I	11	---	1254.652	3331.526	---	31
T	12	---	1355.699	3218.442	---	30
G	13	-1.938	**1412.721**	3117.394	---	29
P	14	---	1509.774	3060.373	---	28
E	15	---	1638.816	2963.320	---	27
Y@	16	---	1881.846	2834.278	---	26
A	17	---	1952.883	2591.248	---	25
T	18	---	2053.931	2520.211	---	24
P	19	---	2150.983	2419.163	---	23
I	20	---	2264.068	2322.110	---	22
I	21	---	2377.152	2209.026	---	21
M	22	---	2508.192	2095.942	---	20
D	23	---	2623.219	1964.902	---	19
M	24	---	2754.259	1849.875	---	18
S	25	---	2841.292	1718.834	---	17
G	26	---	2898.313	1631.802	---	16
H	27	---	3035.372	1574.781	---	15
P	28	---	3132.425	**1437.722**	1.815	14
T	29	---	3233.472	1340.669	---	13
T	30	---	3334.520	1239.622	---	12
S	31	---	3421.552	1138.574	---	11
V	32	---	3520.620	1051.542	---	10
G	33	---	3577.642	952.473	---	9
Q	34	---	3705.700	895.452	---	8
P	35	---	3802.753	**767.393**	-1.066	7
S	36	---	3889.785	670.341	---	6
T	37	---	3990.833	583.309	---	5
S	38	---	4077.865	**482.261**	1.835	4
T	39	---	4178.913	395.229	---	3
F	40	---	4325.981	294.181	---	2
K	41	---	---	147.113	---	1

+2

Seq	#	b: Δ Error	b	y	y: Δ Error	+1
V	1	---	50.541	---	---	41
Y	2	---	132.073	2187.013	---	40
H	3	---	200.603	2105.481	---	39
A	4	---	236.121	2036.952	---	38
Y	5	---	317.653	2001.433	---	37
A	6	---	353.171	1919.901	---	36
E	7	---	417.693	1884.383	---	35
P	8	-4.854	**466.219**	1819.862	---	34
L	9	---	522.761	1771.335	---	33
P	10	3.529	**571.287**	1714.793	---	32
I	11	---	627.830	1666.267	---	31
T	12	---	678.353	**1609.725**	1.677	30
G	13	---	706.864	**1559.201**	-0.360	29
P	14	---	755.390	**1530.690**	1.541	28
E	15	---	819.912	1482.164	---	27
Y@	16	---	941.427	1417.642	---	26
A	17	---	976.945	1296.128	---	25
T	18	---	1027.469	1260.609	---	24
P	19	---	1075.995	1210.085	---	23
I	20	---	1132.537	1161.559	---	22
I	21	---	1189.079	1105.017	---	21
M	22	---	1254.600	1048.475	---	20
D	23	---	1312.113	982.955	---	19
M	24	---	1377.633	925.441	---	18
S	25	---	1421.149	859.921	---	17
G	26	---	1449.660	816.405	---	16
H	27	---	1518.190	787.894	---	15
P	28	---	1566.716	719.365	---	14
T	29	---	1617.240	670.838	---	13
T	30	---	1667.764	620.314	---	12
S	31	---	1711.280	569.791	---	11
V	32	---	1760.814	526.275	---	10
G	33	---	1789.325	476.740	---	9
Q	34	---	1853.354	448.230	---	8
P	35	---	1901.880	384.200	---	7
S	36	---	1945.396	335.674	---	6
T	37	---	1995.920	292.158	---	5
S	38	---	2039.436	241.634	---	4
T	39	---	2089.960	198.118	---	3
F	40	---	2163.494	147.594	---	2
K	41	---	---	74.060	---	1

+3

Seq	#	b: Δ Error	b	y	y: Δ Error	+1
V	1	---	34.030	---	---	41
Y	2	---	88.385	1458.344	---	40
H	3	---	134.071	1403.990	---	39
A	4	---	157.750	1358.304	---	38
Y	5	---	212.104	1334.624	---	37
A	6	---	235.783	1280.270	---	36
E	7	---	278.798	1256.591	---	35
P	8	---	311.148	1213.577	---	34
L	9	---	348.843	1181.226	---	33
P	10	---	381.194	**1143.531**	-1.860	32
I	11	---	418.889	1111.180	---	31
T	12	---	452.571	**1073.486**	2.184	30
G	13	---	471.578	**1039.803**	0.204	29
P	14	---	503.929	**1020.796**	1.536	28
E	15	---	546.944	988.445	---	27
Y@	16	---	627.953	945.431	---	26
A	17	---	651.633	864.421	---	25
T	18	---	685.315	840.742	---	24
P	19	---	717.666	807.059	---	23
I	20	---	755.361	774.708	---	22
I	21	---	793.055	737.014	---	21
M	22	---	836.736	699.319	---	20
D	23	---	875.078	655.639	---	19
M	24	---	918.758	617.296	---	18
S	25	---	947.769	573.616	---	17
G	26	---	966.776	544.606	---	16
H	27	---	1012.462	525.598	---	15
P	28	---	1044.813	479.912	---	14
T	29	---	1078.496	447.561	---	13
T	30	---	1112.178	413.879	---	12
S	31	---	1141.189	380.196	---	11
V	32	---	1174.212	351.185	---	10
G	33	---	1193.219	318.163	---	9
Q	34	---	1235.905	299.156	---	8
P	35	---	1268.256	256.469	---	7
S	36	---	1297.267	224.118	---	6
T	37	---	1330.949	195.108	---	5
S	38	---	1359.960	161.425	---	4
T	39	---	1393.642	132.414	---	3
F	40	---	1442.665	98.732	---	2
K	41	---	---	49.709	---	1

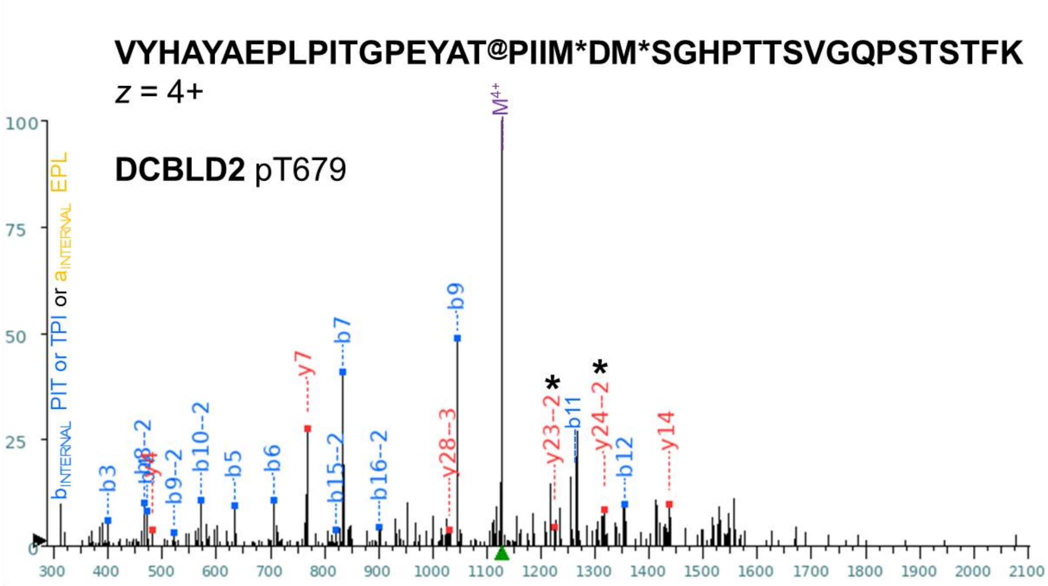

+1

Seq	#	b: Δ Error	b	y	y: Δ Error	+1
V	1	---	100.076	---	---	41
Y	2	---	263.139	4405.008	---	40
H	3	3.448	**400.198**	4241.945	---	39
A	4	-1.096	**471.235**	4104.886	---	38
Y	5	-1.399	**634.298**	4033.849	---	37
A	6	-2.043	**705.335**	3870.785	---	36
E	7	0.552	**834.378**	3799.748	---	35
P	8	---	931.431	3670.706	---	34
L	9	1.505	**1044.515**	3573.653	---	33
P	10	---	1141.568	3460.569	---	32
I	11	---	1254.652	3363.516	---	31
T	12	-1.674	**1355.699**	3250.432	---	30
G	13	---	1412.721	3149.384	---	29
P	14	---	1509.774	3092.363	---	28
E	15	---	1638.816	2995.310	---	27
Y	16	---	1801.880	2866.267	---	26
A	17	---	1872.917	2703.204	---	25
T@	18	---	2053.931	2632.167	---	24
P	19	---	2150.983	2451.153	---	23
I	20	---	2264.068	2354.100	---	22
I	21	---	2377.152	2241.016	---	21
M*	22	---	2524.187	2127.932	---	20
D	23	---	2639.214	1980.897	---	19
M*	24	---	2786.249	1865.870	---	18
S	25	---	2873.281	1718.834	---	17
G	26	---	2930.303	1631.802	---	16
H	27	---	3067.362	1574.781	---	15
P	28	---	3164.414	**1437.722**	-2.006	14
T	29	---	3265.462	1340.669	---	13
T	30	---	3366.510	1239.622	---	12
S	31	---	3453.542	1138.574	---	11
V	32	---	3552.610	1051.542	---	10
G	33	---	3609.632	952.473	---	9
Q	34	---	3737.690	895.452	---	8
P	35	---	3834.743	**767.393**	0.286	7
S	36	---	3921.775	670.341	---	6
T	37	---	4022.823	583.309	---	5
S	38	---	4109.855	**482.261**	4.240	4
T	39	---	4210.903	395.229	---	3
F	40	---	4357.971	294.181	---	2
K	41	---	---	147.113	---	1

+2

Seq	#	b: Δ Error	b	y	y: Δ Error	+1
V	1	---	50.541	---	---	41
Y	2	---	132.073	2203.008	---	40
H	3	---	200.603	2121.476	---	39
A	4	---	236.121	2052.947	---	38
Y	5	---	317.653	2017.428	---	37
A	6	---	353.171	1935.896	---	36
E	7	---	417.693	1900.378	---	35
P	8	-3.086	**466.219**	1835.856	---	34
L	9	1.083	**522.761**	1787.330	---	33
P	10	-1.172	**571.287**	1730.788	---	32
I	11	---	627.830	1682.262	---	31
T	12	---	678.353	1625.720	---	30
G	13	---	706.864	1575.196	---	29
P	14	---	755.390	1546.685	---	28
E	15	2.665	**819.912**	1498.159	---	27
Y	16	-0.608	**901.443**	1433.637	---	26
A	17	---	936.962	1352.106	---	25
T@	18	---	1027.469	**1316.587**	1.574	24
P	19	---	1075.995	**1226.080**	0.334	23
I	20	---	1132.537	1177.554	---	22
I	21	---	1189.079	1121.012	---	21
M*	22	---	1262.597	1064.470	---	20
D	23	---	1320.111	990.952	---	19
M*	24	---	1393.628	933.439	---	18
S	25	---	1437.144	859.921	---	17
G	26	---	1465.655	816.405	---	16
H	27	---	1534.184	787.894	---	15
P	28	---	1582.711	719.365	---	14
T	29	---	1633.235	670.838	---	13
T	30	---	1683.759	620.314	---	12
S	31	---	1727.275	569.791	---	11
V	32	---	1776.809	526.275	---	10
G	33	---	1805.320	476.740	---	9
Q	34	---	1869.349	448.230	---	8
P	35	---	1917.875	384.200	---	7
S	36	---	1961.391	335.674	---	6
T	37	---	2011.915	292.158	---	5
S	38	---	2055.431	241.634	---	4
T	39	---	2105.955	198.118	---	3
F	40	---	2179.489	147.594	---	2
K	41	---	---	74.060	---	1

+3

Seq	#	b: Δ Error	b	y	y: Δ Error	+1
V	1	---	34.030	---	---	41
Y	2	---	88.385	1469.008	---	40
H	3	---	134.071	1414.653	---	39
A	4	---	157.750	1368.967	---	38
Y	5	---	212.104	1345.288	---	37
A	6	---	235.783	1290.933	---	36
E	7	---	278.798	1267.254	---	35
P	8	---	311.148	1224.240	---	34
L	9	---	348.843	1191.889	---	33
P	10	---	381.194	1154.194	---	32
I	11	---	418.889	1121.844	---	31
T	12	---	452.571	1084.149	---	30
G	13	---	471.578	1050.466	---	29
P	14	---	503.929	**1031.459**	-2.156	28
E	15	---	546.944	999.108	---	27
Y	16	---	601.298	956.094	---	26
A	17	---	624.977	901.740	---	25
T@	18	---	685.315	878.061	---	24
P	19	---	717.666	817.723	---	23
I	20	---	755.361	785.372	---	22
I	21	---	793.055	747.677	---	21
M*	22	---	842.067	709.982	---	20
D	23	---	880.409	660.970	---	19
M*	24	---	929.421	622.628	---	18
S	25	---	958.432	573.616	---	17
G	26	---	977.439	544.606	---	16
H	27	---	1023.125	525.598	---	15
P	28	---	1055.476	479.912	---	14
T	29	---	1089.159	447.561	---	13
T	30	---	1122.841	413.879	---	12
S	31	---	1151.852	380.196	---	11
V	32	---	1184.875	351.185	---	10
G	33	---	1203.882	318.163	---	9
Q	34	---	1246.568	299.156	---	8
P	35	---	1278.919	256.469	---	7
S	36	---	1307.930	224.118	---	6
T	37	---	1341.612	195.108	---	5
S	38	---	1370.623	161.425	---	4
T	39	---	1404.306	132.414	---	3
F	40	---	1453.328	98.732	---	2
K	41	---	---	49.709	---	1
