# Supplementary material for: FYN and ABL Regulate the Interaction Networks of the DCBLD Receptor Family
Source: Mol Cell Proteomics. 2020 Nov 25;19(10):1586–601. doi: 10.1074/mcp.RA120.002163 (PMC8015000; doi:10.1074/mcp.RA120.002163)
Supplement: Supplementary file 1 [file mmc1.zip › 161393_0_supp_544325_qbm5fb.pdf]

SUPPLEMENTARY FIGURES

Figure S1. Coomassie-stained gels from two experimental replicates of SILAC immunoprecipitations for the identification of DCBLD1 and DCBLD2 interacting proteins.

Figure S2. Abl-induced interactors of DCBLD proteins common among human and zebrafish.

Figure S3. The GST-14-3-3 fusion constructs bind canonical (mode 1) 14-3-3 binding motif.

Figure S4. 14-3-3 family members bind to the tyrosine kinase Abl.

Figure S5. The Calmodulin dependent kinase 2 (CaMKII) substrate motif.

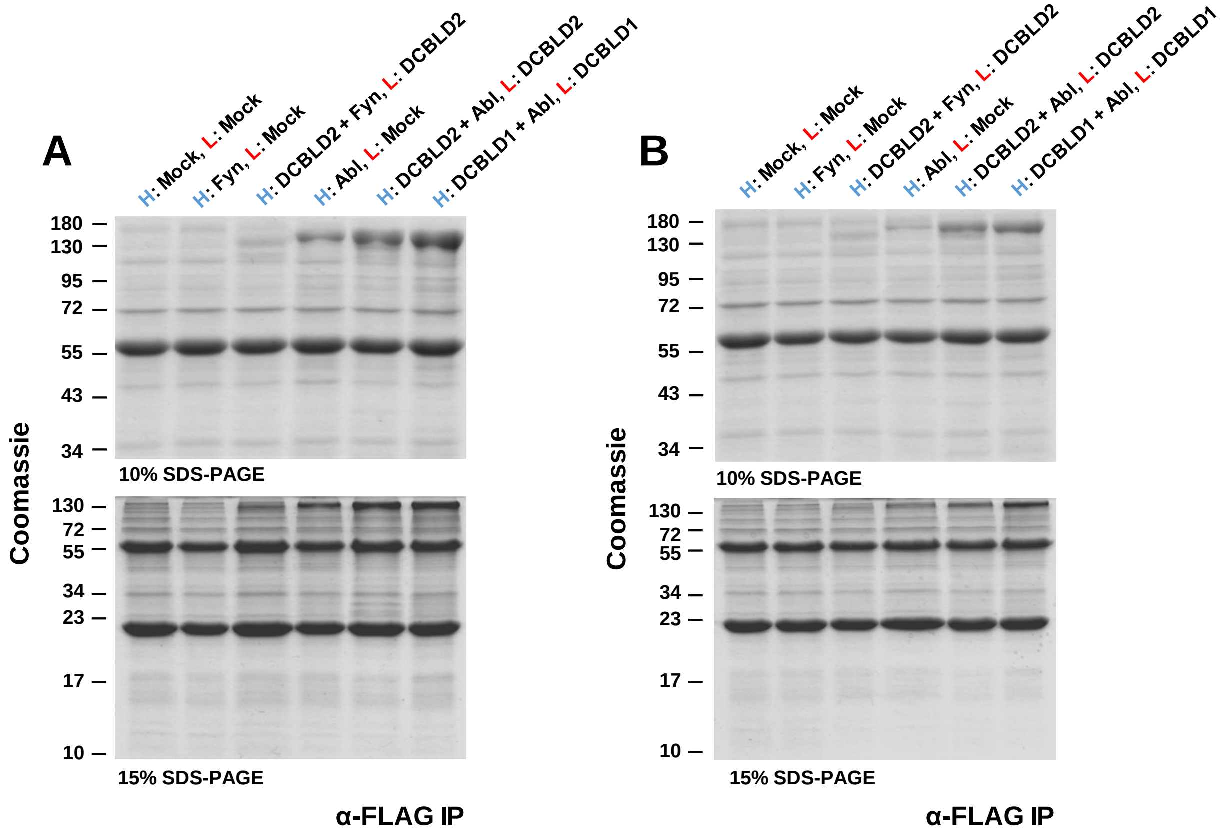

Figure S1. Coomassie-stained gels from two experimental replicates of SILAC immunoprecipitations for the identification of DCBLD1 and DCBLD2 interacting proteins. A,B) 293 cells grown in DMEM supplemented with arginine and lysine containing heavy (H, blue) or light (L, red) stable isotopes of carbon and nitrogen were transiently transfected with plasmids encoding DCBLD family members with or without the tyrosine kinases Fyn and Abl. Immunoprecipitations (α-FLAG) from heavy and light experimental pairs were denatured and combined immediately prior to SDS-PAGE and Coomassie staining. Experimental heavy/light pairs are indicated at the top of each gel lane. To ensure both maximum separation and identification of both high and low molecular weight interactors, heavy/light pairs were split across 10% and 15% gels. Each was analyzed separately via LC-MS/MS. A cut-map of each gel type and the Coomassie-stained gel from the third experimental replicate are included in Figure 2.

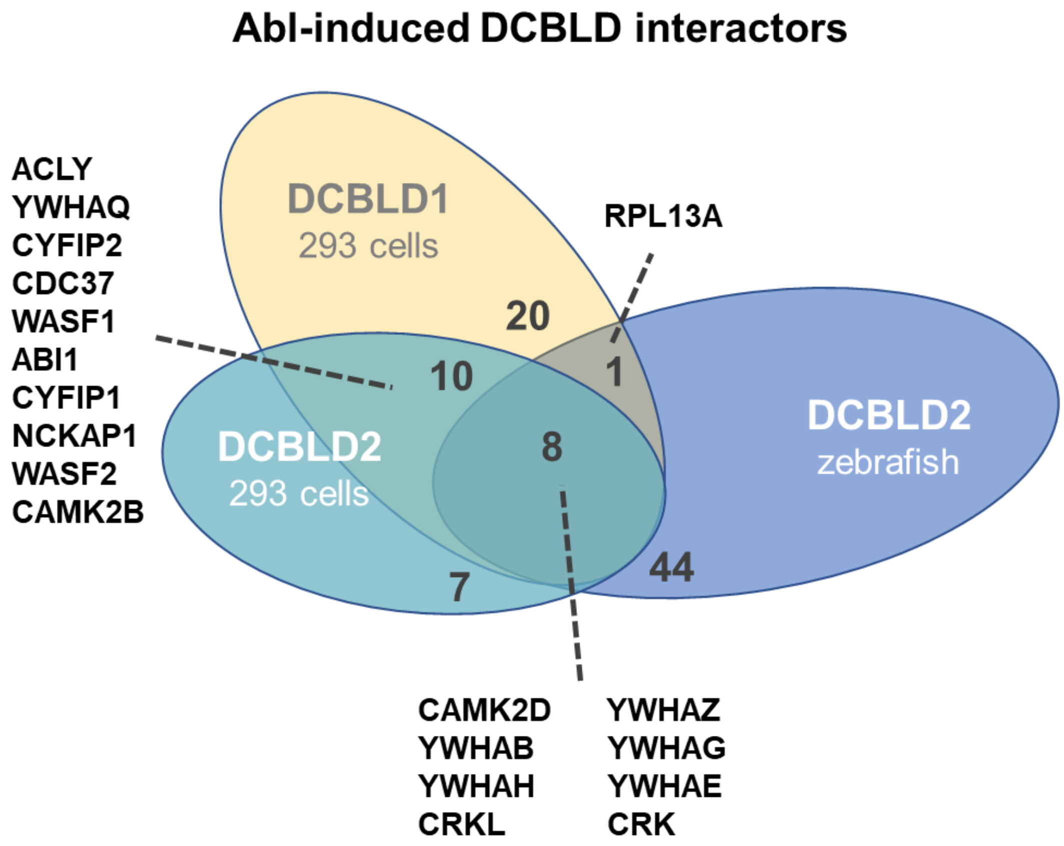

Figure S2. Abl-induced interactors of DCBLD proteins common among human and zebrafish. A Venn diagram displays the overlap of Abl-induced interactors of DCBLD1 and DCBLD2 in human cells (HEK 293) to Abl-induced DCBLD2 interactors in zebrafish lysates. Human DCBLD1 and DCBLD2 interactors were common to the three biological replicates used for the SILAC analysis in Figure 3. Zebrafish DCBLD2 interactors were identified by incubating DCBLD2 immunoprecipitations from 293 cells expressing DCBLD2 alone or DCBLD2 and Abl with zebrafish lysates. Only unique zebrafish peptides were used to identify zebrafish proteins in immune complex with DCBLD2. Zebrafish proteins that were unique to the condition in which Abl was co-expressed with DCBLD2 are represented in the Venn diagram. The full list of zebrafish DCBLD2 interactors are included in Table S8.

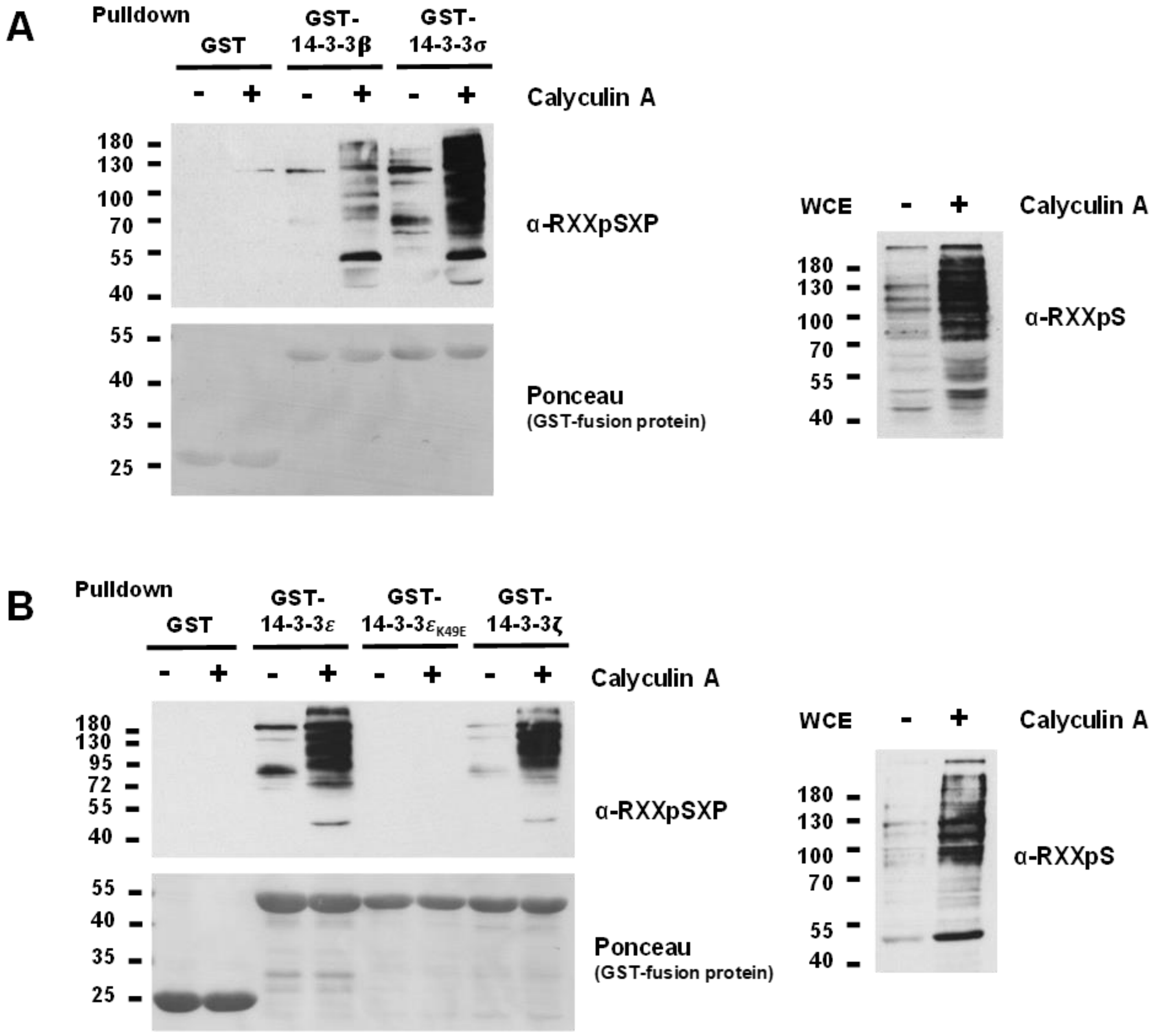

Figure S3. The GST-14-3-3 fusion constructs bind canonical (mode 1) 14-3-3 binding motif. A,B) GST ad GST-14-3-3 fusion proteins were purified from bacterial extracts on glutathione resin and incubated with 293 extracts from cells either left untreated or treated with calyculin A, a serine/threonine phosphatase inhibitor, prior to lysis. Whole cell extracts (WCEs) show an induction of serine/threonine phosphorylation in Akt substrate motifs (α-RXXpS/pT) following calyculin A treatment. Pulldowns demonstrate increased binding of proteins containing the canonical (mode 1) 14-3-3 substrate motif (RXXpSXP) in 293 cells treated with calyculin A. Pulldowns with GST alone and GST-14-3-3ε_{K49E}, which is unable to bind proteins in a phosphorylation-dependent manner, served as controls. Ponceau staining shows levels of GST and GST-fusions.

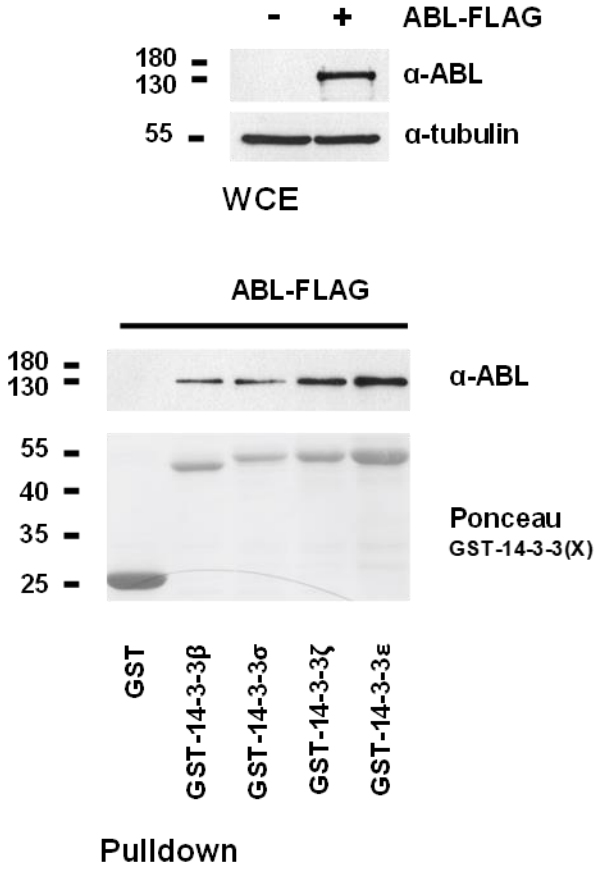

Figure S4. 14-3-3 family members bind to the tyrosine kinase Abl. GST and GST-14-3-3 fusion proteins were purified from bacterial extracts on glutathione resin and incubated with 293 cell extracts from cells transiently expressing ABL-FLAG in a pulldown assay. All four GST fusion with 14-3-3 family members (β,σ,ε,ζ) bound specifically to Abl. Ponceau staining shows levels of GST and GST fusions. Whole cell extracts (WCEs) show expression of ABL-FLAG (α-ABL). The α-tubulin serves as a loading control.

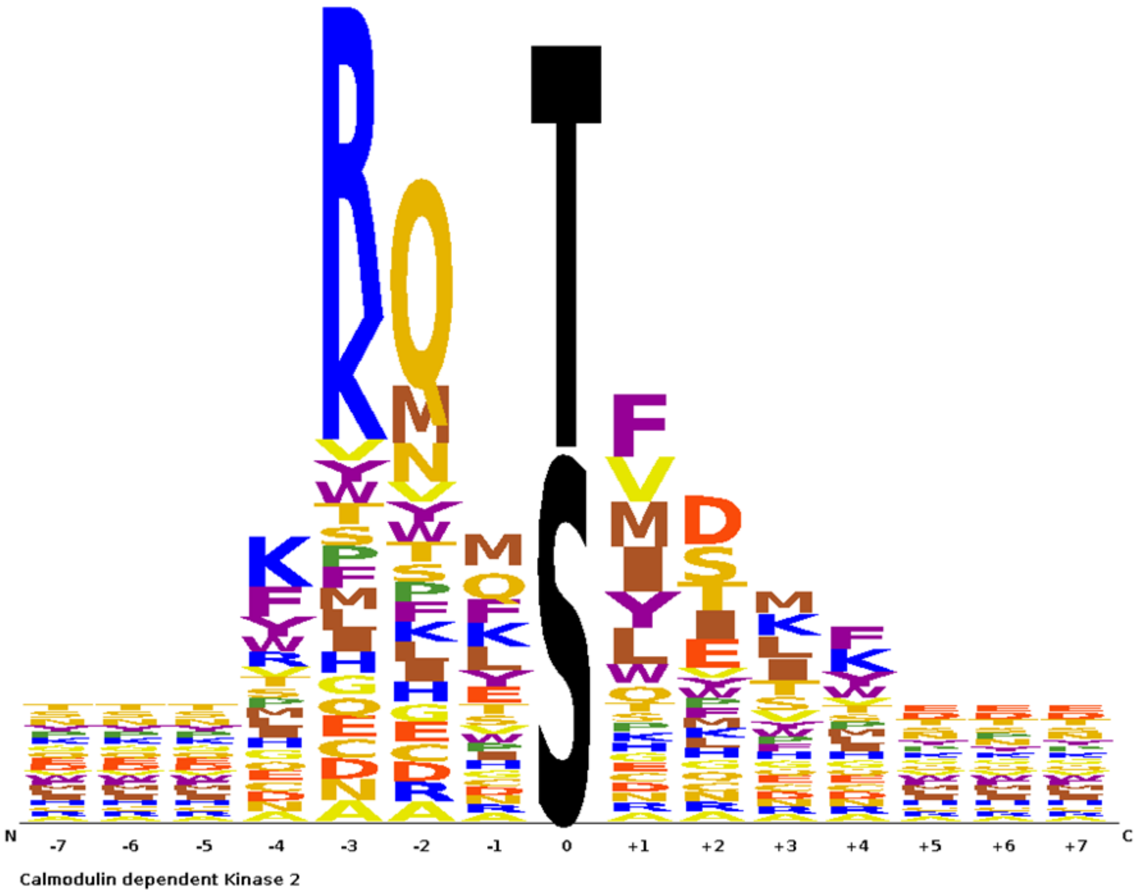

Figure S5. The Calmodulin dependent kinase 2 (CaMKII) substrate motif. Motif logo was reproduced from Scansite 4.0 (scansite4.mit.edu) (1).

REFERENCES

1. Obenauer, J. C., Cantley, L. C., and Yaffe, M. B. (2003) Scansite 2.0: Proteome-wide prediction of cell signaling interactions using short sequence motifs. *Nucleic acids research* 31, 3635-3641
